# New Biobased Polyester Polyols With Tunable *T*_g_ Values From 4‐Vinylguaiacol‐Derived Monomers

**DOI:** 10.1002/cssc.70901

**Published:** 2026-07-23

**Authors:** Erika Zangelmi, Orlando Santoro, Raffaele Cucciniello, Francesco Della Monica, Lorella Izzo

**Affiliations:** ^1^ Department of Biotechnology and Life Science University of Insubria Varese Italy; ^2^ Department of Chemistry and Biology “Adolfo Zambelli” University of Salerno Fisciano Italy

**Keywords:** biopolymers, epoxides, polyesters, polyol, ring‐opening polymerization

## Abstract

Polyesters (PEs) are among the most important classes of plastics. The identification of new, biobased, and renewable sources is necessary to increase the sustainability of PEs and related materials production. Herein, we describe the synthesis of three epoxy monomers derived from 4‐vinylguaiacol, a functional molecule easily obtained from biobased ferulic acid. These monomers, namely 4‐epoxyguaiacol acetate, butanoate, and hexanoate, were used in ring‐opening copolymerization reactions in combination with different cyclic anhydrides, promoted by simple, (organo)catalytic initiators [i.e., bis‐(triphenylphosphine)iminium chloride, tetrabutylammonium bromide, 4‐(dimethylamino)pyridine, and cesium acetate]. Reactions were carried out with and without solvents, allowing the synthesis of 12 new and structurally different PEs containing up to 100% renewable monomers. The obtained PEs have number‐average molecular weights (*M*_*n*_) in the range of 1.8–4.1 kDa, and dispersity indexes (*Đ*) in the range of 1.09–1.62. Only for maleic anhydride, higher *M*_*n*_ and *Đ* values were observed, likely due to side reactions involving the double bond on the anhydride backbone. Thermal analyses revealed that, depending on the PE structure, glass transition temperatures can be modulated between 0 and 78 °C, while mass analyses confirmed the presence of hydroxy‐terminated polymer chains, supporting their application as low molecular‐weight polyester polyols.

## Graphical abstract

Biobased low‐molecular‐weight polyesters were obtained by ROCOP of guaiacol‐derived epoxides with cyclic anhydrides. Varying the length of the acyl moieties on the epoxides and the cyclic anhydride structures, it is possible to tune the *T*_g_'s within an 80 ºC wide temperature range. Such polyesters present hydroxyl chain ends, supporting their applications to increase the sustainability of polymer formulations such as polyurethanes.

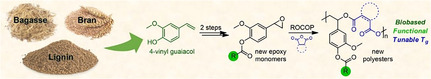

## 1 Introduction

Synthetic polymer materials have had tremendous relevance in the development of virtually all technological sectors of modern society, supported by the availability of cheap, petroleum‐based commodity chemicals such as ethylene, propylene, styrene, and their oxides[1, 2]. However, the use of long‐lasting materials led to environmental pollution due to (micro)plastics accumulation [3, 4, 5]. In addition, the depletion of fossil resources and their availability depending on geopolitical issues will likely lead to an increase in the cost of raw materials, making their use for the production of commodity polymers undesirable and uneconomic. Thus, the synthesis of new, sustainable biopolymers capable to replace current petroleum‐based plastics is attracting more and more attention [6, 7, 8, 9]. For this purpose, the application of sustainable catalytic polymerization processes is pivotal. Indeed, new functional monomers can be prepared from biobased molecules allowing the application of techniques such as radical [10, 11, 12], anionic [13], cationic [14], coordination–insertion [15], and ring‐opening (co)polymerization [RO(CO)P] [16, 17]. Moreover, the identification of new, renewable, and more sustainable raw materials derived from biomass for the synthesis of biopolymers is essential. For example, lignin‐based [18, 19, 20], sugar‐based [21, 22], and terpene‐based building‐blocks [23, 24] are very promising and have focused the efforts of many researchers.

**SCHEME 1:**
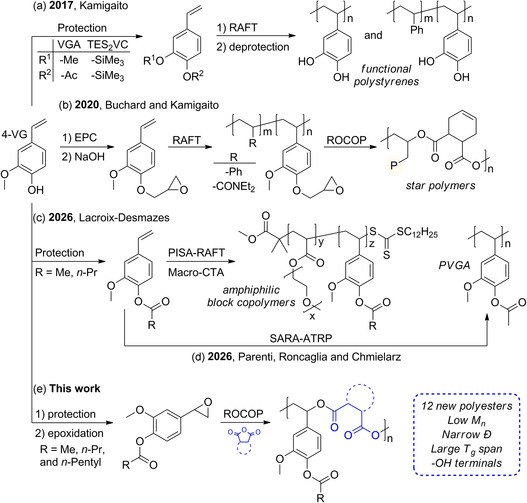
Comparison between examples of 4‐vinylguaiacol (4‐VG) derived functional polystyrenes (a), star polymers (b), amphiphilic block copolymers (c) obtained via RAFT, poly(vinylguaiacol acetate) made via ATRP (d), and polyesters obtained in this work (e).

In this scenario, we selected 4‐vinylguaiacol (4‐VG) as a biobased platform molecule for the production of new biopolymers with potential industrial applications. The 4‐VG can be obtained from ferulic acid (FA), a naturally occurring phenolic acid, via biocatalytic processes [25, 26, 27]. In turn, FA can be recovered from several renewable resources such as bagasse, beetroot pulp, lignin, and wheat bran [28, 29], setting a concrete starting point for future application of envisaged new bio‐plastics [30]. The 4‐VG is a multifunctional molecule and its chemical structure presents a phenolic group and an olefin group, offering the opportunity for chemical modifications aimed at producing biobased monomers and biopolymers thereof (Scheme 1).

However, despite 4‐VG potential applications in polymer chemistry, its functionalization and polymerization are relatively underexplored. For example, in 2017, Kamigaito reported the synthesis of several functional polystyrenes by reversible addition–fragmentation chain transfer (RAFT) polymerization of protected 4‐VG‐based monomers, followed by deprotection to regenerate phenol groups (Scheme 1a) [31]. Later on, Buchard and Kamigaito adopted a sequential combination of RAFT and ROCOP procedures for the synthesis of star polymers starting from a multifunctional monomer obtained by reaction of 4‐VG with epichlorohydrin (EPC, Scheme 1b) [32]. Very recently, Lacroix–Desmazes described the use of polymerization induced self‐assembly RAFT (PISA‐RAFT) emulsion polymerization of 4‐VG acylates for the synthesis of amphiphilic block copolymers with different morphology (Scheme 1c) [33], while Chmielarz et al. reported the supplementary activator and reducing agent (SARA) ATRP of 4‐vinylguaiacol acetate (4‐VGA) toward poly(vinylguaiacol acetate) (PVGA, Scheme 1d) [34]. To the best of our knowledge, all other reports of 4‐VG‐derived monomer applications are based on the double‐bond polymerization via cationic polymerization [35], or free [36, 37, 38, 39], emulsion [40], suspension [41], and controlled radical polymerization techniques [42].

Based on our previous experience on the preparation of biobased epoxides [43, 44, 45], and supported by the previously reported epoxidation of 4‐VGA [46], we envisaged the possibility to convert 4‐VG into functional, styrene oxide‐like monomers and to prepare polyesters (PEs) thereof by ROCOP with cyclic anhydrides (CAs), allowing the facile modulation of structural rigidity by tuning the length of the acyl moieties and the structure of CAs backbones (Scheme 1e). The synthesis of PEs by ROCOP has been largely investigated using both metal‐based [47, 48, 49, 50] and metal‐free catalytic systems [51], and is considered an effective and green methodology completely fulfilling the atom‐economy principle [17]. Notwithstanding that ROCOP reactions often proceed with a good degree of control (dispersity index, *Đ* < 1.30), the formation of PEs with high molecular weights (MW > 10 kDa) is difficult because of the presence of adventitious water or hydrolyzed CAs in the reaction system, resulting in MWs significantly lower than expected [49]. This issue is known to affect ROCOP of epoxides with different monomers [52, 53, 54, 55, 56], and is highly relevant for biobased epoxides such as limonene oxide, in which protic impurities other than water have been demonstrated to limit the formation of high MW polymers [57]. However, low MW polyols are highly useful for polyurethanes (PUs) production [58], and the synthesis of polyester polyols from renewables is required to increase the sustainability of PUs manufacturing [59, 60, 61, 62]. All these aspects are highly relevant to the object of this work since styrene oxide (SO), which is structurally close to the guaiacol‐based epoxides we report, generally reacts in ROCOP with the formation of low MW PEs [63, 64, 65, 66, 67, 68]. In fact, this behavior was attributed also to the acid‐catalyzed formation of phenylacetaldehyde followed by tautomerization to its enol form, thus increasing the concentration of initiating species [69, 70]. Notably, the synthesis of poly(styrene oxide‐*alt*‐phthalic anhydride) with high MW (MW up to 178.4 kDa) was reported only recently by Wang, via a CO_2_‐mediated methodology based on styrene carbonate and promoted by bis(triphenylphosphine)‐iminium salts [71].

Based on this analysis, we envisaged the possibility of obtaining biobased low MW PE polyols from 4‐VG. Thus, here we describe the synthesis of styrene oxide‐like epoxy monomers and their use in ROCOP with various CAs promoted by simple and commercial initiators. Thanks to the modulation of comonomer structural motifs, thermal properties can be tuned, offering an attractive alternative to petroleum‐based polyols currently employed in PUs chemistry.

## 2 Results and Discussion

### 2.1 Synthesis of Epoxy Monomers From 4‐Vinylguaiacol

**SCHEME 2:**
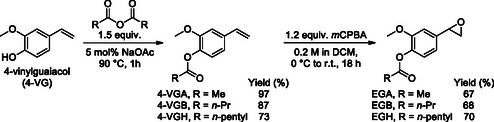
Two‐step procedure for the synthesis of epoxy monomers from 4‐vinylguaiacol.

**FIGURE 1:**
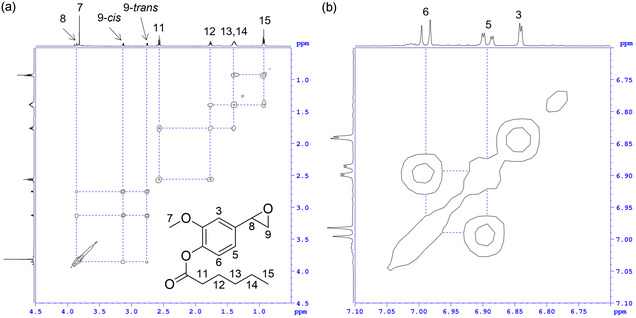
^1^H−^1^H COSY NMR spectrum of EGH in the selected spectral regions from 4.5 to 0.5 ppm (a) and from 7.1 to 6.7 ppm (b) (600 MHz, CDCl_3_, room temperature). Blue dotted lines highlight relevant correlation patterns.

In order to obtain the desired epoxy monomers, namely 4‐epoxyguaiacol acetate (EGA), 4‐epoxyguaiacol butanoate (EGB), and 4‐epoxyguaiacol hexanoate (EGH), we used a two‐step procedure starting with 4‐VG acylation followed by double bond epoxidation (Scheme 2), similarly to what was reported for the synthesis of EGA [46]. For the acylation step, different procedures have been reported before [31, 36, 38]. In particular, chemoenzymatic methods have been proposed for the synthesis of 4‐VGA, supporting the use of 4‐VG‐based monomers for the production of sustainable bio‐polymers [72, 73]. To our convenience, we performed acylation reactions of 4‐VG using 1.5 equivalents of the carboxylic acid anhydrides (i.e., acetic anhydride, butyric anhydride, and hexanoic anhydride) without solvent, at 90 °C, and in the presence of 5 mol% of NaOAc. In the case of acetic anhydride, 4‐VGA was obtained in 97% yield after work‐up, and the product was used as obtained in the next step. Otherwise, for 4‐VGB and 4‐VGH, column chromatography was necessary to obtain the products with sufficient purity, resulting in 87% and 73% isolated yield, respectively. The success of acylation reactions was confirmed by Fourier‐transform infrared spectrometry (FT‐IR) analyses, where the broad absorption band of the –OH phenolic group of 4‐VG at about 3500 cm^−1^ disappears, in parallel with the appearance of a sharp absorption band at 1760 cm^−1^ attributed to the carbonyl stretching in the ester group (see Figures S5, S8 and S11). The same was observed by comparison of ^1^H nuclear magnetic resonance (NMR) spectra of 4‐VG and acylated products, where the singlet at 5.65 ppm due to the phenolic –OH group of 4‐VG is no more detected, while signals characteristic for acetate (Figure S2), butanoate (Figure S6), and hexanoate (Figure S9) moieties appear. The epoxidation of isolated guaiacol acylates was performed by a standard reaction with *meta*‐chloroperbenzoic acid (*m*CPBA), and the desired EGA, EGB, and EGH were isolated by column chromatography in 67%, 68%, and 70% yield, respectively. The obtained yields are comparable to those reported for the syntheses of 3‐methoxy and 4‐acetyl substituted styrene oxides [74]. The products' identities were confirmed by combining FT‐IR, high‐resolution mass spectrometry (HR‐MS), and NMR (^1^H, ^13^C, ^1^H–^1^H COSY, and ^1^H–^13^C HSQC experiments) (see Supporting Information). For example, the ^1^H–^1^H COSY NMR spectrum of EGH is shown in Figure 1.

After epoxidation of 4‐VGH to EGH, the signals of protons in positions 8 and 9 are shifted to higher fields because of the epoxide ring formation. In particular, the proton in benzylic position 8 resonates at lower fields (δ = 3.85 ppm) with respect to protons in position 9 (for *cis*‐9‐H and *trans*‐9‐H δ = 3.13 and 2.75 ppm, respectively), and is very close to the –OCH_3_ methoxide singlet at 3.81 ppm (Figure 1a). The ABCD spin system due to the presence of the hexanoate group was maintained, with signals of protons in positions 13 and 14 overlapping in the range 1.45–1.32 ppm; this was also confirmed by ^1^H–^13^C HSQC NMR, where two ^13^C signals at 31.34 and 22.45 ppm correlate with the multiplet centered at 1.39 ppm (see Figure S38). Moreover, the pattern for the 1,2,4‐trisubstituted benzene ring was observed in the aromatic region, with proton 5 exhibiting coupling with proton 6 (Figure 1b).

### 2.2 ROCOP of 4‐Epoxyguaiacol Acetate With Phthalic Anhydride

**FIGURE 2:**
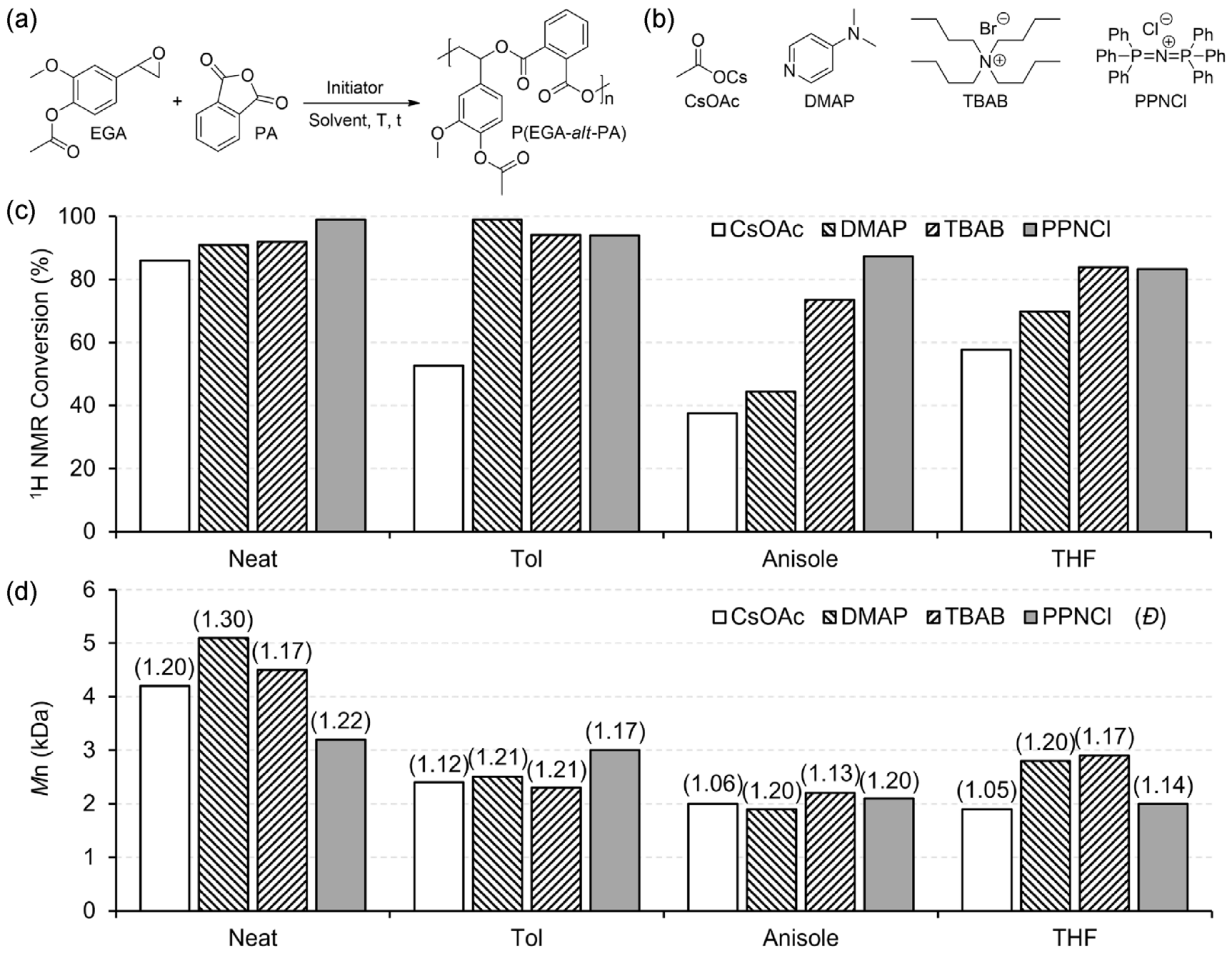
ROCOP of EGA with PA for the synthesis of P(EGA‐*alt*‐PA) (a), and structures of initiators used for the reactions (b). Bar plots of reaction conversions (c) and *M*_*n*_ (d) observed for the ROCOP of EGA with PA with respect to the kind of initiator and solvent used, values in parentheses refer to *Đ* values obtained via SEC. Reaction conditions: [EGA]_0_/[PA]_0_/[I] = 100/100/1, PA = 6.75 · 10^−4^ mol (100 mg), initiator = 6.75 · 10^−6^ mol, [PA]_0_ = 3.4 M, N_2_ atmosphere, reaction time 85 min, T = 110 °C. For neat conditions, [EGA]_0_/[PA]_0_/[I] = 200/100/1. For THF, reaction time 23 h, T = 80 °C. For toluene, reaction time 3.4 h.

To explore the possibility of obtaining PEs from the new guaiacol‐based epoxides, we chose the ROCOP of EGA with phthalic anhydride (PA) as the model reaction to find adequate conditions yielding the desired poly(4‐epoxyguaiacol acetate‐*alt*‐phthalic anhydride) [i.e., P(EGA‐*alt*‐PA), Figure 2a]. The PA is produced from *o*‐xylene oxidation [75], and is commonly used for ROCOP studies thanks to its good reactivity. Because of its synthetic utility [76], several sustainable methods have been proposed for the production of PA from biobased starting materials [77, 78, 79, 80, 81], supporting its use in the sustainable synthesis of semi‐aromatic PEs. With the aim to establish an easy and economic procedure, we selected four different commercially available initiators known to promote the copolymerization of CAs with epoxides, namely cesium acetate (CsOAc), 4‐(dimethylamino)pyridine (DMAP), tetrabutylammonium bromide (TBAB), and bis‐(triphenylphosphine)iminium chloride (PPNCl) (Figure 2b) [82, 83, 84, 85, 86]. Among the possible acetates of alkali metals, we preferred CsOAc because it showed higher activity when tested for the ROCOP of PA with cyclohexene oxide [82].

We performed the screening by testing the initiators under neat conditions or in solution using different solvents [i.e., tetrahydrofuran (THF), toluene, and anisole]. Conversions were determined by ^1^H NMR analyses of aliquots collected at selected reaction times (see Figures S42 and S43), while number‐average molecular weights (*M*_*n*_) and dispersity indexes (*Đ*) were determined by size‐exclusion chromatography (SEC) of polymer samples isolated by precipitation from methanol. Results are listed in Table S1 and selected data summarized in Figure 2. At first, reactions were carried out in the absence of solvent, using 2 equivalents of EGA with respect to PA, with 1.0 mol% of initiator at 110 °C. After 85 min, the reaction mixtures became more viscous, and the reactions were stopped by rapidly cooling the systems to room temperature. Surprisingly, high conversions were observed with all the initiators (conv% ≥ 86%), in particular full conversion was obtained with PPNCl. For comparison, full conversion has been reported for the ROCOP of SO with PPNCl after 12 h under similar reaction conditions [85], while KOAc gave 80.5% conversion after 10 h using 5 equivalents of SO [82]. Polymer identity was confirmed by NMR analyses (see Figures S44−47), and in all cases a perfectly alternated structure was obtained with no evidence of signals due to polyether linkages. Low molecular weights were obtained (3.2 ≤ *M*_*n*_ ≤ 5.1 kDa) with relatively narrow distributions (1.17 ≤ *Đ* ≤ 1.30), as expected for this kind of monomer. Indeed, molecular weights are sensibly lower than the theoretical values calculated for a perfectly controlled process (see entries 1–4, Table S1), but comparable with those reported in the case of poly(SO‐*alt*‐PA) obtained with the same initiators, or other metal‐based catalytic systems [64, 70, 86, 87]. This is likely due to the occurrence of chain‐transfer phenomena and the formation of hydroxy‐terminated species (vide infra).

After these positive results, solvents were tested to avoid the waste of excess epoxide, and a ratio of [EGA]/[PA] = 1/1 was used. When using toluene, PPNCl gave significantly higher conversion (conv% = 66%) with respect to other initiators (compare entries 5–8, Table S1). However, increasing the reaction time to 3.4 h resulted in high monomer conversion for all initiators except for CsOAc (conv% = 53%), likely due to the formation of a tighter ion pair in a nonpolar solvent, such as toluene, as previously proposed by Chen [82]. Somewhat lower *M*_*n*_ values were obtained (2.3 ≤ *M*_*n*_ ≤ 4.1 kDa), maintaining narrow distributions (1.12 ≤ *Đ* ≤ 1.21). Looking for more sustainable alternatives to toluene, anisole was identified according to solvent selection guides [88, 89]. In this case, CsOAc and DMAP exhibited the same activity as in toluene (compare entries 5 and 6 with 13 and 14, Table S1), while TBAB and PPNCl gave higher conversion of 74% and 87%, respectively. However, a bit lower *M*_*n*_ were obtained (compare entries 7 and 8 with 15 and 16, Table S1). At last, THF was tested because it is commonly used for ROCOP reactions. Using THF, the temperature was decreased to 80 °C, resulting in longer reaction times (23 h) to reach moderate to high conversion, with TBAB and PPNCl giving the best results (conv% = 84% and 83%, respectively). In this case, samples with low *M*_*n*_ were obtained too. To evaluate the possible effect of initiator loading, we tested the reaction in toluene solution using a lower concentration of PPNCl, namely 0.25 mol% instead of 1.0 mol% (compare entries 12 and 21, Table S1).

Under these conditions, no significant effect was observed on the molecular weight of P(EGA‐*alt*‐PA) (*M*_*n*_ = 3.1 kDa, and *Đ* = 1.16) but a very good conversion was retained (conv% = 75%). For comparison, the same reaction performed in the absence of initiator gave 25% conversion, but no polymer was obtained by precipitation or detected by SEC analysis (entry 22, Table S1). Finally, we upscaled the reaction of EGA with PA to the gram‐scale under the latter conditions (entry 23, Table S1), obtaining 2.74 g of P(EGA‐*alt*‐PA) (gravimetric yield = 54.4%) with *M*_*n*_ = 3.3 kDa, and *Đ* = 1.14.

### 2.3 Study of Initiator Effect on Polymer Structure by MALDI Analyses

To better understand the structure of P(EGA‐*alt*‐PA), we analyzed the samples obtained with all the initiators, under both neat conditions and in toluene solution, by high‐resolution matrix‐assisted laser desorption/ionization mass spectrometry (HR‐MALDI MS), using 2,5‐dihydroxybenzoic acid (DHBA) as the matrix (Figures S106–S113). In general, all the initiators led to the formation of an alternate microstructure as confirmed by the presence of signals with peak distances equal to the molecular weight of the repeating unit [MW(RU) = 356.09 uma]. In particular, under neat conditions, the sample obtained with CsOAc contains both anhydride‐ and epoxide‐initiated chains with either acetate‐ or hydroxyl‐chain ends, together with cyclic species (Figure S106). A similar situation was observed for TBAB and PPNCl, where a mixture of hydroxyl‐terminated chains and cyclic species was obtained (Figures S108 and S109). Thus, for these systems, the formation of low molecular weight polyester can be attributed to the formation of cyclic species and the occurrence of transesterification phenomena, likely activated by water residues.

On the contrary, when DMAP was used as the initiator, only the presence of DMAP‐initiated linear chains was detected (Figure S107). However, signals compatible with the presence of DMAP‐initiated linear chains bearing alkenyl terminal groups were also detected. Such species may be generated by water elimination from growing polymer chains and explain the slightly larger dispersity compared with other systems (*Đ* = 1.30 vs. *Đ* ≤ 1.22).

**FIGURE 3:**
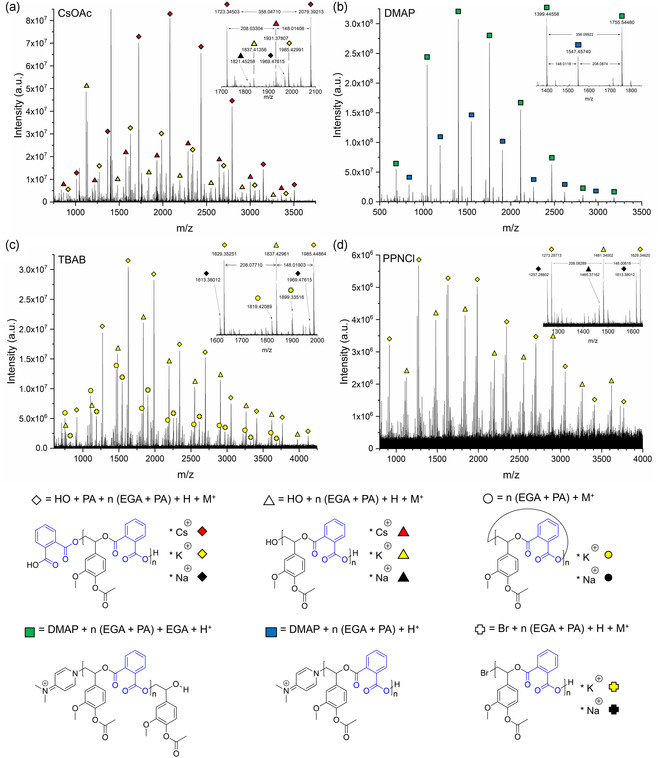
HR‐MALDI spectra of P(EGA‐*alt*‐PA) obtained in toluene with CsOAc (a), DMAP (b), TBAB (c), and PPNCl (d) as in entries 9–12, Table S1. Symbols indicate the species at the bottom. Sodium cations are labeled only in the inserts for clarity.

When toluene was used as the solvent, a simpler situation was observed for almost all the initiators (Figure 3). In the case of CsOAc, only PA‐initiated and EGA‐initiated linear chains bearing hydroxyl‐chain ends were detected, with the former being more abundant than the latter ones (Figure 3a). This is possibly due to the reduced interaction between Cs^+^ cations and EGA molecules in toluene compared with neat conditions, leading to reduced activation of the epoxide ring. In the case of DMAP, the exclusive presence of DMAP‐initiated growing chains was observed, and alkenyl‐terminated species were not detected (Figure 3b). In particular, EGA‐terminated species are more abundant than PA‐terminated ones, suggesting the carboxylate chain‐ends obtained by PA insertion are more reactive than the alcoholate species obtained by EGA insertion. For TBAB, the presence of several linear and cyclic species was observed again (Figure 3c). In detail, apart from linear chains bearing hydroxyl‐chain ends, the formation of Br‐initiated chains was observed, confirming the participation of TBAB in the initiation process. Finally, for PPNCl, the sample is mainly composed of PA‐initiated and EGA‐initiated linear chains bearing hydroxyl terminal groups (Figure 3d). However, a deeper analysis revealed the presence of some Cl‐initiated chains and cyclic species (Figure S114), but in lower amounts if compared with TBAB.

### 2.4 ROCOP of Guaiacol‐Based Epoxides With Cyclic Anhydrides

**SCHEME 3:**
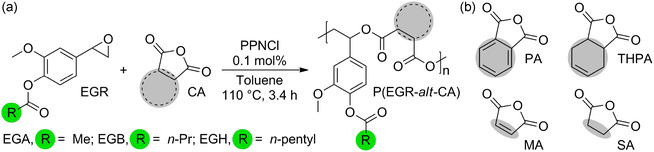
Ring‐opening copolymerization reaction of 4‐epoxyguaiacol acylates (EGR) with cyclic andhyrides (CA) for the synthesis of the corresponding polyesters P(EGR‐alt‐CA) (a). Structures of selected CAs (b).

At this point, we decided to perform the ROCOP of EGA, EGB, and EGH, with PA and other CAs, in order to obtain a set of biobased polyester polyols with different structures (Scheme 3a). We selected monomers whose production from renewables has been demonstrated, i.e., tetrahydrophthalic anhydride (THPA) [90, 91], maleic anhydride (MA) [92, 93], and succinic anhydride (SA) (Scheme 3b) [94]. Based on the results obtained from the previously discussed screening, and the information obtained by MALDI analyses, we decided to use PPNCl as the initiator because it was successful under all tested conditions and produces a large fraction of polyols. Reactions were conducted in toluene solution at 110 °C for 3.4 h, and the products were characterized combining NMR, SEC and MALDI analyses (see Supporting Information).

When PA was used as the cyclic anhydride, a decrease in conversion was observed from 94% to 77% by increasing the acyl chain length from acetate to hexanoate (entries 1–3, Table 1). However, this is likely due to a dilution effect and/or to lower polarity of the reaction medium rather than the steric hindrance of the ester group in position 4 affecting the epoxide ring reactivity. Indeed, similar *M*_*n*_ and *Đ* values were obtained in all cases. To our delight, MALDI analyses confirmed the formation of polyols with alternate microstructure also for P(EGB‐*alt*‐PA) and P(EGH‐*alt*‐PA) (Figures S115, S116).

**TABLE 1:** Ring‐opening copolymerization of guaiacol‐based epoxides with CAs.^a^

Entry	Epoxide	Anhydride	Temperature, °C	Conversion, %^b^	*M*_*n*_ *, * kDa^c^	*Đ* c
1	EGA	PA	110	94	4.1	1.17
2	EGB	PA	110	86	3.1	1.15
3^d^	EGH	PA	110	77	3.6	1.17
4	EGA	THPA	110	81	2.3	1.10
5	EGB	THPA	110	>99	1.8	1.09
6	EGH	THPA	110	80	2.2	1.11
7	EGA	MA	90	89	7.4	10.2
8	EGB	MA	90	>99	5.3	1.86
9	EGH	MA	90	>99	5.5	1.74
10	EGA	SA	110	68	4.0	1.62
11	EGB	SA	110	87	3.2	1.27
12	EGH	SA	110	84	3.7	1.36

a Reaction conditions: [EGR]_0_/[A]_0_/[PPNCl]_0_ = 100/100/1, A = 6.75 · 10^−4^ mol, [A]_0_ = 3.4 M, toluene, N_2_ atmosphere.

b Determined by ^1^H NMR.

c Determined by SEC in THF calibrated with polystyrene standards.

d EGH = 7.8 · 10^−3^ mol (2.05 g), PA = 7.8 · 10^−3^ mol (1.15 g); PPNCl = 7.76 · 10^−5^ mol (44.6 mg).

Since the presence of a double bond in the PE backbone is attractive for further post‐functionalization reactions [43, 45, 95, 96, 97], PA was replaced with the aliphatic anhydrides THPA and MA. In the case of THPA, EGA shows lower reactivity compared to PA under the same conditions (entries 1 and 4, Table 1), while both EGB (entries 2 and 5, Table 1) and EGH (entries 3 and 6, Table 1) gave higher conversion. Tentatively, this difference was attributed to the different solubility of THPA in toluene that, under the reaction conditions, is affected by the presence of different alkyl chains on the epoxides. Indeed, the comparison of MALDI spectra of THPA‐based polyesters revealed that in P(EGA‐*alt*‐THPA) the EGA‐initiated and EGA‐terminated species prevail over THPA‐initiated ones (Figure S116). On the contrary, in P(EGB‐*alt*‐THPA) and P(EGB‐*alt*‐THPA) samples, polymer chains initiated with THPA are predominant (Figures S117, S118). However, in all cases, the formation of perfectly alternated structures with –OH terminals was confirmed. When MA was tested under the same conditions, at 110 °C, all the reactions produced dark and solid materials, insoluble in various solvents (e.g., CH_2_Cl_2_, THF, CHCl_3_, ethyl acetate and petroleum ether). This is likely due to the high reactivity of the MA double bond leading to a crosslink network [98]. So we repeated the reactions at a lower temperature (T = 90 °C), resulting in the formation of ill‐defined species (entries 7–9, Table 1).

Indeed, high conversions were estimated for all the epoxides, but bimodal MW distributions with larger *Đ* values were observed via SEC (Figures S99−101). Moreover, MA‐based polymer samples were soluble in CDCl_3_, and NMR analyses confirmed the formation of the desired polyesters (see Figures S52−55, S68−71, and S84−87). Unfortunately, no polymer signal was detected by MALDI analyses, likely due to molecular weights out of the m/z range limits of the instrument. At last, SA was employed to obtain samples with saturated carbons in the backbone, expecting an increase in chain flexibility affecting thermal properties (vide infra). The reaction with EGA proceeded with good conversion (entry 10, Table 1), forming P(EGA‐*alt*‐SA) with a bimodal distribution (Figure S102). This is possibly due to the increased mobility of the polymer chains leading to an easier formation of cyclic species as observed by MALDI (Figure S120). Indeed, also EGB and EGH polymerizations proceed in good order (entries 11 and 13, Table 1), but the resulting P(EGB‐*alt*‐SA) and P(EGH‐*alt*‐SA) have monomodal MW distributions with low *Đ* values (Figures S103 and S104). Moreover, the formation of hydroxy‐terminated for P(EGB‐*alt*‐SA) was confirmed, in parallel with a low amount of cyclic species (Figure S121).

### 2.5 Thermal Characterization of Polymers

**FIGURE 4:**
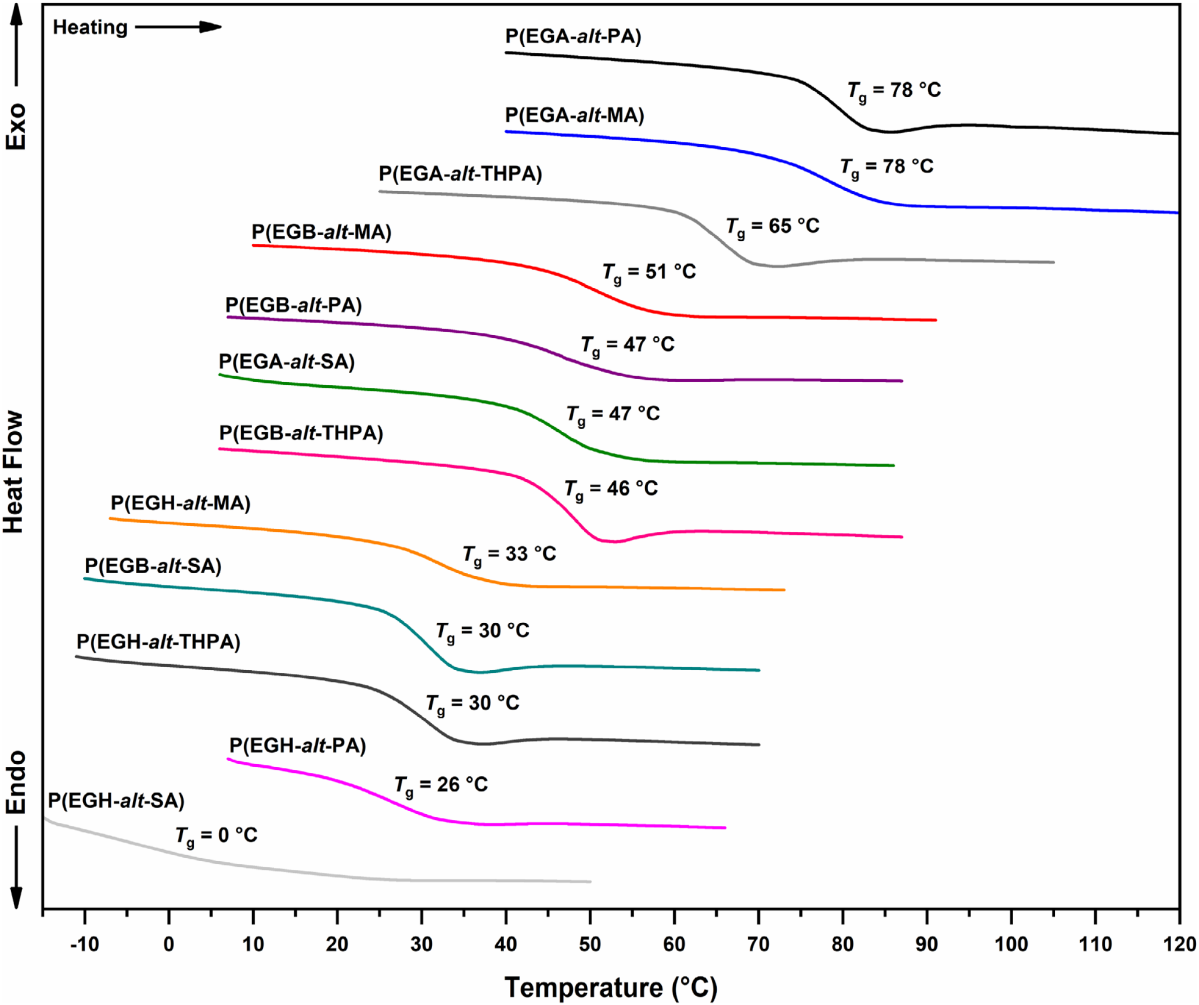
DSC thermograms of new guaiacol‐based polyesters, selected temperature ranges of the second heating cycles showing the corresponding glass‐transition temperatures.

All the new guaiacol‐based polyesters were analyzed by differential scanning calorimetry (DSC) to get information on the structure/thermal properties relationship (Figures S122−134). To facilitate the comparison, we report selected regions of the DSC thermograms showing the observed glass transition temperatures (*T*_g_'s, Figure 4). The highest *T*_g_ value was recorded in the case of the P(EGA‐*alt*‐PA) sample, obtained by copolymerization of PA with EGA (*T*_g_ = 78 °C). This value is higher than that reported for semi‐aromatic poly(SO‐*alt*‐PA) polyester with similar *M*_*n*_, obtained by ROCOP of styrene oxide and PA (4.3 ≤ *M*_*n*_ ≤ 9.0 kDa; 43 ≤ *T*_g_ ≤ 73 °C) [70].

However, higher *T*_g_ was recently reported for high MW poly(SO‐*alt*‐PA) obtained by an alternative route using styrene carbonate (*M*_*n*_ = 178.0 kDa; *T*_g_ = 100 °C) [71]. By varying the cyclic anhydride structure from PA to MA, and maintaining EGA, a very similar *T*_g_ value was observed, likely due to the presence of some cross‐linking in the case of MA, as witnessed by the large *Đ* value (vide supra). In the case of THPA, the *T*_g_ decreases to 65 °C, suggesting that this bicyclic anhydride is sufficient to maintain structural rigidity even in the absence of aromatic groups of PA. Finally, when SA was used, the *T*_g_ drops to 47 °C, as expected for more flexible polymer chains. A similar trend was observed when comparing samples prepared with one epoxide, EGB or EGH, and different anhydrides. On the other hand, the comparison of samples made with PA shows that increasing the length of the acyl moiety by changing the epoxide from EGA to EGB and EGH, the *T*_g_ decreases as expected from 78 to 47 °C and 26 °C, respectively. This is likely due to reduced chain interaction due to the presence of longer and more flexible lateral chains. The same trend was observed for samples based on other CAs, with the highest *T*_g_'s recorded for EGA‐containing PEs. Overall, it is possible to tune the *T*_g_ in the range from 78 °C for P(EGA‐*alt*‐PA), to 0 °C for P(EGH‐*alt*‐SA) by varying the polymer structure simply by tuning the epoxide/cyclic anhydride comonomers.

## 3 Conclusion

Here we described the synthesis and characterization of three styrene oxide‐like epoxy monomers starting from the biobased 4‐vinylguaiacol. These 4‐epoxyguaiacol acylate monomers (EGR), bearing acyl moieties with different lengths (acetate, butanoate, and hexanoate), can be polymerized via ring‐opening copolymerization with CAs by means of a facile and cheap procedure, using simple and commercially available organic initiators (i.e., PPNCl, DMAP, TBAB, and CsOAc). In particular, the effect of the initiator on the initiation process was investigated by MALDI analysis of poly(guaiacol acetate‐*alt*‐phthalic anhydride) samples obtained under different conditions, observing the formation of polyesters with perfectly alternated microstructures, low molecular weights (1.9 ≤ *M*_*n*_ ≤ 5.1 kDa) and narrow distributions (1.06 ≤ *Đ* ≤ 1.30). Only with DMAP, the persistence of DMAP‐initiated polyester chains was observed, while other initiators produce samples containing hydroxy‐terminated chains. In particular, the formation of polyols was more efficient when PPNCl was used as the initiator. Overall, twelve new polyesters were obtained by combining the EGR monomers with phthalic anhydride, tetrahydrophthalic anhydride, maleic anhydride, and succinic anhydride. In particular, the formation of polyols was confirmed in all cases except maleic anhydride, for which the presence of the double bond likely led to the creation of partially cross‐linked samples with higher molecular weights hampering MALDI analyses. Finally, the polymer structure/thermal properties relationship was investigated by measuring glass transition temperatures (*T*_g_'s) with DSC. Overall, the effect of both the rigidity of anhydrides backbones and length of the acyl moiety on the epoxides is evident, allowing us to tune the *T*_g_ in a large temperature range between 0 and 78 °C. In conclusion, with the intention to address the need for sustainable polyesters production methods, we propose the possibility to obtain low molecular weight polyols from biobased monomers derived from 4‐vinylguaiacol. In our opinion, these results are highly relevant to several applications in polymer chemistry, in particular for polyurethane synthesis where the use of polyols is essential.

## Author Contributions

**Erika Zangelmi**: data curation (supporting), investigations (lead), methodology (supporting), validation (equal), writing – review and editing (equal). **Orlando Santoro**: investigations (supporting), visualization (supporting), writing – review and editing (equal). **Raffaele Cucciniello**: investigations (supporting), resources (supporting), writing – review and editing (equal). **Francesco Della Monica**: conceptualization (lead), data curation (lead), funding acquisition (supporting), investigations (supporting), methodology (lead), project administration (equal), resources (supporting), supervision (equal), validation (equal), visualization (lead), writing – original draft (lead), writing – review and editing (equal). **Lorella Izzo**: conceptualization (supporting), funding acquisition (lead), project administration (equal), resources (lead), supervision (equal), writing – review and editing (equal).

## Funding

This publication is part of the project NODES which has received funding from the MUR – M4C2 1.5 of PNRR funded by the European Union ‐ NextGenerationEU (Grant agreement no. ECS00000036 ‐ CUP J83B22000050001). Open Access funding provided by Università degli Studi dell'Insubria within the CRUI‐CARE Agreement.

## Conflict of Interest

The authors declare no conflicts of interest.

## Supporting information

The authors have cited additional references within the Supporting Information [31, 38, 74, 99].

## Data Availability

The data that supports the findings of this study are available in the supplementary material of this article.
