# Supplementary material for: New Biobased Polyester Polyols With Tunable T g Values From 4‐Vinylguaiacol‐Derived Monomers
Source: ChemSusChem. 2026 Jul 23;19(15):e70901. doi: 10.1002/cssc.70901 (PMC13393342; doi:10.1002/cssc.70901)
Supplement: Supplementary file 1 — The authors have cited additional references within the Supporting Information [31, 38, 74, 99]. [file CSSC-19-e70901-s001.pdf]

Supporting Information

New Biobased Polyester Polyols with Tuneable T_g Values from 4-Vinylguaiacol-Derived Monomers

Erika Zangelmi,[a] Orlando Santoro,[a] Raffaele Cucciniello,[b] Francesco Della Monica,*[a] and Lorella Izzo*[a]

[a] Dr. E. Zangelmi, Dr. O. Santoro, Prof. F. Della Monica, Prof. L. Izzo
Department of Biotechnology and Life Science
University of Insubria
Via Jean Henry Dunant, 3, 21100 – Varese, Italy

[b] Prof. R. Cucciniello
Department of Chemistry and Biology "Adolfo Zambelli"
University of Salerno
Via Giovanni Paolo II, 132, 84084 – Fisciano, Italy

*Corresponding authors: f.dellamonica@uninsubria.it, lorella.izzo@uninsubria.it

Table of contents

1. General Considerations 9

2. Reagents and Monomers 9

3. Synthesis of 4-vinyl guaiacol acylates 10

FigureS 1. ^{1}H NMR of commercial 4-vinyl guaiacol (400 MHz, rt, $CDCl_3$). 10

3.1 Synthesis of 4-vinyl guaiacol acetate (4-VGA) 10

FigureS 2. ^{1}H NMR of 4-vinyl guaiacol acetate after work-up (400 MHz, rt, $CDCl_3$). 11

FigureS 3. ^{1}H NMR of 4-vinyl guaiacol acetate after column chromatography (400 MHz, rt, $CDCl_3$). 11

FigureS 4. ATR-FTIR of 4-vinyl guaiacol acetate. 12

FigureS 5. Comparison of the ATR-FTIR spectra of 4-vinyl guaiacol (blue curve) and of 4-vinyl guaiacol acetate (red curve). 12

3.2 Synthesis of 4-vinyl guaiacol butanoate (4-VGB) 13

FigureS 6. ^{1}H NMR of 4-vinyl guaiacol butanoate (400 MHz, rt, $CDCl_3$). Signals due to butanoic acid and butanoic anhydride impurities are marked with *. 13

FigureS 7. ATR-FTIR of 4-vinyl guaiacol butanoate. 14

FigureS 8. Comparison of the ATR-FTIR spectra of 4-vinyl guaiacol (blue curve) and of 4-vinyl guaiacol butanoate (red curve). 14

3.3 Synthesis of 4-vinyl guaiacol hexanoate (4-VGH) 15

FigureS 9. ^{1}H NMR of 4-vinyl guaiacol hexanoate (400 MHz, rt, $CDCl_3$). 15

FigureS 10. ATR-FTIR of 4-vinyl guaiacol hexanoate. 16

FigureS 11. Comparison of the ATR-FTIR spectra of 4-vinyl guaiacol (blue curve) and of 4-vinyl guaiacol hexanoate (red curve). 16

4. Synthesis of 4-epoxy guaiacol acylates 17

4.1 Synthesis of 4-epoxy guaiacol acetate (EGA) 17

FigureS 12. High Resolution ESI MS measured (top) and calculated (bottom) spectra of 4-epoxy guaiacol acetate in MeOH, region from 203 to 219 m/z. $[C_{11}H_{13}O_4]^+$ calcd. = 209.08084 uma; measured = 209.08111 uma. 17

FigureS 13. ^{1}H NMR of 4-epoxy guaiacol acetate (600 MHz, rt, $CDCl_3$). 18

FigureS 14. ^{13}C NMR of 4-epoxy guaiacol acetate (151 MHz, rt, $CDCl_3$). 18

FigureS 15. ^{1}H-^{1}H COSY NMR of 4-epoxy guaiacol acetate (600 MHz, rt, $CDCl_3$). 19

FigureS 16. ^{1}H-^{1}H COSY NMR of 4-epoxy guaiacol acetate from 4.0 to 2.0 ppm (600 MHz, rt, $CDCl_3$). 19

FigureS 17. ^{1}H-^{1}H COSY NMR of 4-epoxy guaiacol acetate from 7.05 to 6.75 ppm (600 MHz, rt, $CDCl_3$). 20

FigureS 18. ^{1}H-^{13}C HSQC NMR of 4-epoxy guaiacol acetate (600 MHz, rt, $CDCl_3$). 20

FigureS 19. ATR-FTIR of 4-epoxy guaiacol acetate. 21

FigureS 20. Comparison of the ATR-FTIR spectra of 4-vinyl guaiacol acetate (blue curve) and of 4-epoxy guaiacol acetate (red curve). 21

FigureS 21. Comparison of the ATR-FTIR spectra of 4-vinyl guaiacol acetate (blue curve) and of 4-epoxy guaiacol acetate (red curve). Region from 2000 to 650 cm^{-1}. 22

4.2 Synthesis of 4-epoxy guaiacol butanoate (VGB) 23

FigureS 22. High Resolution ESI MS measured (top) and calculated (bottom) spectra of 4-epoxy guaiacol butanoate in MeOH, region from 233 to 242 m/z. $[C_{11}H_{13}O_4]^+$ calcd. = 237.11214 uma; measured = 237.11226 uma. 23

FigureS 23. ^{1}H NMR of 4-epoxy guaiacol butanoate (400 MHz, rt, $CDCl_3$). 24

FigureS 24. ^{13}C NMR of 4-epoxy guaiacol butanoate (151 MHz, rt, $CDCl_3$). 24

FigureS 25. ^{1}H-^{1}H COSY NMR of 4-epoxy guaiacol butanoate (600 MHz, rt, $CDCl_3$). 25

FigureS 26. ^{1}H-^{1}H COSY NMR of 4-epoxy guaiacol butanoate from 4.5 to 0.5 ppm (600 MHz, rt, $CDCl_3$). 25

FigureS 27. ^{1}H-^{1}H COSY NMR of 4-epoxy guaiacol butanoate from 7.1 to 6.7 ppm (600 MHz, rt, $CDCl_3$). 26

FigureS 28. ^{1}H-^{13}C HSQC NMR of 4-epoxy guaiacol butanoate (600 MHz, rt, $CDCl_3$). 26

FigureS 29. ATR-FTIR of 4-epoxy guaiacol butanoate. 27

FigureS 30. Comparison of the ATR-FTIR spectra of 4-vinyl guaiacol butanoate (blue curve) and of 4-epoxy guaiacol butanoate (red curve). 27

FigureS 31. Comparison of the ATR-FTIR spectra of 4-vinyl guaiacol butanoate (blue curve) and of 4-epoxy guaiacol butanoate (red curve). Region from 2000 to 650 cm^{-1}. 28

4.3 Synthesis of 4-epoxy guaiacol hexanoate (4-VGH) 29

FigureS 32. High Resolution ESI MS measured (top) and calculated (bottom) spectra of 4-epoxy guaiacol hexanoate in MeOH, region from 250 to 280 m/z. $[C_{11}H_{13}O_4]^+$ calcd. = 265.14344 uma; measured = 265.14391 uma. 29

FigureS 33. ^{1}H NMR of 4-epoxy guaiacol hexanoate (400 MHz, rt, $CDCl_3$). 30

FigureS 34. ^{13}C NMR of 4-epoxy guaiacol hexanoate (151 MHz, rt, $CDCl_3$). 30

FigureS 35. ^{1}H-^{1}H COSY NMR of 4-epoxy guaiacol hexanoate (600 MHz, rt, $CDCl_3$). 31

FigureS 36. ^{1}H-^{1}H COSY NMR of 4-epoxy guaiacol hexanoate from 4.5 to 0.5 ppm (600 MHz, rt, $CDCl_3$). 31

FigureS 37. ^{1}H-^{1}H COSY NMR of 4-epoxy guaiacol hexanoate from 7.1 to 6.7 ppm (600 MHz, rt, $CDCl_3$). 32

FigureS 38. ^{1}H-^{13}C HSQC NMR of 4-epoxy guaiacol hexanoate (600 MHz, rt, $CDCl_3$). 32

FigureS 39. ATR-FTIR of 4-epoxy guaiacol hexanoate. 33

FigureS 40. Comparison of the ATR-FTIR spectra of 4-vinyl guaiacol hexanoate (blue curve) and of 4-epoxy guaiacol hexanoate (red curve). 33

FigureS 41. Comparison of the ATR-FTIR spectra of 4-vinyl guaiacol hexanoate (blue curve) and of 4-epoxy guaiacol hexanoate (red curve). Region from 2000 to 650 cm^{-1}. 34

5. Ring-Opening Copolymerization Procedures 35

5.1 Catalyst screening for EGA/PA ROCOP without solvent 35

FigureS 42. ^{1}H NMR spectra of an aliquot of the reaction mixture for the formation of P(EGA-*alt*-PA) for conversion determination (entry 1, Table S1) (400 MHz, rt, $CDCl_3$,) 35

5.2 Catalyst screening for EGA/PA ROCOP with different solvents 36

FigureS 43. ^{1}H NMR spectra of an aliquot of the reaction mixture for the formation of P(EGA-*alt*-PA) for conversion determination (entry 12, Table S1) (400 MHz, rt, $CDCl_3$,) 36

TableS 1. Screening of catalysts and solvents for the ROCOP of EGA with PA.[a)] 37

5.3 ROCOP of 4-Epoxyguaiacol Acylate with Cyclic Anhydrides 38

6. NMR Characterization of Polymers 39

FigureS 44. ^{1}H NMR of P(EGA-*alt*-PA) (400 MHz, rt, $CDCl_3$). 39

FigureS 45. ^{13}C NMR of P(EGA-*alt*-PA) (600 MHz, rt, $CDCl_3$). 39

FigureS 46. ^{1}H-^{1}H COSY NMR of P(EGA-*alt*-PA) (600 MHz, rt, $CDCl_3$). 40

FigureS 47. ^{1}H-^{13}C HSQC NMR of P(EGA-*alt*-PA) (600 MHz, rt, $CDCl_3$). 40

FigureS 48. ^{1}H NMR of P(EGA-*alt*-THPA) (600 MHz, rt, $CDCl_3$). 41

FigureS 49. ^{13}C NMR of P(EGA-*alt*-THPA) (600 MHz, rt, $CDCl_3$). 41

FigureS 50. ^{1}H-^{1}H COSY NMR of P(EGA-*alt*-THPA) (600 MHz, rt, $CDCl_3$) 42

FigureS 51. ^{1}H-^{13}C HSQC NMR of P(EGA-*alt*-THPA) (600 MHz, rt, $CDCl_3$). 42

FigureS 52. ^{1}H NMR of P(EGA-*alt*-MA) (600 MHz, rt, $CDCl_3$). 43

FigureS 53. ^{13}C NMR of P(EGA-*alt*-MA) (600 MHz, rt, $CDCl_3$). 43

FigureS 54. ^{1}H-^{1}H COSY NMR of P(EGA-*alt*-MA) (600 MHz, rt, $CDCl_3$). 44

FigureS 55. ^{1}H-^{13}C HSQC NMR of P(EGA-*alt*-MA) (600 MHz, rt, $CDCl_3$). 44

FigureS 56. ^{1}H NMR of P(EGA-*alt*-SA) (600 MHz, rt, $CDCl_3$). 45

FigureS 57. ^{13}C NMR of P(EGA-*alt*-SA) (600 MHz, rt, $CDCl_3$). 45

FigureS 58. ^{1}H-^{1}H COSY NMR of P(EGA-*alt*-SA) (600 MHz, rt, $CDCl_3$). 46

FigureS 59. ^{1}H-^{13}C HSQC NMR of P(EGA-*alt*-SA) (600 MHz, rt, $CDCl_3$). 46

FigureS 60. ^{1}H NMR of P(EGB-*alt*-PA) (600 MHz, rt, $CDCl_3$). 47

FigureS 61. ^{13}C NMR of P(EGB-*alt*-PA) (600 MHz, rt, $CDCl_3$). 47

FigureS 62. ^{1}H-^{1}H COSY NMR of P(EGB-*alt*-PA) (600 MHz, rt, $CDCl_3$). 48

FigureS 63. ^{1}H-^{13}C HSQC NMR of P(EGB-*alt*-PA) (600 MHz, rt, $CDCl_3$). 48

FigureS 64. ^{1}H NMR of P(EGB-*alt*-THPA) (600 MHz, rt, $CDCl_3$). 49
FigureS 65. ^{13}C NMR of P(EGB-*alt*-THPA) (600 MHz, rt, $CDCl_3$). 49
FigureS 66. ^{1}H-^{1}H COSY NMR of P(EGB-*alt*-THPA) (600 MHz, rt, $CDCl_3$). 50
FigureS 67. ^{1}H-^{13}C HSQC NMR of P(EGB-*alt*-THPA) (600 MHz, rt, $CDCl_3$). 50
FigureS 68. ^{1}H NMR of P(EGB-*alt*-MA) (600 MHz, rt, $CDCl_3$). 51
FigureS 69. ^{13}C NMR of P(EGB-*alt*-MA) (600 MHz, rt, $CDCl_3$). 51
FigureS 70. ^{1}H-^{1}H COSY NMR of P(EGB-*alt*-MA) (600 MHz, rt, $CDCl_3$). 52
FigureS 71. ^{1}H-^{13}C HSQC NMR of P(EGB-*alt*-MA) (600 MHz, rt, $CDCl_3$). 52
FigureS 72. ^{1}H NMR of P(EGB-*alt*-SA) (600 MHz, rt, $CDCl_3$). 53
FigureS 73. ^{13}C NMR of P(EGB-*alt*-SA) (600 MHz, rt, $CDCl_3$). 53
FigureS 74. ^{1}H-^{1}H COSY NMR of P(EGB-*alt*-SA) (600 MHz, rt, $CDCl_3$). 54
FigureS 75. ^{1}H-^{13}C HSQC NMR of P(EGB-*alt*-SA) (600 MHz, rt, $CDCl_3$). 54
FigureS 76. ^{1}H NMR of P(EGH-*alt*-PA) (600 MHz, rt, $CDCl_3$). 55
FigureS 77. ^{13}C NMR of P(EGH-*alt*-PA) (600 MHz, rt, $CDCl_3$). 55
FigureS 78. ^{1}H-^{1}H COSY NMR of P(EGH-*alt*-PA) (600 MHz, rt, $CDCl_3$). 56
FigureS 79. ^{1}H-^{13}C HSQC NMR of P(EGH-*alt*-PA) (600 MHz, rt, $CDCl_3$). 56
FigureS 80. ^{1}H NMR of P(EGH-*alt*-THPA) (600 MHz, rt, $CDCl_3$). 57
FigureS 81. ^{13}C NMR of P(EGH-*alt*-THPA) (600 MHz, rt, $CDCl_3$). 57
FigureS 82. ^{1}H-^{1}H COSY NMR of P(EGH-*alt*-THPA) (600 MHz, rt, $CDCl_3$). 58
FigureS 83. ^{1}H-^{13}C HSQC NMR of P(EGH-*alt*-PA) (600 MHz, rt, $CDCl_3$). 58
FigureS 84. ^{1}H NMR of P(EGH-*alt*-MA) (600 MHz, rt, $CDCl_3$). 59
FigureS 85. ^{13}C NMR of P(EGH-*alt*-MA) (600 MHz, rt, $CDCl_3$). 59
FigureS 86. ^{1}H-^{1}H COSY NMR of P(EGH-*alt*-MA) (600 MHz, rt, $CDCl_3$). 60
FigureS 87. ^{1}H-^{13}C HSQC NMR of P(EGH-*alt*-MA) (600 MHz, rt, $CDCl_3$). 60
FigureS 88. ^{1}H NMR of P(EGH-*alt*-SA) (600 MHz, rt, $CDCl_3$). 61
FigureS 89. ^{13}C NMR of P(EGH-*alt*-SA) (600 MHz, rt, $CDCl_3$). 61
FigureS 90. ^{1}H-^{1}H COSY NMR of P(EGH-*alt*-SA) (600 MHz, rt, $CDCl_3$). 62
FigureS 91. ^{1}H-^{13}C HSQC NMR of P(EGH-*alt*-SA) (600 MHz, rt, $CDCl_3$). 62
7. SEC Analyses of Polymers 63
FigureS 92. SEC trace of P(EGA-*alt*-PA) prepared as in entry 23, Table S1. 63
FigureS 93. SEC trace of P(EGA-*alt*-PA) prepared as in entry 1, Table 1. 63
FigureS 94. SEC trace of P(EGB-*alt*-PA) prepared as in entry 2, Table 1. 64

FigureS 95. SEC trace of P(EGH-*alt*-PA) prepared as in entry 3, Table 1. 64

FigureS 96. SEC trace of P(EGA-*alt*-THPA) prepared as in entry 4, Table 1. 65

FigureS 97. SEC trace of P(EGB-*alt*-THPA) prepared as in entry 5, Table 1. 65

FigureS 98. SEC trace of P(EGH-*alt*-THPA) prepared as in entry 6, Table 1. 66

FigureS 99. SEC trace of P(EGA-*alt*-MA) prepared as in entry 7, Table 1. 66

FigureS 100. SEC trace of P(EGB-*alt*-MA) prepared as in entry 8, Table 1. 67

FigureS 101. SEC trace of P(EGH-*alt*-MA) prepared as in entry 9, Table 1. 67

FigureS 102. SEC trace of P(EGA-*alt*-SA) prepared as in entry 10, Table 1. 68

FigureS 103. SEC trace of P(EGB-*alt*-SA) prepared as in entry 11, Table 1. 68

FigureS 104. SEC trace of P(EGH-*alt*-SA) prepared as in entry 12, Table 1. 69

FigureS 105. SEC trace of P(EGH-*alt*-PA) prepared as in entry 10, Table 1. 69

8. HR-MALDI FT-ICR MS analyses of polymers 70

FigureS 106. High Resolution MALDI spectrum of P(EGA-*alt*-PA) as obtained in entry 1, Table S1. a) Region from 600 to 3500 m/z. b) Region from 1575 to 2075 m/z. Symbols ◇, △, ○, □, and ✣ indicate the species at the bottom. Sodium cations are labeled only in insert (b) for clarity. 70

FigureS 107. High Resolution MALDI spectrum of P(EGA-*alt*-PA) as obtained in entry 2, Table S1. a) Region from 500 to 5000 m/z. b) Region from 1370 to 1410 m/z. c) Region from 2700 to 3300 m/z. Symbols ◇, △, and ○, indicate the species at the bottom. 71

FigureS 108. High Resolution MALDI spectrum of P(EGA-*alt*-PA) as obtained in entry 3, Table S1. a) Region from 600 to 4500 m/z. b) Region from 1450 to 1850 m/z. Symbols ◇, △, □ and ○ indicate the species at the bottom. Sodium cations are labeled only in insert (b) for clarity. 72

FigureS 109. High Resolution MALDI spectrum of P(EGA-*alt*-PA) as obtained in entry 4, Table S1. a) Region from 1425 to 1875 m/z. Symbols ◇, △, ○, □, and ✣ indicate the species at the bottom. Sodium cations are labeled only in insert (b) for clarity. 73

FigureS 110. High Resolution MALDI spectrum of P(EGA-*alt*-PA) as obtained in entry 9, Table S1. a) Region from 350 to 3750 m/z. b) Region from 1700 to 2100 m/z. Symbols ◇ and △ indicate the species at the bottom. Sodium cations are labeled only in insert (b) for clarity. 74

FigureS 111. High Resolution MALDI spectrum of P(EGA-*alt*-PA) as obtained in entry 10, Table S1. a) Region from 500 to 3500 m/z. b) Region from 1350 to 1850 m/z. Symbols ◇, and △ indicate the species at the bottom. 75

FigureS 112. High Resolution MALDI spectrum of P(EGA-*alt*-PA) as obtained in entry 11, Table S1. a) Region from 600 to 4250 m/z. b) region from 1600 to 2000 m/z. Symbols ◇, △, ○, and ✣ indicate the species at the bottom. Sodium cations are labeled only in insert (b) for clarity. 76

FigureS 113. High Resolution MALDI spectrum of P(EGA-*alt*-PA) as obtained in entry 12, Table S1. a) Region from 1240 to 1640 m/z. Symbols ◇, and △ indicate the species at the bottom. Sodium cations are labeled only in insert (b) for clarity. 77

FigureS 114. Comparison between High Resolution MALDI spectrum of P(EGA-*alt*-PA) as obtained in entry 12, Table S1 in the region from 1440 to 1510 m/z (a), and calculated spectrum for the species at the bottom indicated with symbols ○, △, and □ (b). Measured and calculated isotopic peaks values are reported in the captions. 78

FigureS 115. High Resolution MALDI spectrum of P(EGB-*alt*-PA) as obtained in entry 2, Table 1. a) Region from 600 to 4500 m/z. b) Region from 2100 to 2550 m/z. Symbols ◇, △, and □ indicate the species at the bottom. Sodium cations are labeled only in insert (b) for clarity. 79

FigureS 116. High Resolution MALDI spectrum of P(EGH-*alt*-PA) as obtained in entry 3, Table 1. a) Region from 650 to 3500 m/z. b) Region from 1400 to 1900 m/z. Symbols ◇, △, □, and ○ indicate the species at the bottom. 80

FigureS 117. High Resolution MALDI spectrum of P(EGA-*alt*-THPA) as obtained in entry 4, Table 1. a) Region from 800 to 3500 m/z. b) Region from 1825 to 2050 m/z. Symbols ◇, △, and □ indicate the species at the bottom. Sodium cations are labeled only in insert (b) for clarity. 81

FigureS 118. High Resolution MALDI spectrum of P(EGB-*alt*-THPA) as obtained in entry 5, Table 1. a) Region from 600 to 3000 m/z. b) Region from 1325 to 1775 m/z. Symbols ◇, and △ indicate the species at the bottom. Sodium cations are labeled only in insert (b) for clarity. 82

FigureS 119. High Resolution MALDI spectrum of P(EGH-*alt*-THPA) as obtained in entry 6, Table 1. a) Region from 600 to 3000 m/z. b) Region from 1425 to 1900 m/z. Symbols ◇, and △ indicate the species at the bottom. Sodium cations are labeled only in insert (b) for clarity. 83

FigureS 120. High Resolution MALDI spectrum of P(EGA-*alt*-SA) as obtained in entry 10, Table 1. a) Region from 600 to 3000 m/z. b) Region from 1350 to 1750 m/z. Symbols ◇, △, and ○ indicate the species at the bottom. 84

FigureS 121. High Resolution MALDI spectrum of P(EGB-*alt*-SA) as obtained in entry 11, Table 1. a) Region from 600 to 3500 m/z. b) Region from 1450 to 1850 m/z. Symbols ◇, △, and ○ indicate the species at the bottom. Sodium cations are labeled only in insert (b) for clarity. 85

9. DSC analyses of polymers 86

FigureS 122. DSC thermogram of P(EGA-*alt*-PA) prepared as in entry 23, Table S1. 86

FigureS 123. DSC thermogram of P(EGA-*alt*-PA) prepared as in entry 1, Table 1 86

FigureS 124. DSC thermogram of P(EGB-*alt*-PA) prepared as in entry 2, Table 1 87

FigureS 125. DSC thermogram of P(EGH-*alt*-PA) prepared as in entry 3, Table 1. 87

FigureS 126. DSC thermogram of P(EGA-*alt*-THPA) prepared as in entry 4, Table 1. 88

FigureS 127. DSC thermogram of P(EGB-*alt*-THPA) prepared as in entry 5, Table 1. 88

FigureS 128. DSC thermogram of P(EGH-*alt*-THPA) prepared as in entry 6, Table 1. 89

FigureS 129. DSC thermogram of P(EGA-*alt*-MA) prepared as in entry 7, Table Y. 89

FigureS 130. DSC thermogram of P(EGB-*alt*-MA) prepared as in entry 8, Table 1. 90

FigureS 131. DSC thermogram of P(EGH-*alt*-MA) prepared as in entry 9, Table 1. 90

FigureS 132. DSC thermogram of P(EGA-*alt*-SA) prepared as in entry 10, Table 1. 91

FigureS 133. DSC thermogram of P(EGB-*alt*-SA) prepared as in entry 11, Table 1. 91

FigureS 134. DSC thermogram of P(EGH-*alt*-SA) prepared as in entry 12, Table 1. 92

References 93

1. General Considerations

All water-sensitive operations were carried out under nitrogen atmosphere using standard vacuum line and Schlenk techniques. Nuclear magnetic resonance (NMR) spectra were acquired either on a Bruker Avance 400 (operating at 400 MHz for ^{1}H and 101 MHz for ^{13}C) or a Bruker Avance III HD 600 (operating at 600 MHz for ^{1}H and 151 MHz for ^{13}C) spectrometers, at ambient temperature in deuterated solvents. Chemical shifts values are given as δ in [ppm], with ^{1}H NMR spectra referenced to the residual solvent peak at δ 7.26 ppm for $CDCl_3$, and ^{13}C NMR spectra referenced to the residual solvent peak at δ 77.16 ppm for $CDCl_3$. Values of coupling constants, *J*, are given in Hz. Multiplicities are indicated as: *s* (singlet), *d* (doublet), *t* (triplet), *q* (quartet), *quint* (quintet), *sext* (sextet) and *m* (multiplet). NMR peaks assignments were confirmed using 2D ^{1}H correlated spectroscopy (COSY), and 2D $^{1}H-^{13}C$ heteronuclear single quantum coherence (HSQC) where necessary. Attenuated Total Reflectance - Fourier Transform Infrared (ATR-FTIR) spectra were recorded on a Cary 630 FTIR spectrometer (Agilent Technologies) at room temperature with 16 scans and a resolution of 4 cm^{-1}. Differential scanning calorimetry (DSC) analyses for determination of the glass transition temperatures (T_g) were measured under a N_2 atmosphere using a Discovery DSC25 instrument (Waters TA Instruments) equipped with a two-stage refrigeration system (Refrigerated Cooling System 90) operating in the temperature range from -90 to 550°C. Samples were weighed into 20 *μ*L aluminum crucibles and subjected to two heating cycles at a heating rate of 10 °C/min. Size exclusion chromatography (SEC) measurements were performed using a Jasco LC-4000 HPLC apparatus equipped with a Jasco RI-4030 refractive index (RI) detector, using a Shodex KF-804L column (8 x 300 mm; 7 μm particle size), operating at a flow rate of 1 mL/min, in THF at 30 °C. Samples were analyzed at a concentration of 1.5 $mg \cdot mL^{-1}$ after filtration through a PTFE 0.45 μm pore-size membrane (ISOLAB).The values of M_n, M_w, and *Đ* were derived from the RI signal by a calibration curve based on polystyrene standards (EasiVial PS-M, 2 mL, Agilent) for the polymer analyses.

High resolution Electrospray Ionization Fourier Transform Ion Cyclotron Resonance Mass Spectrometry (ESI FT-ICR MS) measurements of monomers were performed on a Bruker Solaris XR instrument using MeOH as the solvent. HR-Matrix-Assisted Laser Desorption/Ionization FT-ICR MS (HR-MALDI FT-ICR MS) measurements of polymers were performed on a Bruker Solarix XR instrument using the following conditions: sample (4 μL, 1 mg/mL in CH_2Cl_2), 2,5-DHBA as a matrix (45 μL, 40 mM in CH_2Cl_2).

2. Reagents and Monomers

All reagents, including 4-vinylguaiacol, were purchased from commercial suppliers (Merck and TCI) and used as received if not differently specified. Solvents were purchased from Sigma-Aldrich, and Carlo Erba Reagents (HPLC grade) and dried using activated 4 Å molecular sieves. Phthalic anhydride (PA) was recrystallized from hot chloroform and dried under vacuum for 48 h over P_2O_5 before use. *Cis*-1,2,3,6-tetrahydrophthalic anhydride (THPA) and maleic anhydride (MA) were recrystallized from dichloromethane at -20 °C, and dried under vacuum for 48 h over P_2O_5 before use. Succinic anhydride (SA) was recrystallized from dichloromethane at − 20 °C, washed twice with diethyl ether, and dried under vacuum for 48 h over P_2O_5 before use. The initiators bis(triphenylphosphine)iminium chloride (PPNCl), tetra-*n*-butylammonium bromide (TBAB), cesium acetate (CsOAc), and 4-(dimethylamino)pyridine (DMAP) are commercially available, were stored in a desiccator over P_2O_5 and used as received. The monomers 4-epoxyguaiacol acetate (EGA), 4-epoxyguaiacol butyrate (EGB), and 4-epoxyguaiacol hexanoate (EGH) were dried under vacuum for 48 h over P_2O_5 and stored under nitrogen atmosphere prior to use in copolymerization reactions.

3. Synthesis of 4-vinyl guaiacol acylates

The 4-viniyl guaiacol acylates were prepared by reaction with carboxylic acid anhydrides, following a procedure similar to that previously reported.[38, 99] Product identities were confirmed by ^{1}H NMR analyses, that were in agreement with previous reports.[31, 38, 99] The ^{1}H NMR spectra of 4-vinyl-guaiacol (4-VG) is reported for comparison (Figure S1).

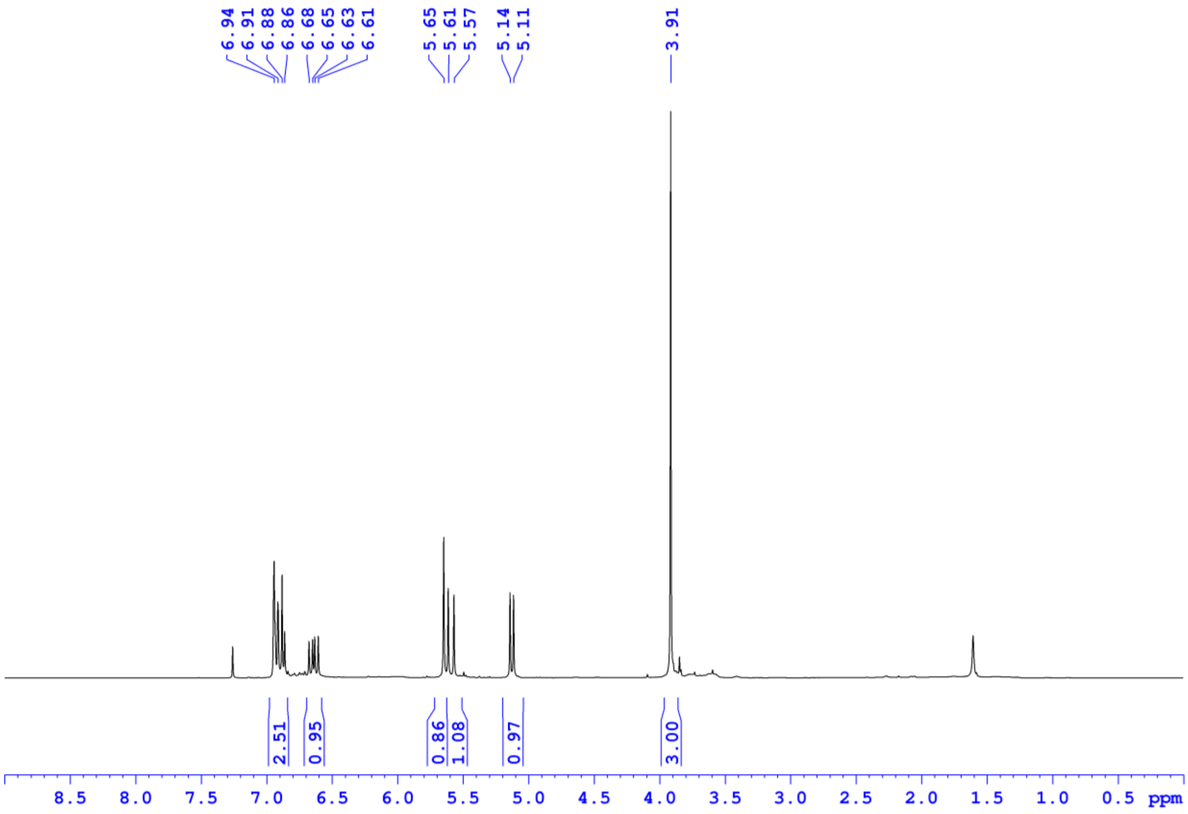

FigureS 1. ^{1}H NMR of commercial 4-vinyl guaiacol (400 MHz, rt, $CDCl_3$).

3.1 Synthesis of 4-vinyl guaiacol acetate (4-VGA)

The 4-vinylguaiacol (5.00 g, 33.3 mmol) was weighted in a 25 mL round-bottomed flask equipped with a condenser, dissolved in acetic anhydride (4.7 mL, 5.08 g, 49.7 mmol), and sodium acetate (137 mg, 1.67 mmol) was added. The reaction mixture was stirred at 90 °C for 1 hour, then allowed to cool down to room temperature and diluted with EtOAc (50 mL). The organic phase was extracted sequentially with 1 M HCl (3 × 50 mL), deionised water (2 × 5 mL), and brine (2 × 50 mL). The organic phase was dried over anhydrous sodium sulfate, and the solvent removed by rotary evaporation at reduced pressure yielding 4-VGA (yield = 97.0%, 6.21 g, 32.3 mmol). The product was used, for the following reactions, without further purifications. However, some impurities were detected by ^{1}H NMR (Figure S2) and the product was further purified by column chromatography on silica gel using a solvent mixture of petroleum ether/EtOAc = 9/1, yielding pure 4-VGA (yield = 87.0%, 5.57 g, 29.0 mmol) (see Figure S3).

NMR Spectroscopic data of 4-VGA

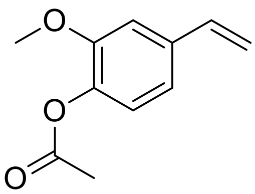

^{1}H NMR (400 MHz, $CDCl_3$, rt): δ 7.01-6.99 (*m*, 3H, Ar-H); 6.68 (*dd*, 1H, *J* = 17.6 Hz, *J* = 10.8 Hz, -C*H*=CH$_2$); 5.70 (*d*, 1H, *J* = 17.6 Hz, *trans*-CH=C*H*$_2$); 5.25 (*d*, 1H, *J* = 10.8 Hz, *cis*-CH=C*H*$_2$); 3.85 (*s*, 1H, -OC*H*$_3$); 2.31 (*s*, 1H, -OCOC*H*$_3$).

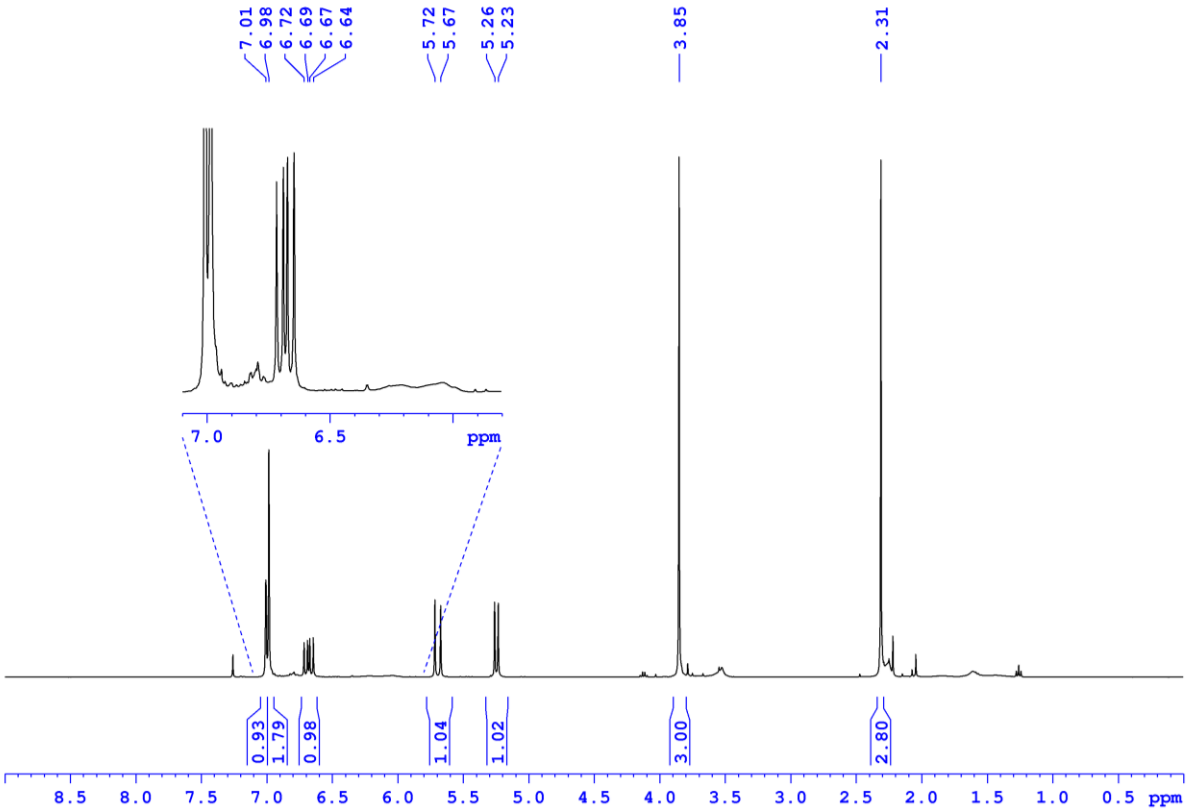

FigureS 2. ^{1}H NMR of 4-vinyl guaiacol acetate after work-up (400 MHz, rt, $CDCl_3$).

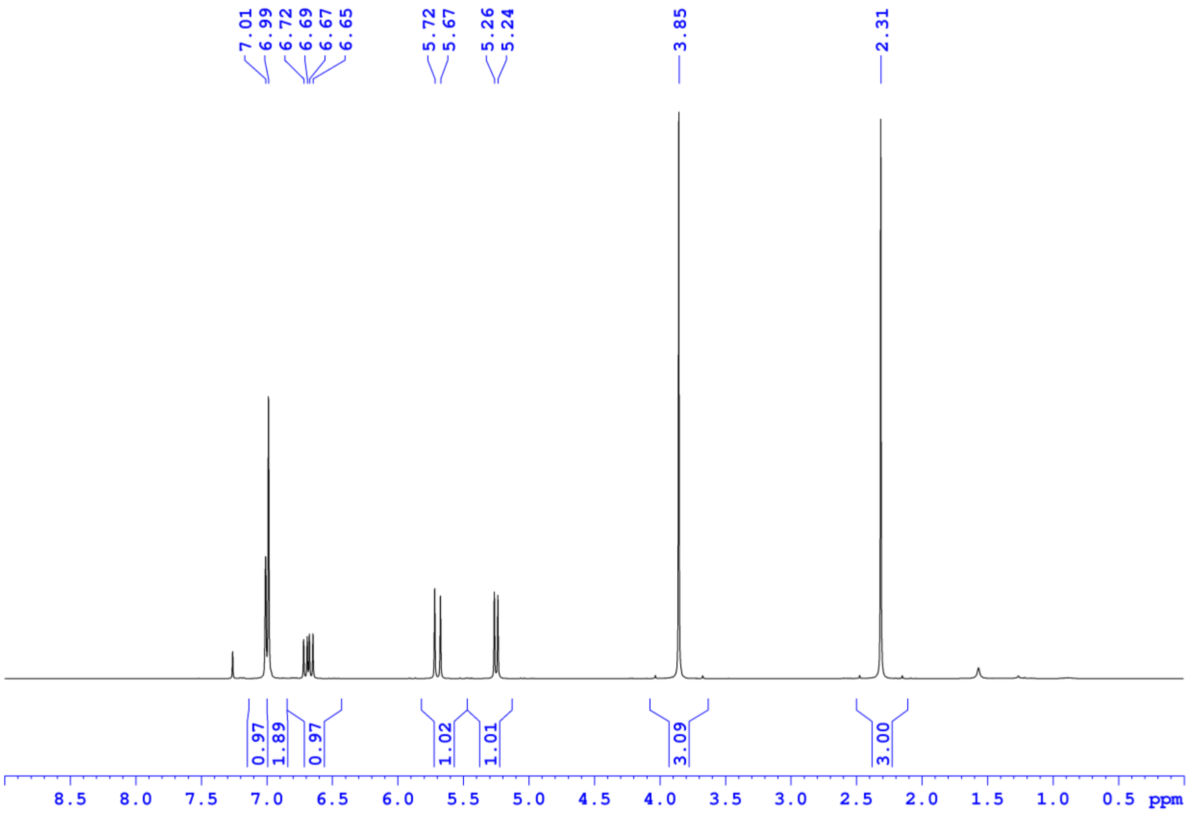

FigureS 3. ^{1}H NMR of 4-vinyl guaiacol acetate after column chromatography (400 MHz, rt, $CDCl_3$).

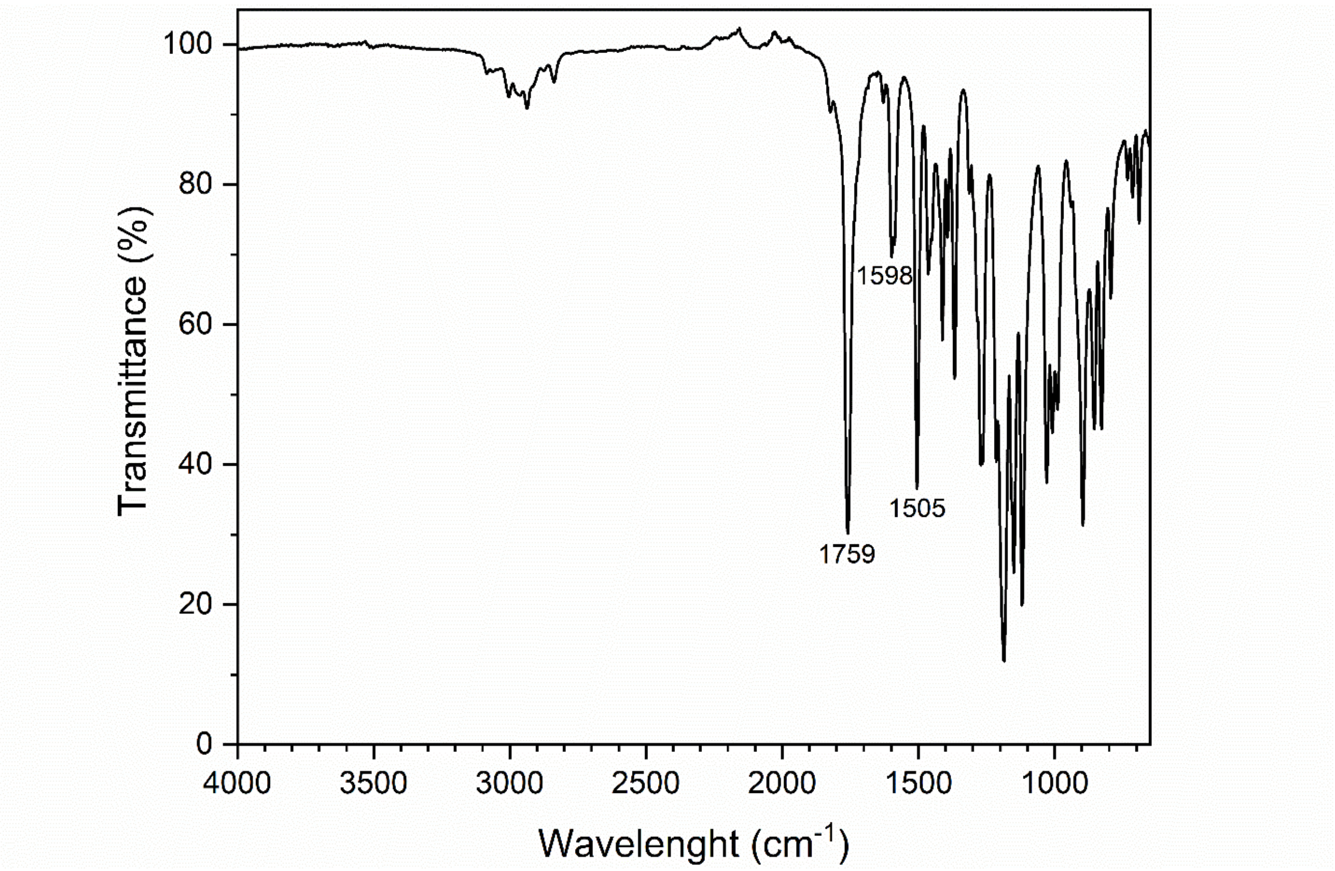

FigureS 4. ATR-FTIR of 4-vinyl guaiacol acetate.

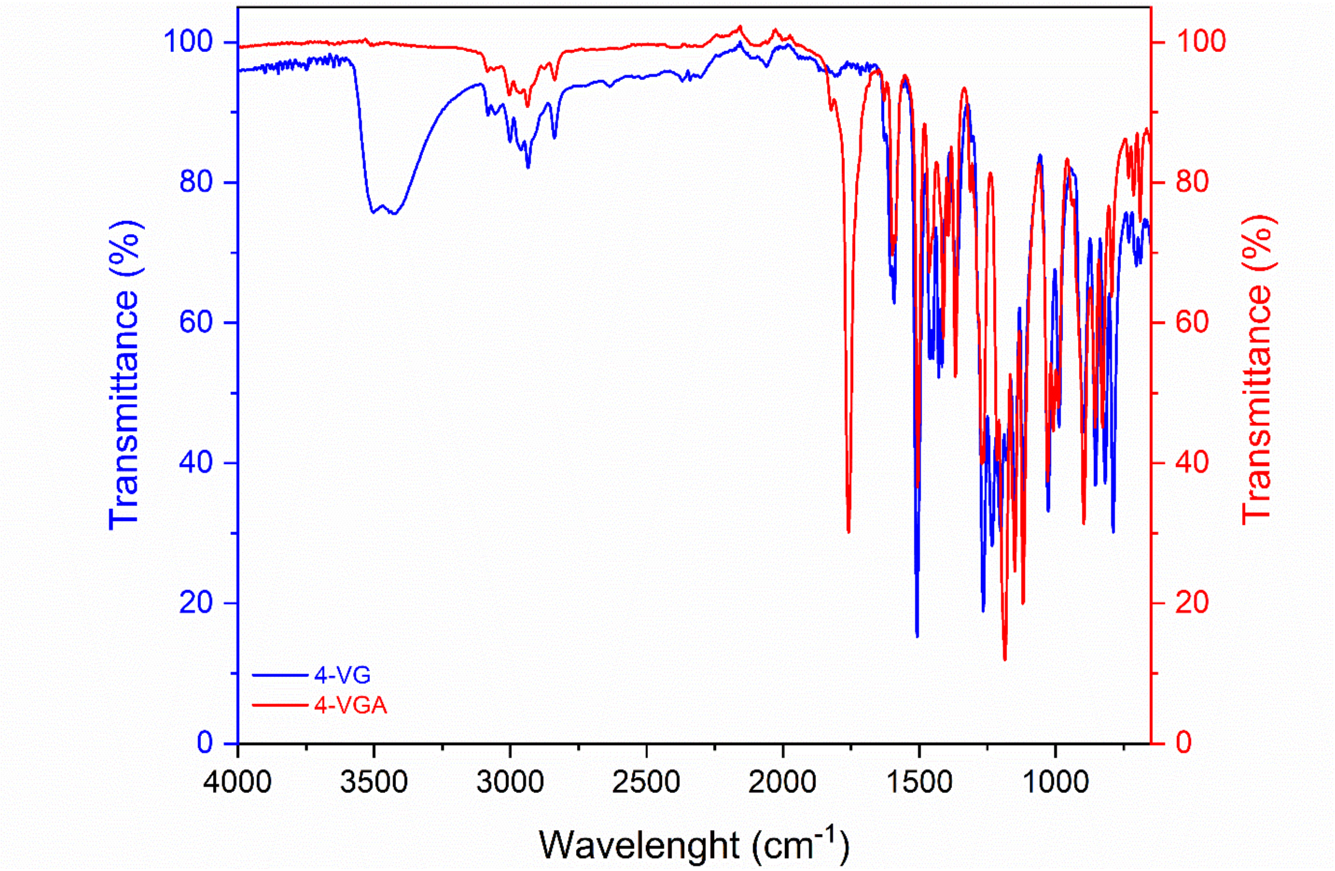

FigureS 5. Comparison of the ATR-FTIR spectra of 4-vinyl guaiacol (blue curve) and of 4-vinyl guaiacol acetate (red curve).

3.2 Synthesis of 4-vinyl guaiacol butanoate (4-VGB)

The 4-vinylguaiacol (3.00 g, 20.0 mmol) was weighted in a 25 mL round-bottomed flask equipped with a condenser, dissolved in butyric anhydride (4.9 mL, 4.74 g, 30.1 mmol), and sodium acetate (82 mg, 0.99 mmol) was added. The reaction mixture was stirred at 90 °C for 1 hour, then allowed to cool down to room temperature and diluted with Et_2O (35 mL). The organic phase was extracted sequentially with 1 M HCl (3 × 35 mL), saturated Na_2CO_3 (3 × 35 mL), deionised water (3 × 35 mL), and brine (2 × 35 mL). The organic phase was dried over anhydrous sodium sulfate, the solvent removed by rotary evaporation at reduced pressure, and the product purified by column chromatography on silica gel using a solvent mixture of petroleum ether/EtOAc = 9/1, yielding 4-VGB (yield = 87.5%, 3.85 g, 17.5 mmol).

NMR Spectroscopic data of 4-VGB

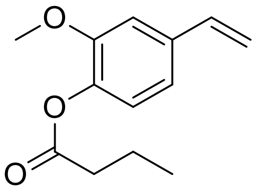

^{1}H NMR (400 MHz, $CDCl_3$, rt): δ 7.00-6.98 (*m*, 3H, Ar-H); 6.68 (*dd*, 1H, *J* = 17.6 Hz, *J* = 10.8 Hz, -C*H*=CH_2); 5.69 (*d*, 1H, *J* = 17.6 Hz, *trans*-CH=CH_2); 5.24 (*d*, 1H, *J* = 10.8 Hz, *cis*-CH=CH_2); 3.84 (*s*, 1H, -OCH_3); 2.56 (*t*, 2H, -OCOC$H_2$$CH_2$$CH_3$); 1.80 (sext, 2H, -OCO$CH_2CH_2$$CH_3$); 1.05 (*t*, 3H, -OCO$CH_2$$CH_2CH_3$).

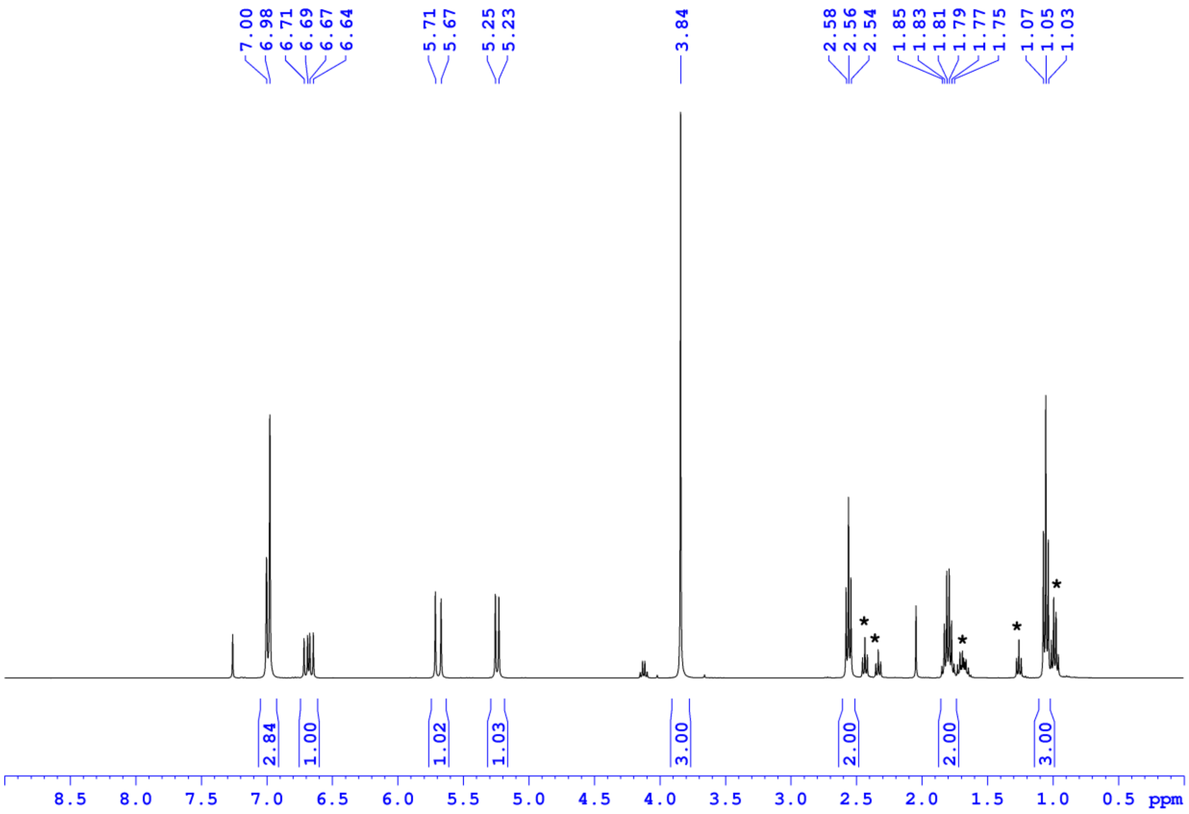

FigureS 6. ^{1}H NMR of 4-vinyl guaiacol butanoate (400 MHz, rt, $CDCl_3$). Signals due to butanoic acid and butanoic anhydride impurities are marked with *.

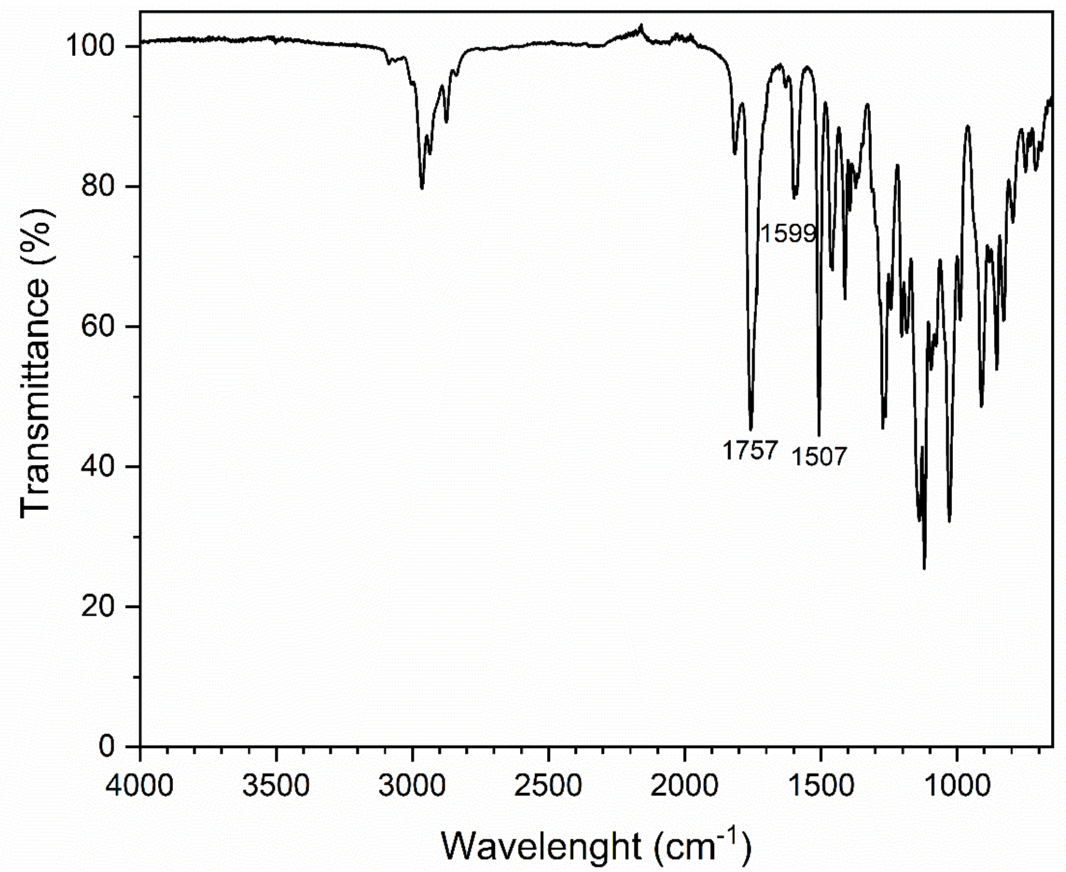

FigureS 7. ATR-FTIR of 4-vinyl guaiacol butanoate.

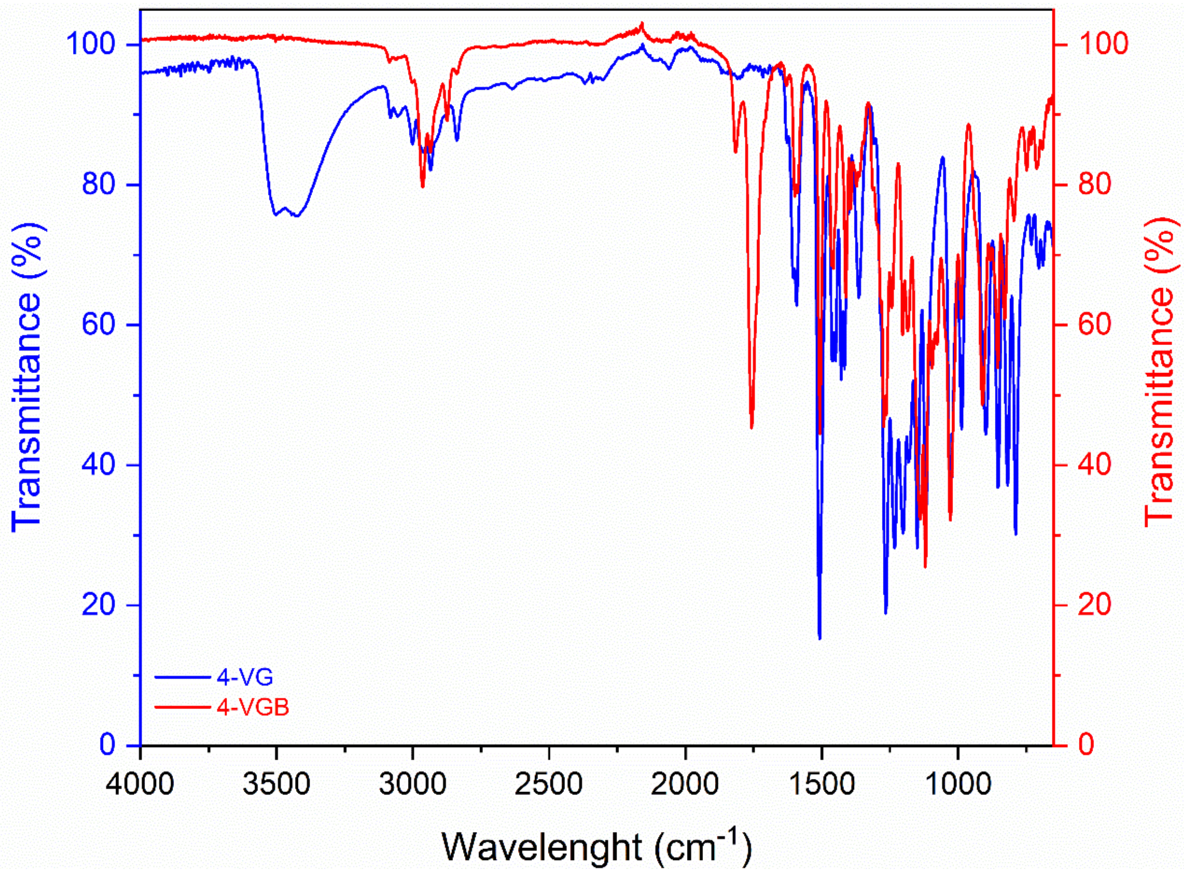

FigureS 8. Comparison of the ATR-FTIR spectra of 4-vinyl guaiacol (blue curve) and of 4-vinyl guaiacol butanoate (red curve).

3.3 Synthesis of 4-vinyl guaiacol hexanoate (4-VGH)

The 4-vinylguaiacol (3.00 g, 20.0 mmol) was weighted in a 25 mL round-bottomed flask equipped with a condenser, dissolved in hexanoic anhydride (6.9 mL, 6.40 g, 29.9 mmol), and sodium acetate (82 mg, 0.99 mmol) was added. The reaction mixture was stirred at 90 °C for 1 hour, then allowed to cool down to room temperature and diluted with Et_2O (35 mL). The organic phase was extracted sequentially with 1 M HCl (3 × 35 mL), saturated Na_2CO_3 (3 × 35 mL), deionised water (3 × 35 mL), and brine (2 × 35 mL). The organic phase was dried over anhydrous sodium sulfate, the solvent removed by rotary evaporation at reduced pressure, and the product purified by column chromatography on silica gel using a solvent mixture of petroleum ether/EtOAc = 9/1, yielding 4-VGH (yield = 73.0%, 3.86 g, 14.6 mmol).

NMR Spectroscopic data of 4-VGH

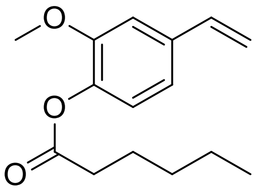

^{1}H NMR (400 MHz, $CDCl_3$, rt): δ 7.00-6.98 (*m*, 3H, Ar-H); 6.68 (*dd*, 1H, *J* = 17.6 Hz, *J* = 10.8 Hz, -C*H*=CH_2); 5.69 (*d*, 1H, *J* = 17.6 Hz, *trans*-CH=C*H*$_2$); 5.24 (*d*, 1H, *J* = 10.8 Hz, *cis*-CH=C*H*$_2$); 3.84 (*s*, 1H, -OC*H*$_3$); 2.57 (*t*, 2H, -OCOC*H*$_2$$CH_2CH_2CH_2CH_3$); 1.77 (*quint*, 2H, -OCOCH_2C*H*$_2$$CH_2CH_2CH_3$); 1.45-1.37 (*m* overlapped, 4H, -OCOCH_2CH_2C*H*$_2$C*H*$_2$$CH_3$); 0.93 (*t*, 3H, -OCO$CH_2CH_2CH_2CH_2$C*H*$_3$).

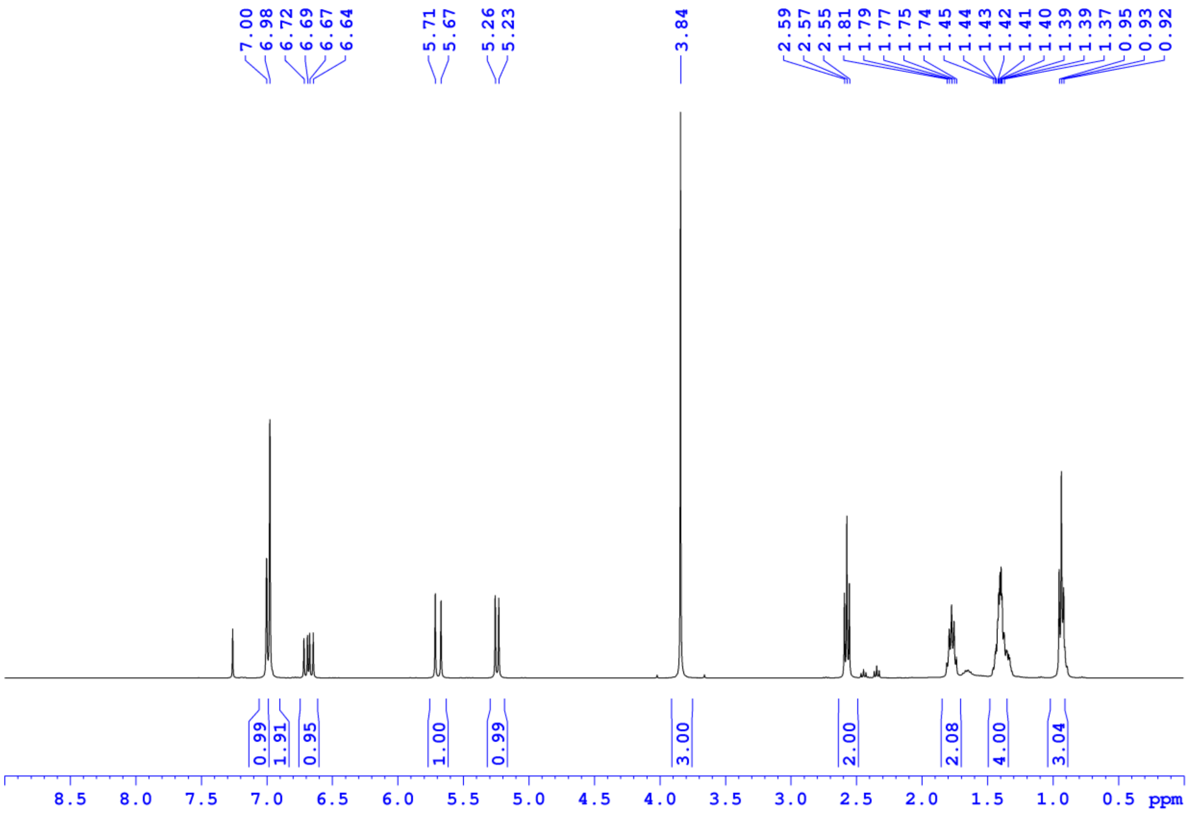

FigureS 9. ^{1}H NMR of 4-vinyl guaiacol hexanoate (400 MHz, rt, $CDCl_3$).

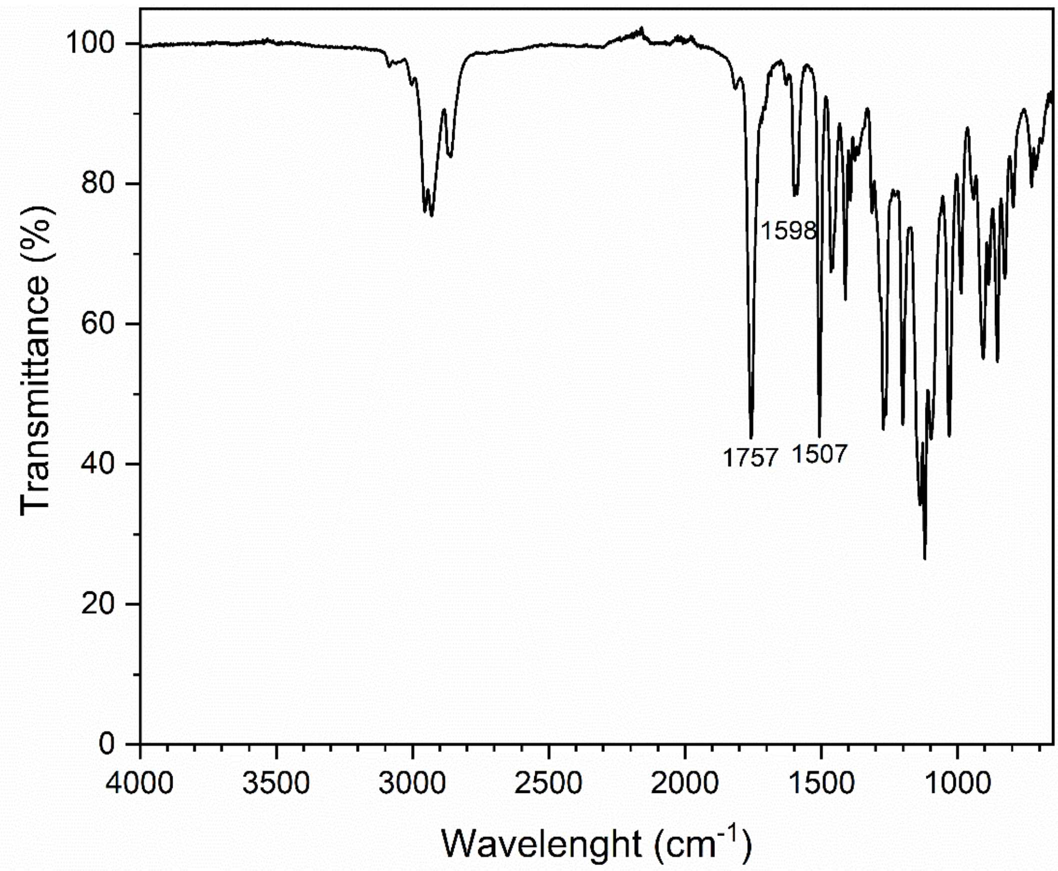

FigureS 10. ATR-FTIR of 4-vinyl guaiacol hexanoate.

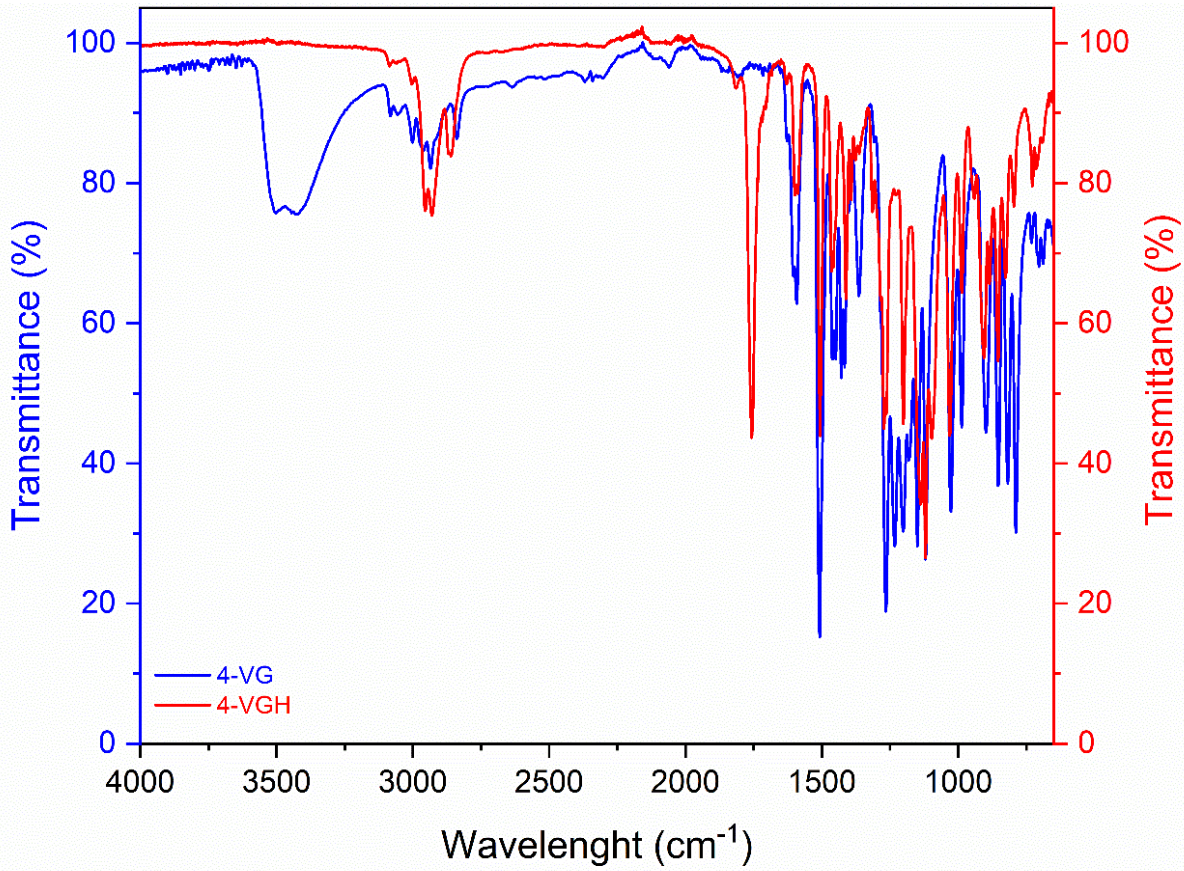

FigureS 11. Comparison of the ATR-FTIR spectra of 4-vinyl guaiacol (blue curve) and of 4-vinyl guaiacol hexanoate (red curve).

4. Synthesis of 4-epoxy guaiacol acylates

The guaiacol-based epoxy monomers were prepared via epoxidation of 4-guaiacol acylates, following a procedure similar to that reported for structurally similar substituted styrenes.[74]

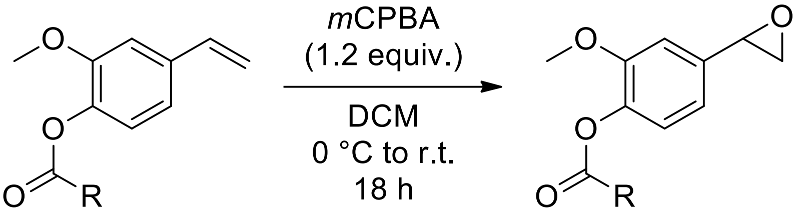

4.1 Synthesis of 4-epoxy guaiacol acetate (EGA)

The 4-VGA (1.04 g, 5.41 mmol) was weighed into a vial and transferred to a 50 mL round-bottomed flask. After dissolving in dichloromethane (25 mL, [4-VGA] = 0.2 M), the solution was cooled to 0 °C with an ice bath. Meta-chloroperbenzoic acid, *m*CPBA (77 % w/w, 1.49 g, 6.65 mmol) was added portion-wise during 15 minutes, and the suspension was stirred for further 18 hours, allowing to warm up from 0 °C to room temperature. Dichloromethane (40 mL) was added to the suspension and the solution was sequentially washed with saturated $Na_2S_2O_3$ (3 × 25 mL), a saturated $NaHCO_3$ (3 × 25 mL), and deionized water (3 × 30 mL). The recovered organic phase was dried over Na_2SO_4 and the solvent removed by rotary evaporation under reduced pressure. The product was purified by column chromatography on silica gel using an initial solvent mixture of petroleum ether/EtOAc = 9/1, followed by petroleum ether/EtOAc = 8/2, yielding the desired EGA (0.75 g; yield = 66.6%).

NMR Spectroscopic data of EGA

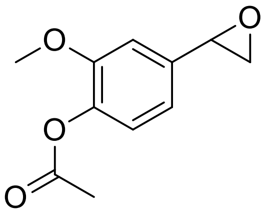

^{1}H NMR (600 MHz, $CDCl_3$, rt): δ 6.93 (*d*, 1H, *J* = 8.1 Hz, -Ar*H*); δ 6.87 (*dd*, 1H, *J* = 8.1 Hz, *J* = 1.9 Hz, -Ar*H*); 6.83 (*d*, 1H, J = 1.9 Hz, -Ar*H*); 3.82 (*dd*, 1H, *J* = 4.0 Hz, *J* = 2.6 Hz, -C*H*OCH$_2$, epoxide); 3.79 (*s*, 3H, -OC*H*$_3$); 3.09 (*dd*, 1H, *J* = 5.5 Hz, *J* = 4.0 Hz *cis*-CHOC*H*$_2$, epoxide); 2.72 (*dd*, 1H, *J* = 5.5 Hz, *J* = 2.6 Hz *trans*-CHOC*H*$_2$, epoxide); 2.27 (*s*, 1H, -OCOC*H*$_3$). ^{13}C NMR (151 MHz, $CDCl_3$, rt): δ 168.94; 151.33; 139.54; 136.67; 122.75; 117.97; 108.91; 55.81; 52.04; 51.15; 20.54.

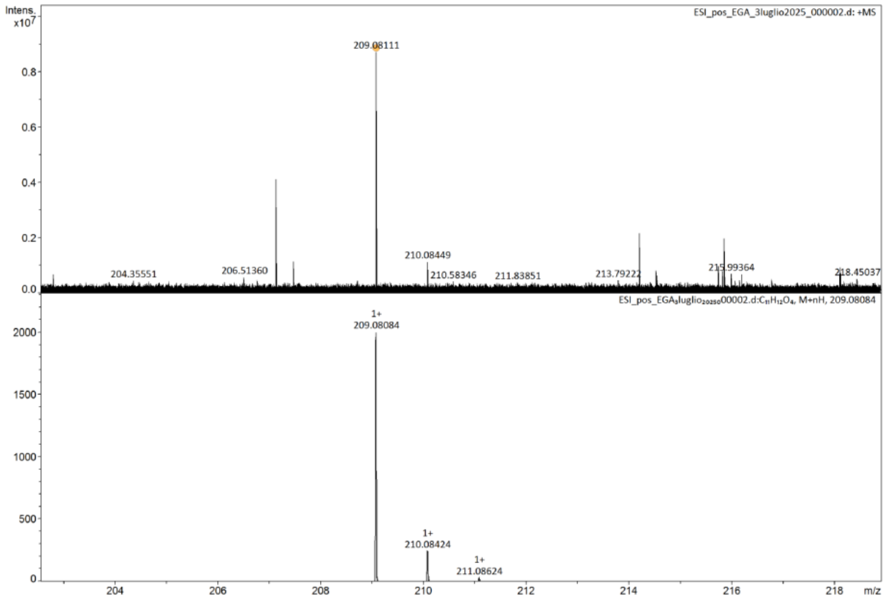

FigureS 12. High Resolution ESI MS measured (top) and calculated (bottom) spectra of 4-epoxy guaiacol acetate in MeOH, region from 203 to 219 m/z. $[C_{11}H_{13}O_4]^+$ calcd. = 209.08084 uma; measured = 209.08111 uma.

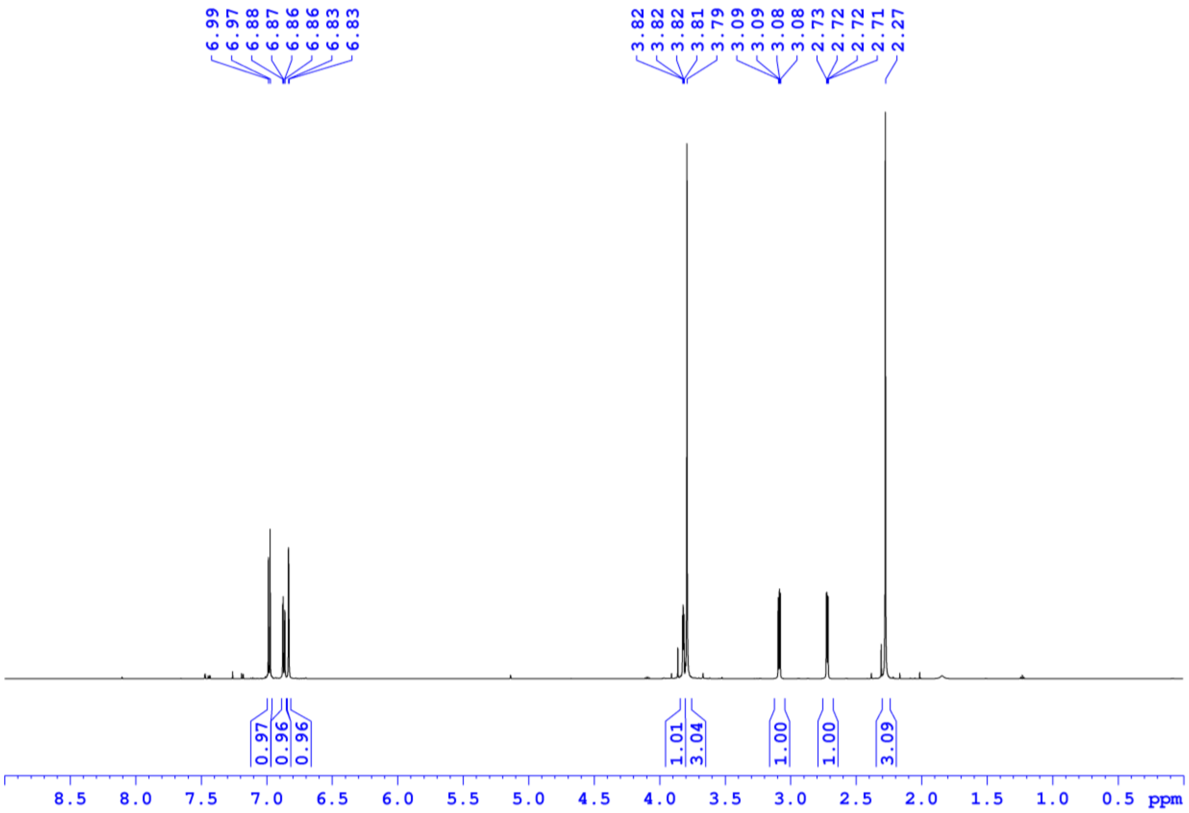

FigureS 13. ^{1}H NMR of 4-epoxy guaiacol acetate (600 MHz, rt, $CDCl_3$).

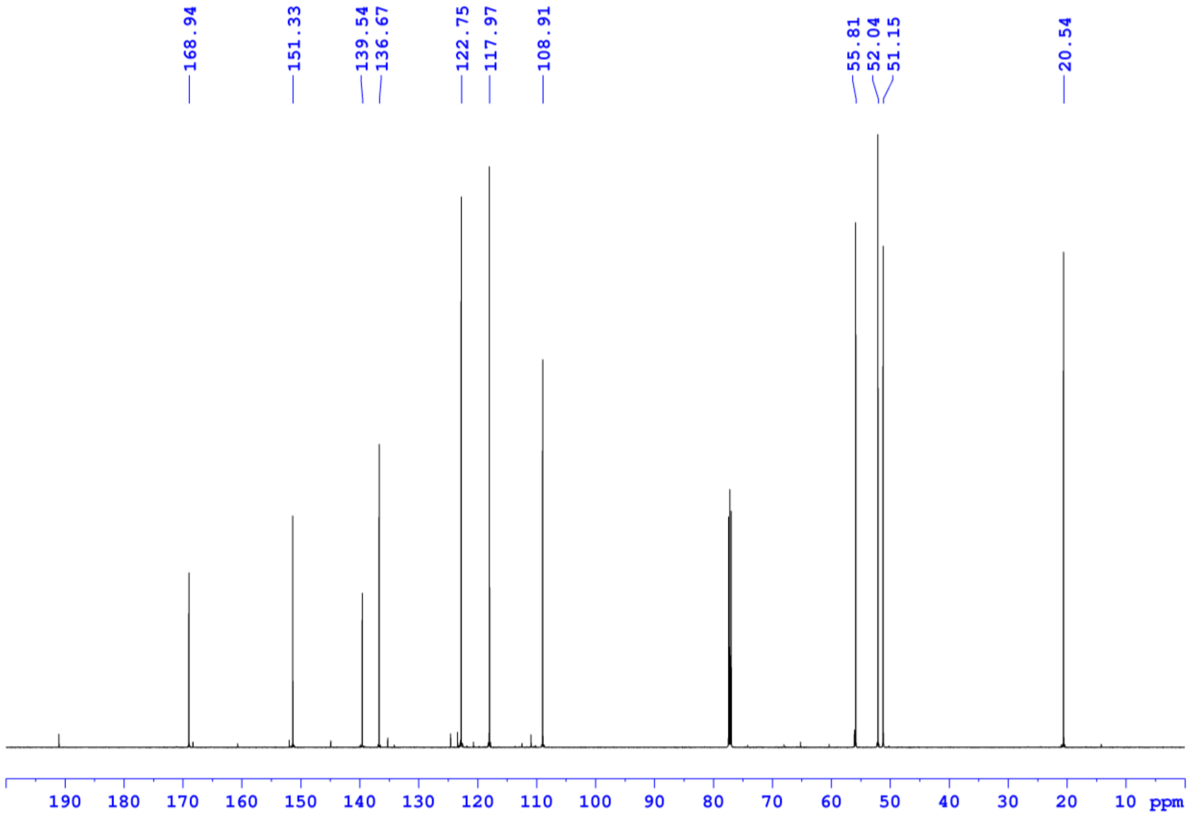

FigureS 14. ^{13}C NMR of 4-epoxy guaiacol acetate (151 MHz, rt, $CDCl_3$).

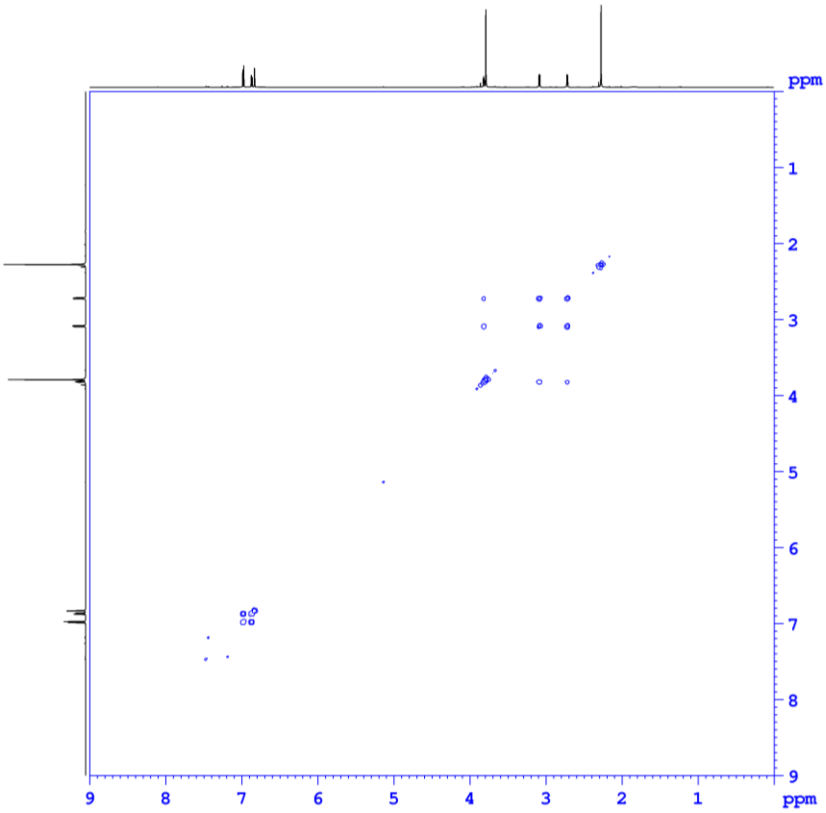

FigureS 15. ^{1}H-^{1}H COSY NMR of 4-epoxy guaiacol acetate (600 MHz, rt, $CDCl_3$).

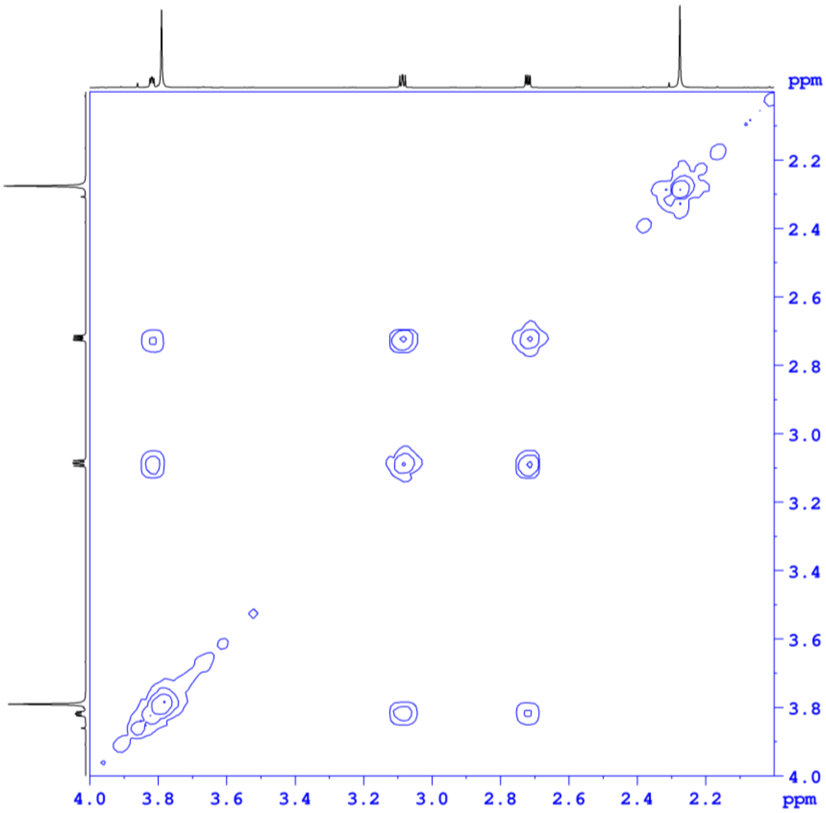

FigureS 16. ^{1}H-^{1}H COSY NMR of 4-epoxy guaiacol acetate from 4.0 to 2.0 ppm (600 MHz, rt, $CDCl_3$).

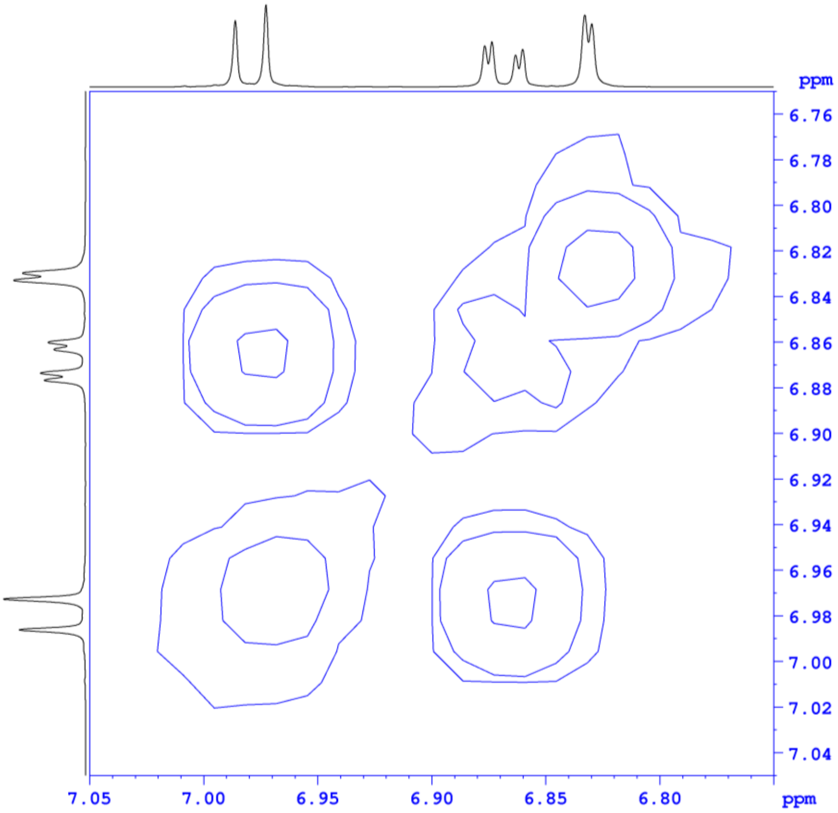

FigureS 17. ^{1}H-^{1}H COSY NMR of 4-epoxy guaiacol acetate from 7.05 to 6.75 ppm (600 MHz, rt, $CDCl_3$).

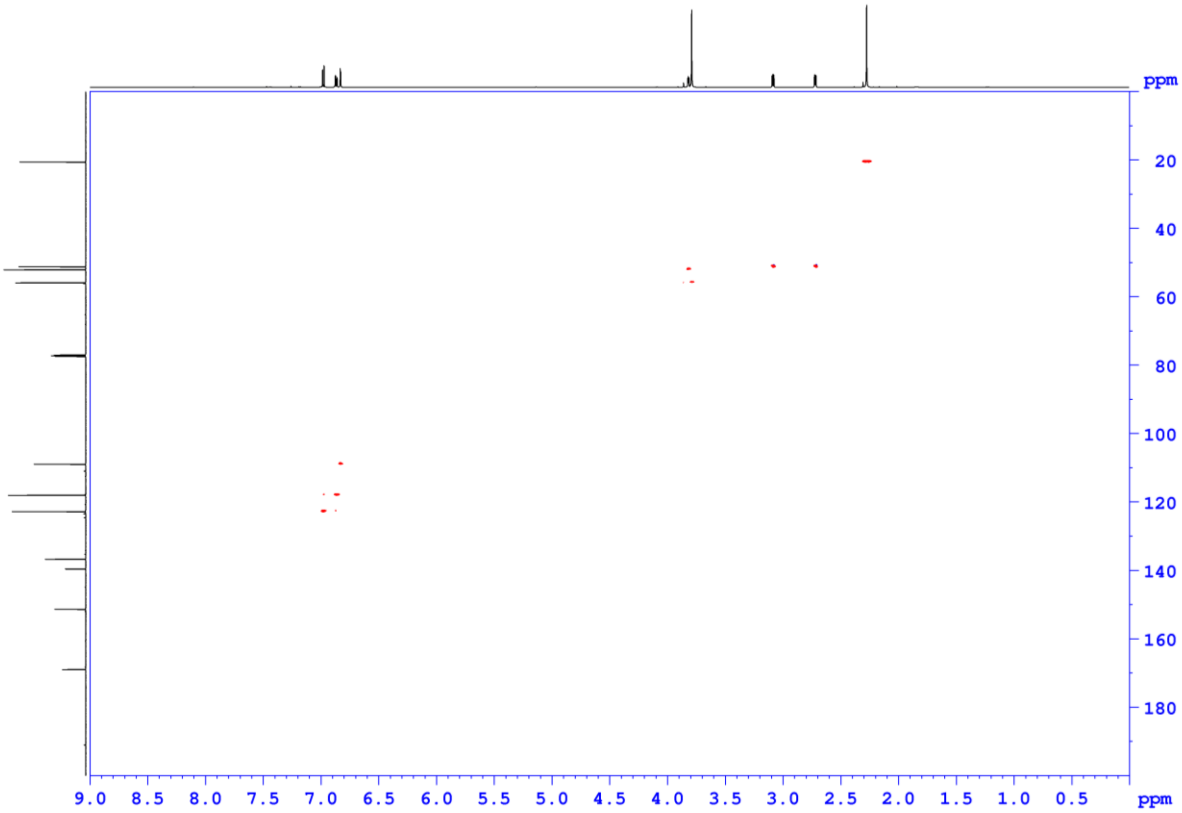

FigureS 18. ^{1}H-^{13}C HSQC NMR of 4-epoxy guaiacol acetate (600 MHz, rt, $CDCl_3$).

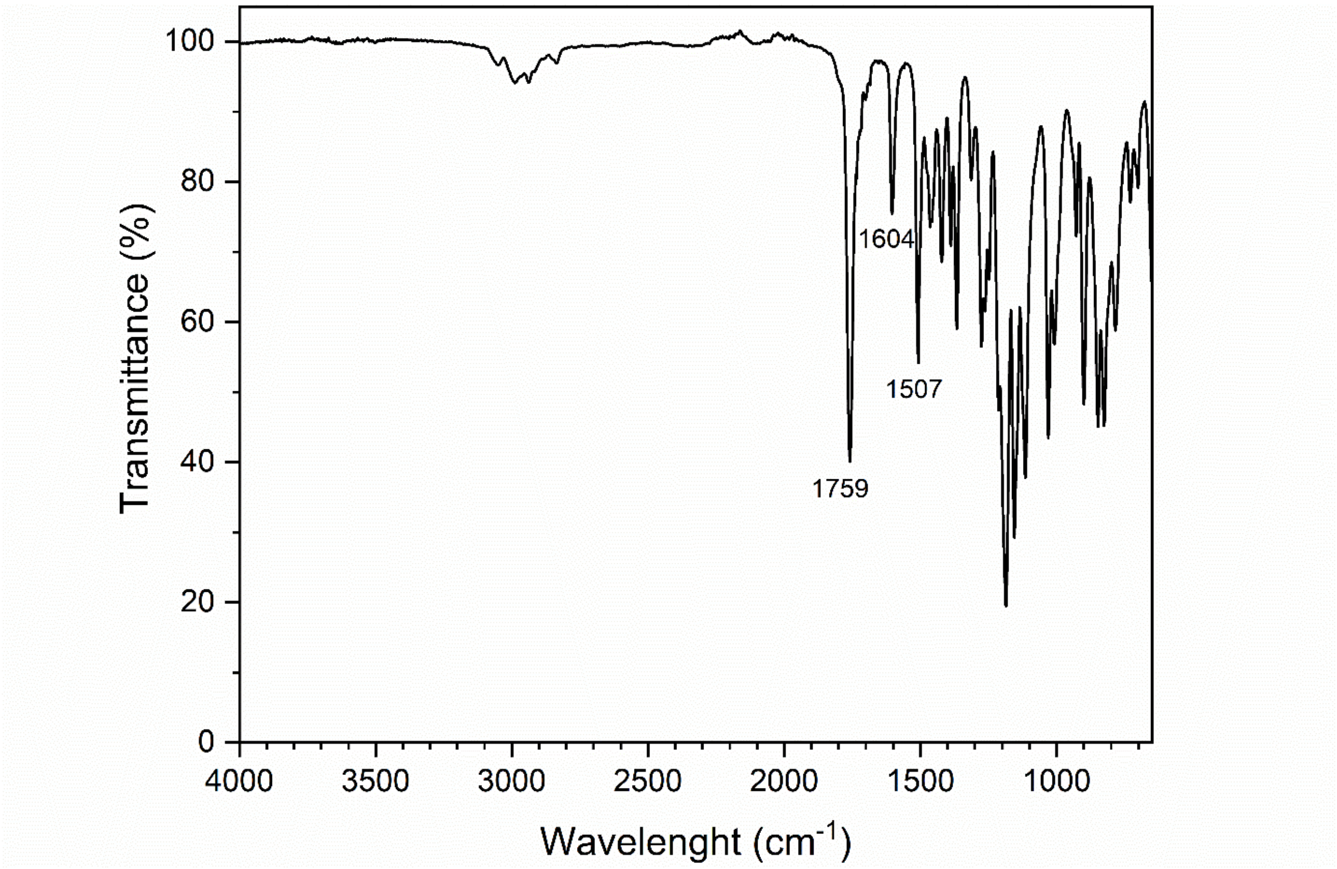

FigureS 19. ATR-FTIR of 4-epoxy guaiacol acetate.

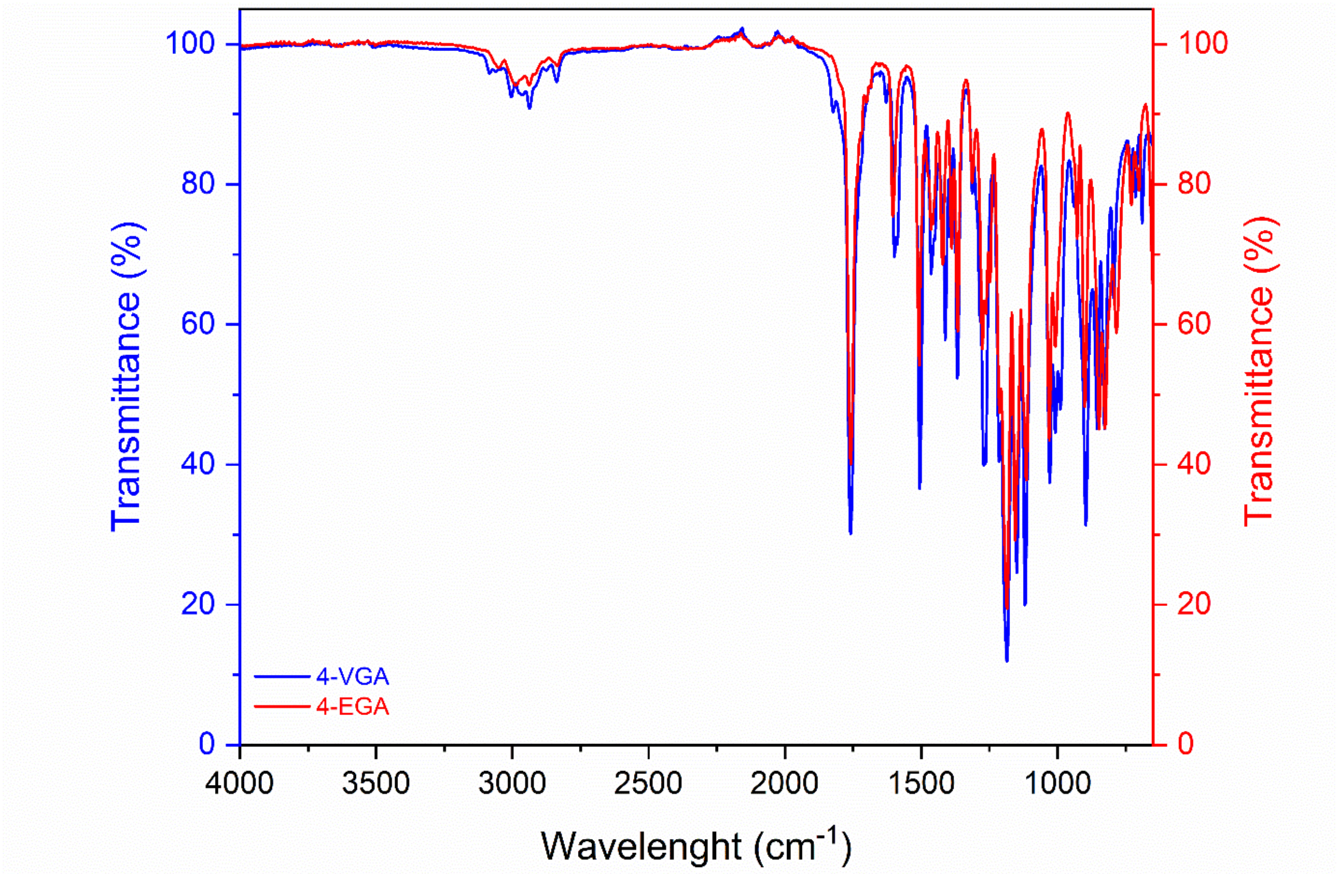

FigureS 20. Comparison of the ATR-FTIR spectra of 4-vinyl guaiacol acetate (blue curve) and of 4-epoxy guaiacol acetate (red curve).

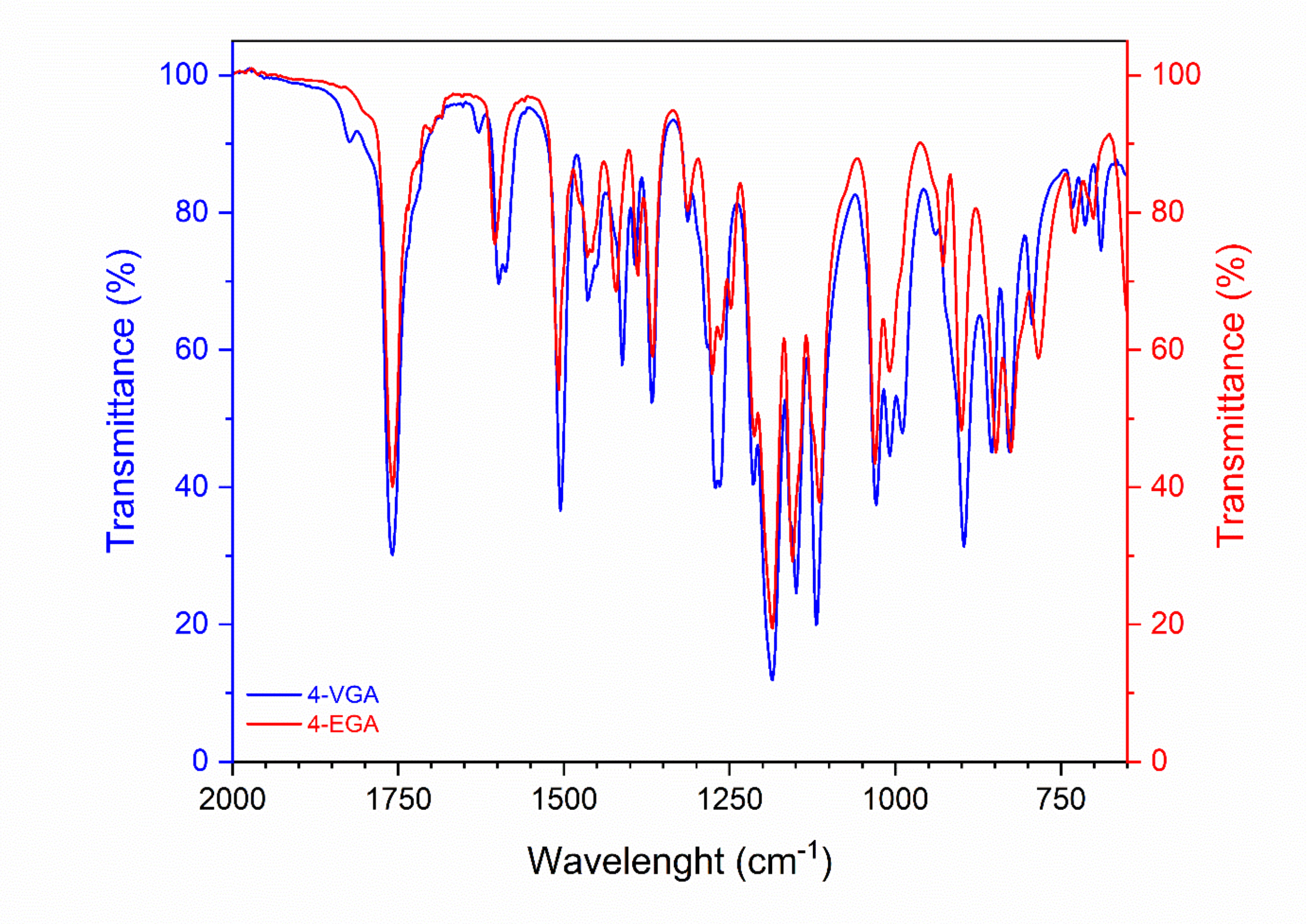

FigureS 21. Comparison of the ATR-FTIR spectra of 4-vinyl guaiacol acetate (blue curve) and of 4-epoxy guaiacol acetate (red curve). Region from 2000 to 650 cm^{-1}.

4.2 Synthesis of 4-epoxy guaiacol butanoate (VGB)

The 4-VGB (3.85 g, 17.48 mmol) was weighed into a vial and transferred to a 250 mL round-bottomed flask. After dissolving in dichloromethane (85 mL, [4-VGB] = 0.2 M), the solution was cooled to 0 °C with an ice bath. Meta-chloroperbenzoic acid, *m*CPBA (77 % w/w, 4.86 g, 21.69 mmol) was added portion-wise during 15 minutes, and the suspension was stirred for further 18 hours, allowing to warm up from 0 °C to room temperature. Dichloromethane (60 mL) was added to the suspension and the solution was sequentially washed with saturated $Na_2S_2O_3$ (3 × 75 mL), saturated $NaHCO_3$ (3 × 75 mL), and deionized water (3 × 75 mL). The recovered organic phase was dried over Na_2SO_4 and the solvent removed by rotary evaporation under reduced pressure. The product was purified by column chromatography on silica gel using an initial solvent mixture of petroleum ether/EtOAc = 9/1, followed by petroleum ether/EtOAc = 8/2, yielding the desired EGB (2.80 g; yield = 67.8%).

NMR Spectroscopic data of EGB

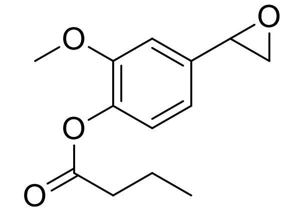

^{1}H NMR (400 MHz, $CDCl_3$, rt): δ 6.99 (*d*, 1H, *J* = 8.1 Hz, -Ar*H*); δ 6.89 (*dd*, 1H, *J* = 8.1 Hz, *J* = 1.3 Hz, -Ar*H*); 6.84 (*d*, 1H, J = 1.3 Hz, -Ar*H*); 3.85 (*dd*, 1H, *J* = 4.1 Hz, *J* = 2.4 Hz, -C*HO*CH_2, epoxide); 3.81 (*s*, 3H, -OCH_3); 3.13 (*dd*, 1H, *J* = 5.4 Hz, *J* = 4.1 Hz *cis*-CHOCH_2, epoxide); 2.75 (*dd*, 1H, *J* = 5.4 Hz, *J* = 2.4 Hz *trans*-CHOCH_2, epoxide); 2.55 (*t*, 2H, -OCOC$H_2$$CH_2$$CH_3$); 1.79 (sext, 2H, -OCO$CH_2CH_2$$CH_3$); 1.05 (*t*, 2H, -OCO$CH_2$$CH_2CH_3$). ^{13}C NMR (151 MHz, $CDCl_3$, rt): δ 171.67; 151.36; 139.64; 136.48; 122.75; 117.97; 108.96; 55.79; 52.09; 51.17; 35.79; 18.48; 13.50.

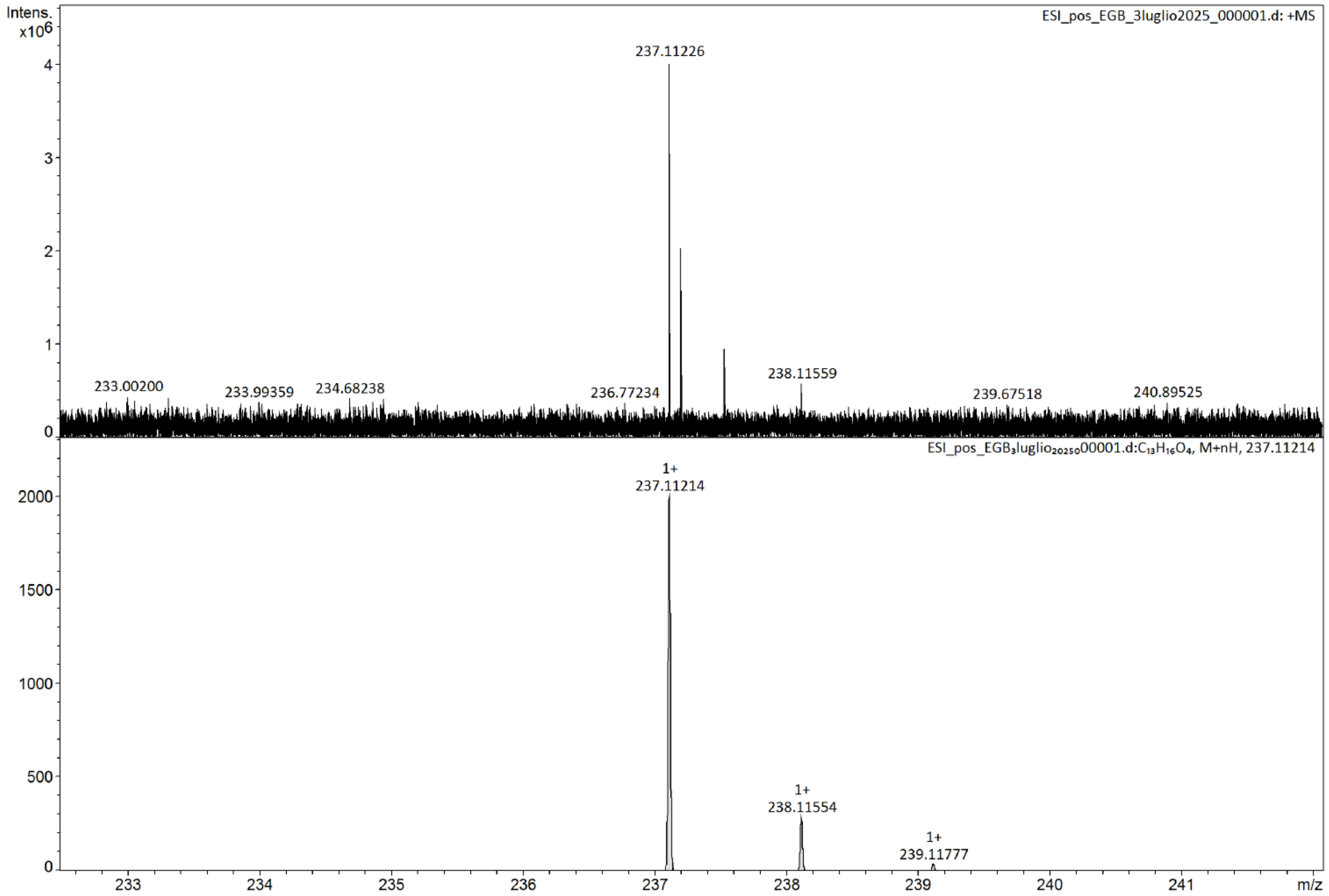

FigureS 22. High Resolution ESI MS measured (top) and calculated (bottom) spectra of 4-epoxy guaiacol butanoate in MeOH, region from 233 to 242 m/z. $[C_{11}H_{13}O_4]^+$ calcd. = 237.11214 uma; measured = 237.11226 uma.

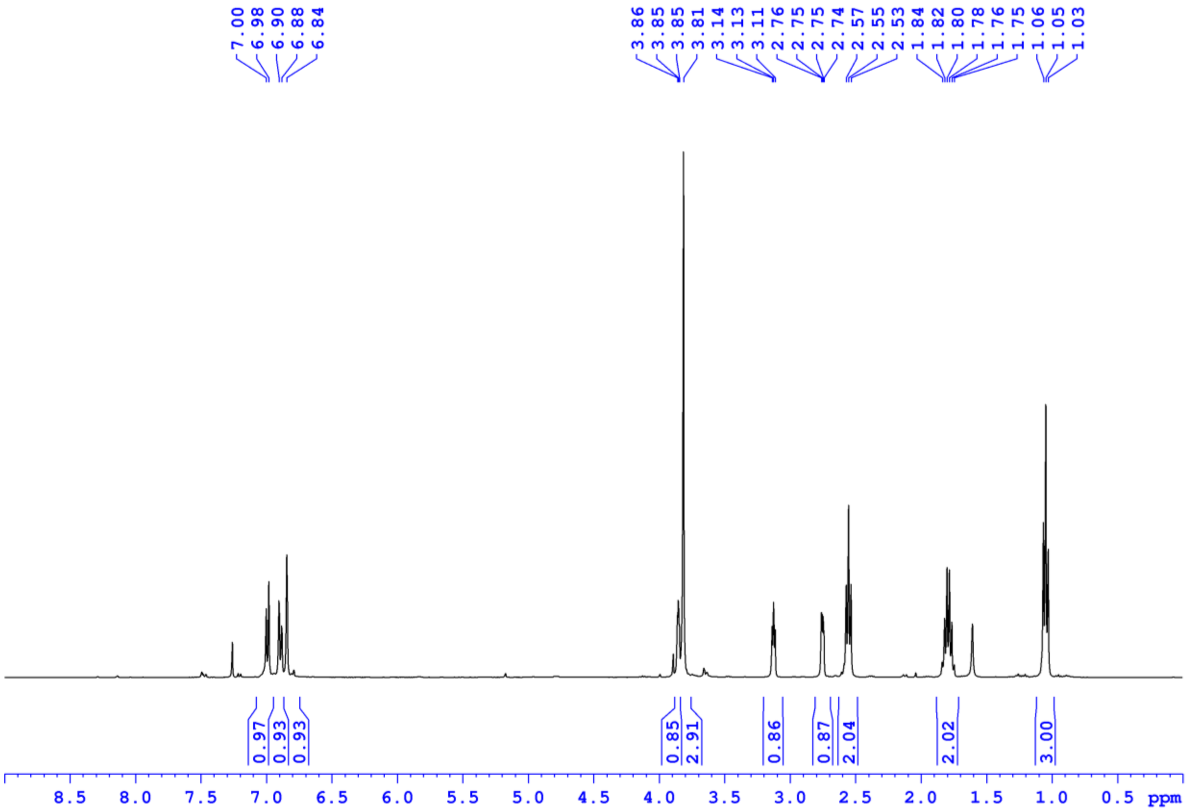

FigureS 23. ^{1}H NMR of 4-epoxy guaiacol butanoate (400 MHz, rt, $CDCl_3$).

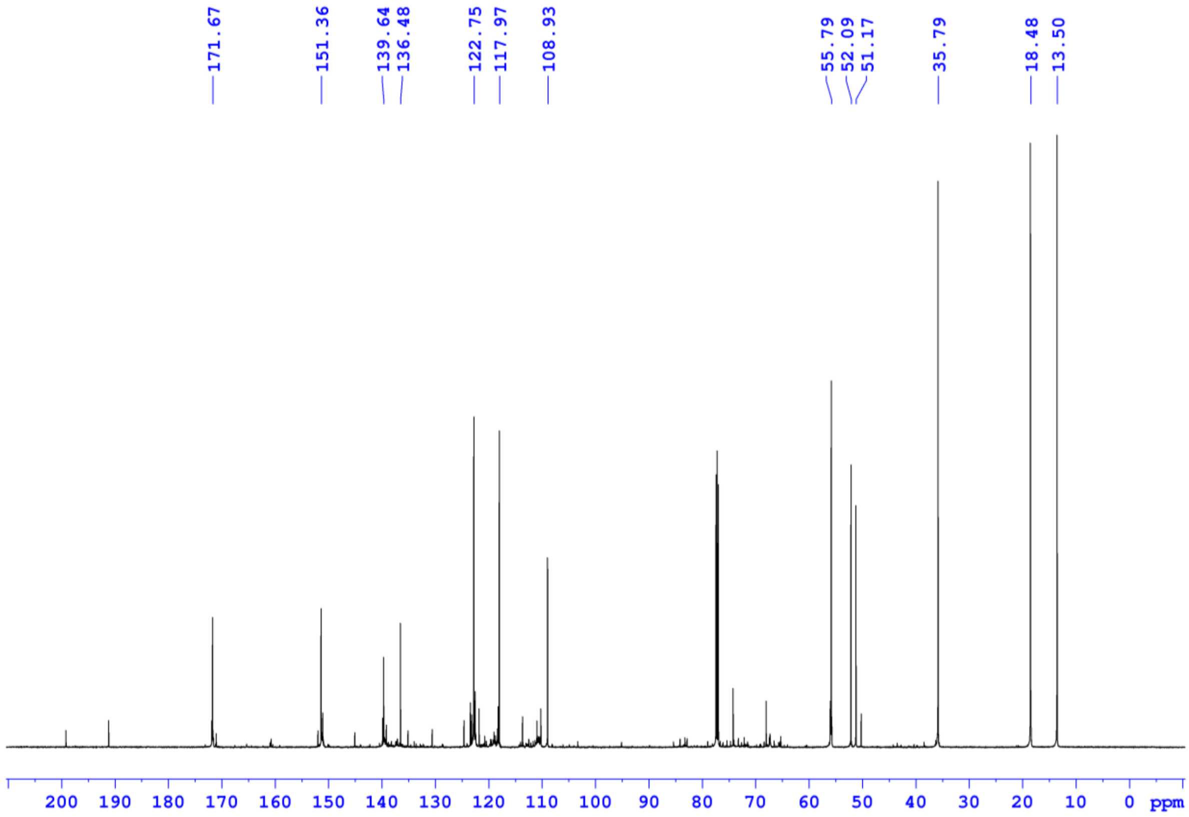

FigureS 24. ^{13}C NMR of 4-epoxy guaiacol butanoate (151 MHz, rt, $CDCl_3$).

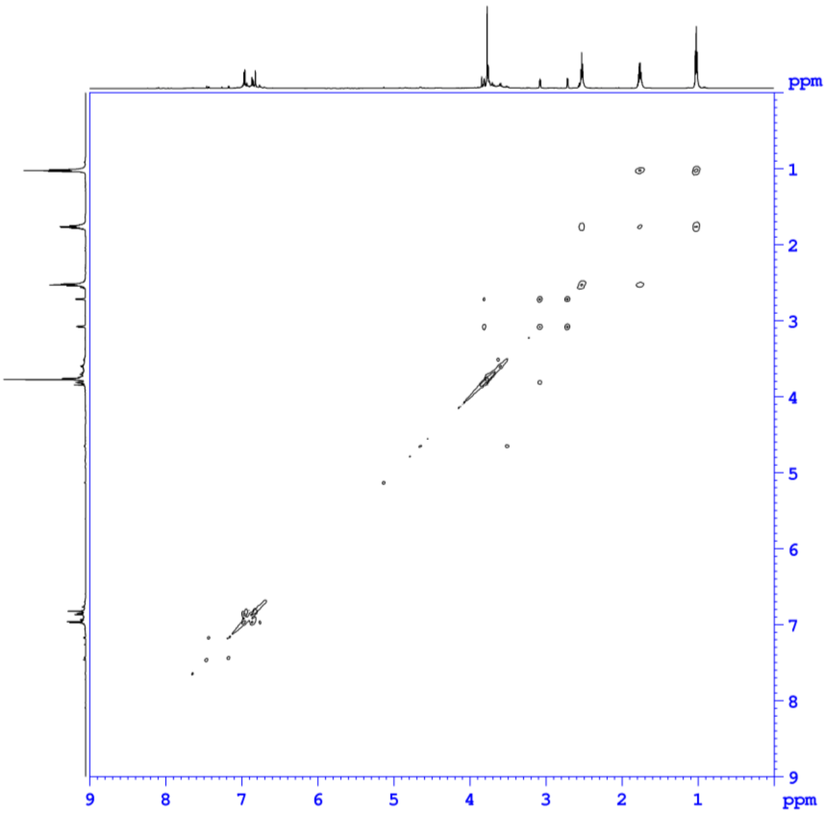

FigureS 25. ^{1}H-^{1}H COSY NMR of 4-epoxy guaiacol butanoate (600 MHz, rt, $CDCl_3$).

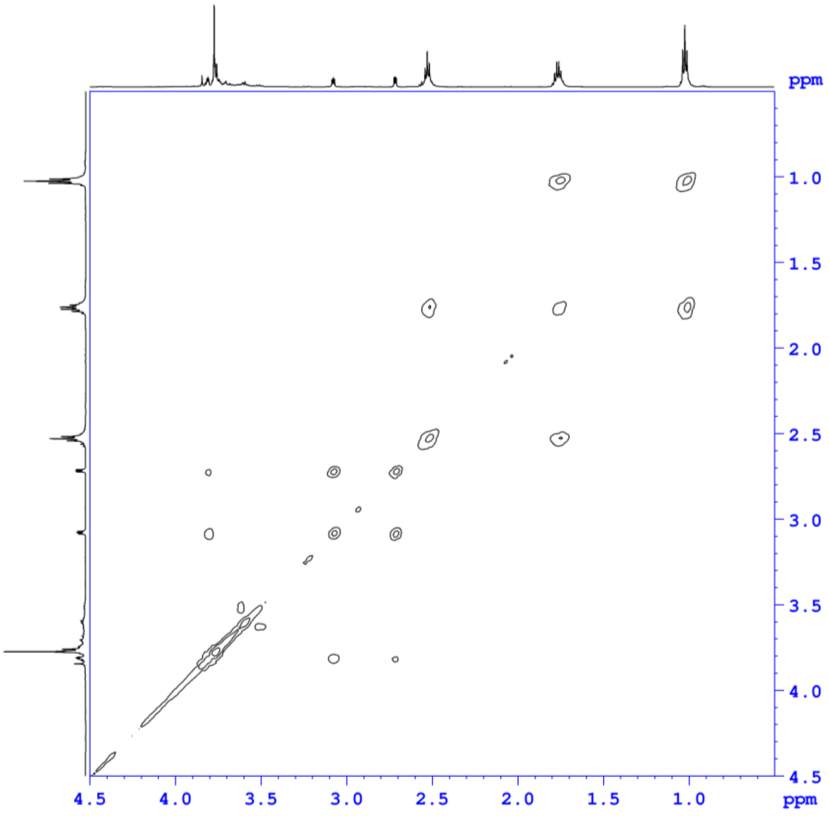

FigureS 26. ^{1}H-^{1}H COSY NMR of 4-epoxy guaiacol butanoate from 4.5 to 0.5 ppm (600 MHz, rt, $CDCl_3$).

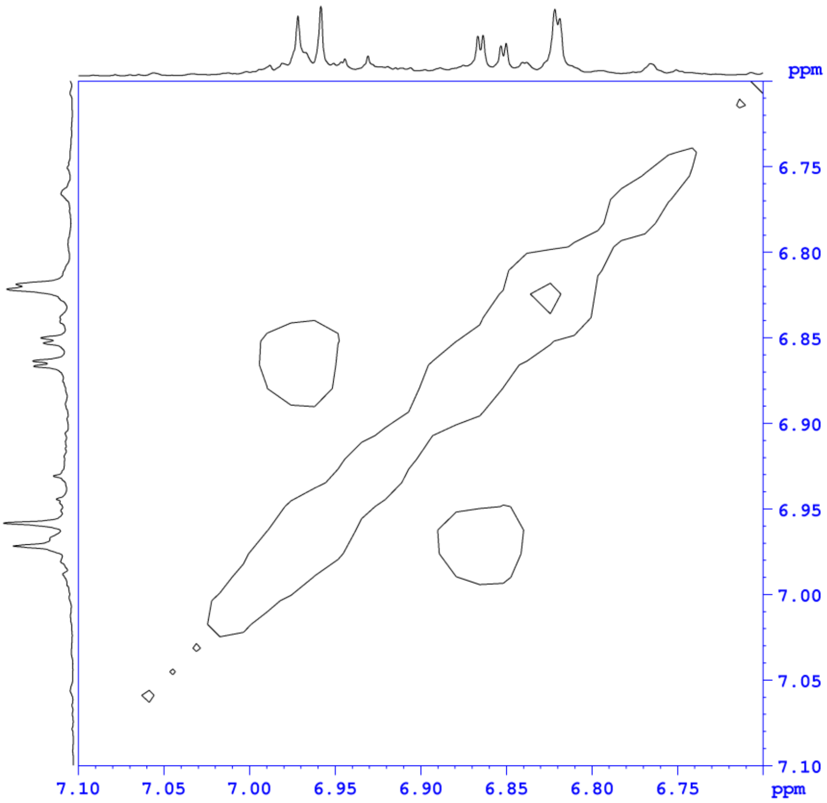

FigureS 27. ^{1}H-^{1}H COSY NMR of 4-epoxy guaiacol butanoate from 7.1 to 6.7 ppm (600 MHz, rt, $CDCl_3$).

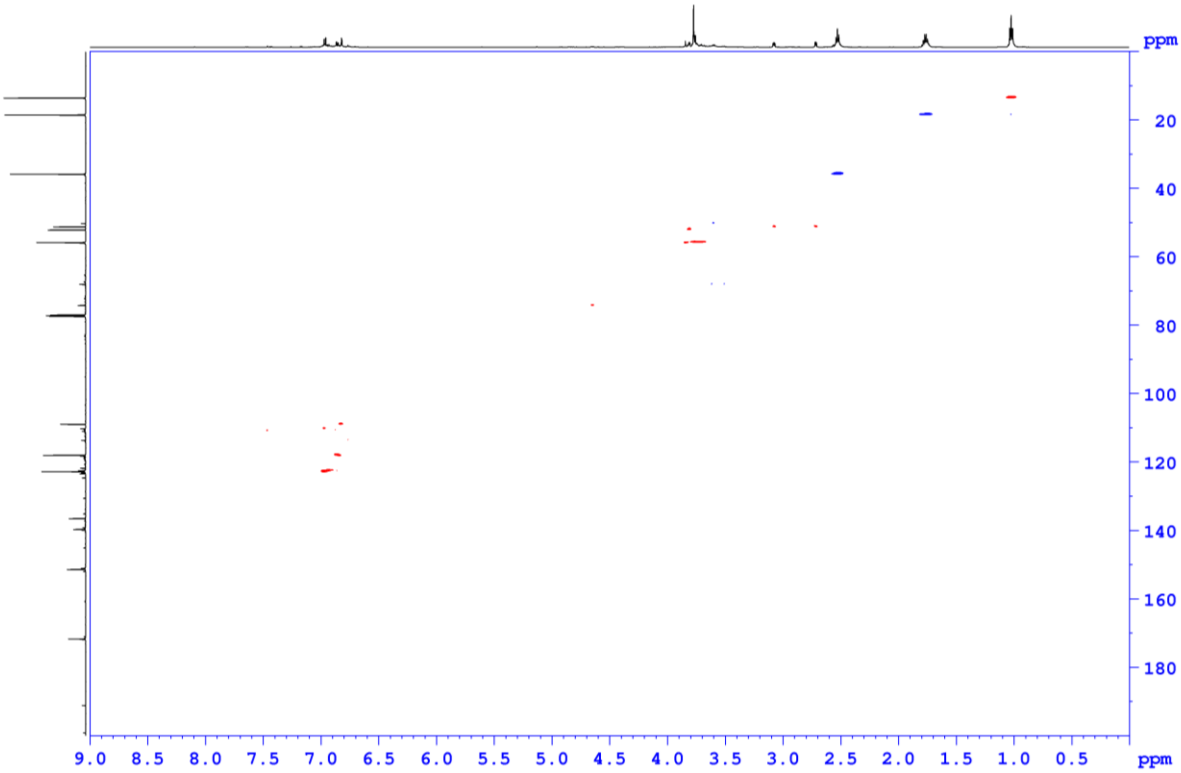

FigureS 28. ^{1}H-^{13}C HSQC NMR of 4-epoxy guaiacol butanoate (600 MHz, rt, $CDCl_3$).

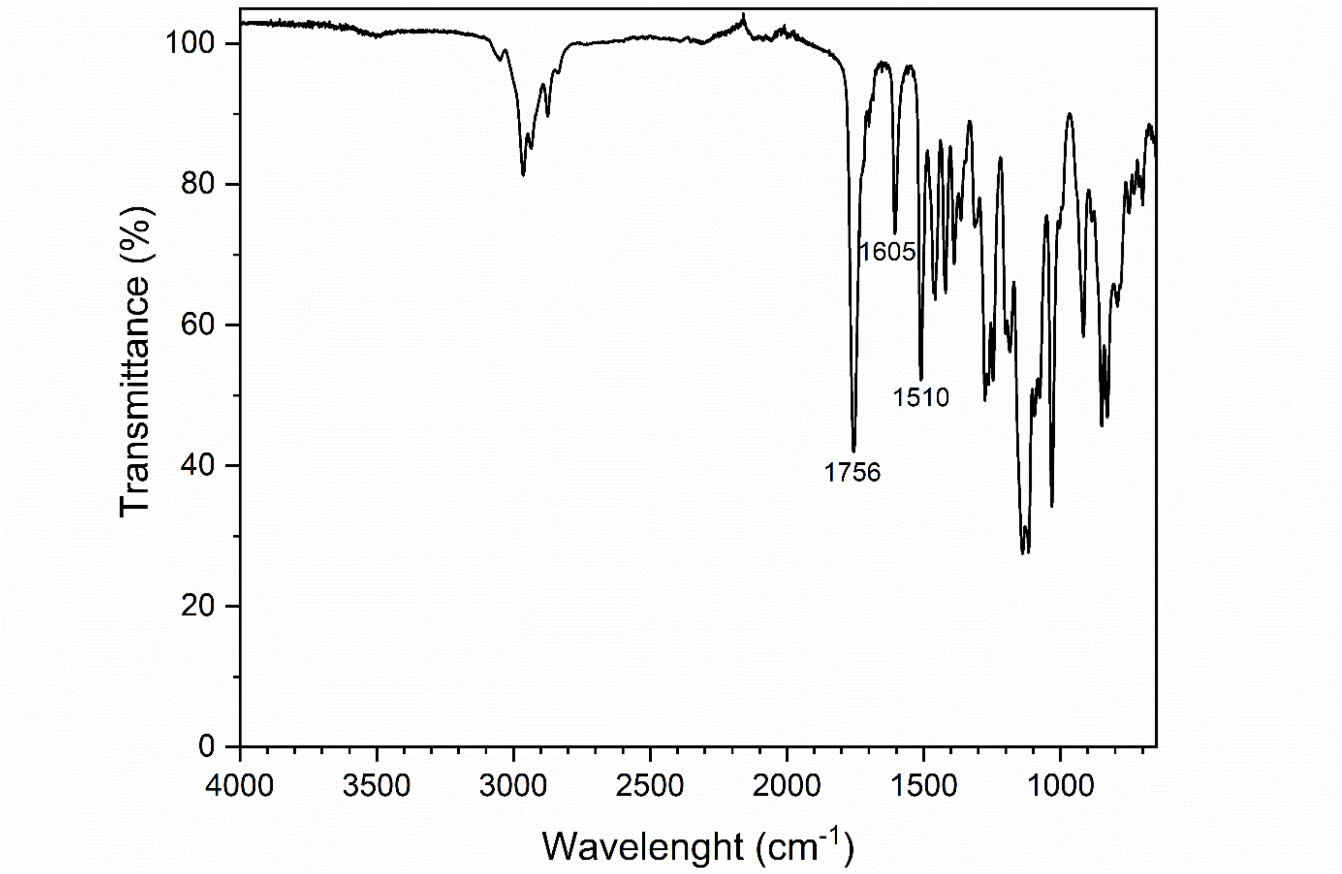

FigureS 29. ATR-FTIR of 4-epoxy guaiacol butanoate.

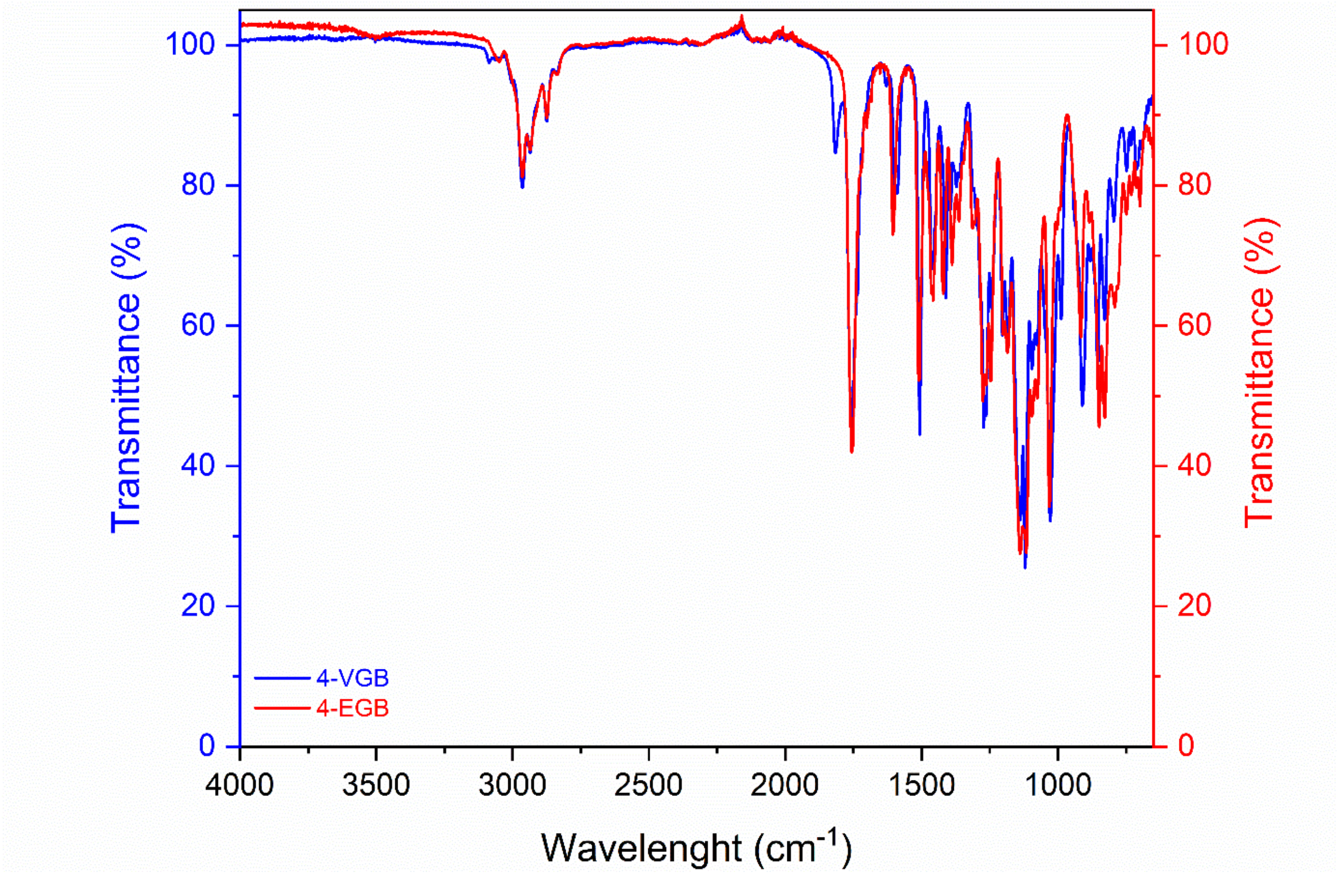

FigureS 30. Comparison of the ATR-FTIR spectra of 4-vinyl guaiacol butanoate (blue curve) and of 4-epoxy guaiacol butanoate (red curve).

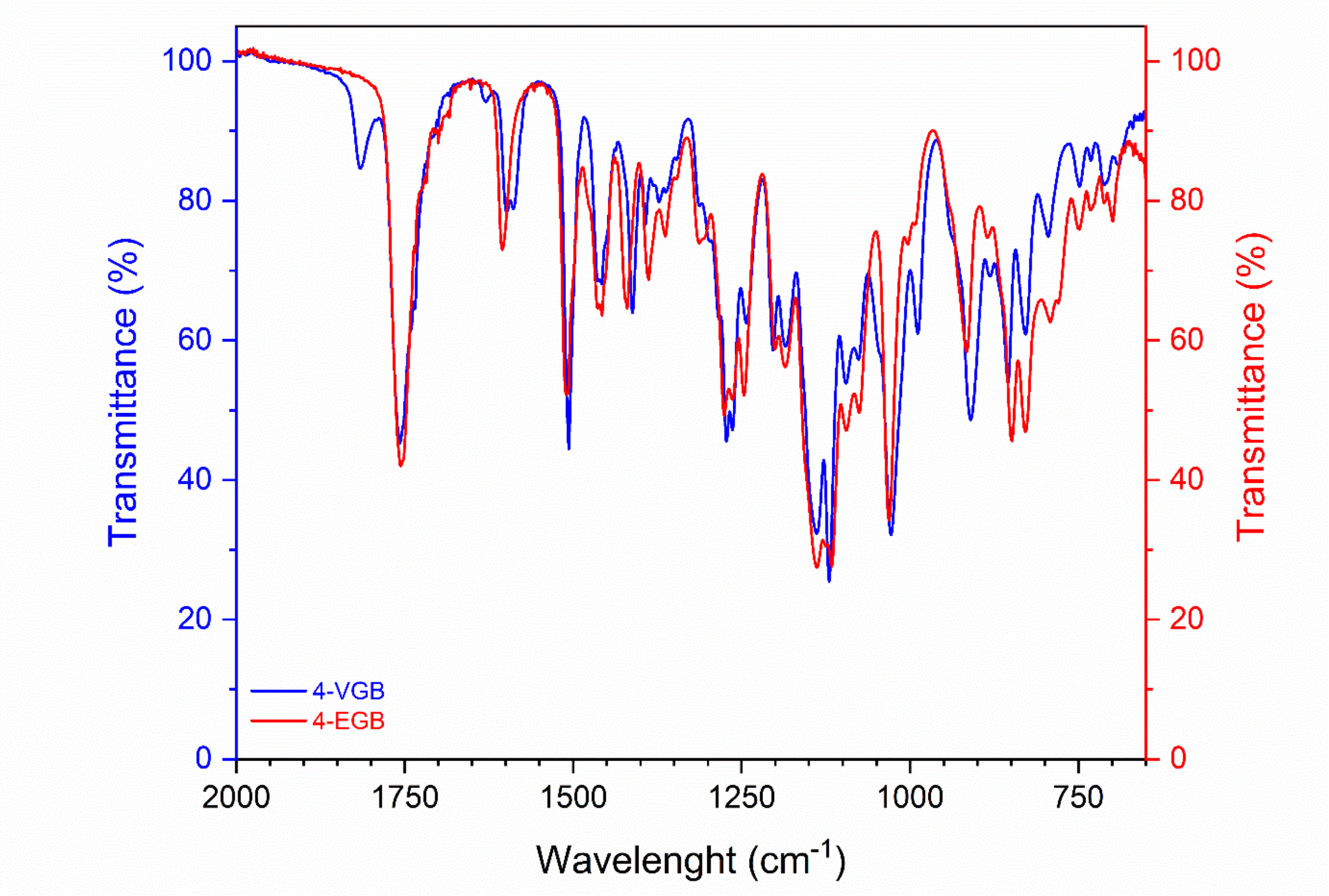

FigureS 31. Comparison of the ATR-FTIR spectra of 4-vinyl guaiacol butanoate (blue curve) and of 4-epoxy guaiacol butanoate (red curve). Region from 2000 to 650 cm^{-1}.

4.3 Synthesis of 4-epoxy guaiacol hexanoate (4-VGH)

The 4-VGH (3.59 g, 14.46 mmol) was weighed into a vial and transferred to a 250 mL round-bottomed flask. After dissolving in dichloromethane (70 mL, [4-VGB] = 0.2 M), the solution was cooled to 0 °C with an ice bath. Meta-chloroperbenzoic acid, *m*CPBA (77 % w/w, 4.02 g, 17.94 mmol) was added portion-wise during 15 minutes, and the suspension was stirred for further 18 hours, allowing to warm up from 0 °C to room temperature. Dichloromethane (60 mL) was added to the suspension and the solution was sequentially washed with saturated $Na_2S_2O_3$ (3 × 70 mL), saturated $NaHCO_3$ (3 × 70 mL), and deionized water (3 × 70 mL). The recovered organic phase was dried over Na_2SO_4 and the solvent removed by rotary evaporation under reduced pressure. The product was purified by column chromatography on silica gel using an initial solvent mixture of petroleum ether/EtOAc = 9/1, followed by petroleum ether/EtOAc = 8/2, yielding the desired EGH (2.69 g; yield = 70.4%).

NMR Spectroscopic data of EGH

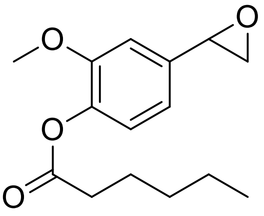

^{1}H NMR (400 MHz, $CDCl_3$, rt): δ 6.99 (*d*, 1H, *J* = 8.1 Hz, -Ar*H*); δ 6.89 (*dd*, 1H, *J* = 8.1 Hz, *J* = 1.5 Hz, -Ar*H*); 6.84 (*d*, 1H, J = 1.5 Hz, -Ar*H*); 3.85 (*dd*, 1H, *J* = 4.1 Hz, *J* = 2.3 Hz, -C*HO*CH_2, epoxide); 3.81 (*s*, 3H, -OCH_3); 3.13 (*dd*, 1H, *J* = 5.5 Hz, *J* = 4.1 Hz *cis*-CHOCH_2, epoxide); 2.75 (*dd*, 1H, *J* = 5.5 Hz, *J* = 2.3 Hz *trans*-CHOCH_2, epoxide); 2.56 (*t*, 2H, -OCOC$H_2CH_2CH_2CH_2CH_3$); 1.76 (quint, 2H, -OCOCH_2C$H_2CH_2CH_2CH_3$); 1.44-1.34 (m, overlapped, 4H, -OCOCH_2CH_2CH_2CH_2CH_3); 0.93 (*t*, 2H, -OCO$CH_2CH_2CH_2CH_2$CH_3). ^{13}C NMR (151 MHz, $CDCl_3$, rt): δ 172.02; 151.56; 139.84; 136.63; 122.96; 118.18; 109.01; 56.00; 52.30; 51.39; 34.12; 31.34; 24.83; 22.45; 14.07.

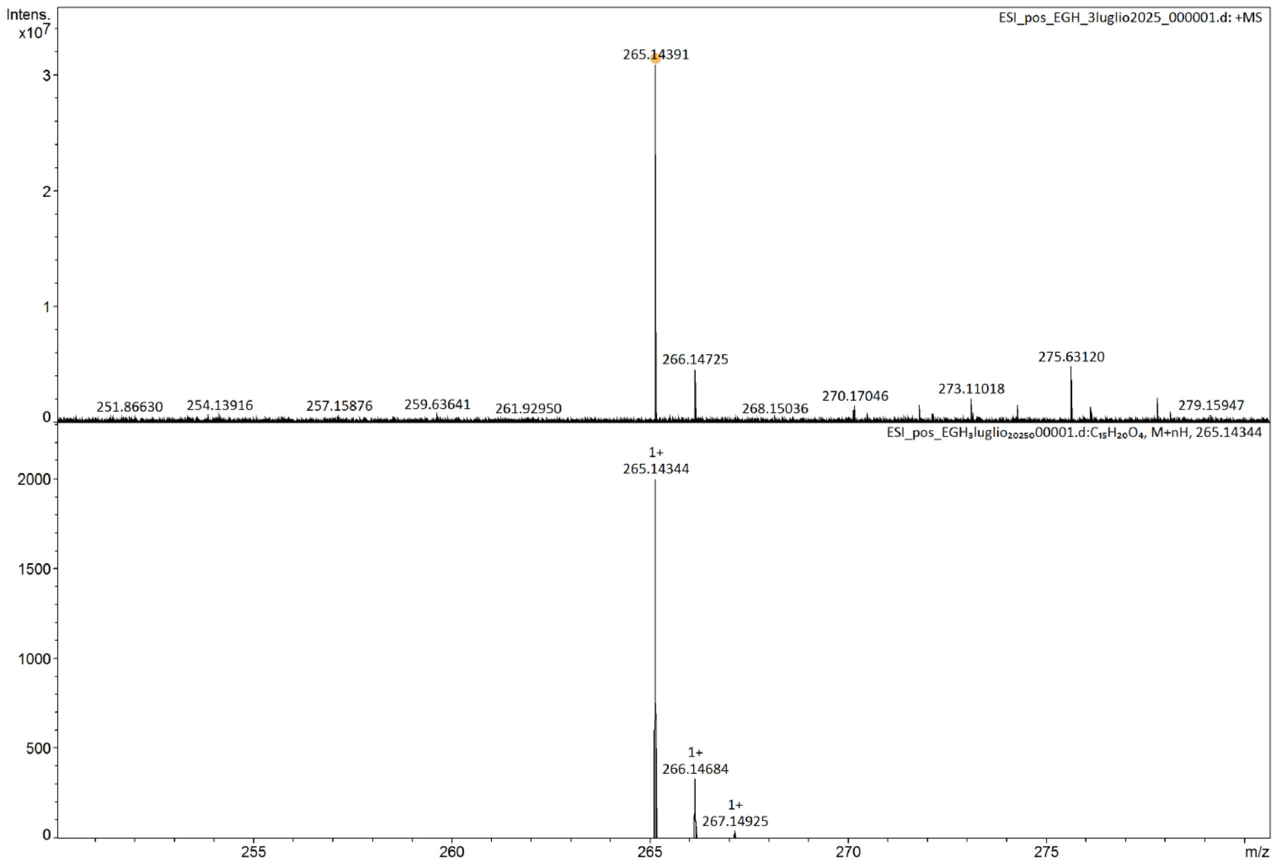

FigureS 32. High Resolution ESI MS measured (top) and calculated (bottom) spectra of 4-epoxy guaiacol hexanoate in MeOH, region from 250 to 280 m/z. $[C_{11}H_{13}O_4]^+$ calcd. = 265.14344 uma; measured = 265.14391 uma.

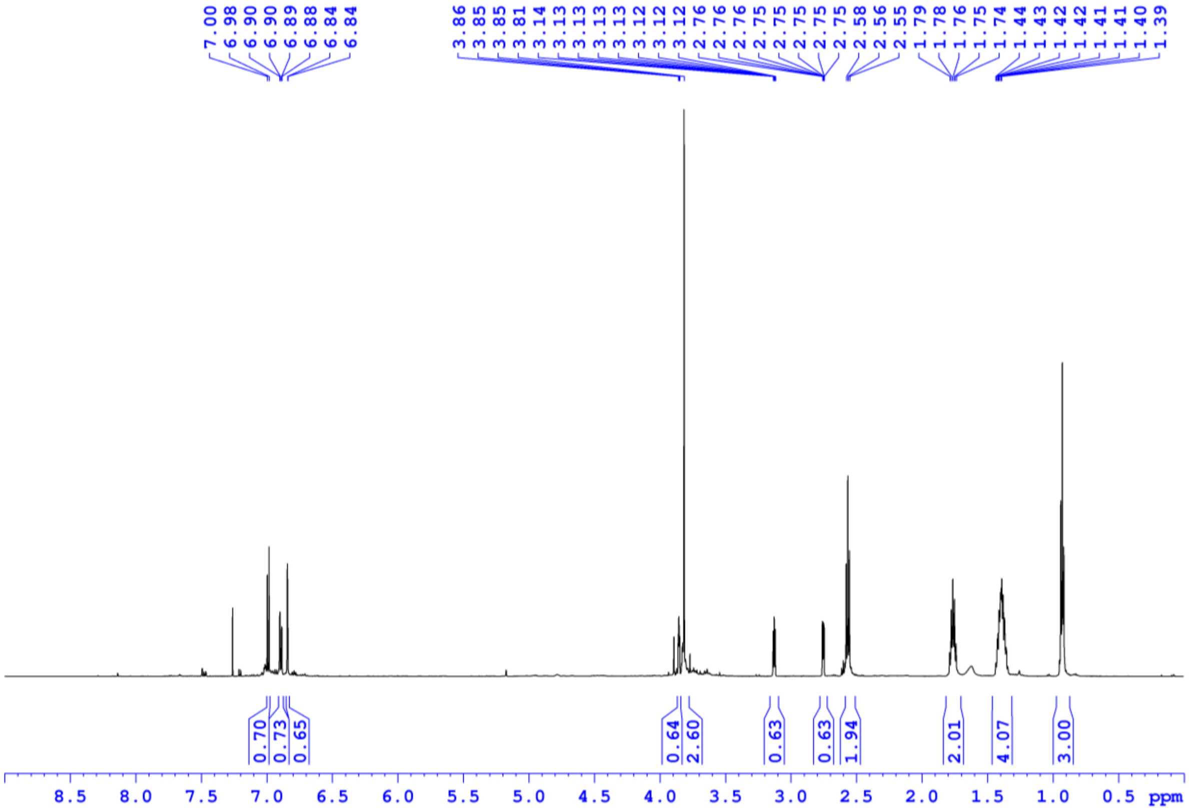

FigureS 33. ^{1}H NMR of 4-epoxy guaiacol hexanoate (400 MHz, rt, $CDCl_3$).

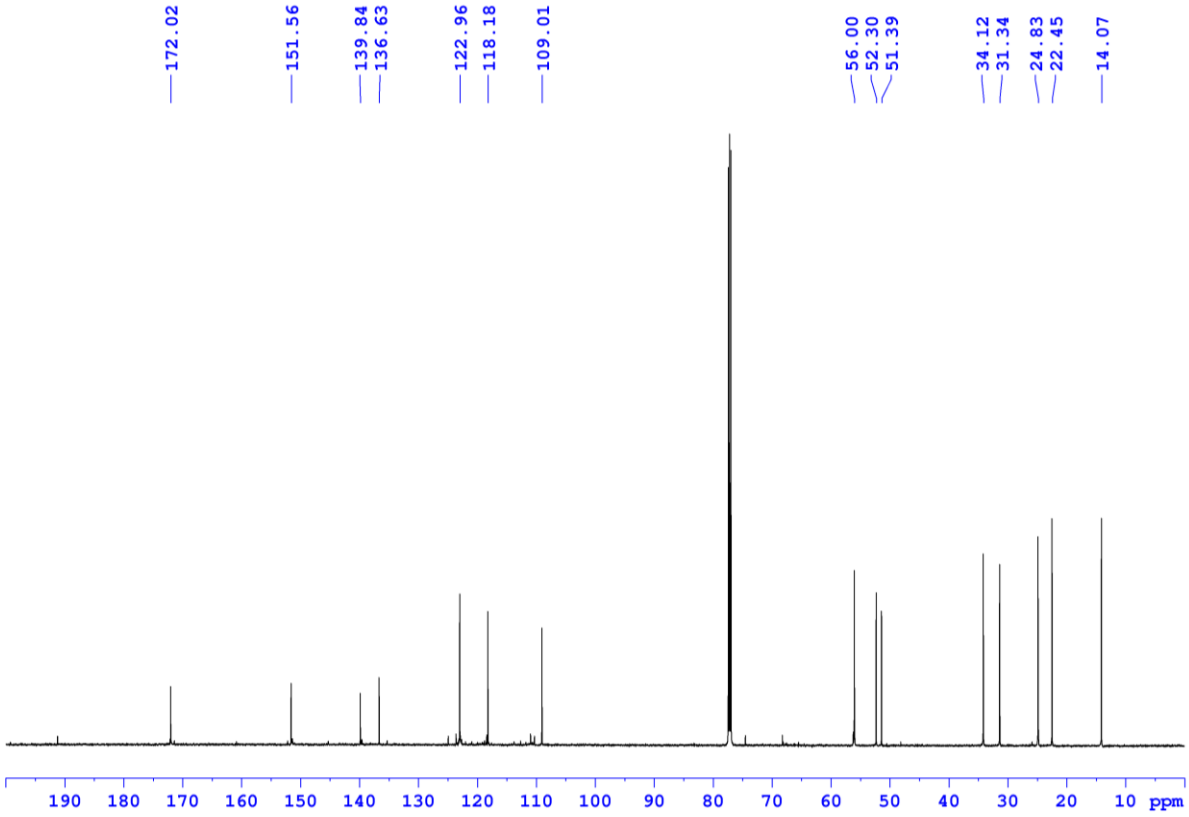

FigureS 34. ^{13}C NMR of 4-epoxy guaiacol hexanoate (151 MHz, rt, $CDCl_3$).

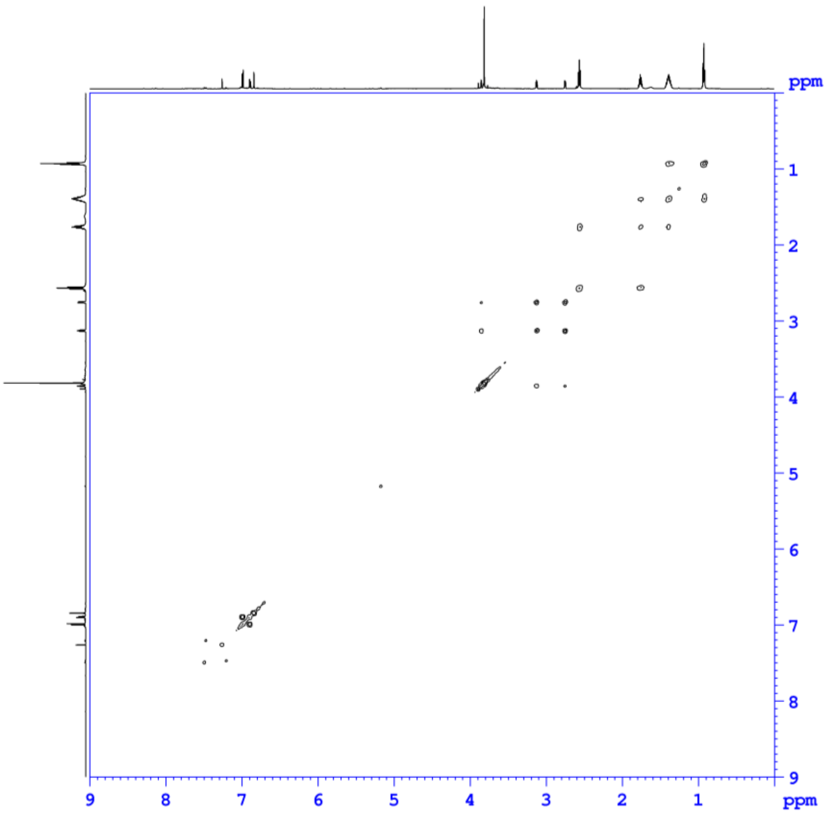

FigureS 35. ^{1}H-^{1}H COSY NMR of 4-epoxy guaiacol hexanoate (600 MHz, rt, $CDCl_3$).

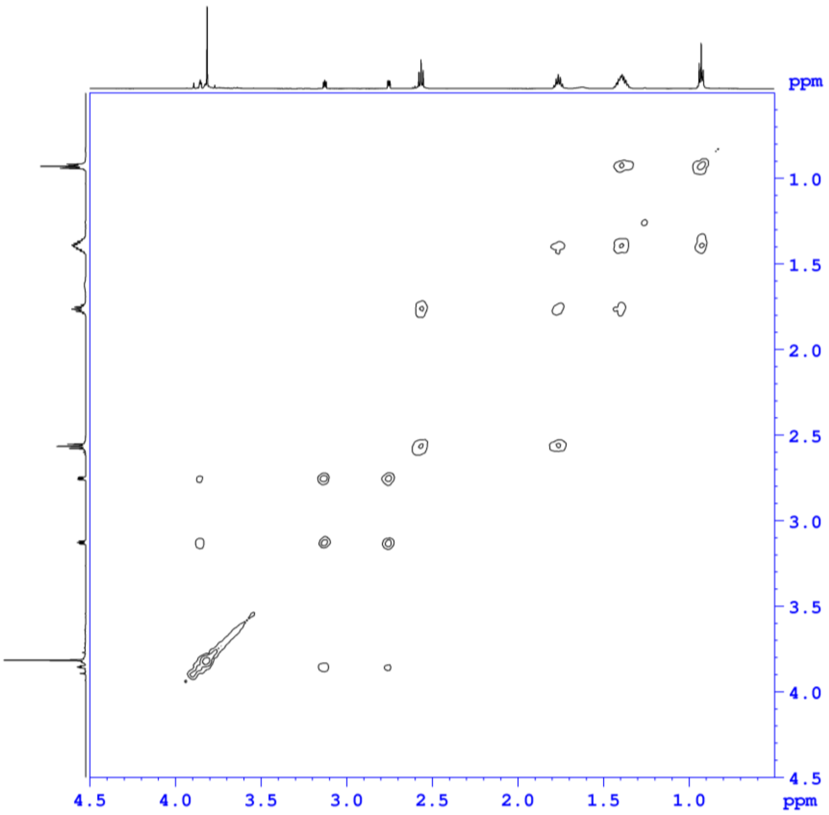

FigureS 36. ^{1}H-^{1}H COSY NMR of 4-epoxy guaiacol hexanoate from 4.5 to 0.5 ppm (600 MHz, rt, $CDCl_3$).

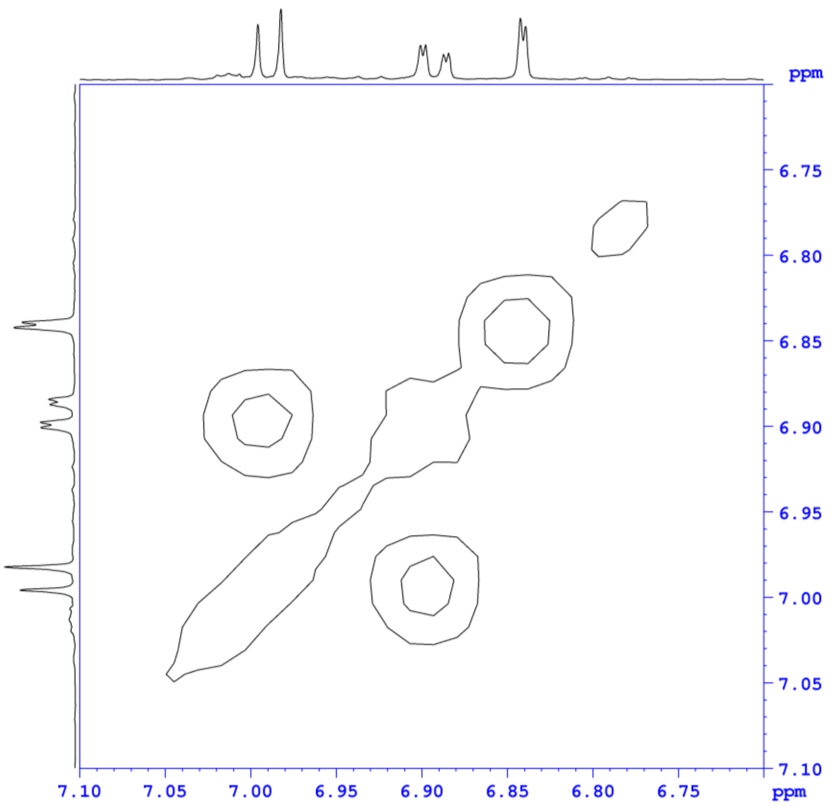

FigureS 37. ^{1}H-^{1}H COSY NMR of 4-epoxy guaiacol hexanoate from 7.1 to 6.7 ppm (600 MHz, rt, $CDCl_3$).

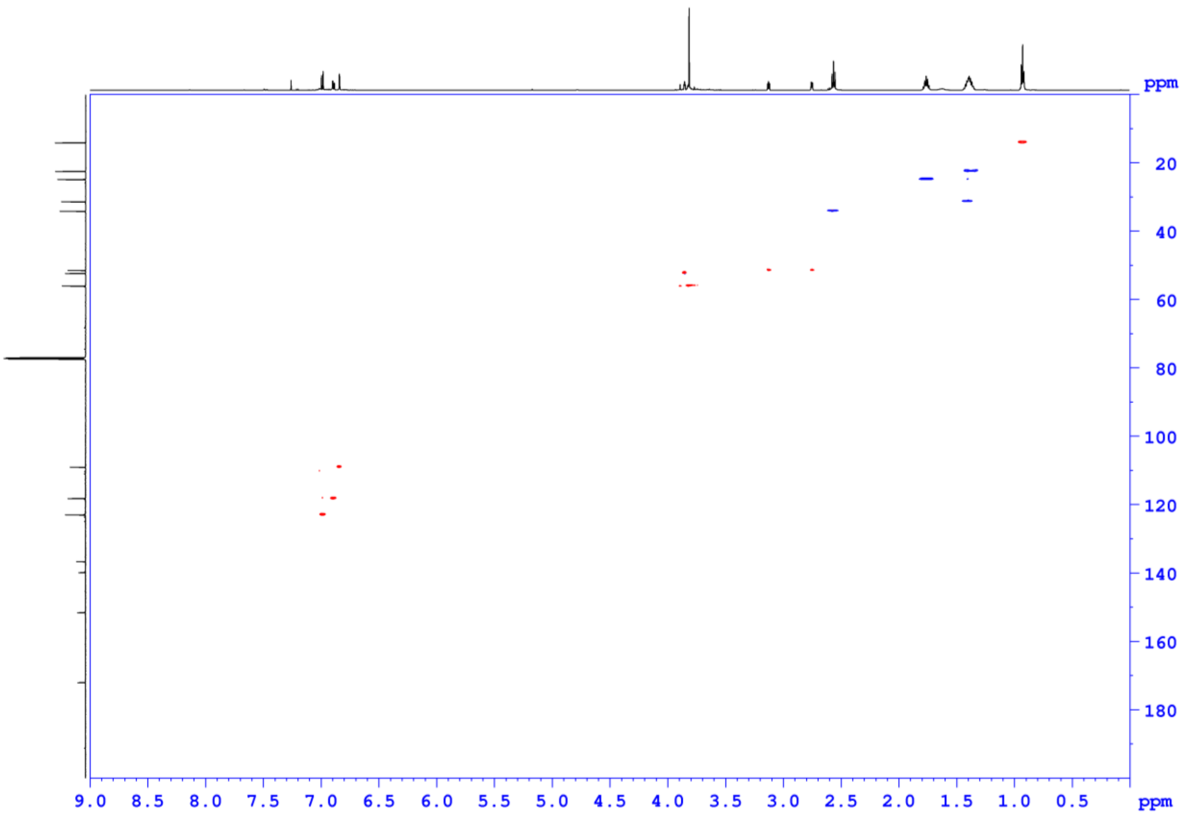

FigureS 38. ^{1}H-^{13}C HSQC NMR of 4-epoxy guaiacol hexanoate (600 MHz, rt, $CDCl_3$).

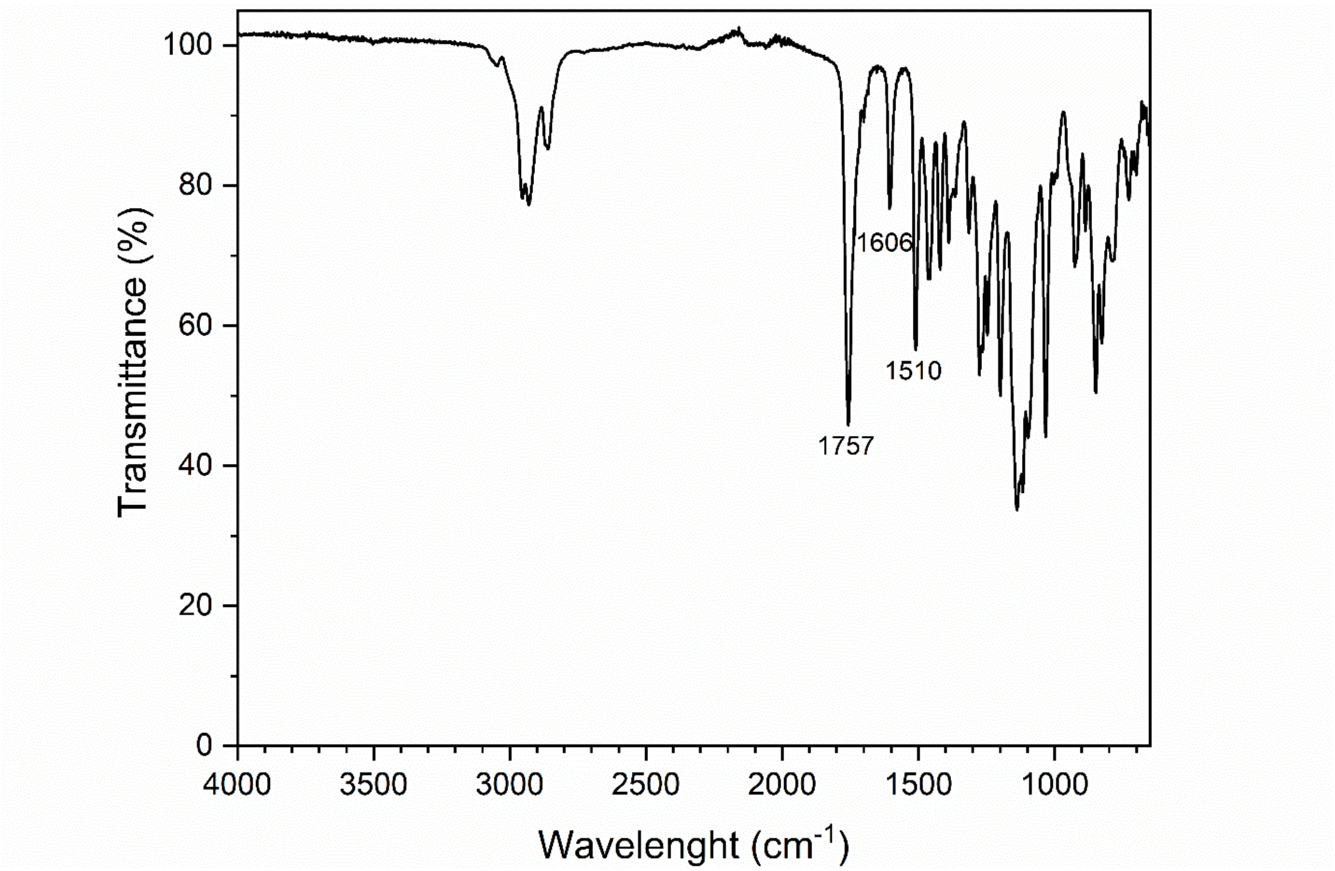

FigureS 39. ATR-FTIR of 4-epoxy guaiacol hexanoate.

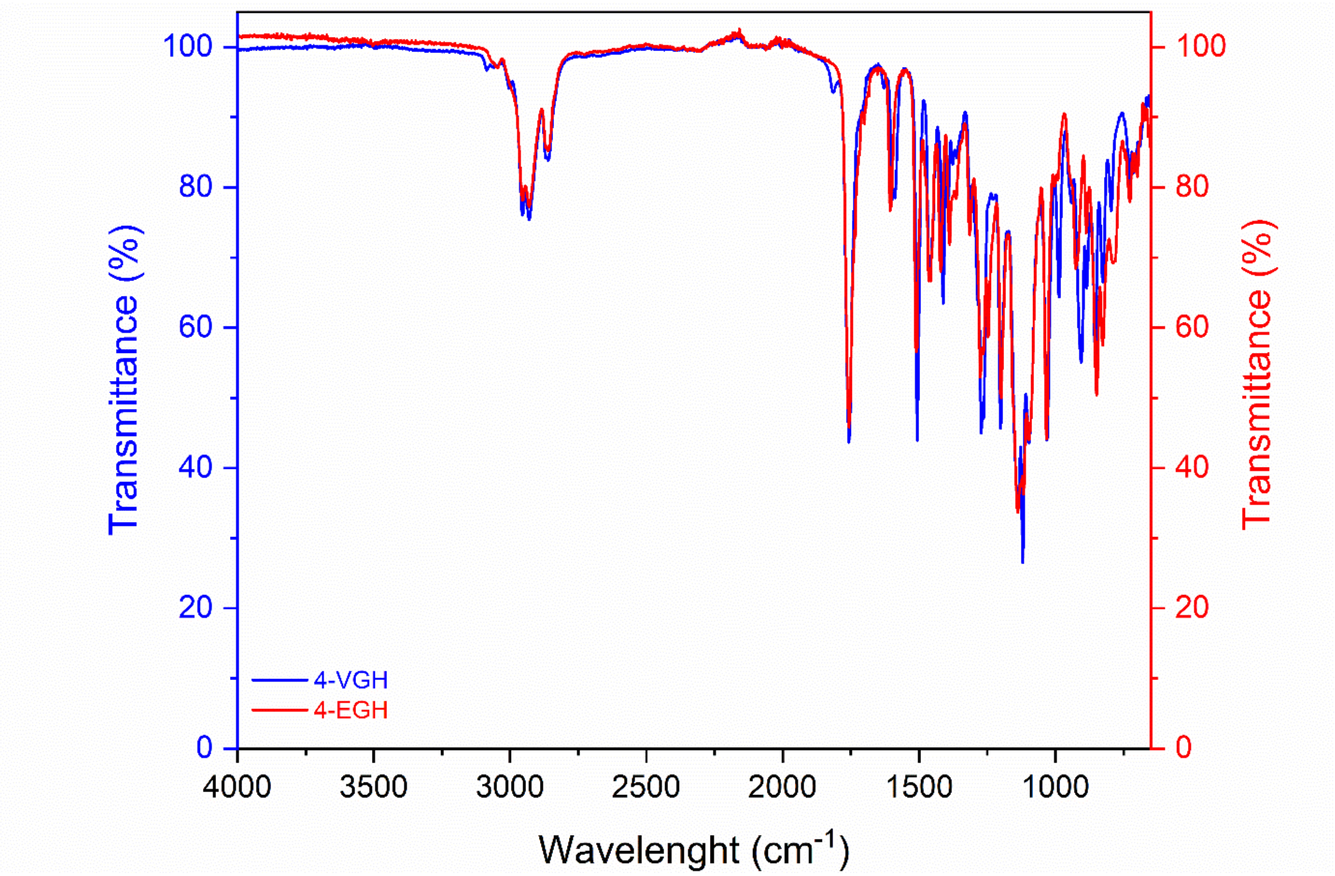

FigureS 40. Comparison of the ATR-FTIR spectra of 4-vinyl guaiacol hexanoate (blue curve) and of 4-epoxy guaiacol hexanoate (red curve).

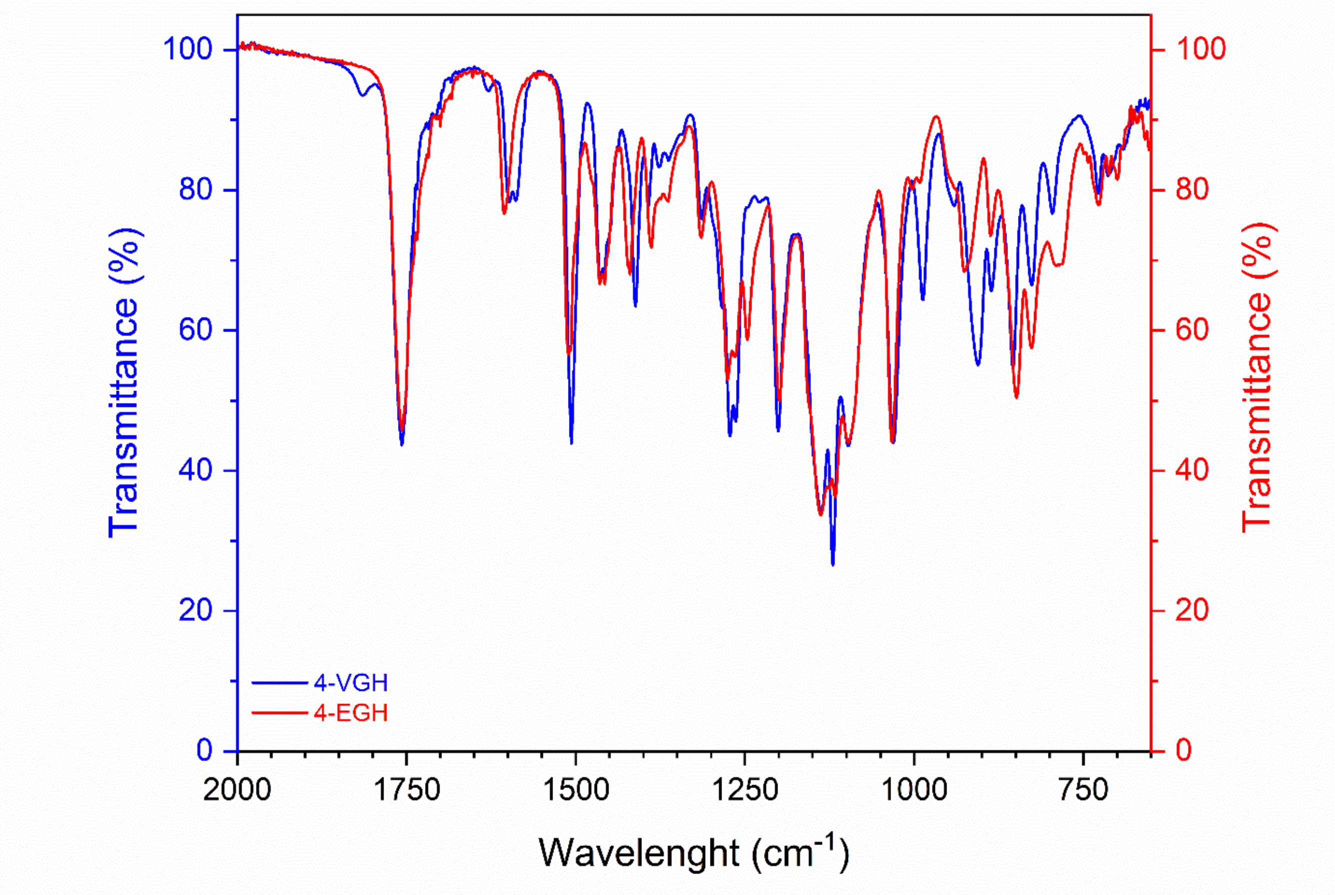

FigureS 41. Comparison of the ATR-FTIR spectra of 4-vinyl guaiacol hexanoate (blue curve) and of 4-epoxy guaiacol hexanoate (red curve). Region from 2000 to 650 cm^{-1}.

5. Ring-Opening Copolymerization Procedures

5.1 Catalyst screening for EGA/PA ROCOP without solvent

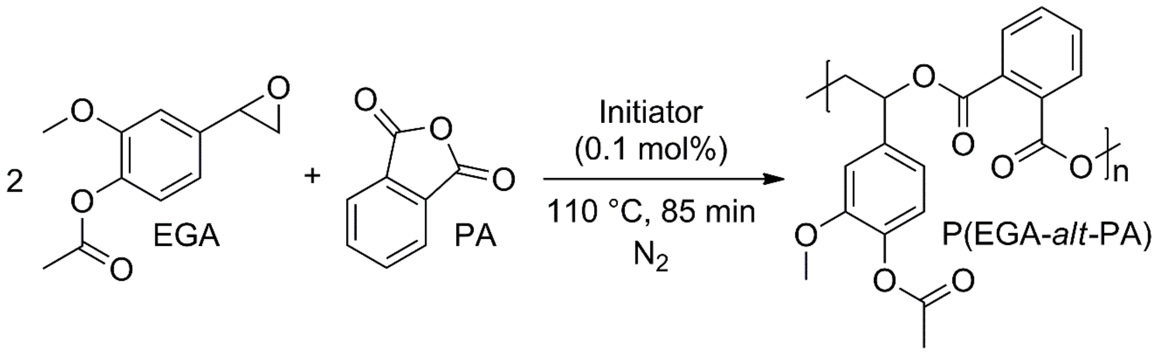

All polymerizations of EGA with PA performed without solvent to test different initiators were conducted according to the following procedure, which refers to entry 1 of Table S1.

The CsOAc (1.3 mg, 6.75 µmol), PA (100 mg, 0.68 mmol), and EGA (281 mg, 1.35 mmol) were added in this order to a 10 mL Schlenk tube equipped with a magnetic stirring bar under nitrogen. The tube was evacuated and filled with nitrogen three times, closed and placed into a heating oil bath at 110 °C. The reaction mixture was kept stirring for the 85 minutes. To stop the reaction, the tube was cooled down to room temperature and opened to air. An aliquot was collected and dissolved in $CDCl_3$ to determine the conversion by ^1H NMR analysis (Figure S42), then the reaction mixture was poured into methanol under stirring and the polymer was collected by filtration. The polymer was purified by dissolution into dichloromethane and precipitated from methanol. Conversion (NMR) = 85.9 %; M_n = 4.2 kDa; $Đ$ = 1.22.

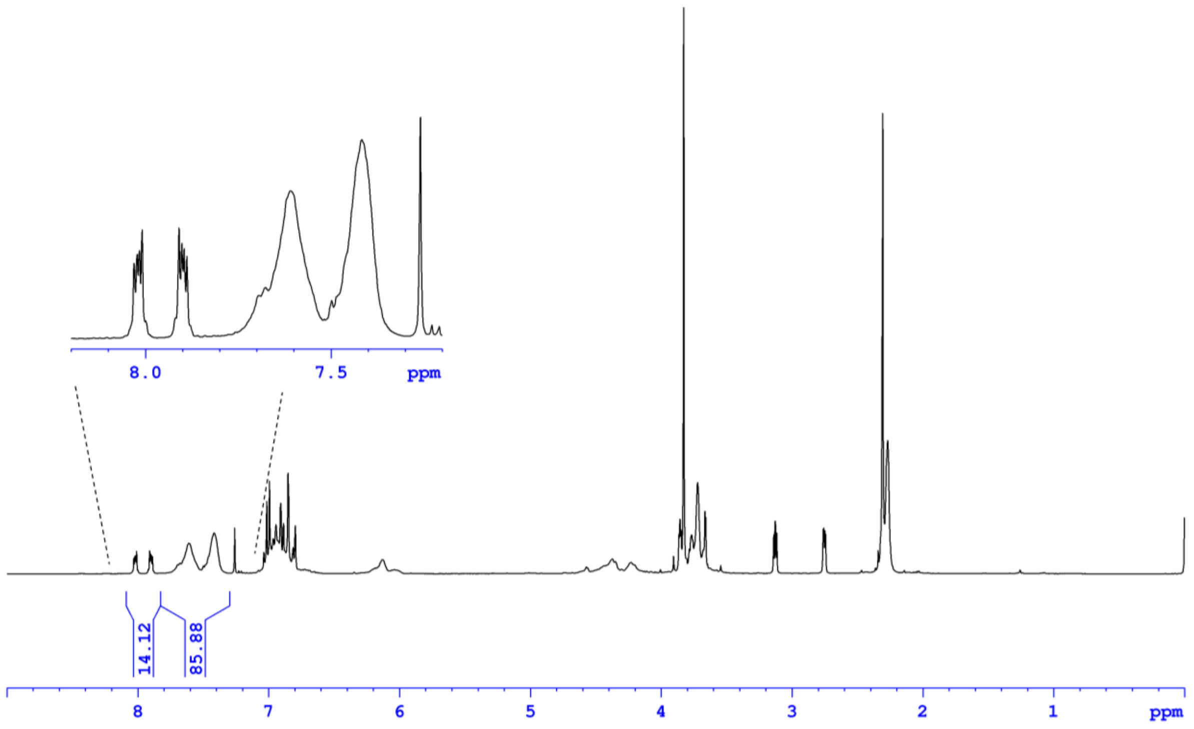

FigureS 42. ^1H NMR spectra of an aliquot of the reaction mixture for the formation of P(EGA-*alt*-PA) for conversion determination (entry 1, Table S1) (400 MHz, rt, $CDCl_3$,).

5.2 Catalyst screening for EGA/PA ROCOP with different solvents

All polymerizations of EGA with PA performed to test different solvents and catalysts were conducted according to the following procedure, which refers to entry 12 of Table S1.

The PPNCl (3.9 mg, 6.75 µmol), PA (100 mg, 0.68 mmol), and EGA (141 mg, 0.68 mmol) were added in this order to a 10 mL Schlenk tube equipped with a magnetic stirring bar under nitrogen. The tube was evacuated and filled with nitrogen three times. Toluene (0.2 mL) was added using a glass syringe, then the tube was closed and placed into a heating oil bath at 110 °C. The reaction mixture was kept stirring for the 205 minutes. To stop the reaction, the tube was cooled down to room temperature and opened to air. An aliquot was collected and dissolved in $CDCl_3$ to determine the conversion by ^{1}H NMR analysis (Figure S43), then the reaction mixture was poured into methanol under stirring and the polymer was collected by filtration. The polymer was purified by dissolution into dichloromethane and precipitated from methanol. Conversion (NMR) = 94.2 %; M_n = 4.1 kDa; $Đ$ = 1.17.

NMR Spectroscopic data of P(EGA-*alt*-PA)

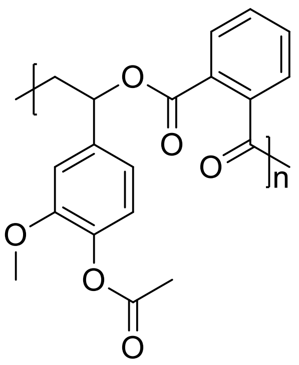

^{1}H NMR (400 MHz, $CDCl_3$, rt): δ 7.64 (*s* broad, 2H, Ar-H phthalic anhydride moiety); 7.42 (*s* broad, 2H, Ar-H phthalic anhydride moiety); 7.42 (*s* broad, 2H, Ar-H guaiacol moiety); 7.0-6.7 (*m*, overlapped, 3H, Ar-H guaiacol moiety); 6.3-6.0 (*m*, 1H, C*H*, benzylic); 4.6-4.1 (*m*, overlapped, 2H, C*H₂*); 3.77-3.68 (*s*, 3H, -OC*H₃*); 2.27 (*s*, 3H, -COC*H₃*).

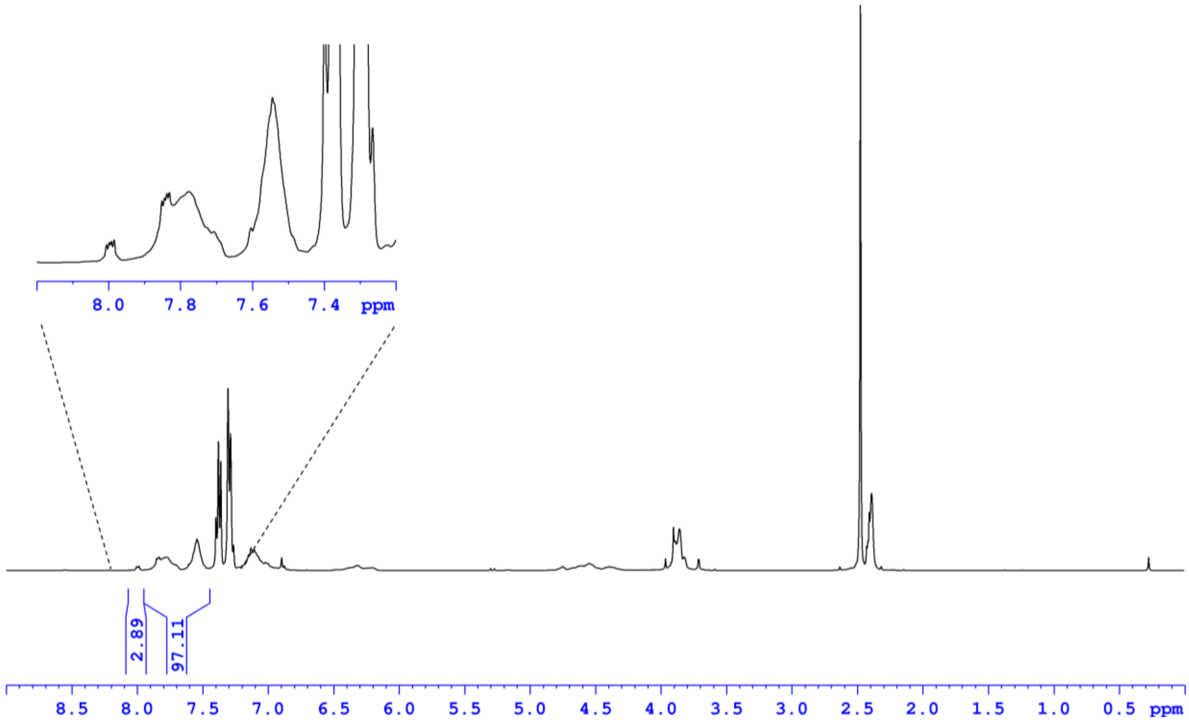

FigureS 43. ^{1}H NMR spectra of an aliquot of the reaction mixture for the formation of P(EGA-*alt*-PA) for conversion determination (entry 12, Table S1) (400 MHz, rt, $CDCl_3$,).

TableS 1. Screening of catalysts and solvents for the ROCOP of EGA with PA.[a)]

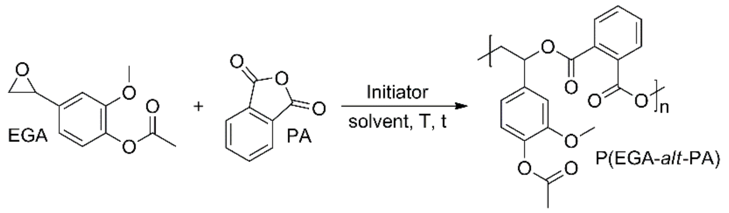

Entry	Initiator	Solvent	Time [h]	Conversion [b)] [%]	M_{calcd} [kDa]	M_n [c)] [kDa]	$Đ$ [c)]
1 [d)]	CsOAc	No	1.4	86	30.6	4.2	1.20
2 [d)]	DMAP	No	1.4	91	32.4	5.1	1.17
3 [d)]	TBAB	No	1.4	92	32.8	4.5	1.30
4 [d)]	PPNCl	No	1.4	>99	35.6	3.2	1.22
5	CsOAc	Toluene	1.4	32	11.4	n.d. [e)]	n.d. [e)]
6	DMAP	Toluene	1.4	44	15.7	2.7	1.15
7	TBAB	Toluene	1.4	30	10.7	2.5	1.16
8	PPNCl	Toluene	1.4	66	23.5	2.9	1.14
9	CsOAc	Toluene	3.4	53	18.8	2.4	1.12
10	DMAP	Toluene	3.4	>99	35.6	2.5	1.21
11	TBAB	Toluene	3.4	94	33.6	2.3	1.21
12	PPNCl	Toluene	3.4	94	31.1	4.1	1.17
13	CsOAc	Anisole	1.4	38	13.4	2.0	1.06
14	DMAP	Anisole	1.4	44	15.8	1.9	1.13
15	TBAB	Anisole	1.4	74	26.2	2.2	1.20
16	PPNCl	Anisole	1.4	87	31.1	2.1	1.20
17 [f)]	CsOAc	THF	23.0	58	20.6	1.9	1.05
18 [f)]	DMAP	THF	23.0	70	24.9	2.8	1.20
19 [f)]	TBAB	THF	23.0	84	29.9	2.9	1.17
20 [f)]	PPNCl	THF	23.0	83	29.7	2.0	1.14
21 [g)]	PPNCl	Toluene	3.4	75	107.2	3.1	1.16
22	No	Toluene	3.4	25	8.9	n.d. [e)]	n.d [e)]
23 [h)]	PPNCl	Toluene	3.4	78	111.2	3.3	1.14

[a)] Reaction conditions: [EGA]/[PA]/[Initiator] = 100/100/1, PA = 6.75 · 10^{-4} mol (100 mg), initiator = 6.75 · 10^{-6} mol, [PA] = 3.4 M, N_2 atmosphere, temperature = 110 °C; [b)] Determined by ^1H NMR; [c)] Determined by GPC in THF calibrated with polystyrene standards; [d)] [EGA]/[PA]/[Initiator] = 200/100/1; [e)] n.d. = not determined; [f)] Temperature = 80 °C; [g)] [EGA]/[PA]/[Initiator] = 400/400/1; h) [EGA]/[PA]/[Initiator] = 400/400/1, EGA = 1.42 · 10^{-2} mol (2.9 g), PA = 1.42 · 10^{-2} mol (2.1 g); PPNCl = 3.54 · 10^{-5} mol (20.3 mg).

5.3 ROCOP of 4-Epoxyguaiacol Acylate with Cyclic Anhydrides

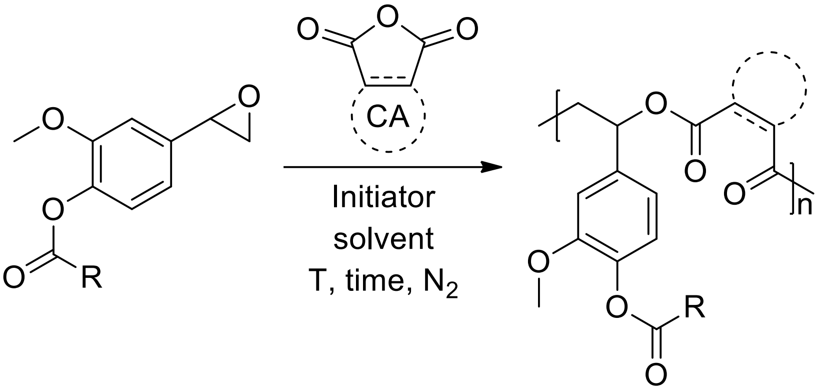

All polymerizations of EGA, EGB, and EGH with PA, THPA, MA and SA performed in toluene were conducted according to the following procedure, which refers to entry 1 of Table 1.

The PPNCl (20.3 mg, 35.4 μmol), PA (2.100 g, 14.2 mmol), and EGA (2.952 g, 14.2 mmol) were added in this order to a 25 mL Schlenk tube equipped with a magnetic stirring bar under nitrogen. The tube was evacuated and filled with nitrogen three times. Toluene (4.2 mL) was added using a glass syringe, then the tube was closed and placed into a heating oil bath at 110 °C. The reaction mixture was kept stirring for the 205 minutes. To stop the reaction, the tube was cooled down to room temperature and opened to air. An aliquot was collected and dissolved in $CDCl_3$ to determine the conversion by ^1H NMR analysis, then the reaction mixture was poured into methanol under stirring and the polymer was collected by filtration. The polymer was purified by dissolution into dichloromethane and precipitated from petroleum ether and dried in a vacuum oven at 30 °C for 24 hours. Conversion (NMR) = 78.0 %; gravimetric yield = 2.75 g (54.4 %); M_n = 3.3 kDa; $Đ$ = 1.14.

6. NMR Characterization of Polymers

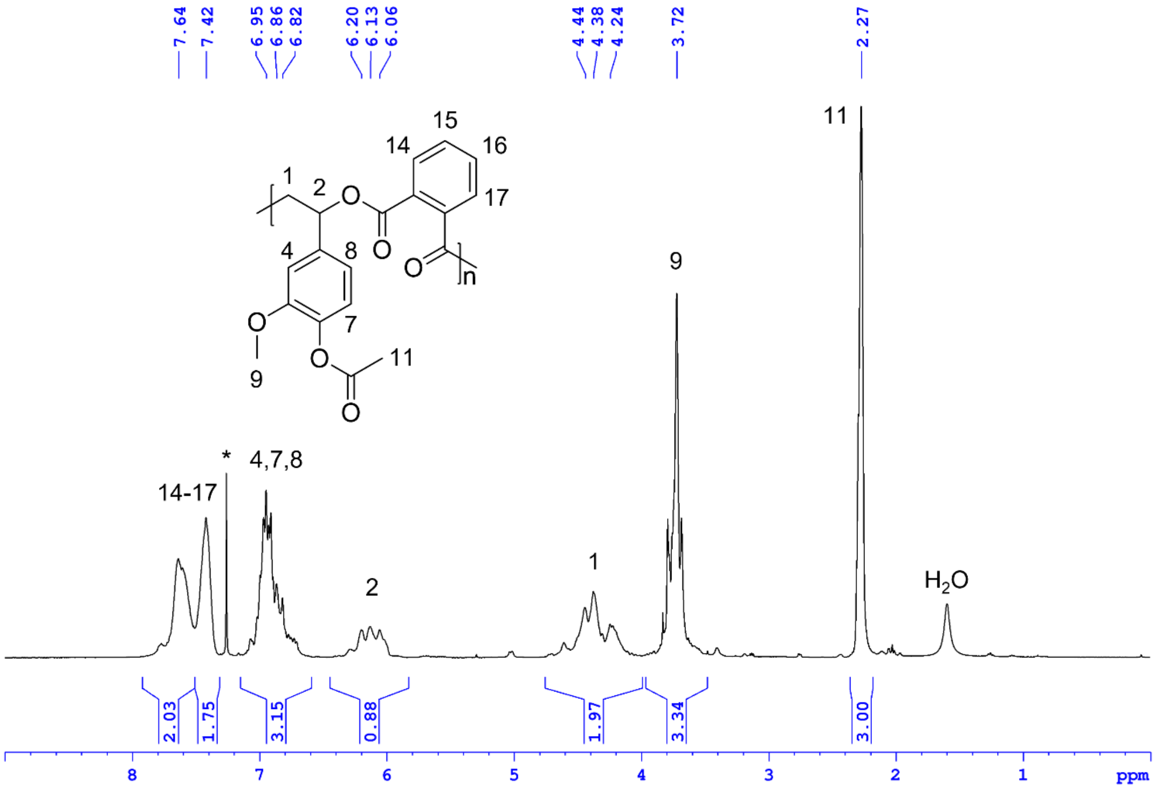

FigureS 44. ^{1}H NMR of P(EGA-*alt*-PA) (400 MHz, rt, $CDCl_3$).

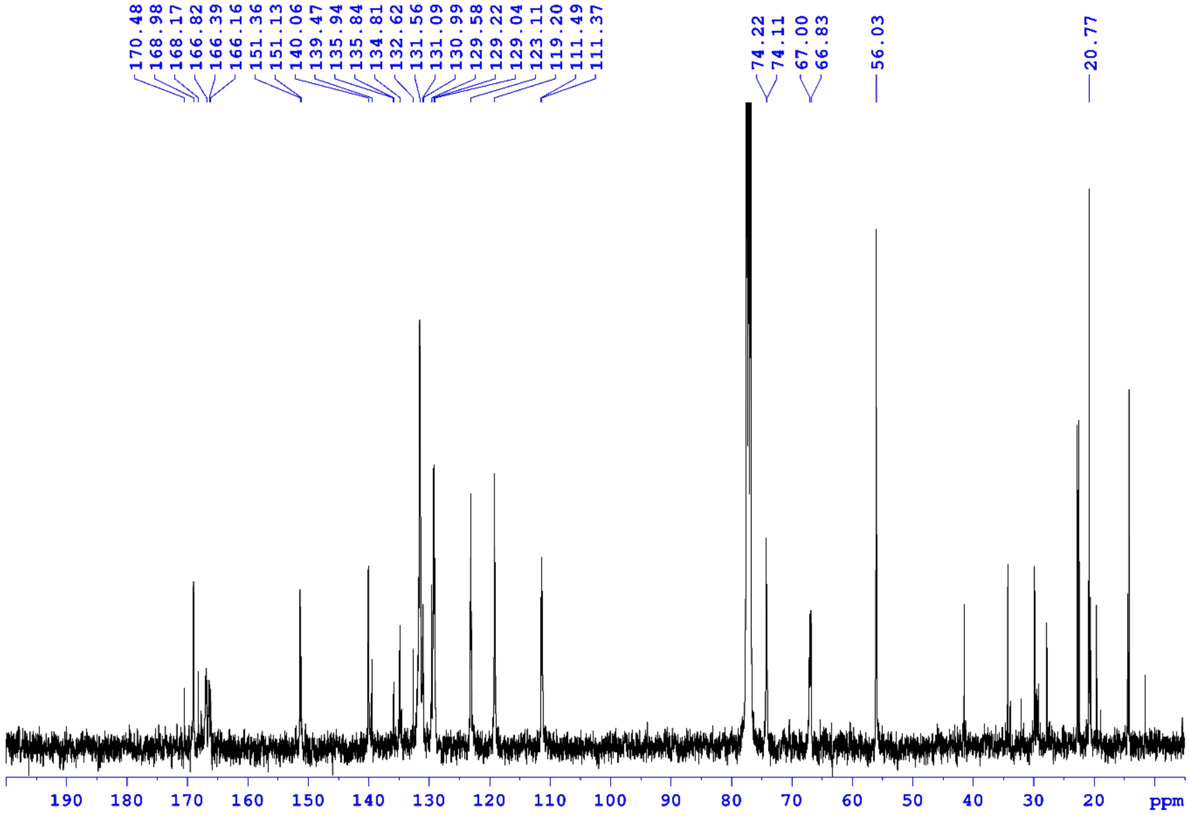

FigureS 45. ^{13}C NMR of P(EGA-*alt*-PA) (600 MHz, rt, $CDCl_3$).

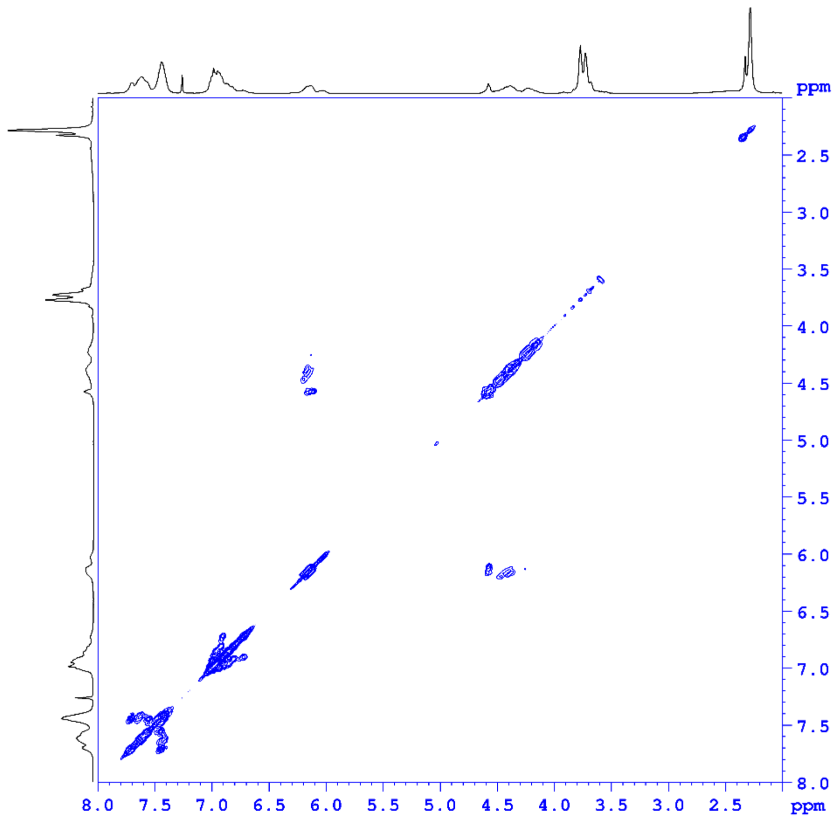

FigureS 46. ^{1}H-^{1}H COSY NMR of P(EGA-*alt*-PA) (600 MHz, rt, $CDCl_3$).

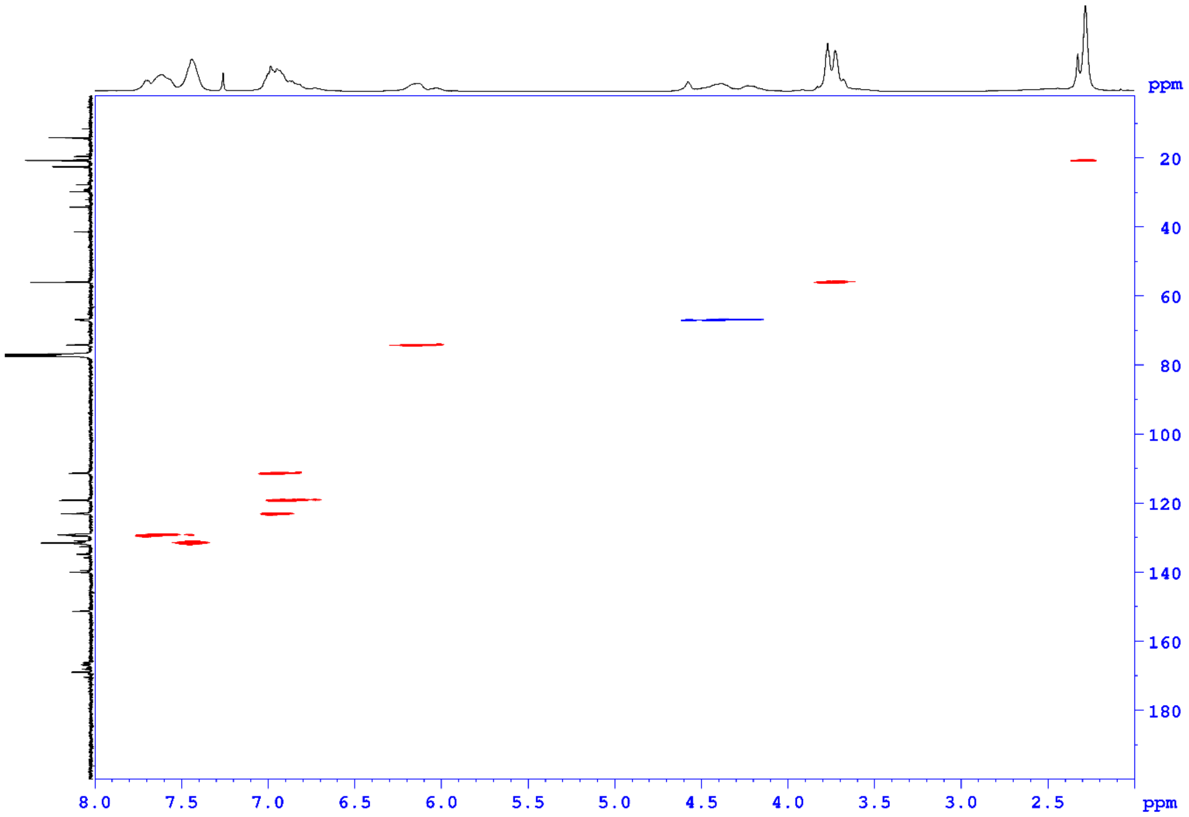

FigureS 47. ^{1}H-^{13}C HSQC NMR of P(EGA-*alt*-PA) (600 MHz, rt, $CDCl_3$).

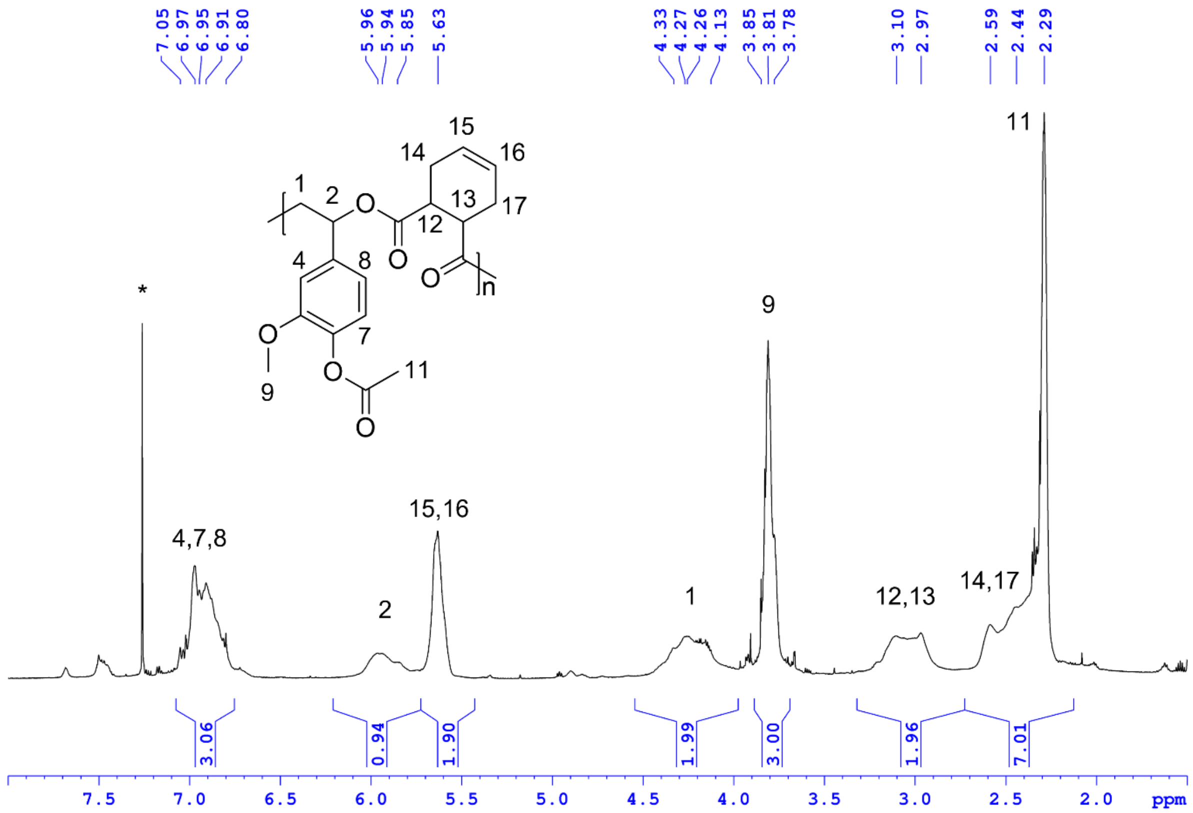

FigureS 48. ^{1}H NMR of P(EGA-*alt*-THPA) (600 MHz, rt, $CDCl_3$).

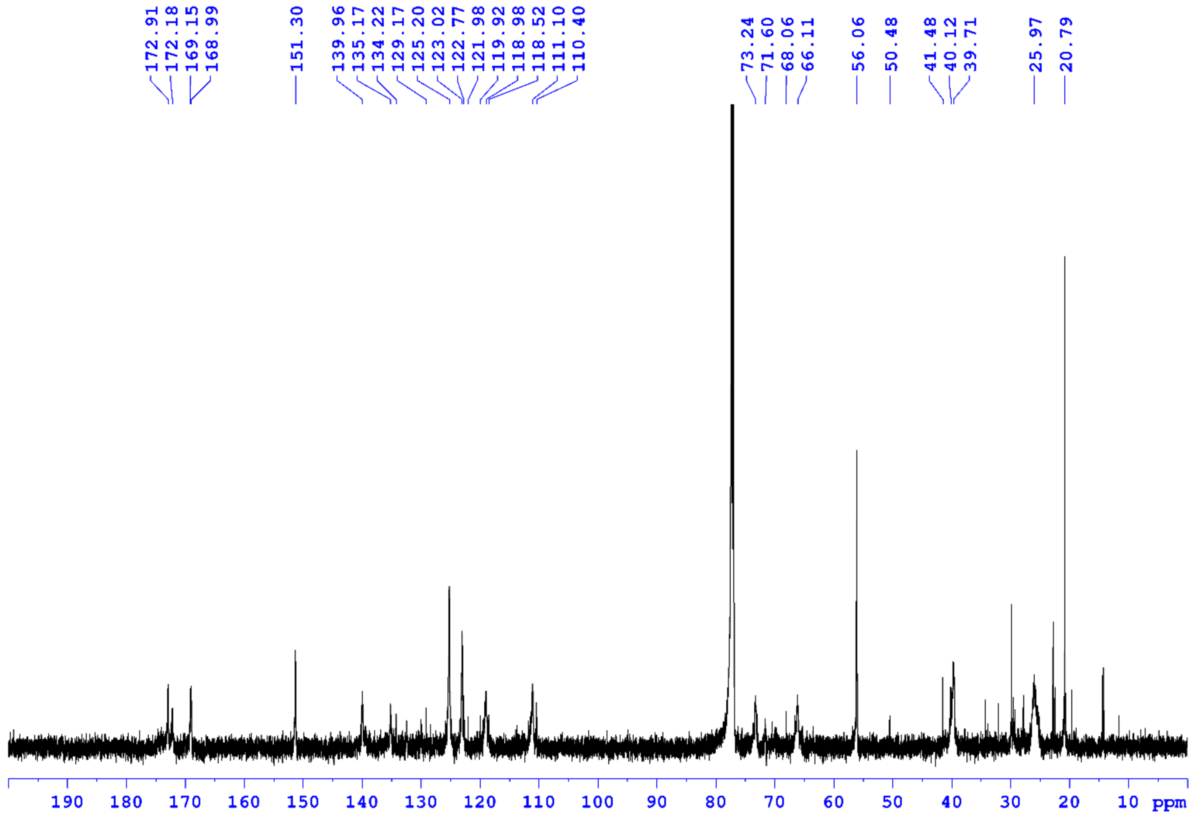

FigureS 49. ^{13}C NMR of P(EGA-*alt*-THPA) (600 MHz, rt, $CDCl_3$).

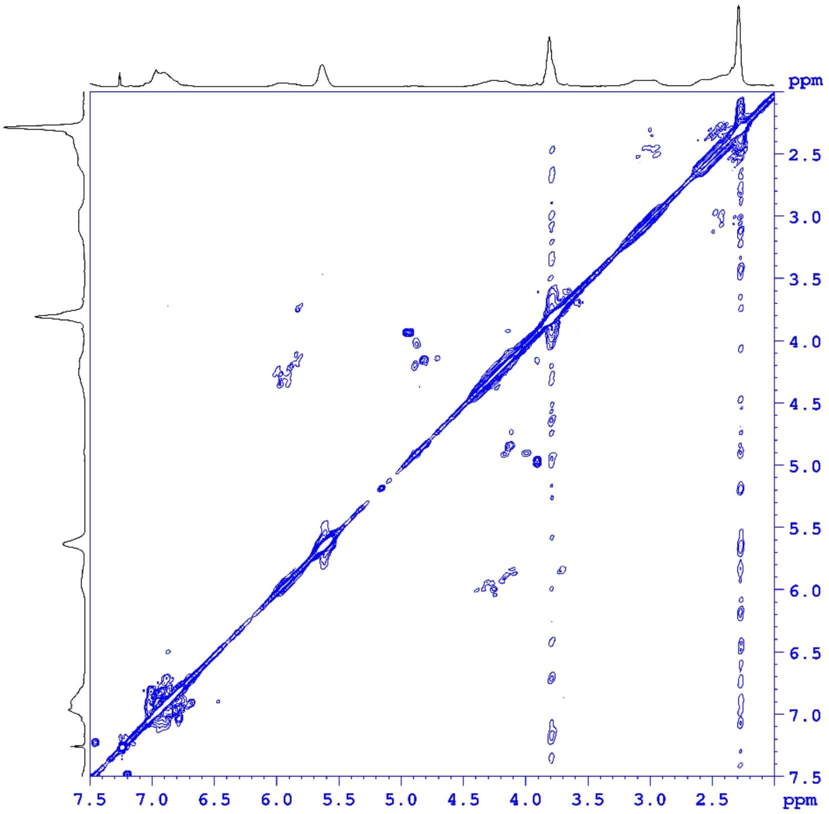

FigureS 50. ^1H-^1H COSY NMR of P(EGA-*alt*-THPA) (600 MHz, rt, $CDCl_3$).

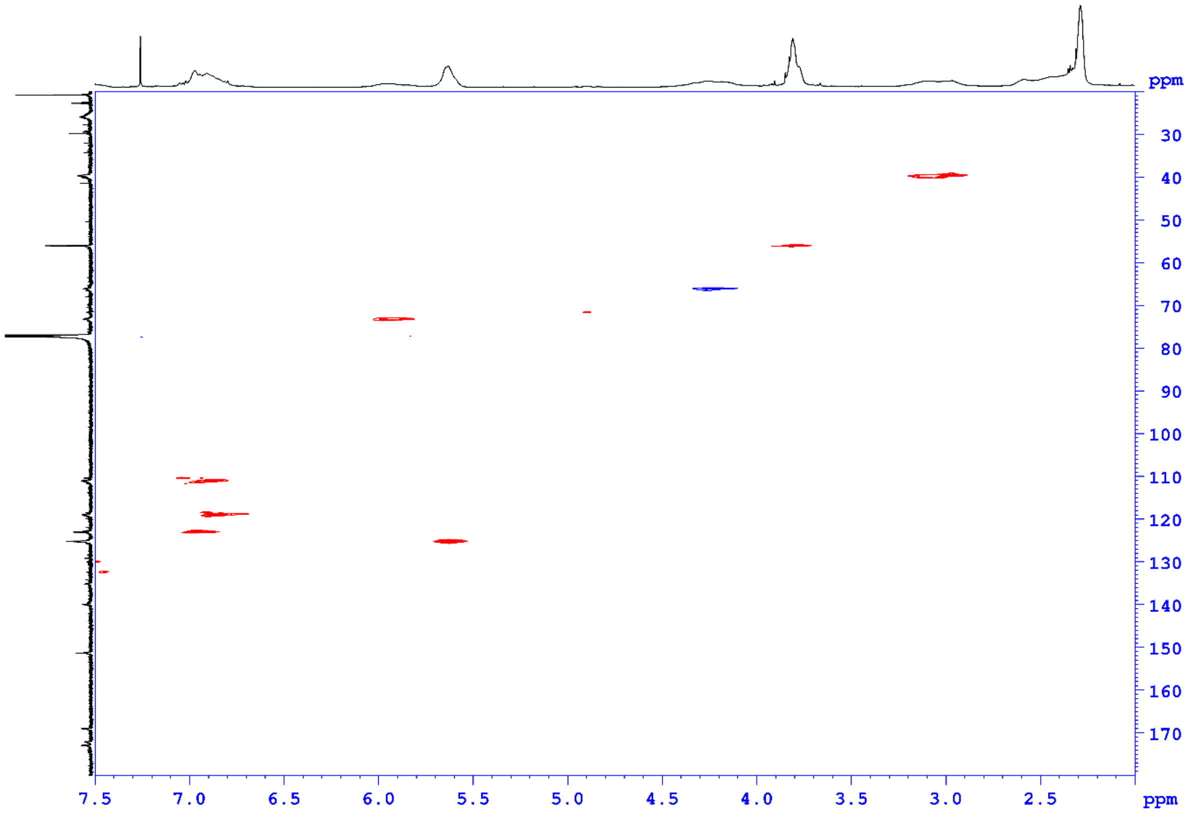

FigureS 51. ^1H-^{13}C HSQC NMR of P(EGA-*alt*-THPA) (600 MHz, rt, $CDCl_3$).

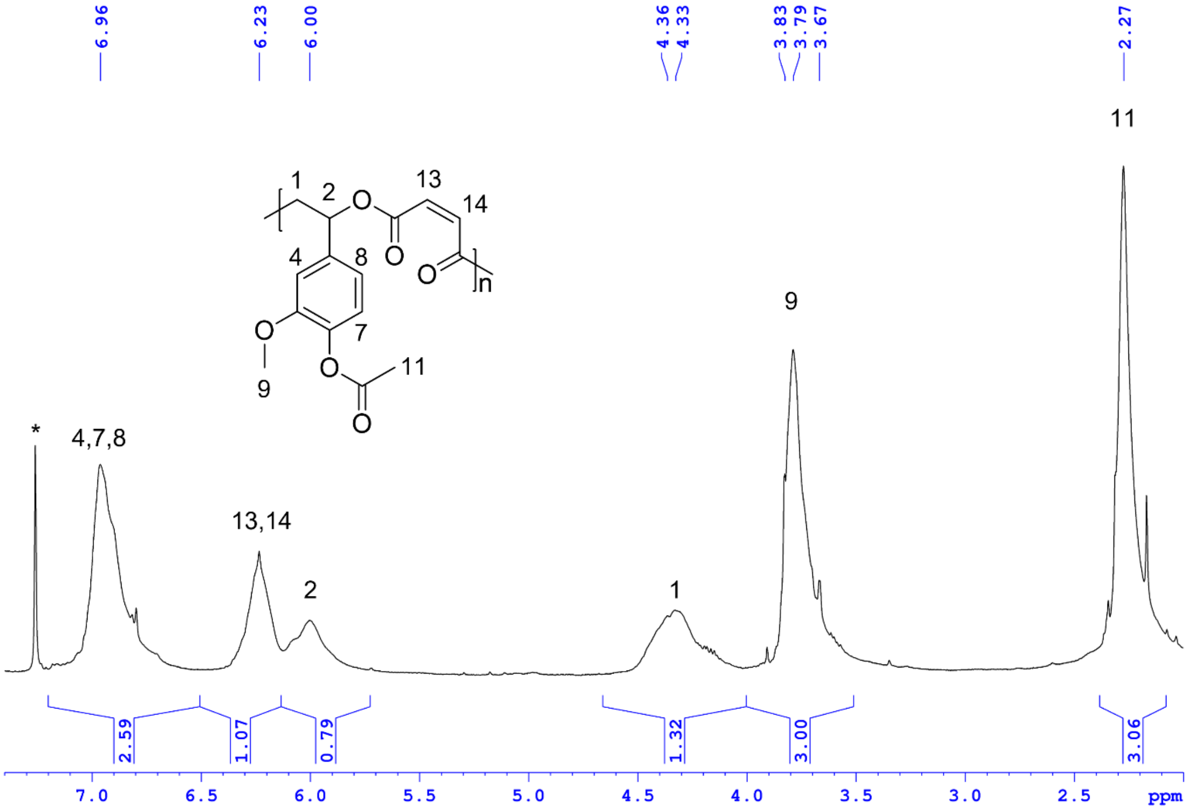

FigureS 52. ^{1}H NMR of P(EGA-*alt*-MA) (600 MHz, rt, $CDCl_3$).

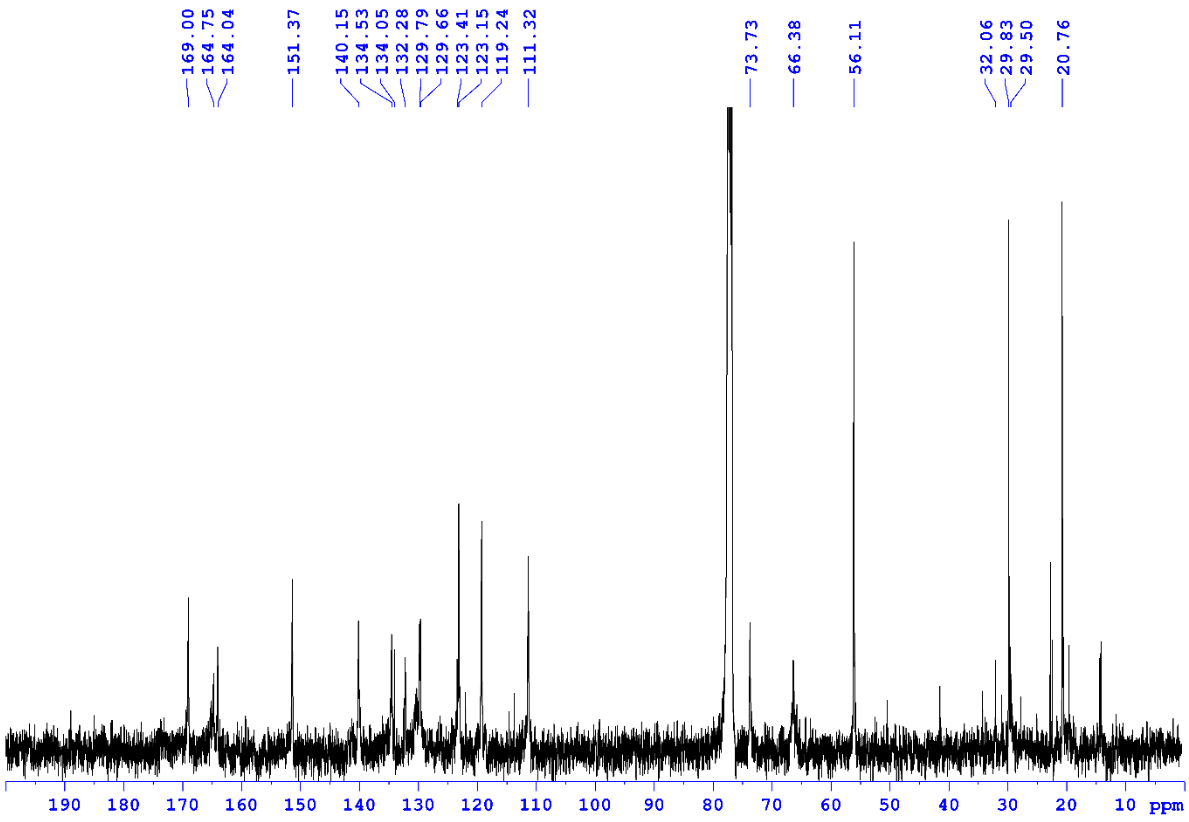

FigureS 53. ^{13}C NMR of P(EGA-*alt*-MA) (600 MHz, rt, $CDCl_3$).

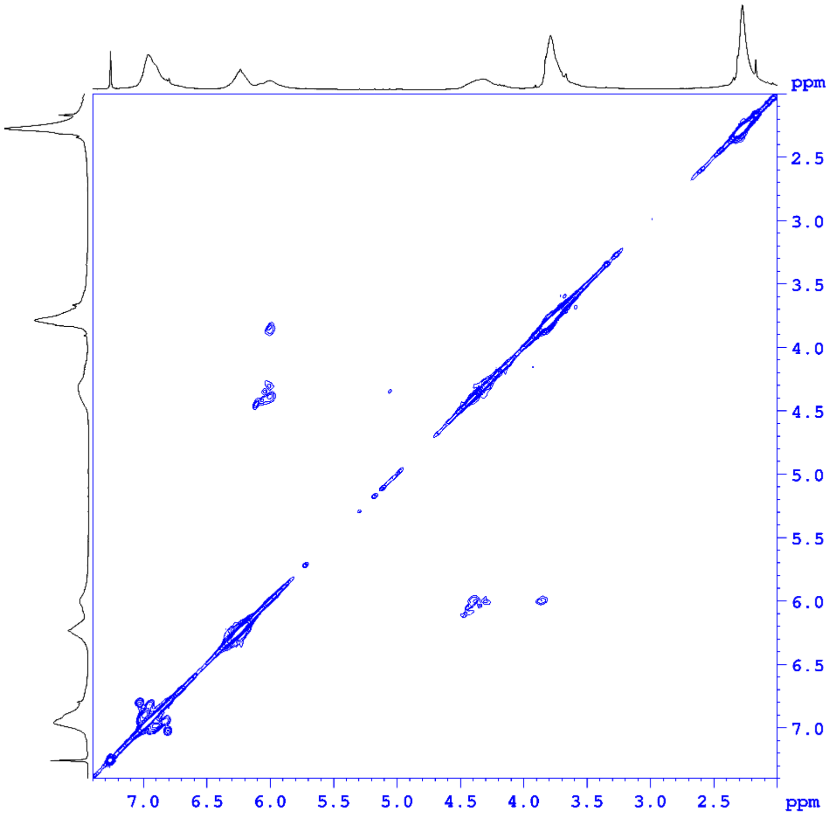

FigureS 54. ^{1}H-^{1}H COSY NMR of P(EGA-*alt*-MA) (600 MHz, rt, $CDCl_3$).

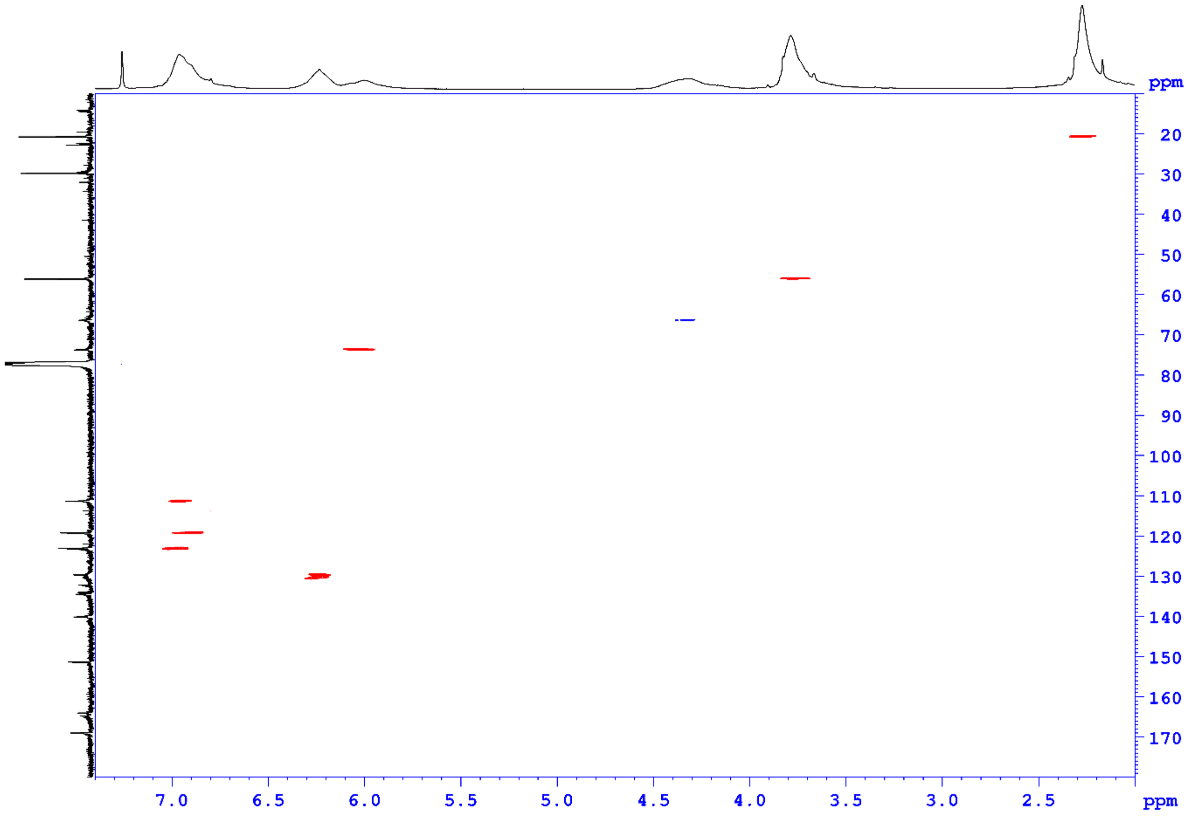

FigureS 55. ^{1}H-^{13}C HSQC NMR of P(EGA-*alt*-MA) (600 MHz, rt, $CDCl_3$).

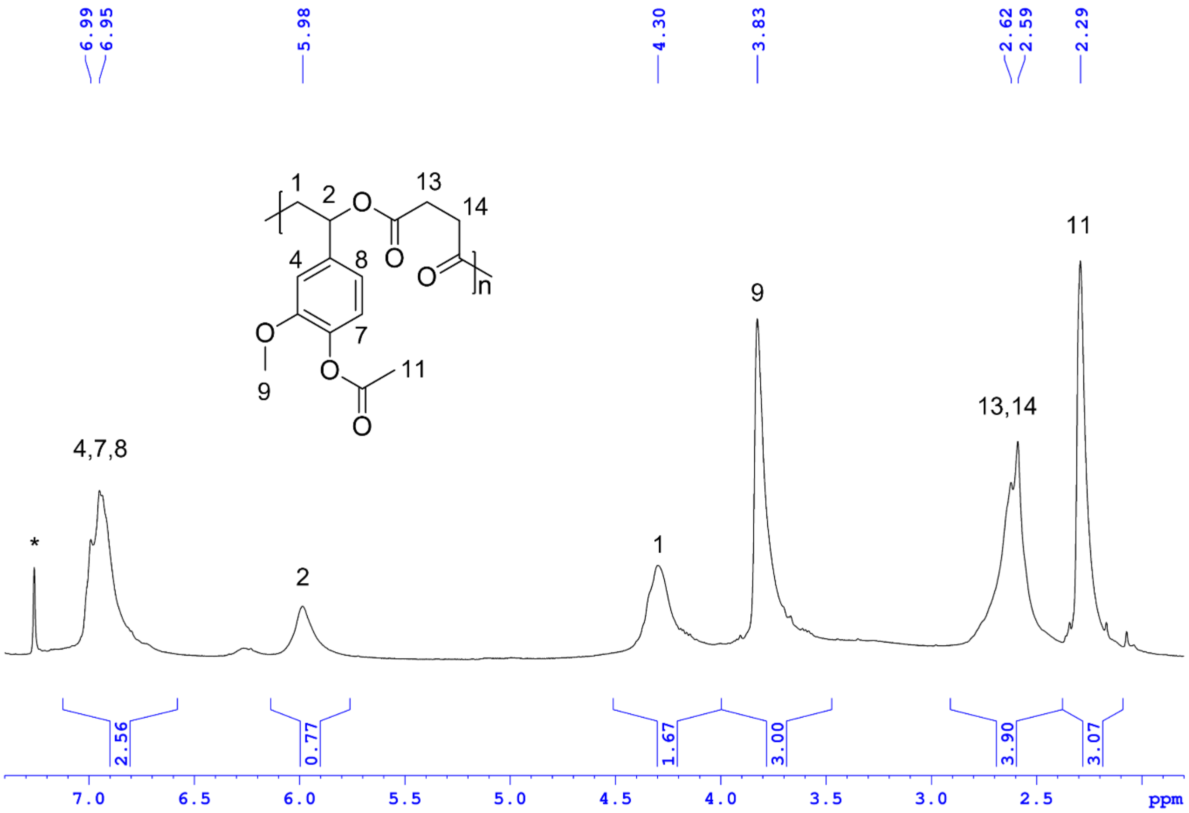

FigureS 56. ^{1}H NMR of P(EGA-*alt*-SA) (600 MHz, rt, $CDCl_3$).

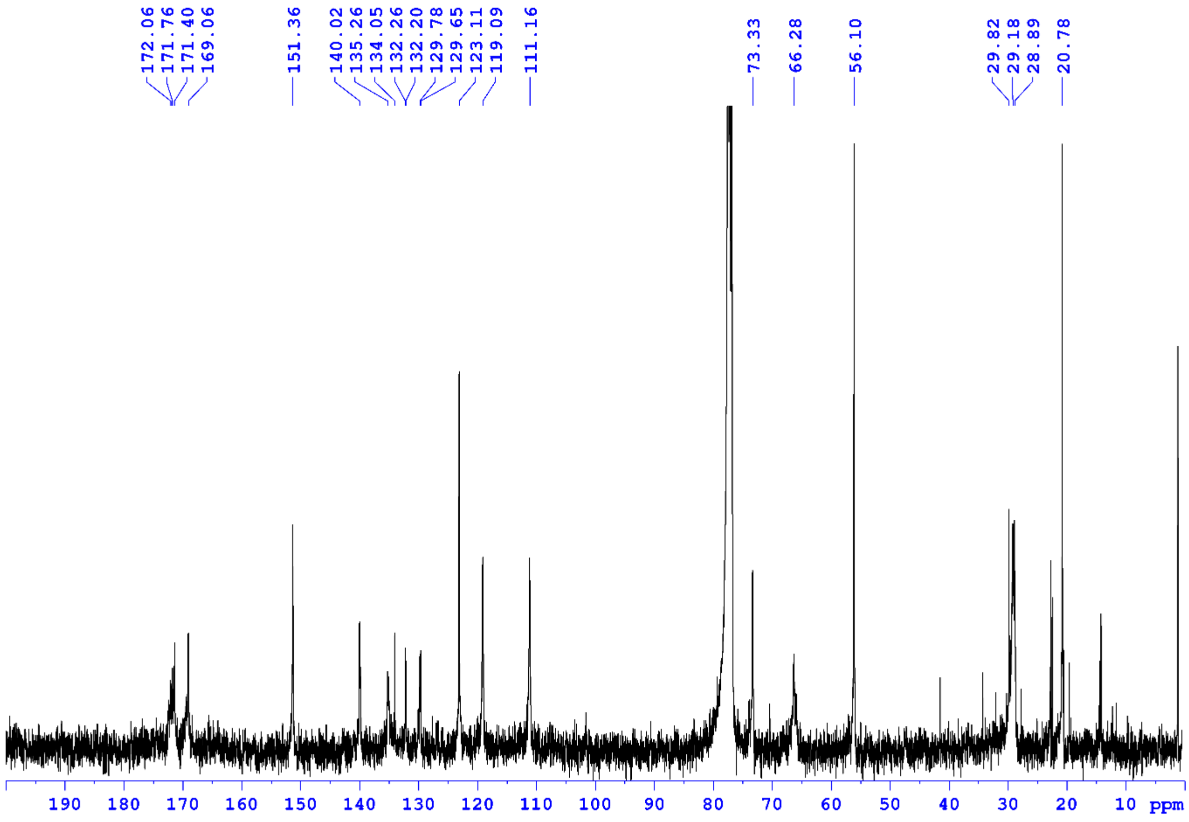

FigureS 57. ^{13}C NMR of P(EGA-*alt*-SA) (600 MHz, rt, $CDCl_3$).

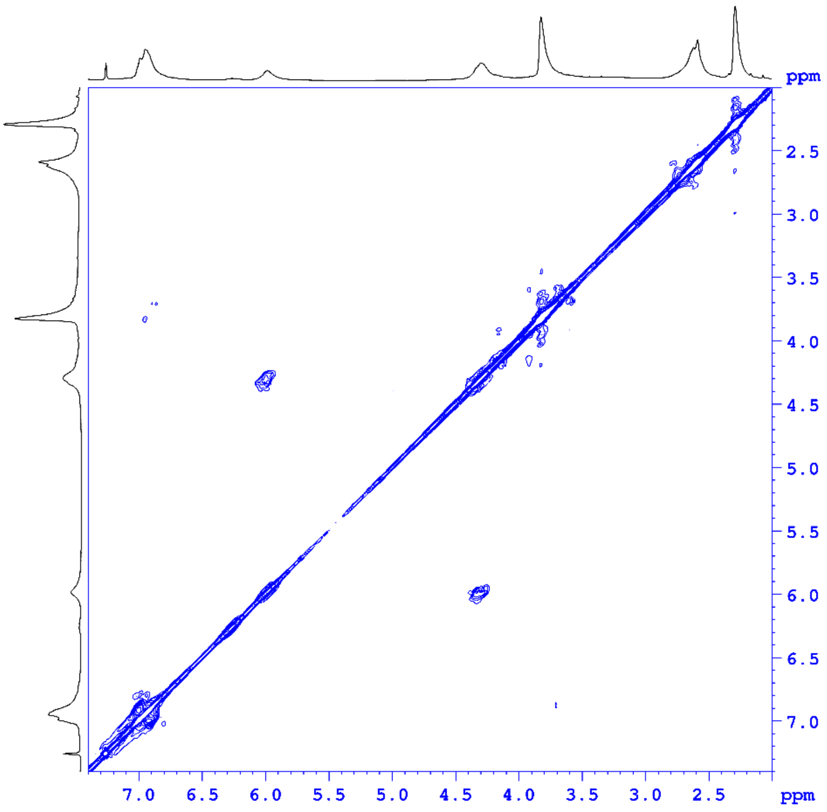

FigureS 58. ^{1}H-^{1}H COSY NMR of P(EGA-*alt*-SA) (600 MHz, rt, $CDCl_3$).

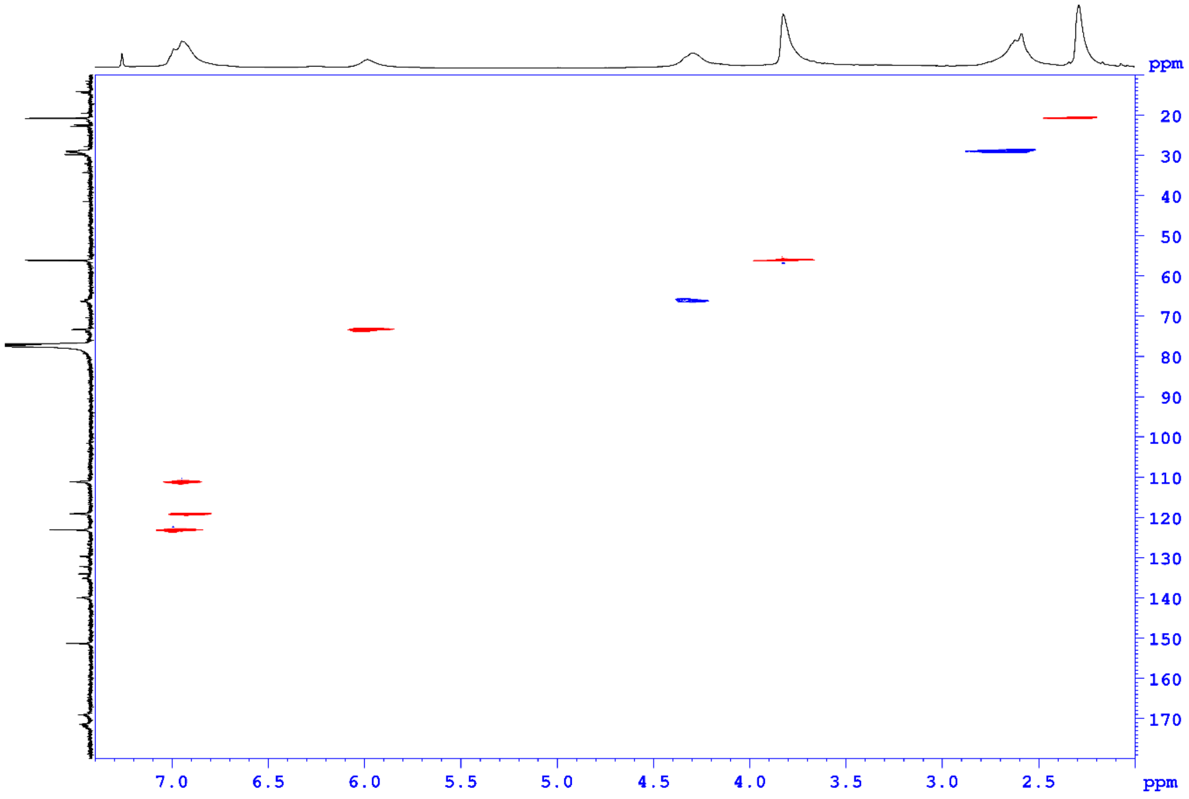

FigureS 59. ^{1}H-^{13}C HSQC NMR of P(EGA-*alt*-SA) (600 MHz, rt, $CDCl_3$).

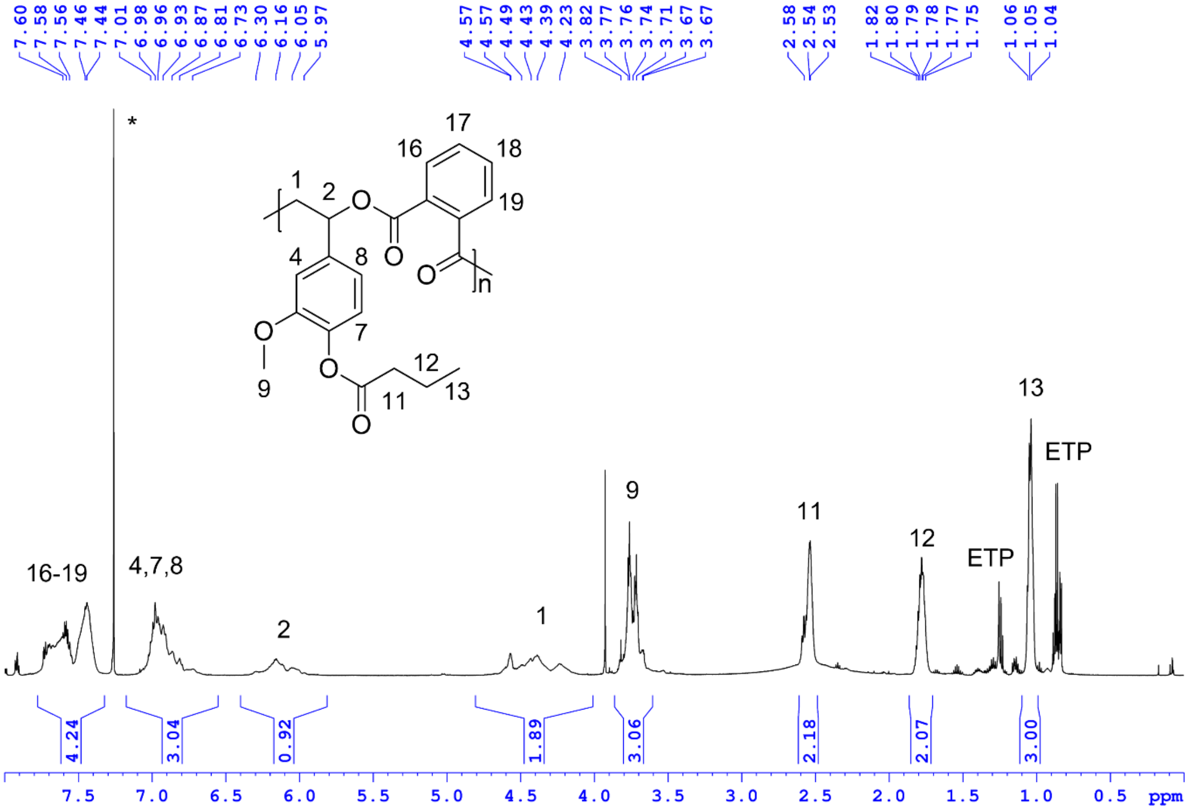

FigureS 60. ^{1}H NMR of P(EGB-*alt*-PA) (600 MHz, rt, $CDCl_3$).

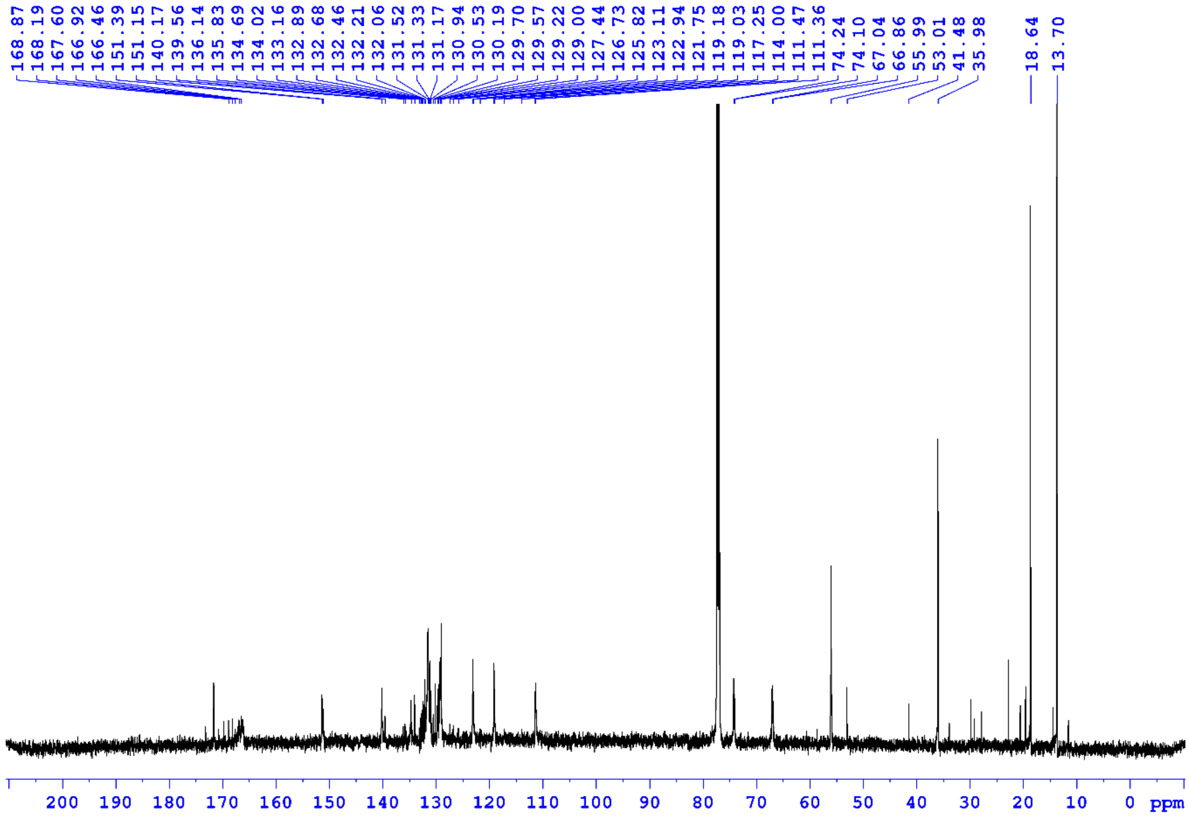

FigureS 61. ^{13}C NMR of P(EGB-*alt*-PA) (600 MHz, rt, $CDCl_3$).

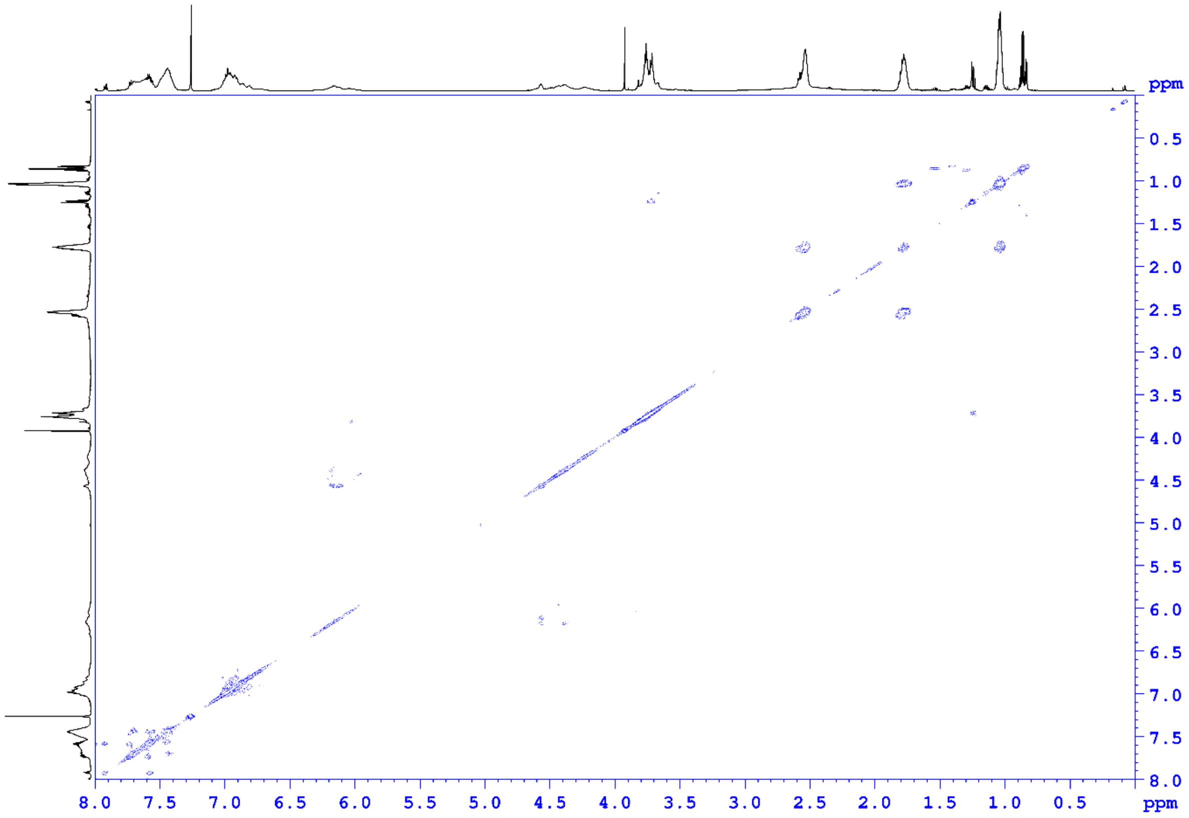

FigureS 62. ^{1}H-^{1}H COSY NMR of P(EGB-*alt*-PA) (600 MHz, rt, $CDCl_3$).

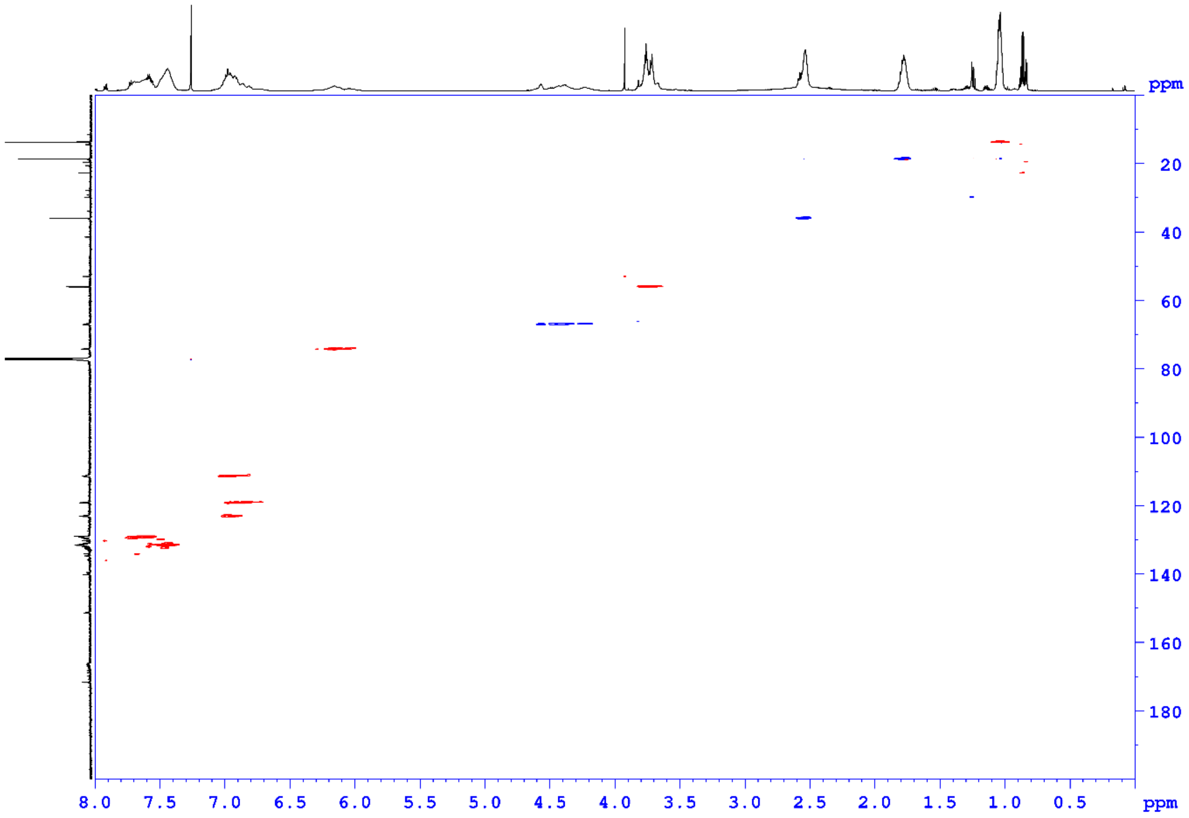

FigureS 63. ^{1}H-^{13}C HSQC NMR of P(EGB-*alt*-PA) (600 MHz, rt, $CDCl_3$).

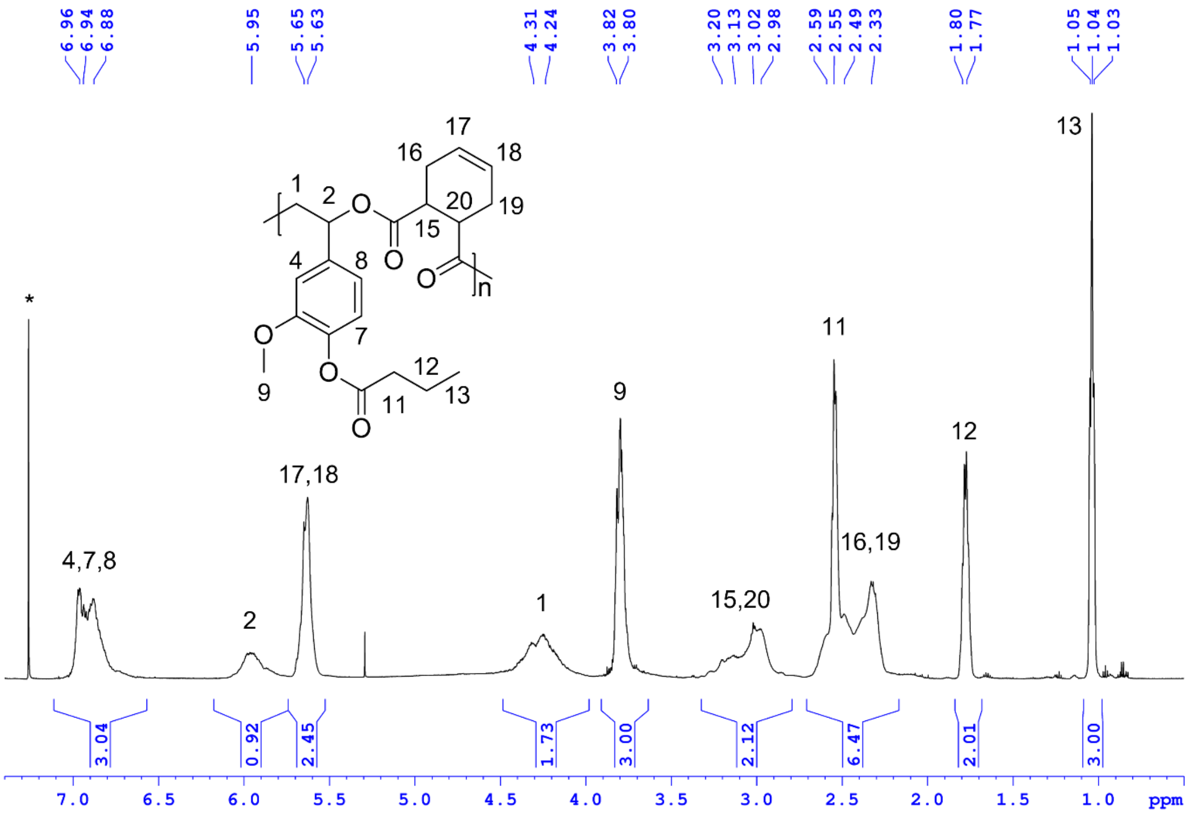

FigureS 64. ^{1}H NMR of P(EGB-*alt*-THPA) (600 MHz, rt, $CDCl_3$).

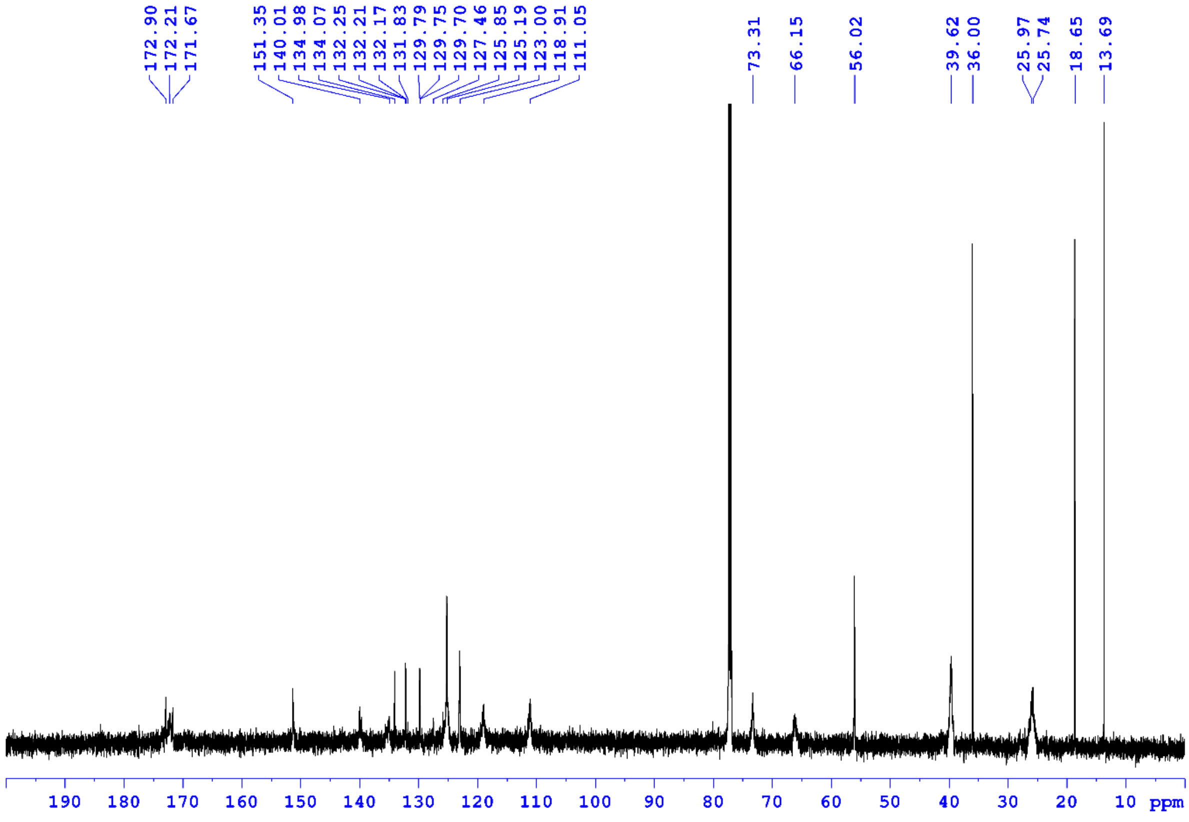

FigureS 65. ^{13}C NMR of P(EGB-*alt*-THPA) (600 MHz, rt, $CDCl_3$).

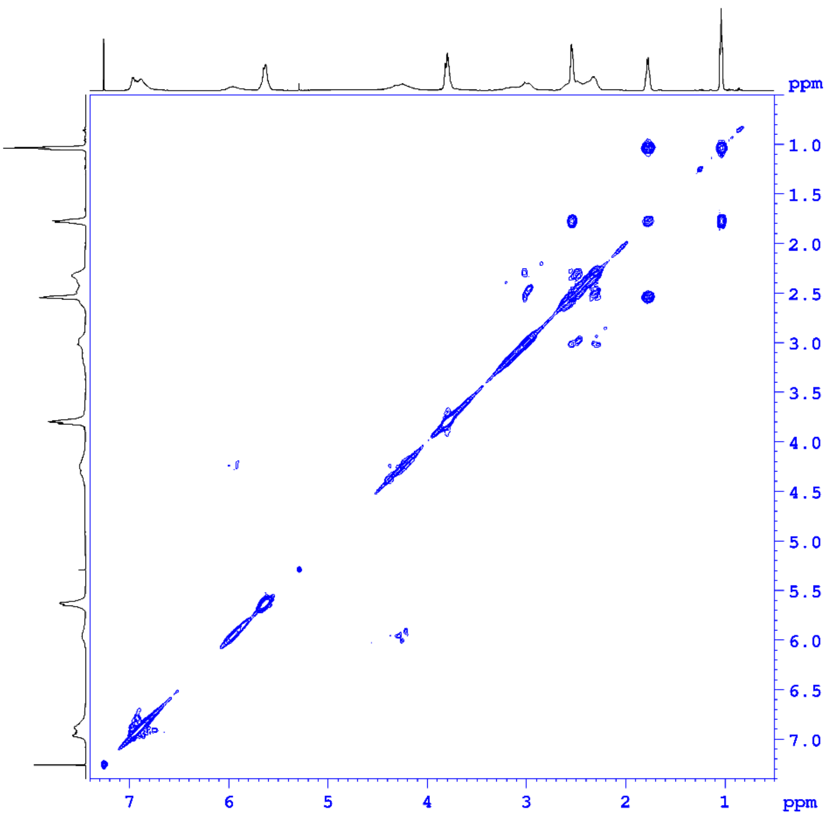

FigureS 66. ^1H-^1H COSY NMR of P(EGB-*alt*-THPA) (600 MHz, rt, $CDCl_3$).

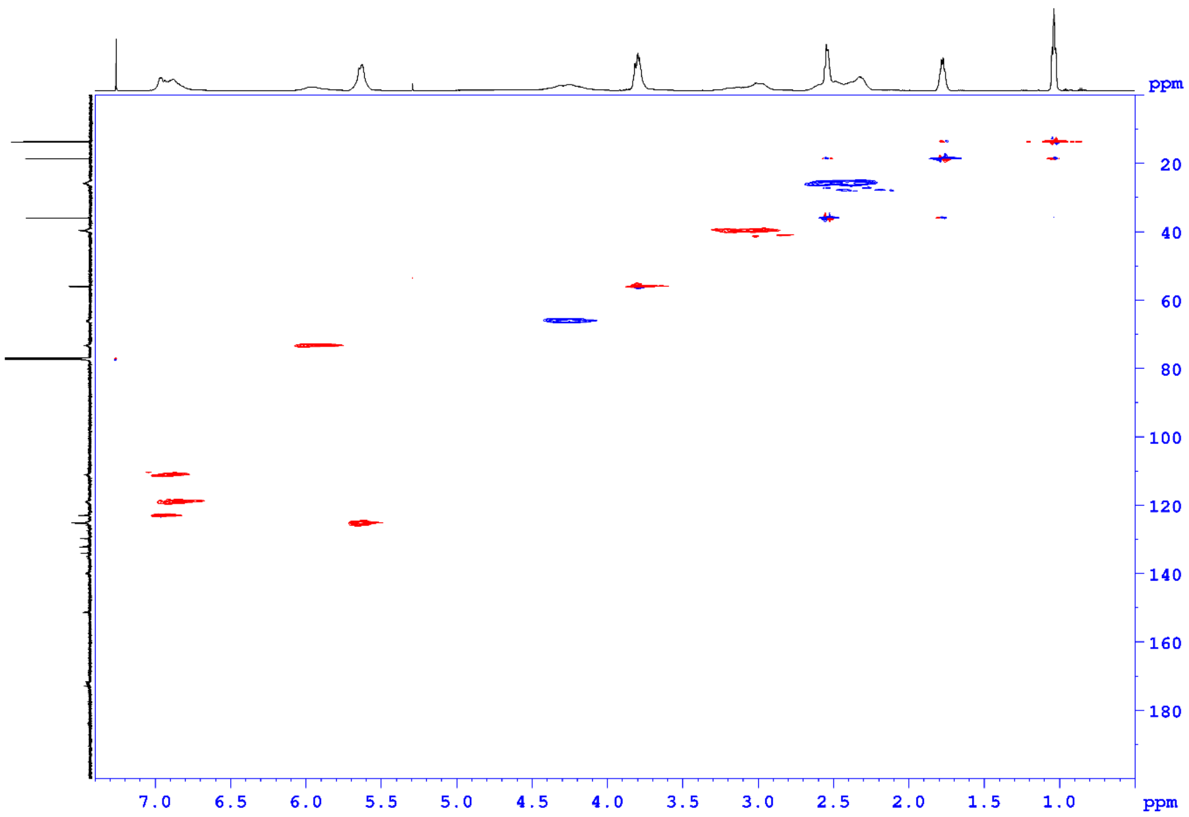

FigureS 67. ^1H-^{13}C HSQC NMR of P(EGB-*alt*-THPA) (600 MHz, rt, $CDCl_3$).

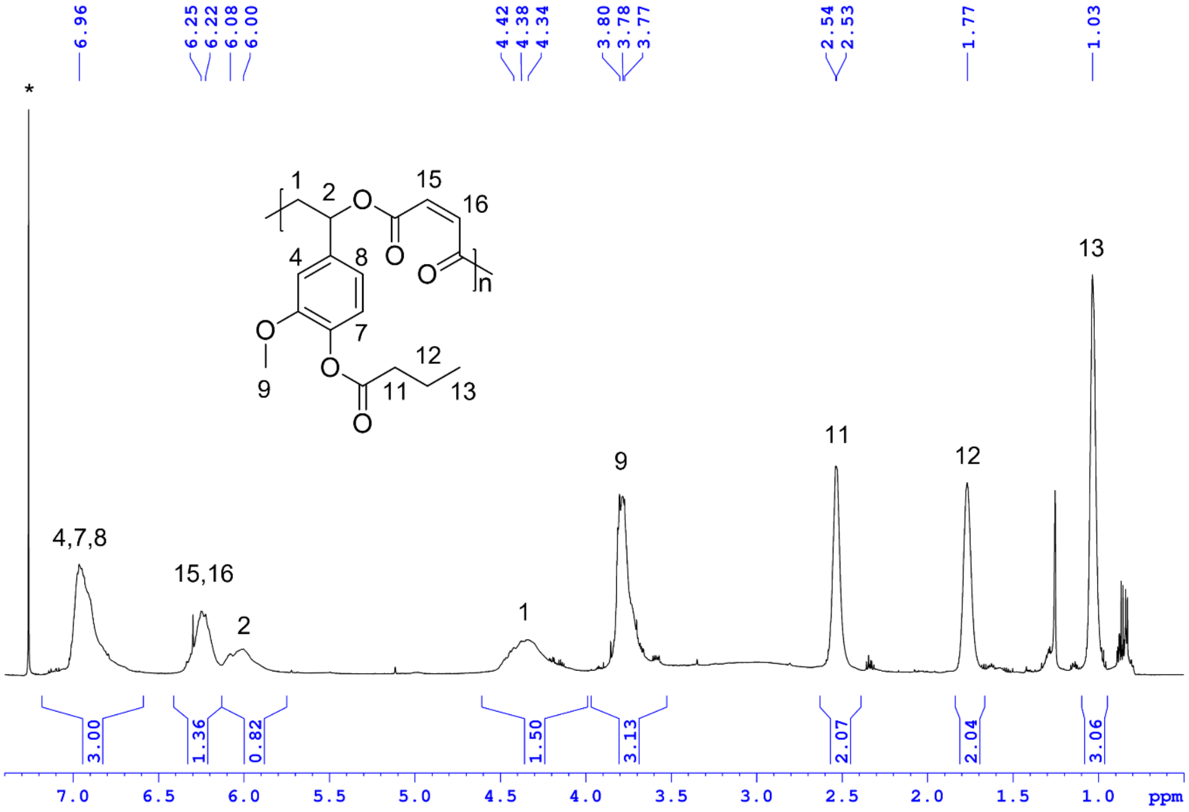

FigureS 68. ^{1}H NMR of P(EGB-*alt*-MA) (600 MHz, rt, $CDCl_3$).

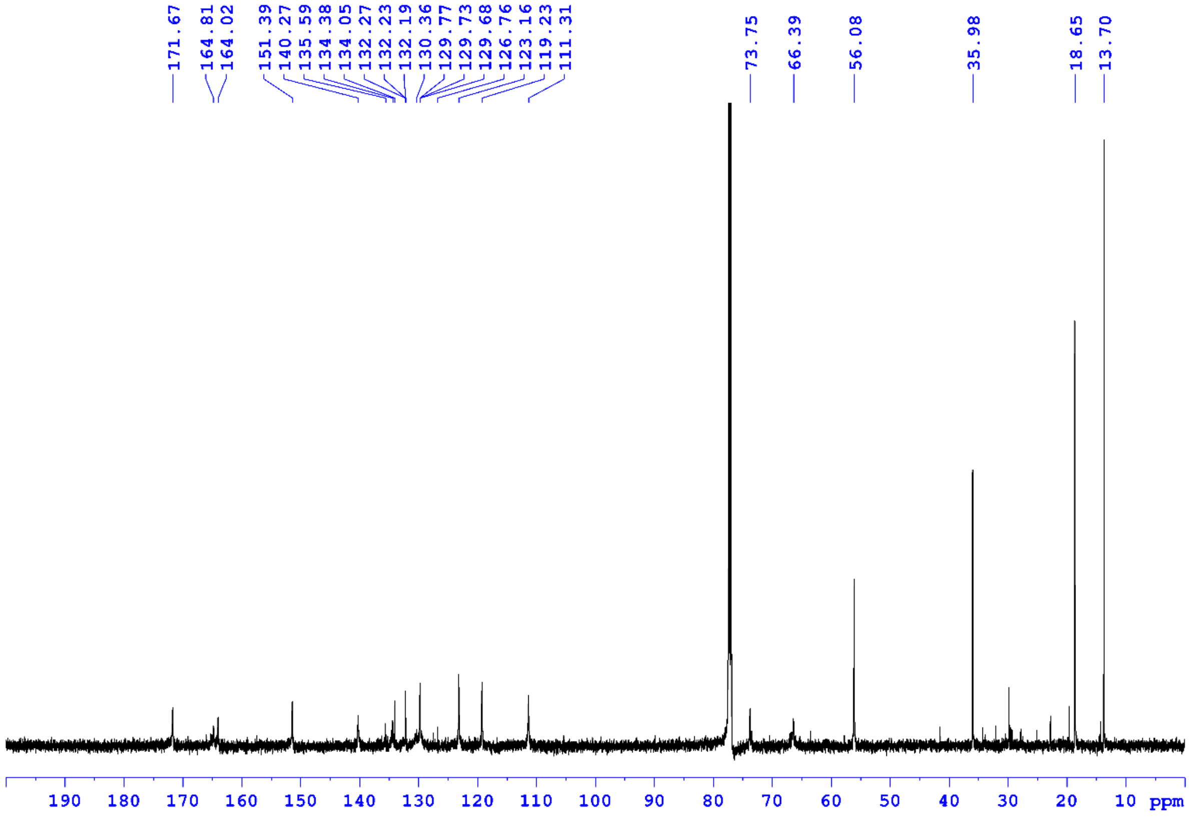

FigureS 69. ^{13}C NMR of P(EGB-*alt*-MA) (600 MHz, rt, $CDCl_3$).

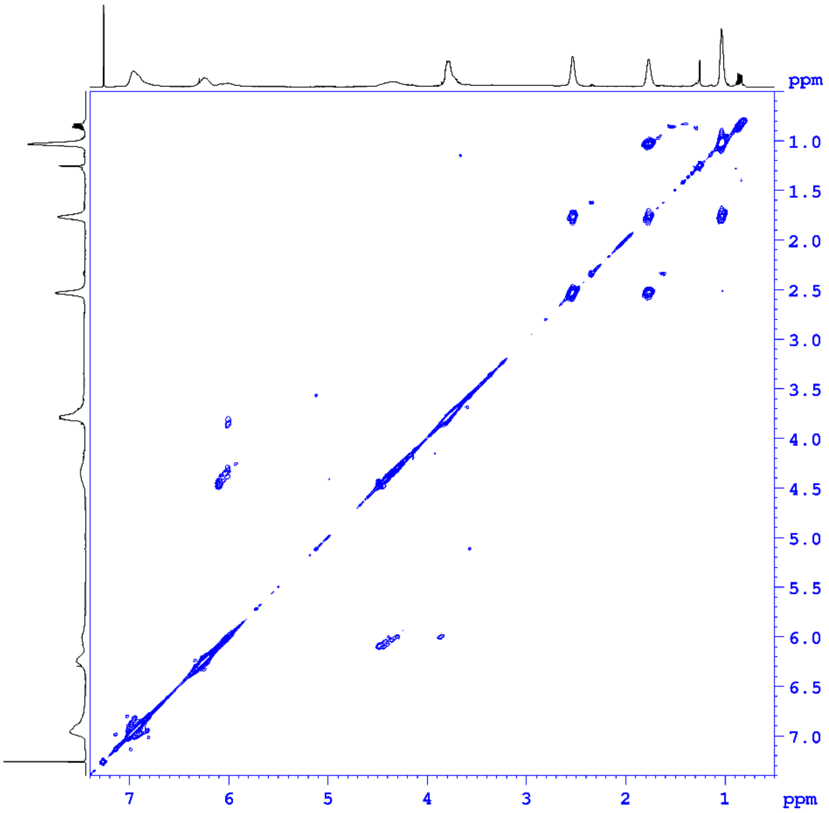

FigureS 70. ^{1}H-^{1}H COSY NMR of P(EGB-*alt*-MA) (600 MHz, rt, $CDCl_3$).

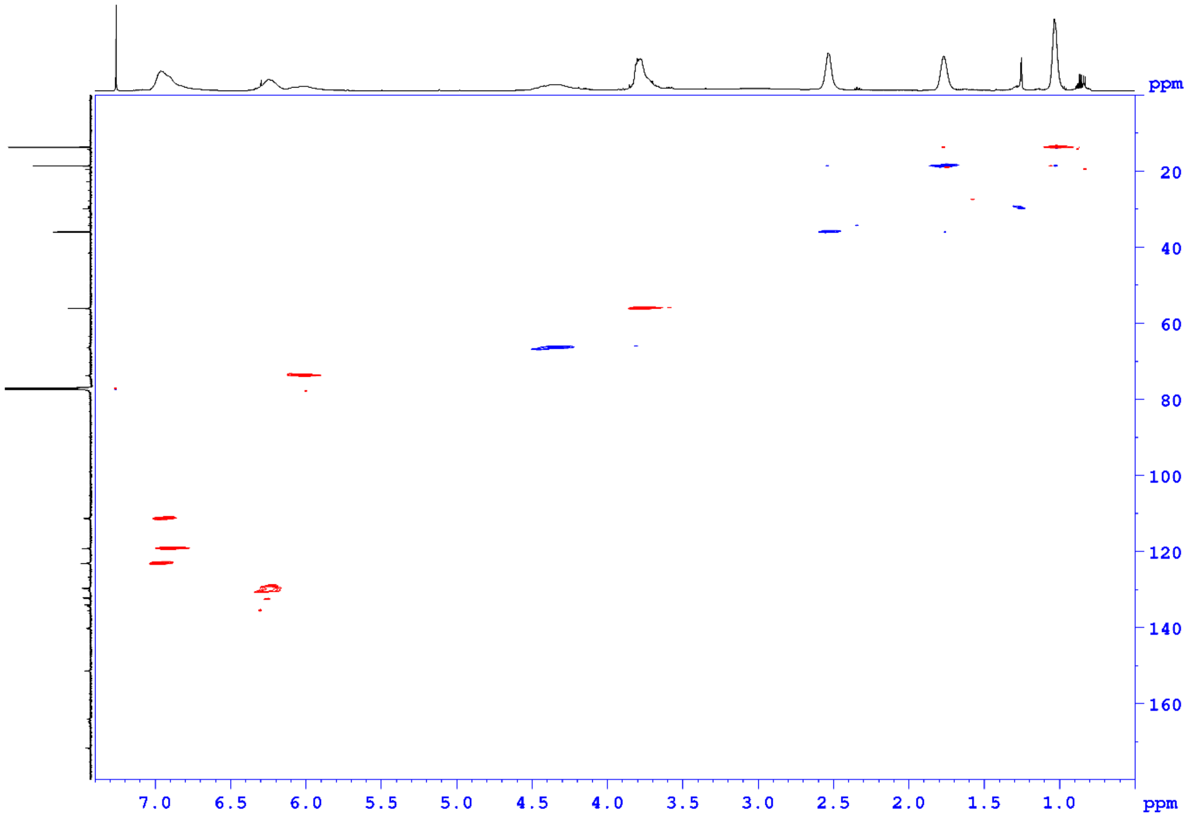

FigureS 71. ^{1}H-^{13}C HSQC NMR of P(EGB-*alt*-MA) (600 MHz, rt, $CDCl_3$).

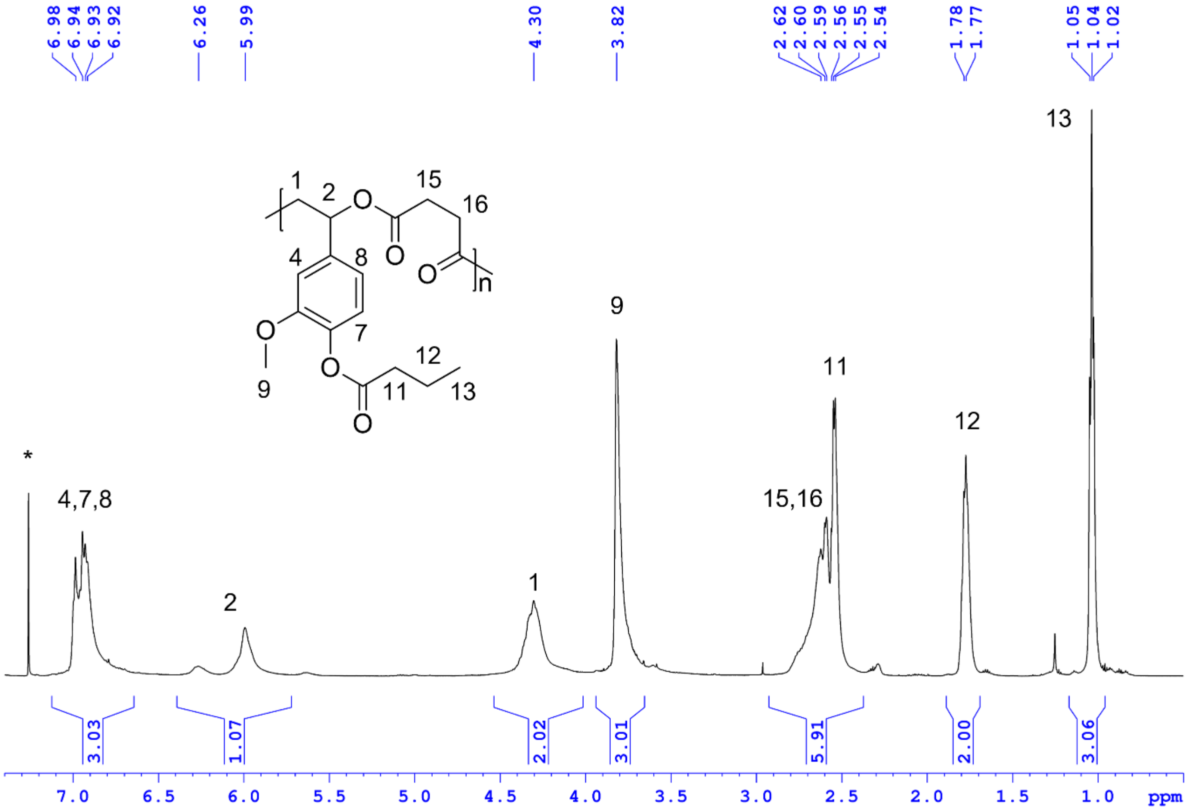

FigureS 72. ^{1}H NMR of P(EGB-*alt*-SA) (600 MHz, rt, $CDCl_3$).

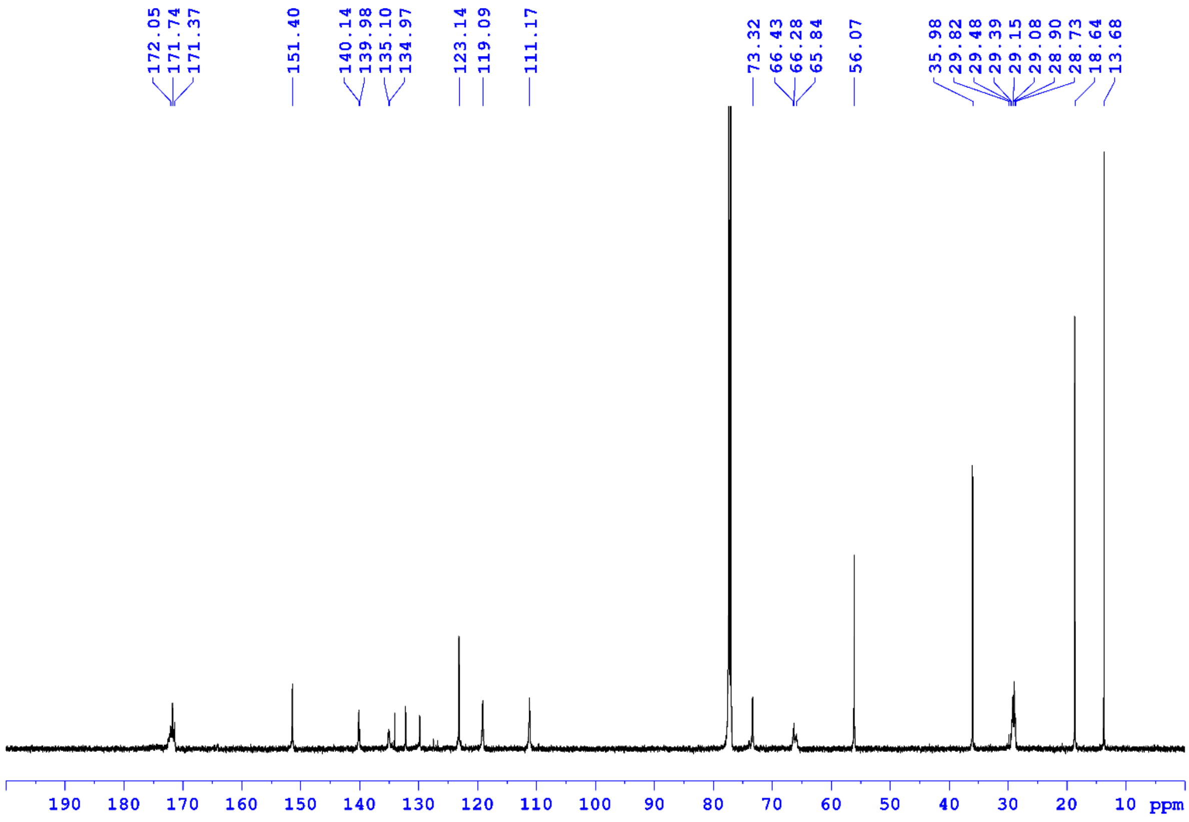

FigureS 73. ^{13}C NMR of P(EGB-*alt*-SA) (600 MHz, rt, $CDCl_3$).

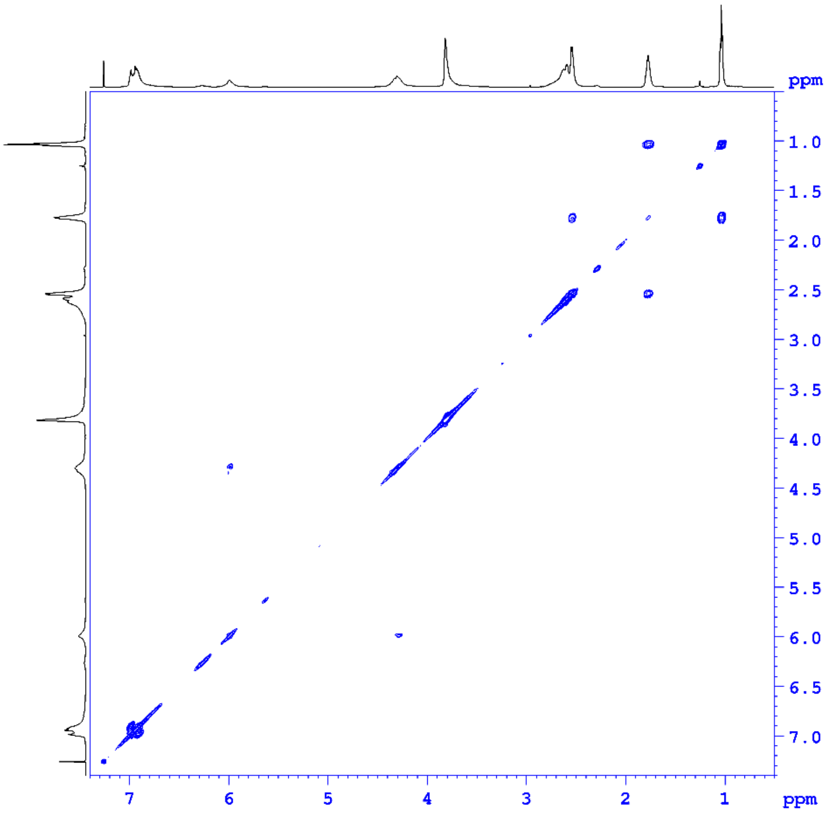

FigureS 74. 1H-1H COSY NMR of P(EGB-*alt*-SA) (600 MHz, rt, $CDCl_3$).

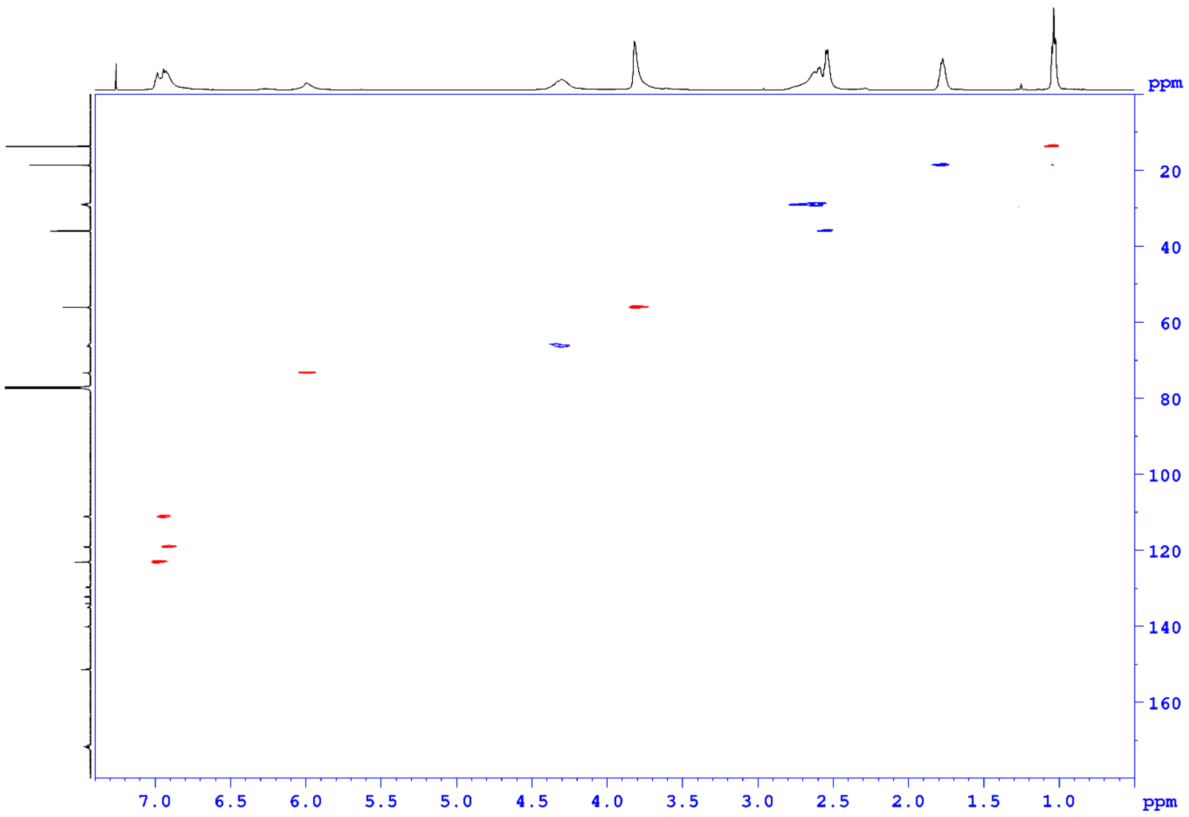

FigureS 75. 1H-13C HSQC NMR of P(EGB-*alt*-SA) (600 MHz, rt, $CDCl_3$).

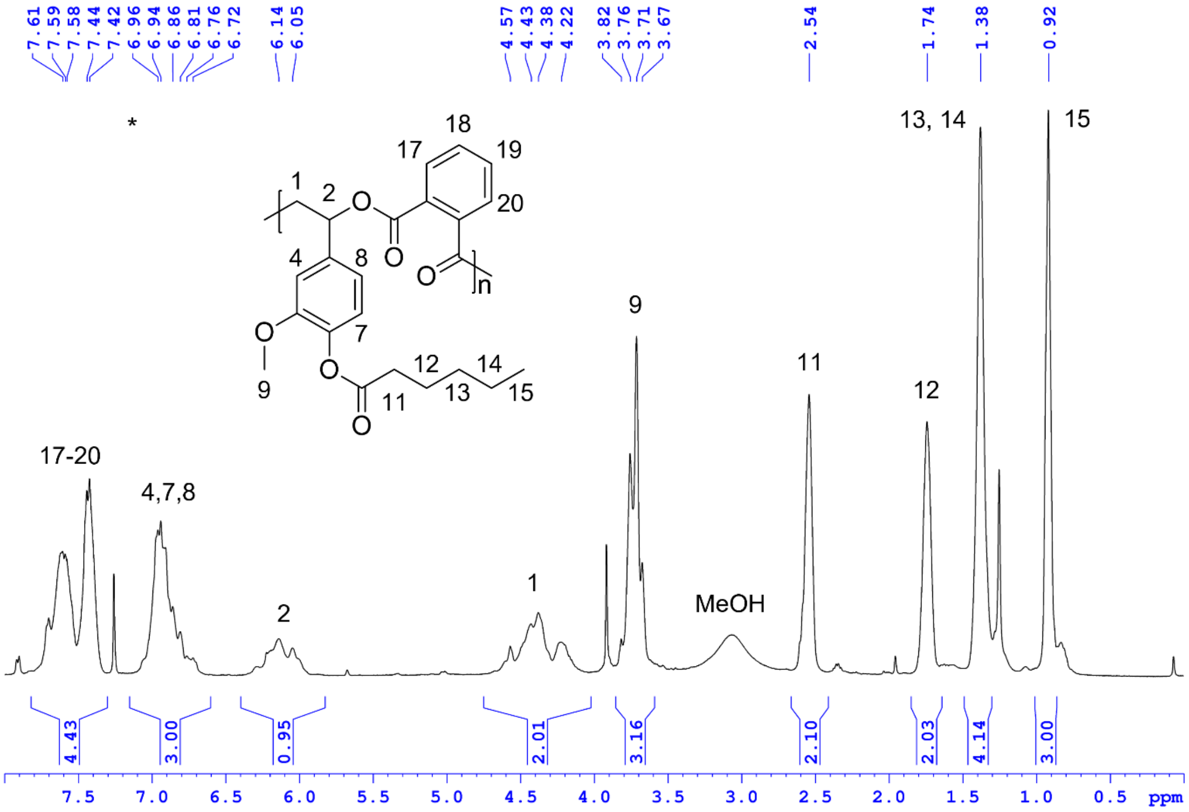

FigureS 76. ^{1}H NMR of P(EGH-*alt*-PA) (600 MHz, rt, $CDCl_3$).

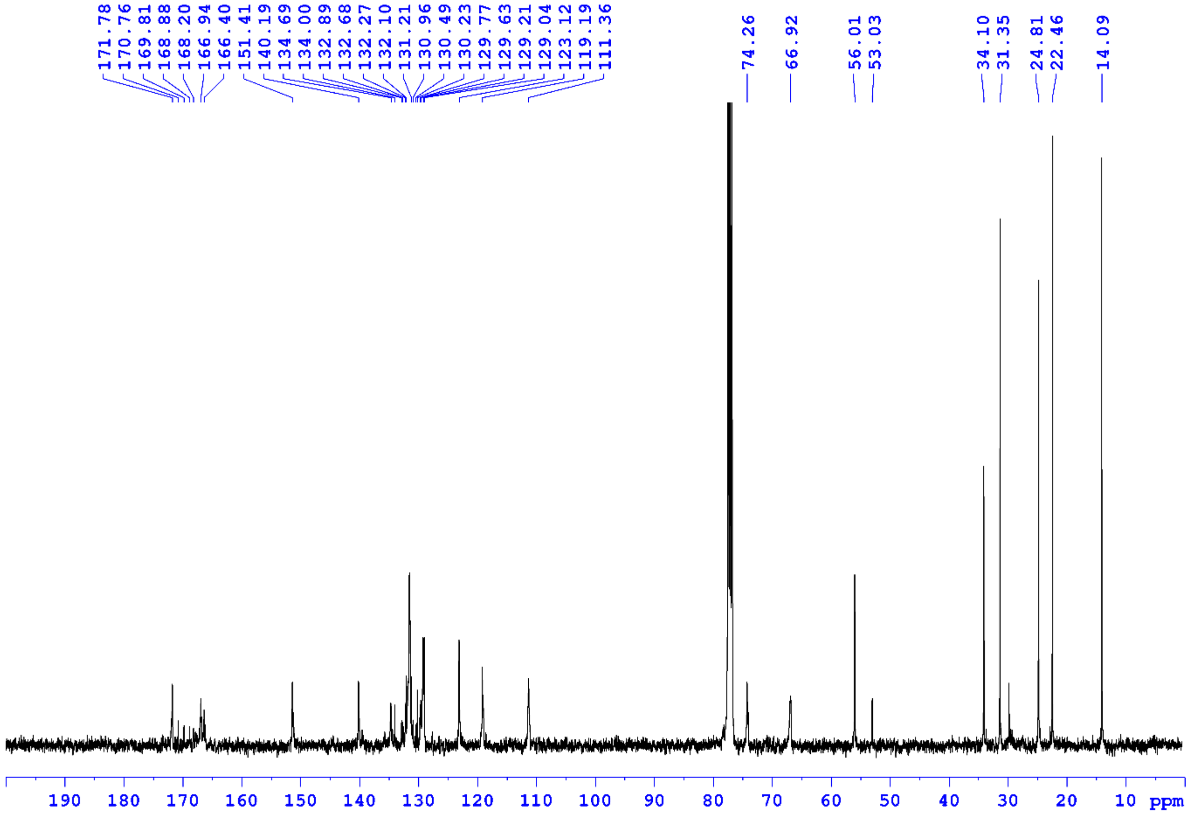

FigureS 77. ^{13}C NMR of P(EGH-*alt*-PA) (600 MHz, rt, $CDCl_3$).

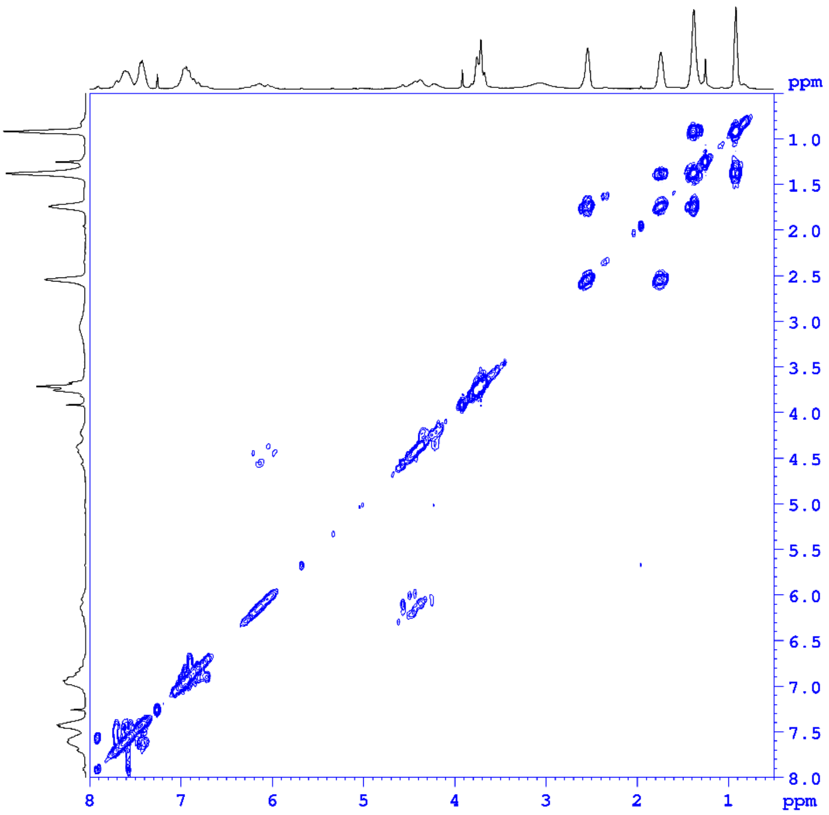

FigureS 78. ^{1}H-^{1}H COSY NMR of P(EGH-*alt*-PA) (600 MHz, rt, $CDCl_3$).

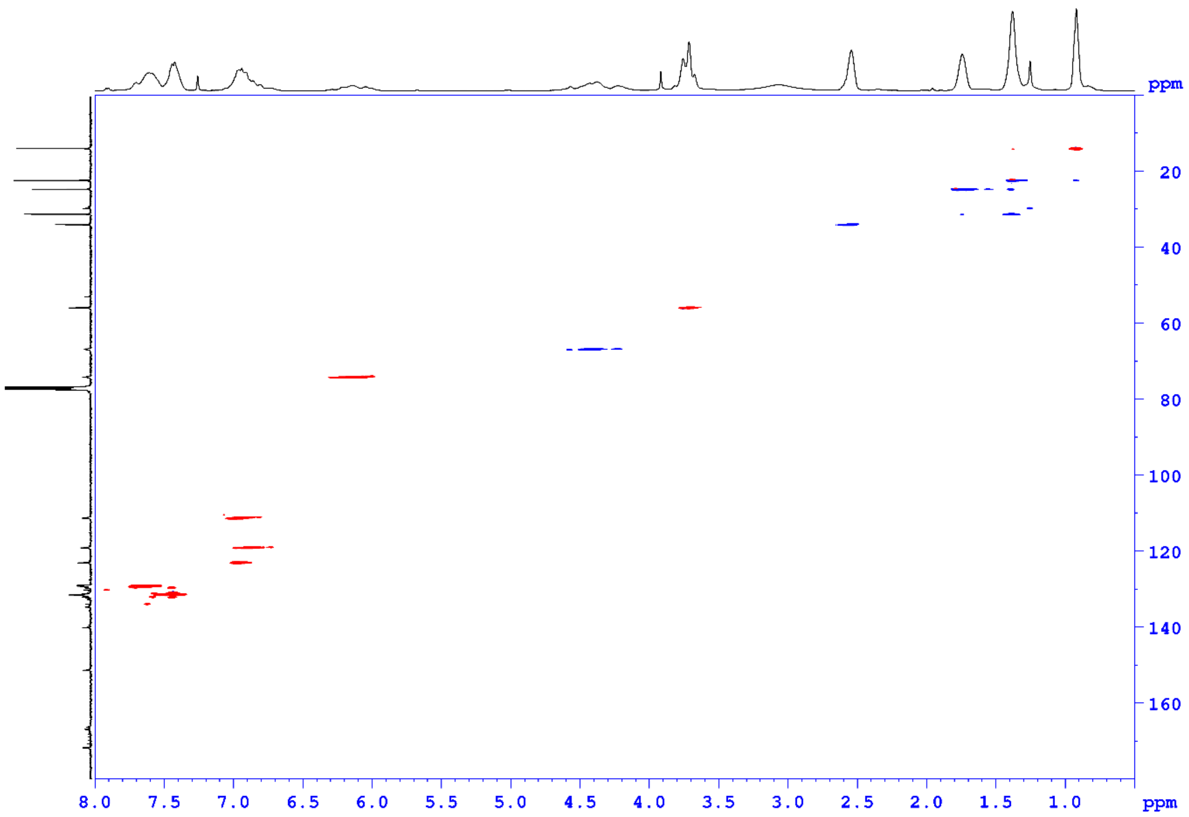

FigureS 79. ^{1}H-^{13}C HSQC NMR of P(EGH-*alt*-PA) (600 MHz, rt, $CDCl_3$).

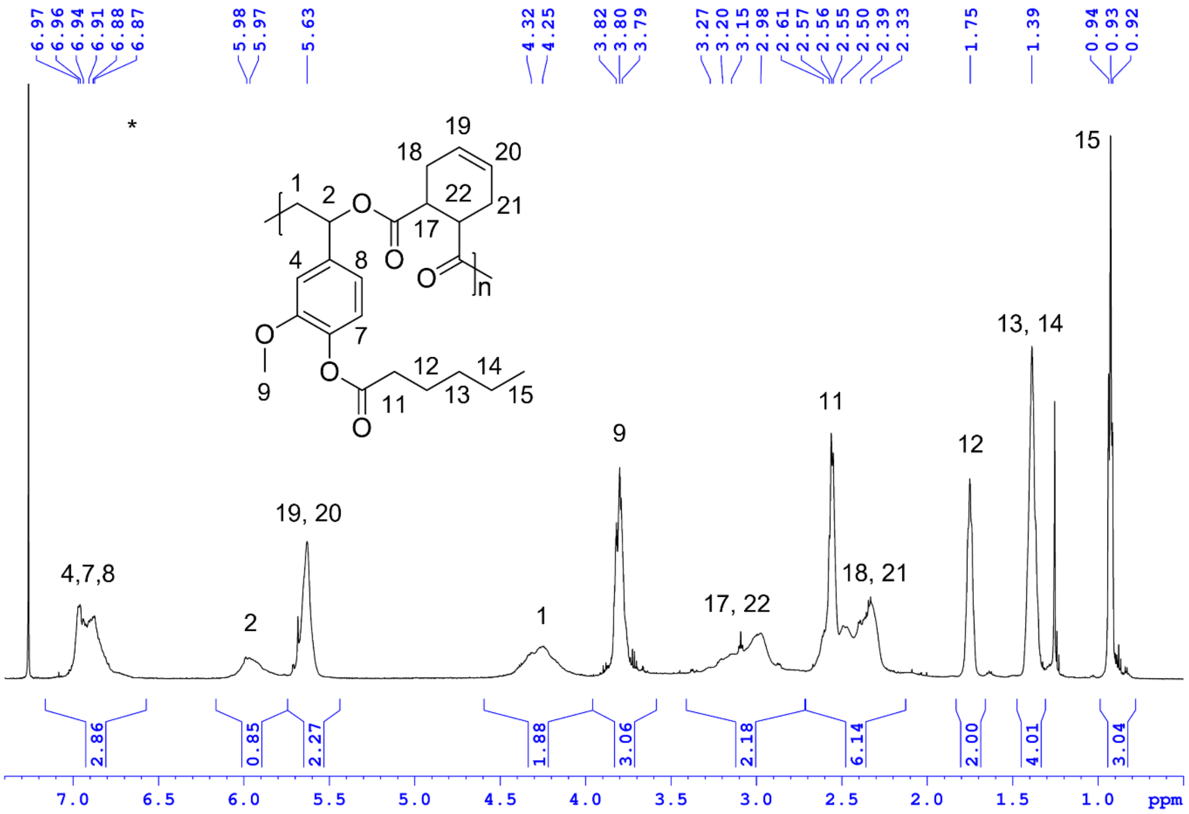

FigureS 80. ^{1}H NMR of P(EGH-*alt*-THPA) (600 MHz, rt, $CDCl_3$).

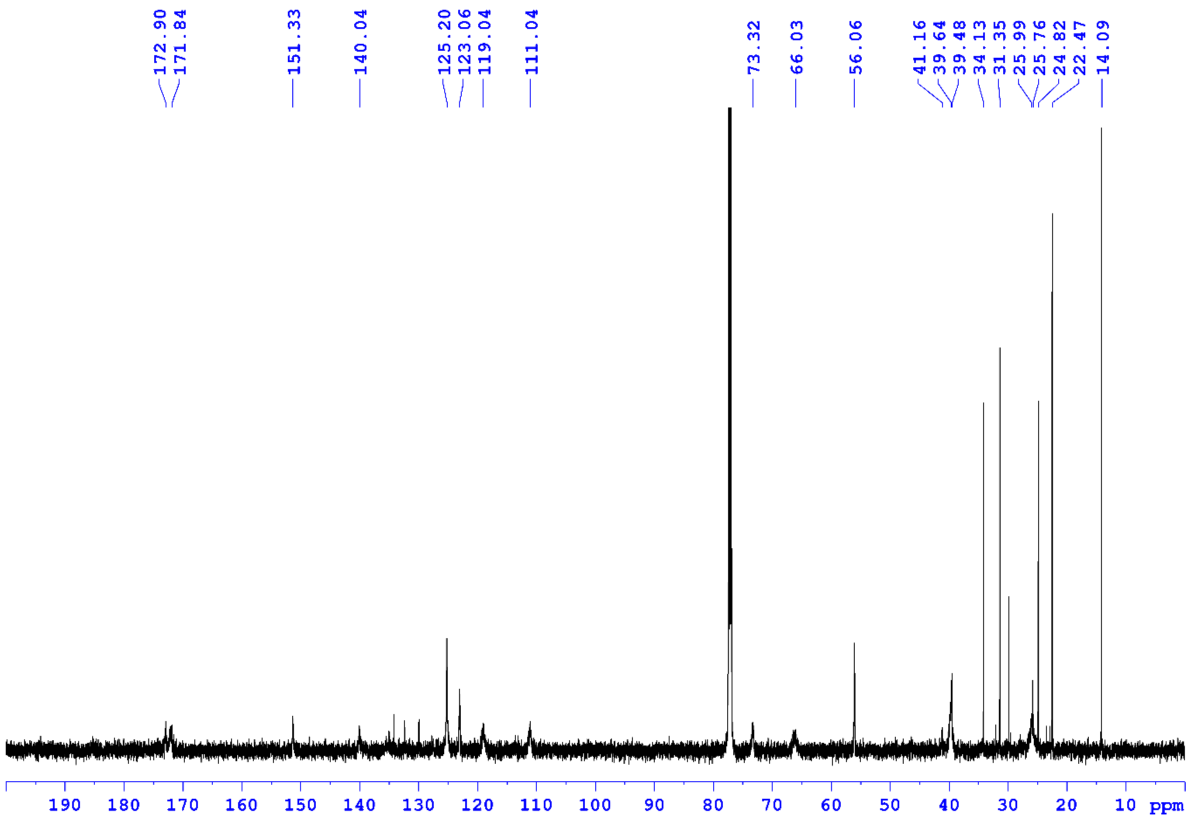

FigureS 81. ^{13}C NMR of P(EGH-*alt*-THPA) (600 MHz, rt, $CDCl_3$).

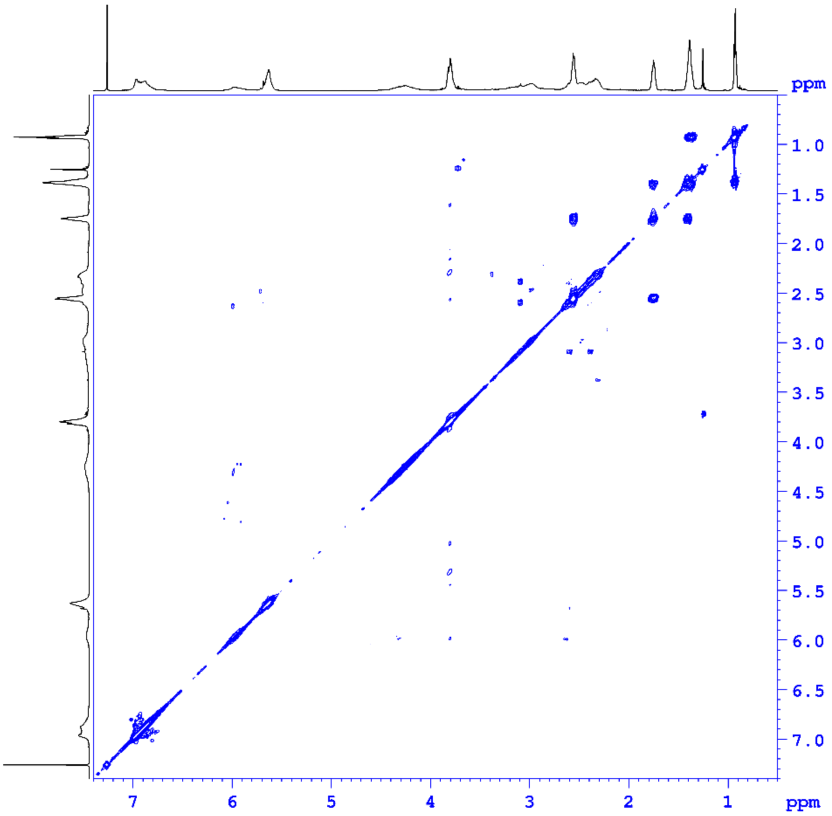

FigureS 82. ^{1}H-^{1}H COSY NMR of P(EGH-*alt*-THPA) (600 MHz, rt, $CDCl_3$).

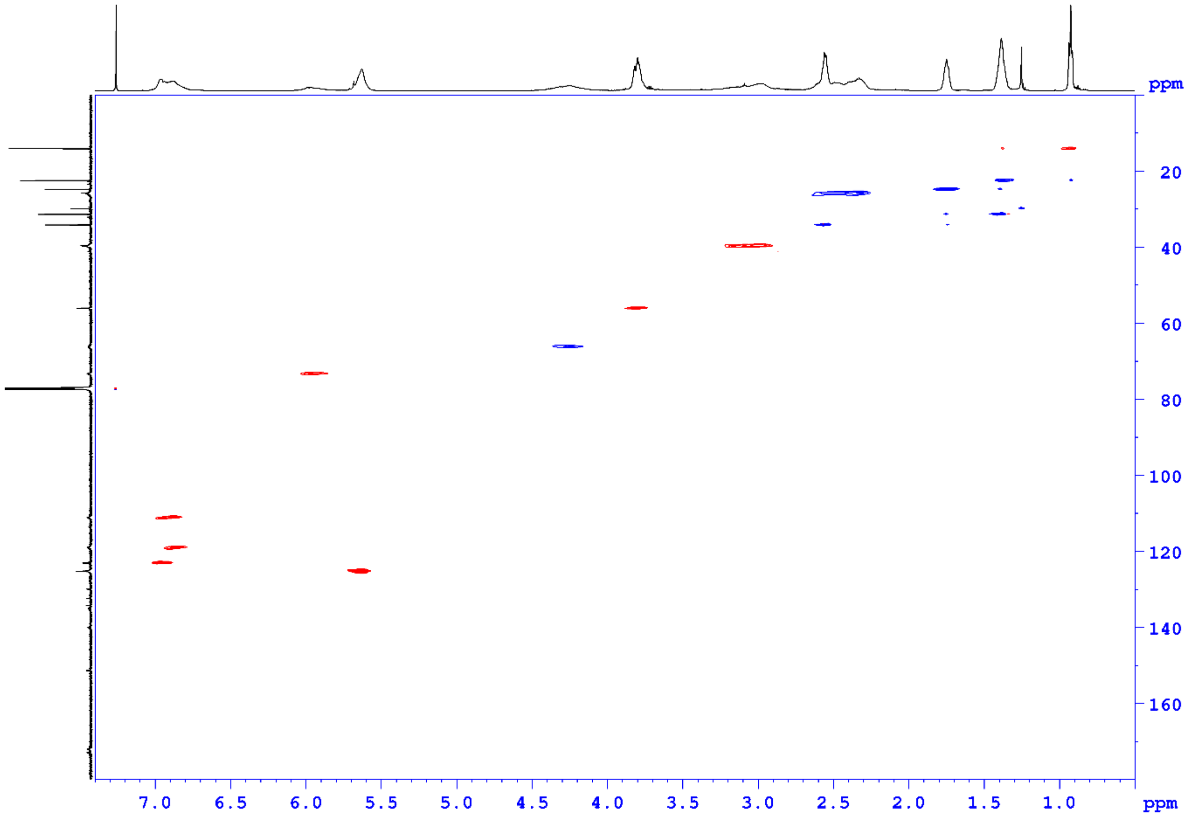

FigureS 83. ^{1}H-^{13}C HSQC NMR of P(EGH-*alt*-PA) (600 MHz, rt, $CDCl_3$).

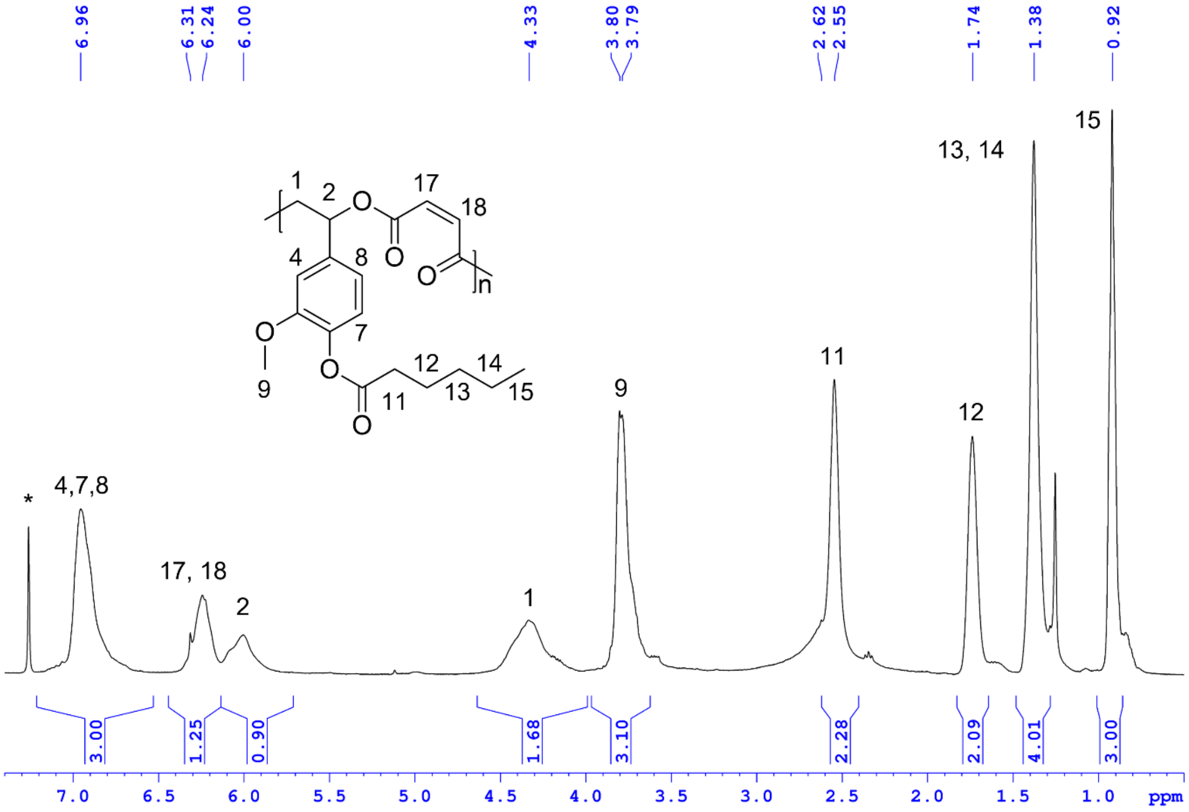

FigureS 84. ^{1}H NMR of P(EGH-*alt*-MA) (600 MHz, rt, $CDCl_3$).

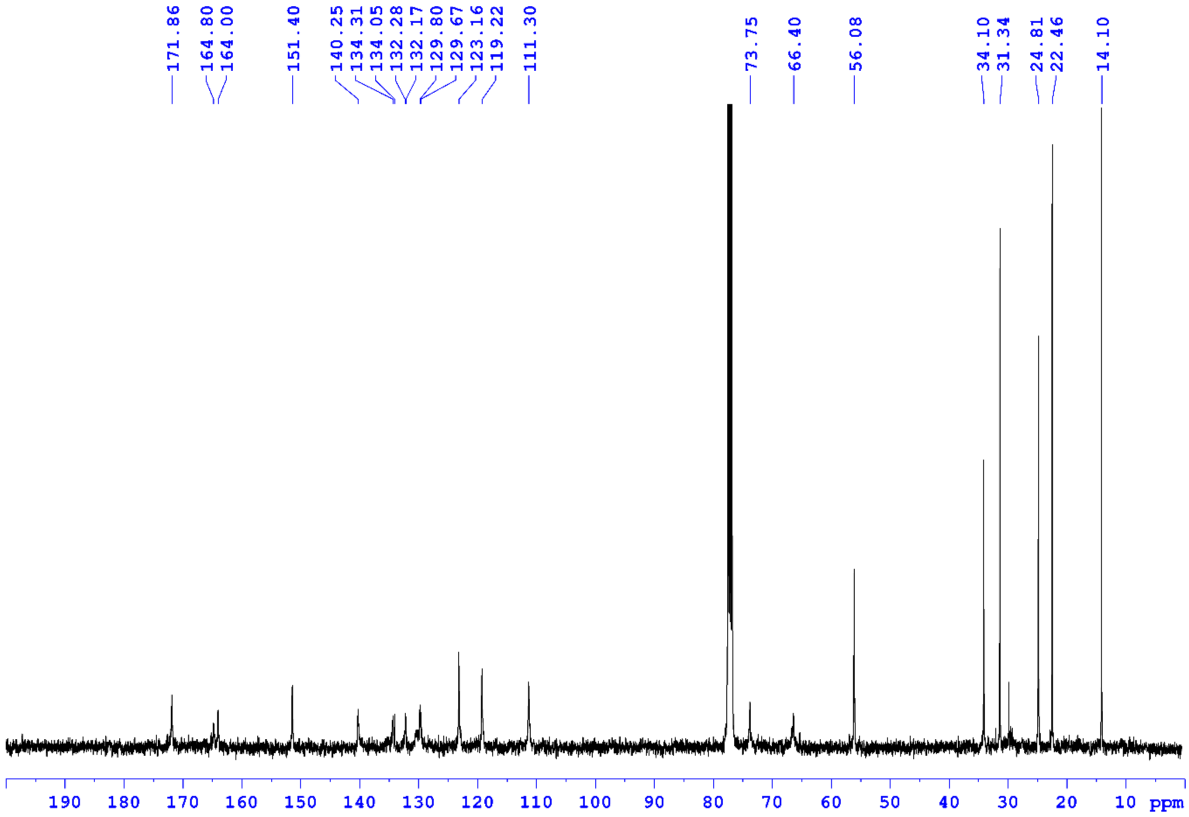

FigureS 85. ^{13}C NMR of P(EGH-*alt*-MA) (600 MHz, rt, $CDCl_3$).

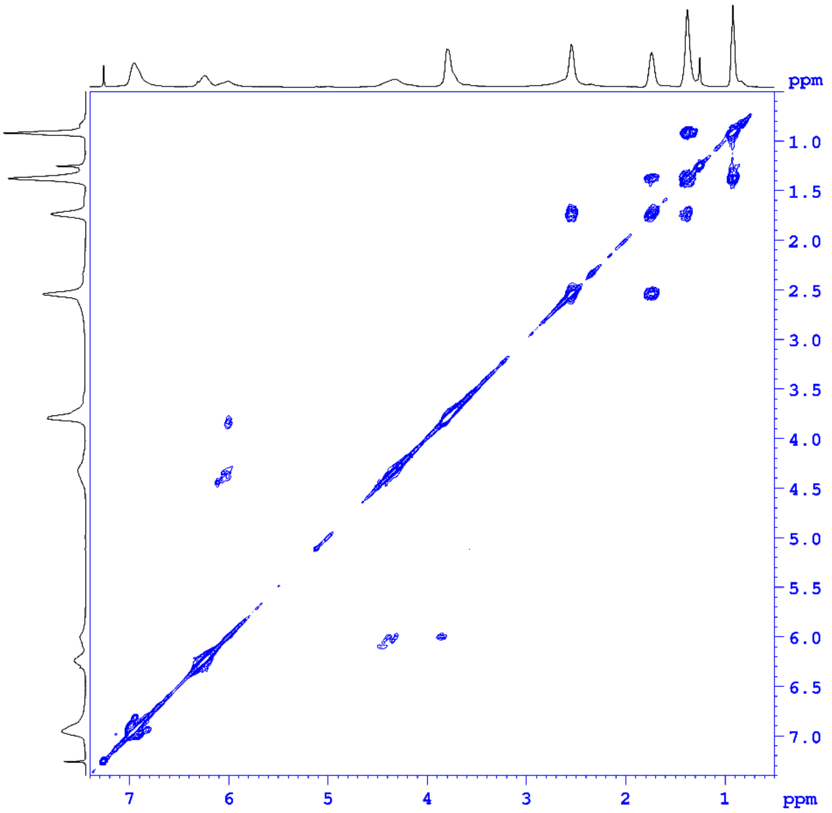

FigureS 86. ^{1}H-^{1}H COSY NMR of P(EGH-*alt*-MA) (600 MHz, rt, $CDCl_3$).

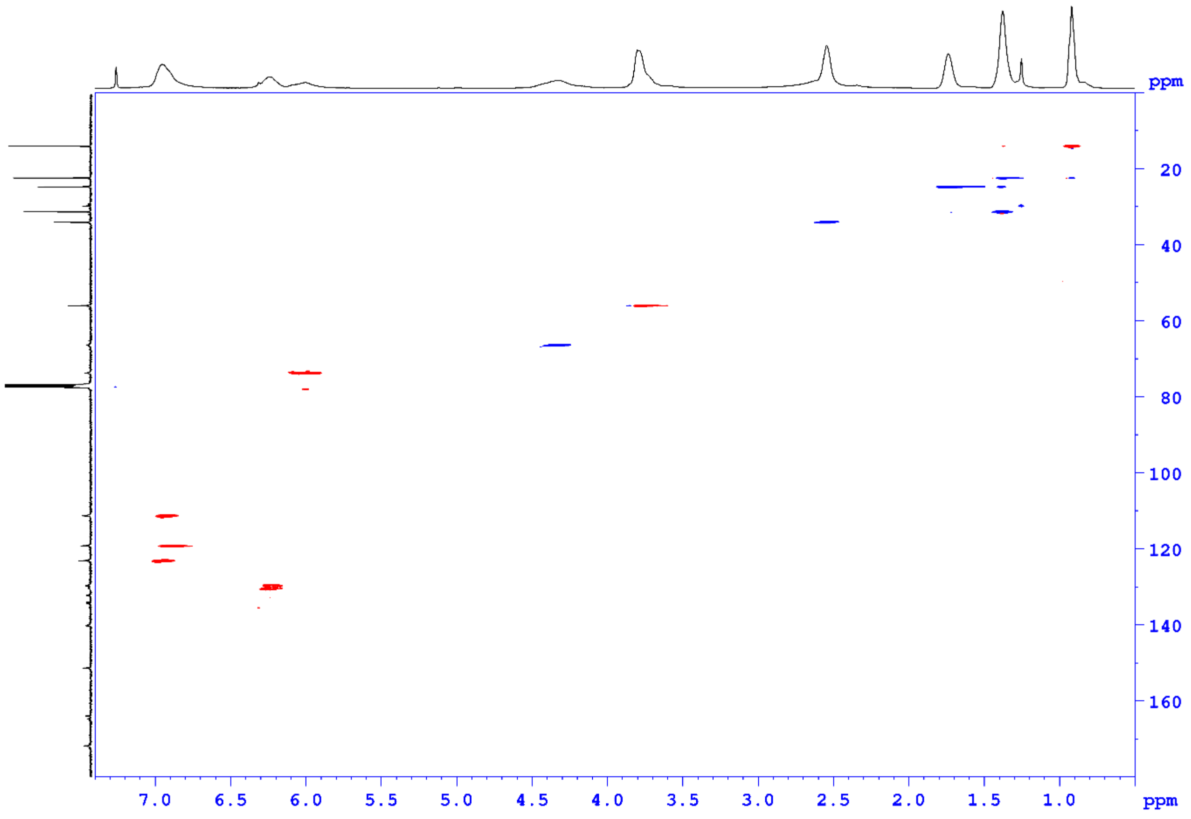

FigureS 87. ^{1}H-^{13}C HSQC NMR of P(EGH-*alt*-MA) (600 MHz, rt, $CDCl_3$).

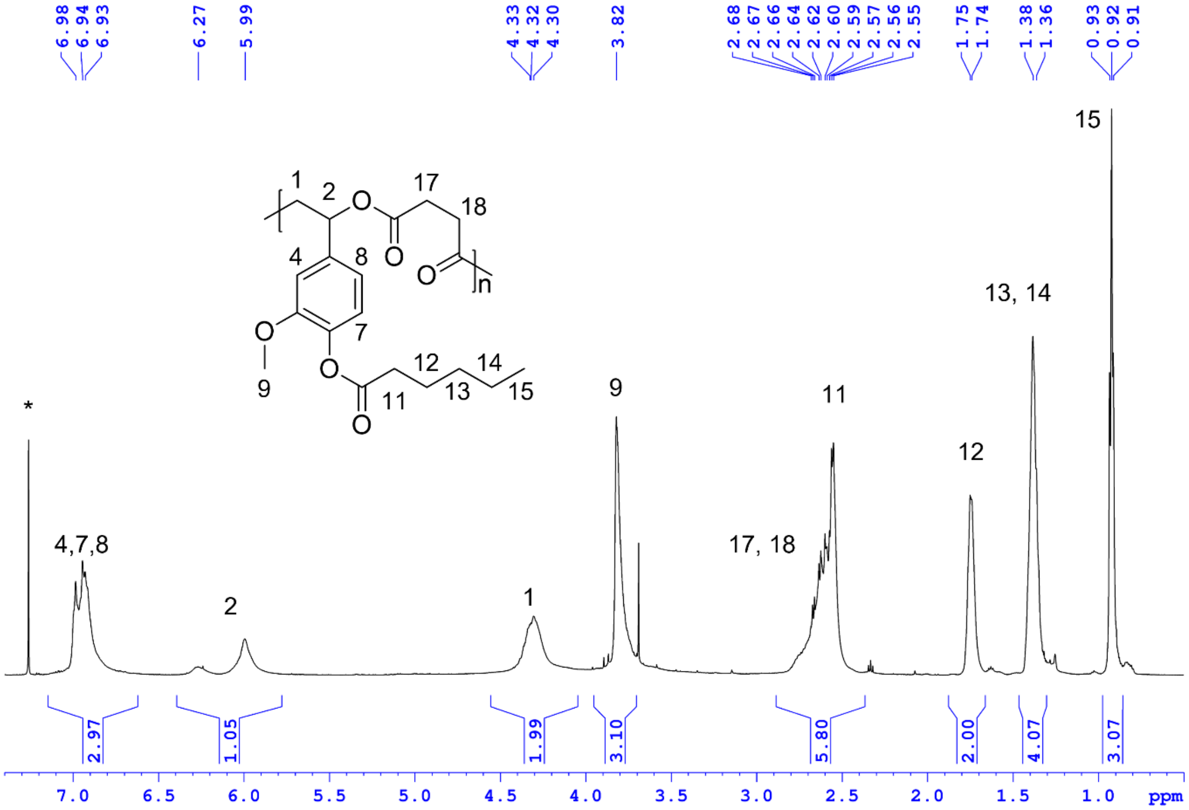

FigureS 88. ^{1}H NMR of P(EGH-*alt*-SA) (600 MHz, rt, $CDCl_3$).

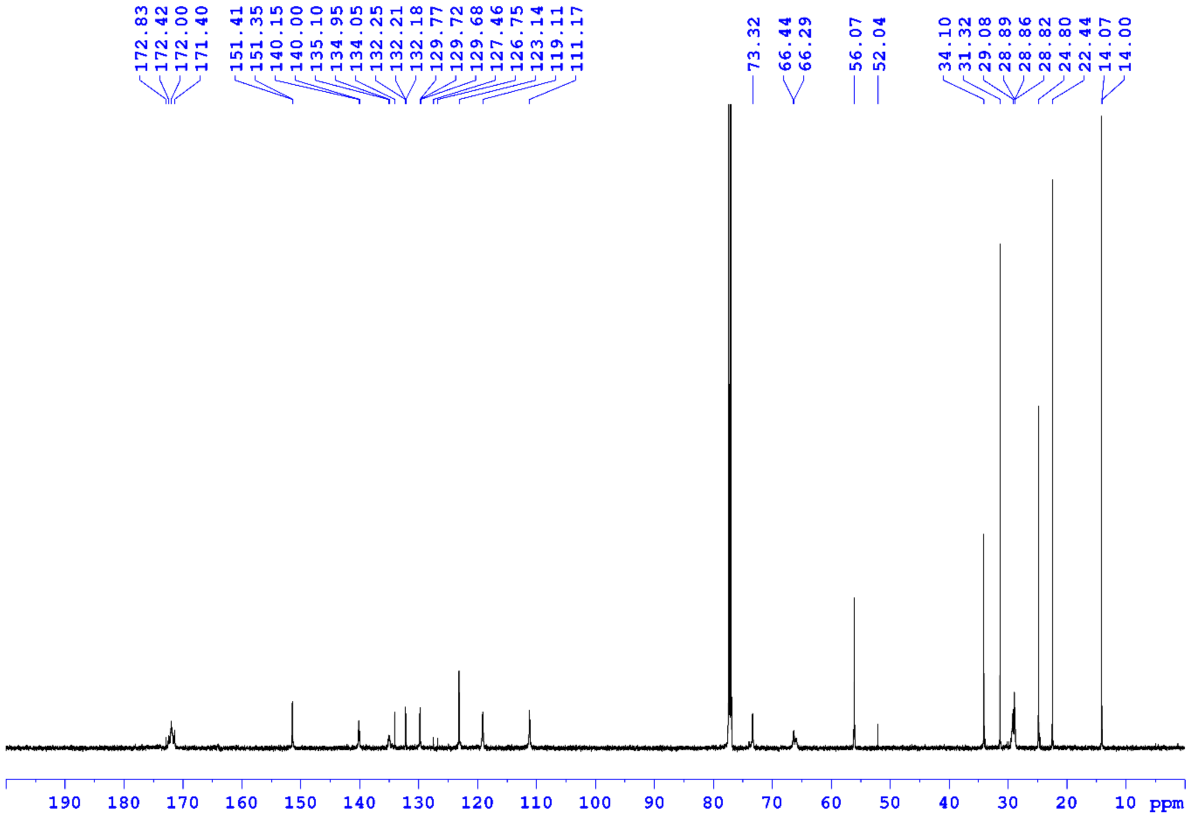

FigureS 89. ^{13}C NMR of P(EGH-*alt*-SA) (600 MHz, rt, $CDCl_3$).

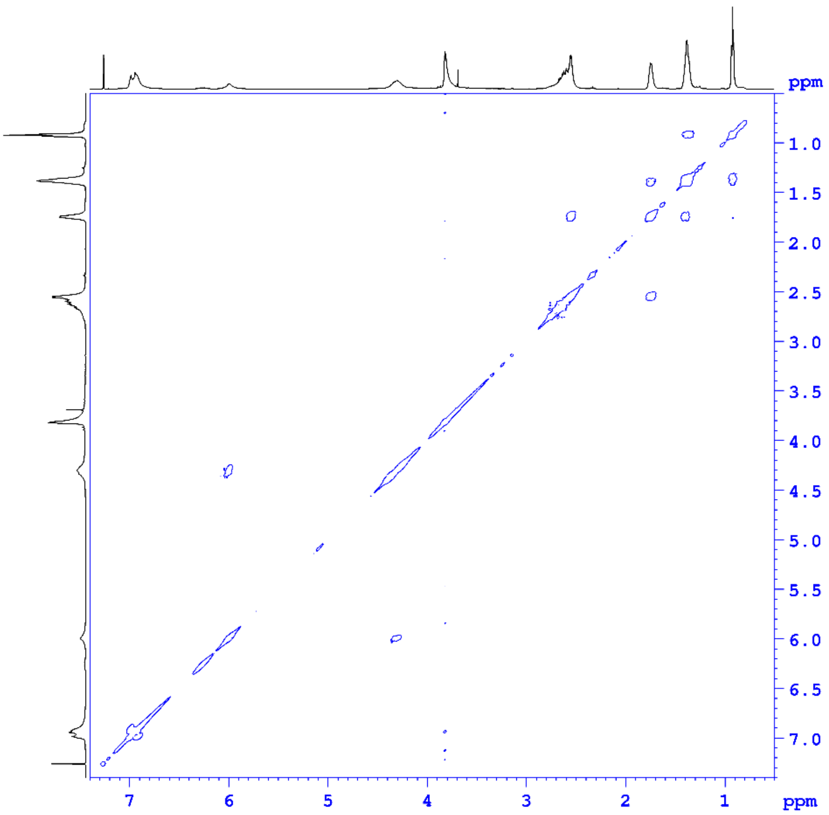

FigureS 90. ^{1}H-^{1}H COSY NMR of P(EGH-*alt*-SA) (600 MHz, rt, $CDCl_3$).

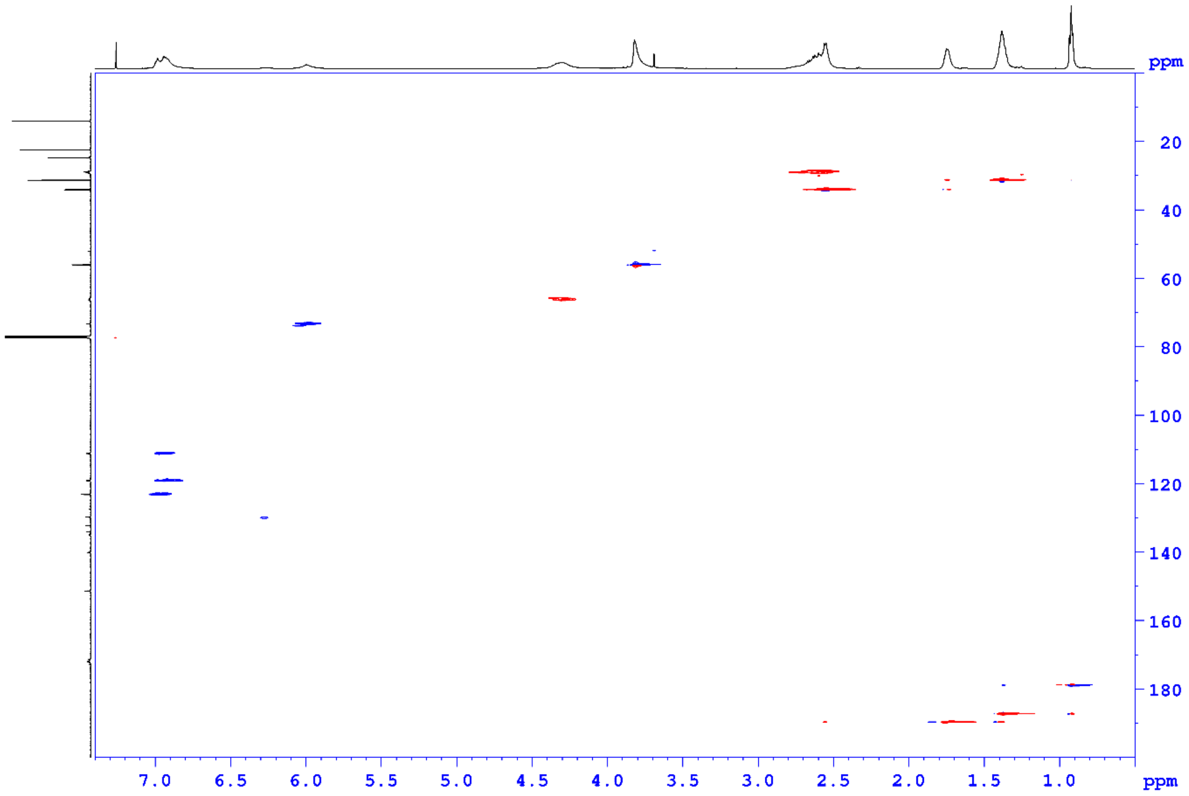

FigureS 91. ^{1}H-^{13}C HSQC NMR of P(EGH-*alt*-SA) (600 MHz, rt, $CDCl_3$).

7. SEC Analyses of Polymers

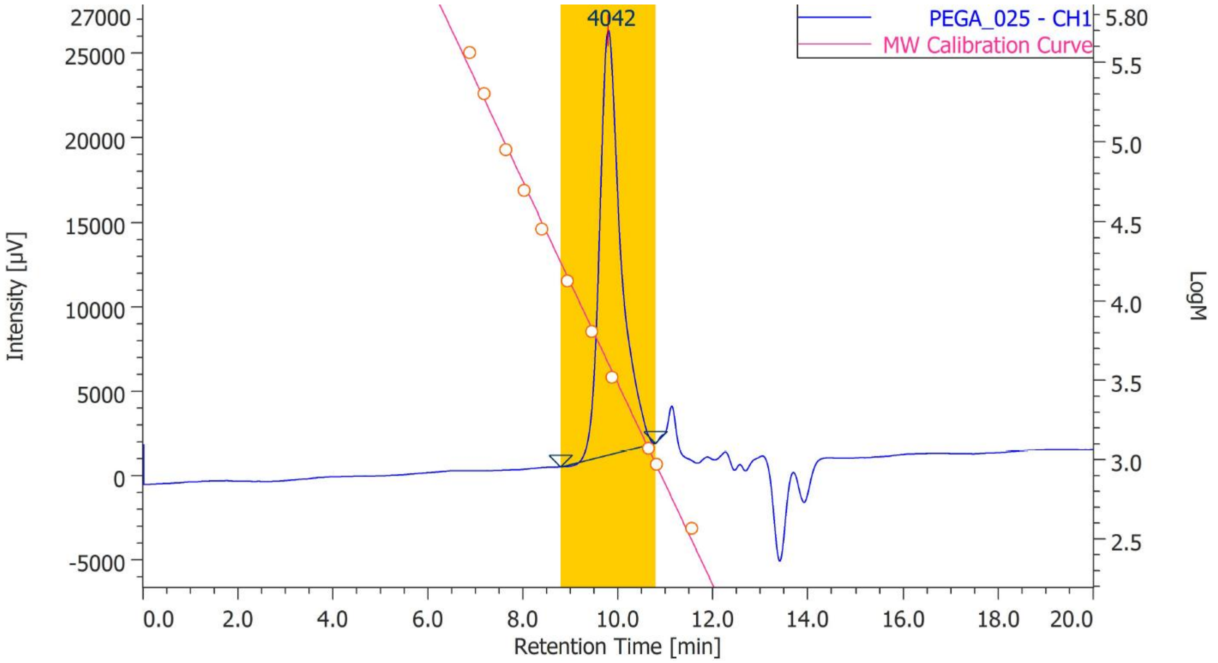

M_p (Da)	M_n (Da)	M_w (Da)	Đ
4042	3321	3770	1.14

FigureS 92. SEC trace of P(EGA-*alt*-PA) prepared as in entry 23, Table S1.

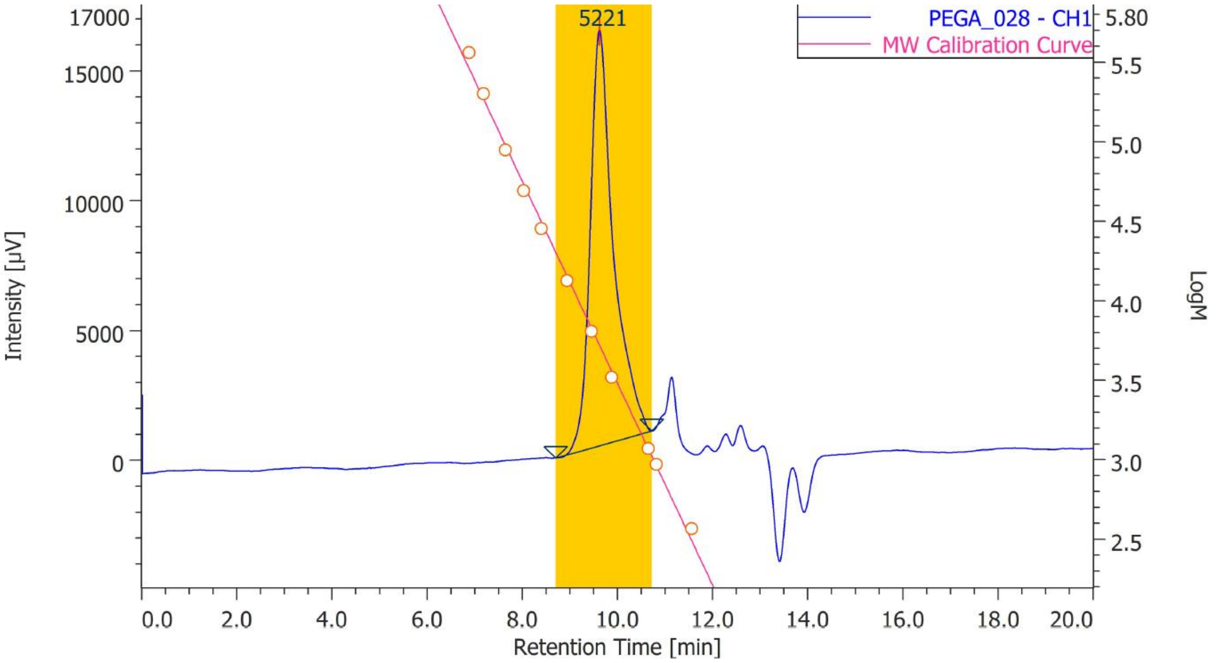

M_p (Da)	M_n (Da)	M_w (Da)	Đ
5221	4088	4820	1.17

FigureS 93. SEC trace of P(EGA-*alt*-PA) prepared as in entry 1, Table 1.

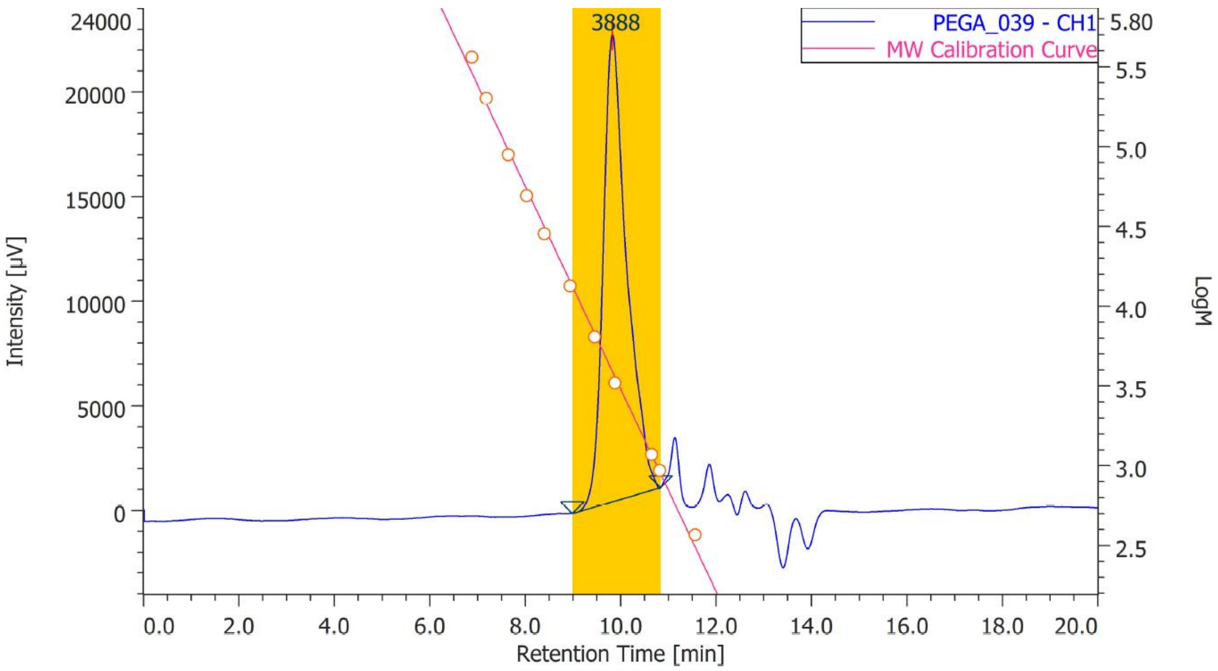

M_p (Da)	M_n (Da)	M_w (Da)	Đ
3888	3117	3586	1.15

FigureS 94. SEC trace of P(EGB-*alt*-PA) prepared as in entry 2, Table 1.

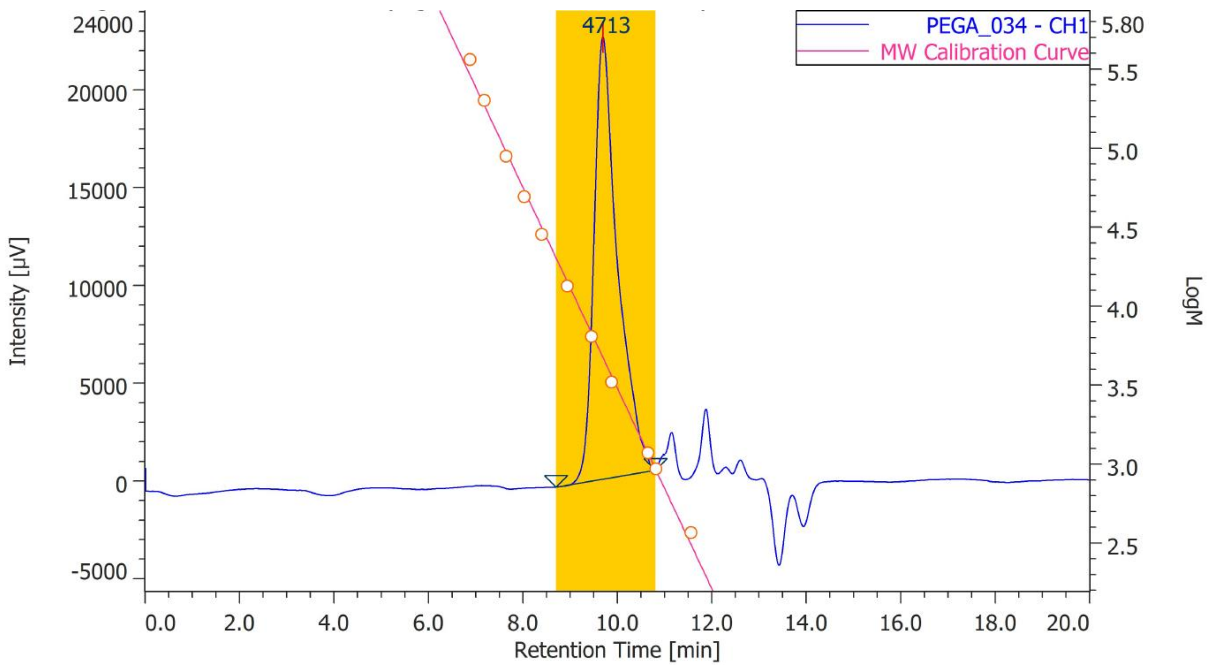

M_p (Da)	M_n (Da)	M_w (Da)	Đ
4713	3620	4243	1.17

FigureS 95. SEC trace of P(EGH-*alt*-PA) prepared as in entry 3, Table 1.

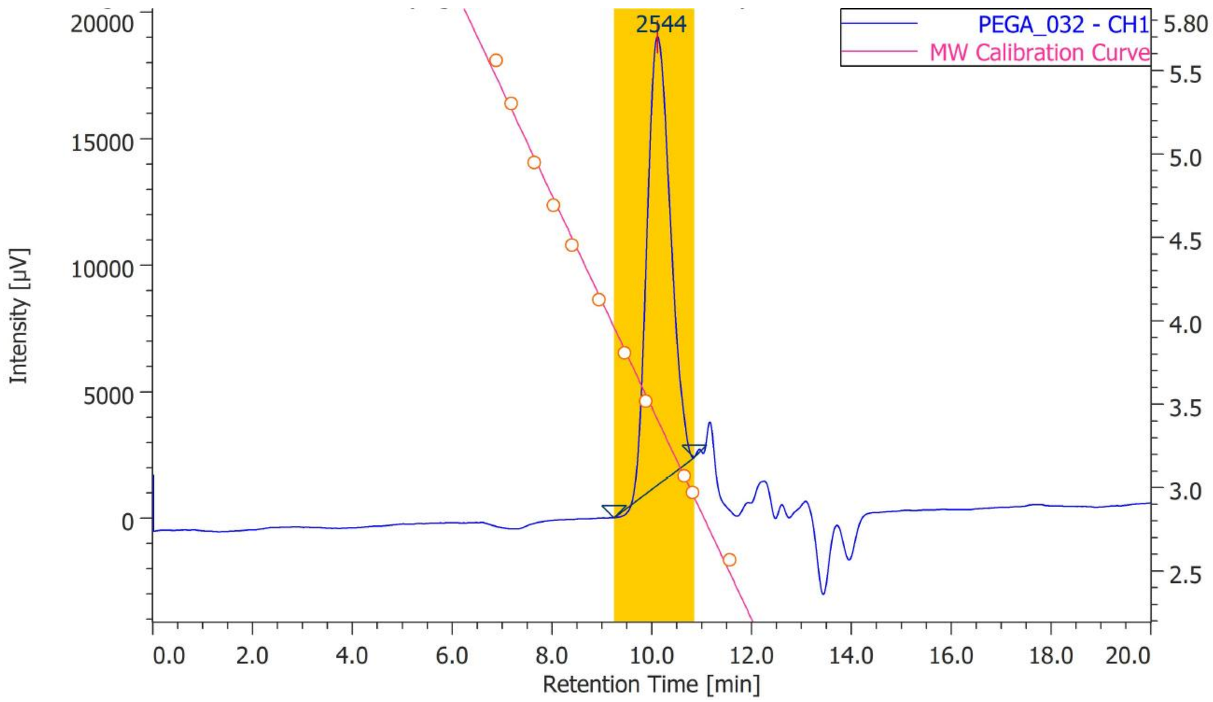

M_p (Da)	M_n (Da)	M_w (Da)	Đ
2544	2276	2504	1.10

FigureS 96. SEC trace of P(EGA-*alt*-THPA) prepared as in entry 4, Table 1.

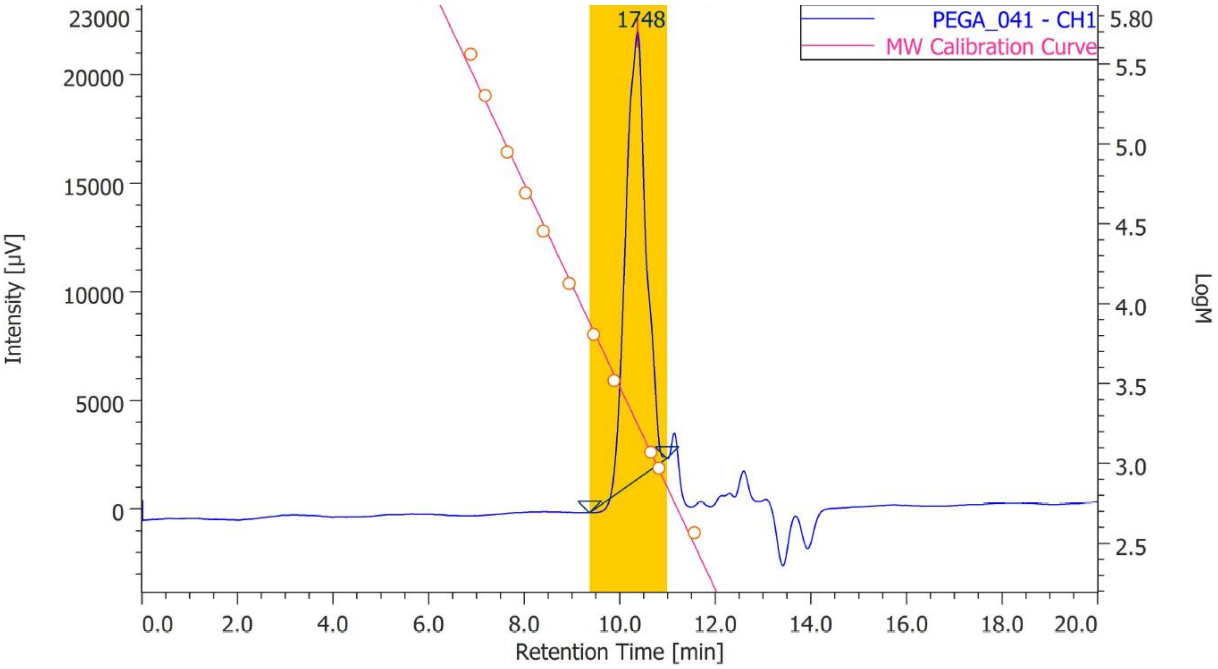

M_p (Da)	M_n (Da)	M_w (Da)	Đ
1748	1753	1902	1.09

FigureS 97. SEC trace of P(EGB-*alt*-THPA) prepared as in entry 5, Table 1.

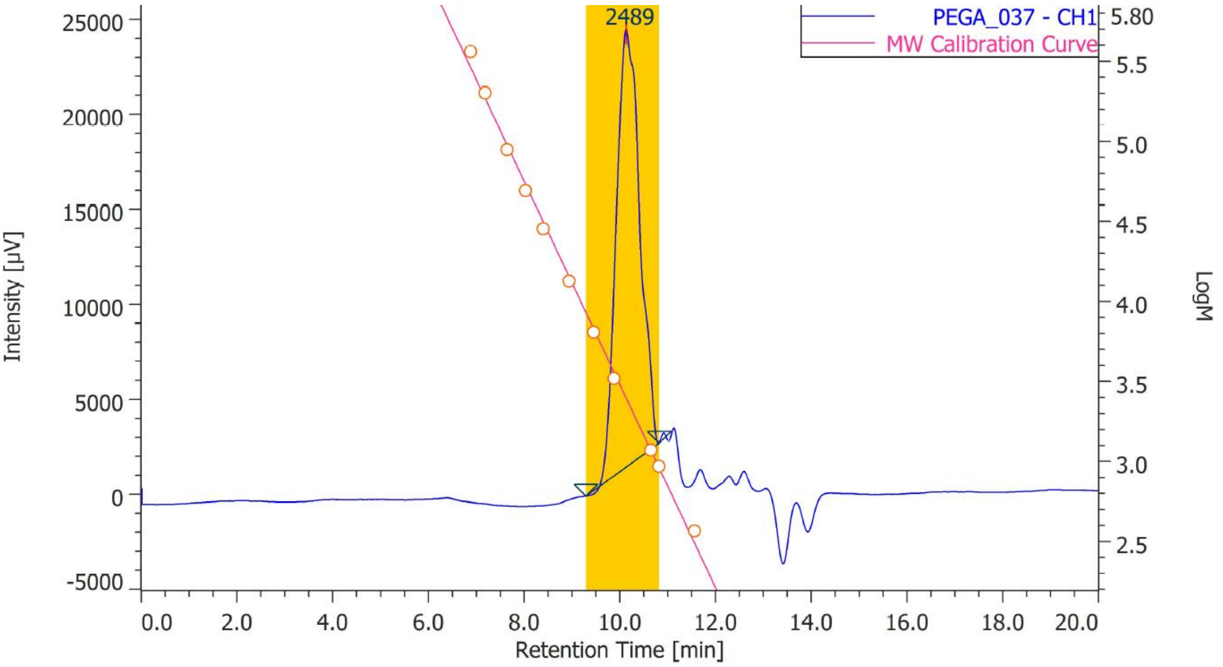

M_p (Da)	M_n (Da)	M_w (Da)	Đ
2489	2181	2414	1.11

FigureS 98. SEC trace of P(EGH-*alt*-THPA) prepared as in entry 6, Table 1.

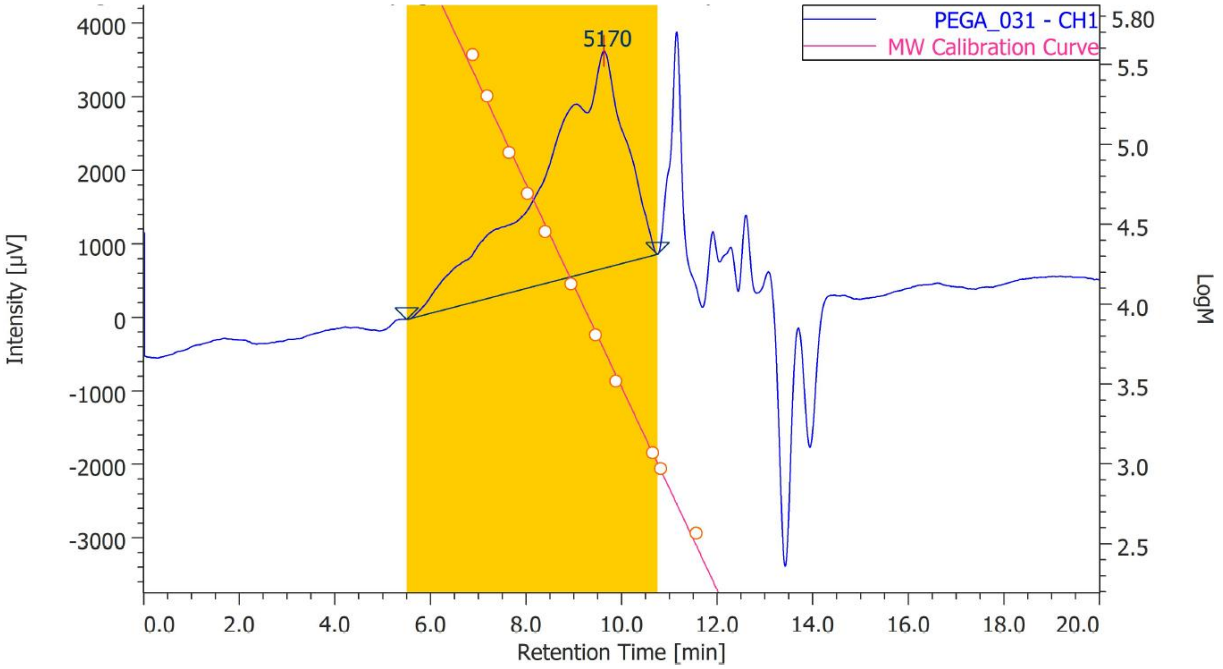

M_p (Da)	M_n (Da)	M_w (Da)	Đ
5170	7421	75706	10.20

FigureS 99. SEC trace of P(EGA-*alt*-MA) prepared as in entry 7, Table 1.

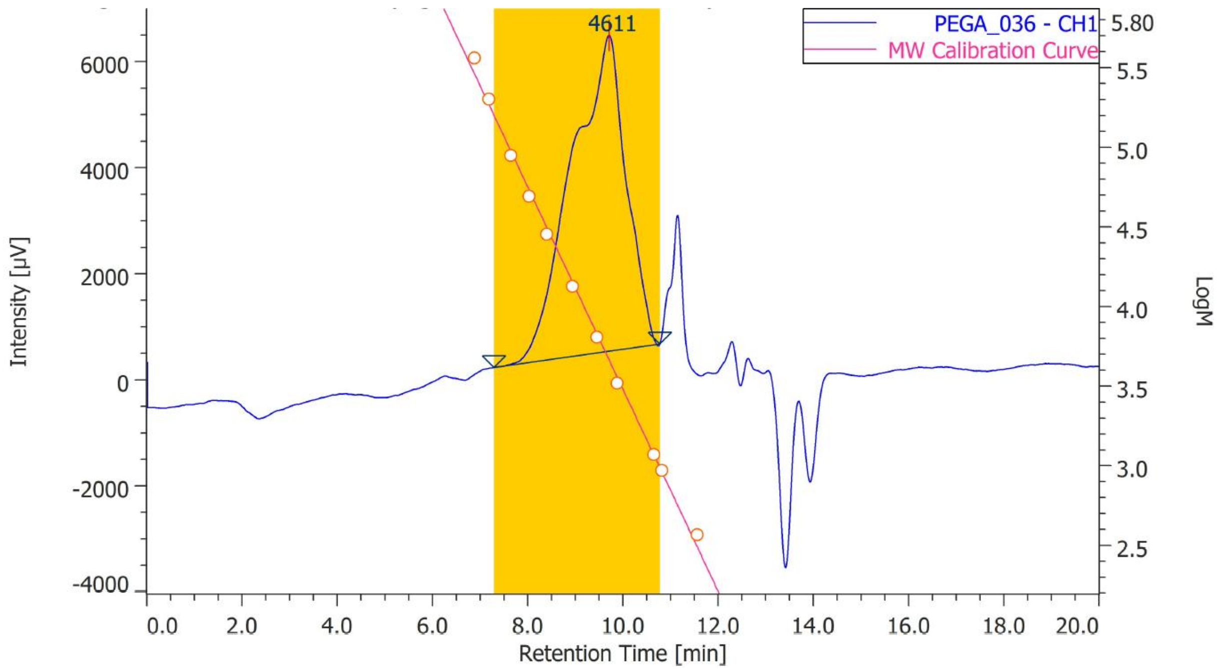

M_p (Da)	M_n (Da)	M_w (Da)	Đ
4611	5304	9878	1.86

FigureS 100. SEC trace of P(EGB-*alt*-MA) prepared as in entry 8, Table 1.

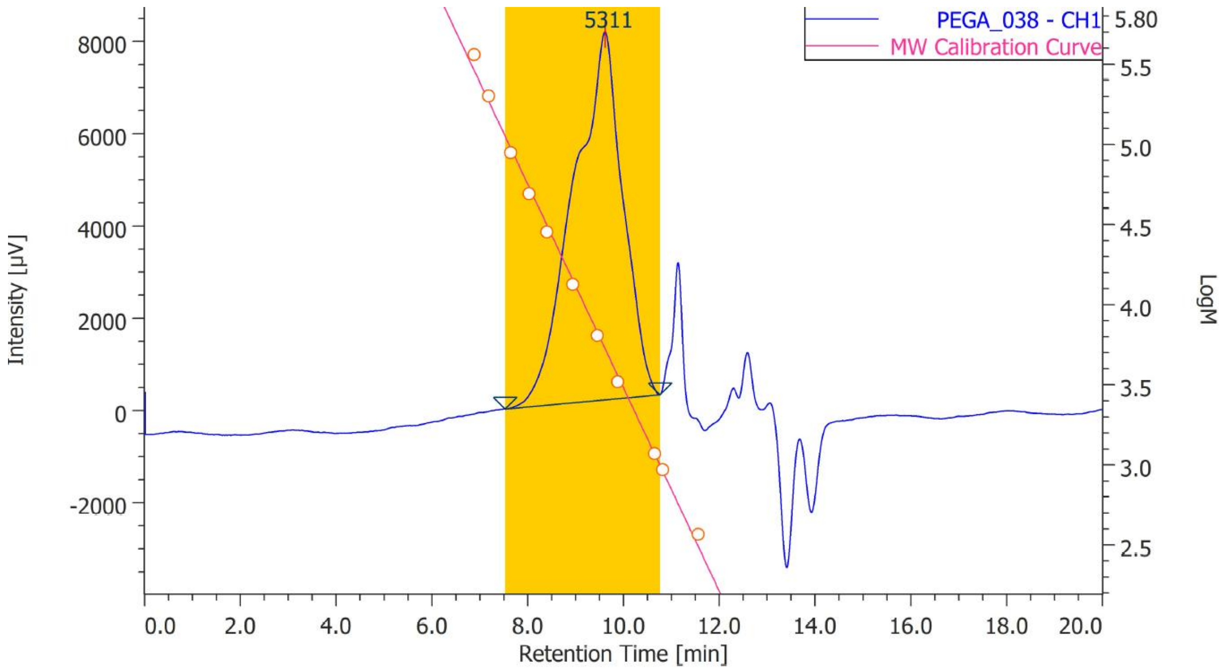

M_p (Da)	M_n (Da)	M_w (Da)	Đ
5311	5519	9602	1.74

FigureS 101. SEC trace of P(EGH-*alt*-MA) prepared as in entry 9, Table 1.

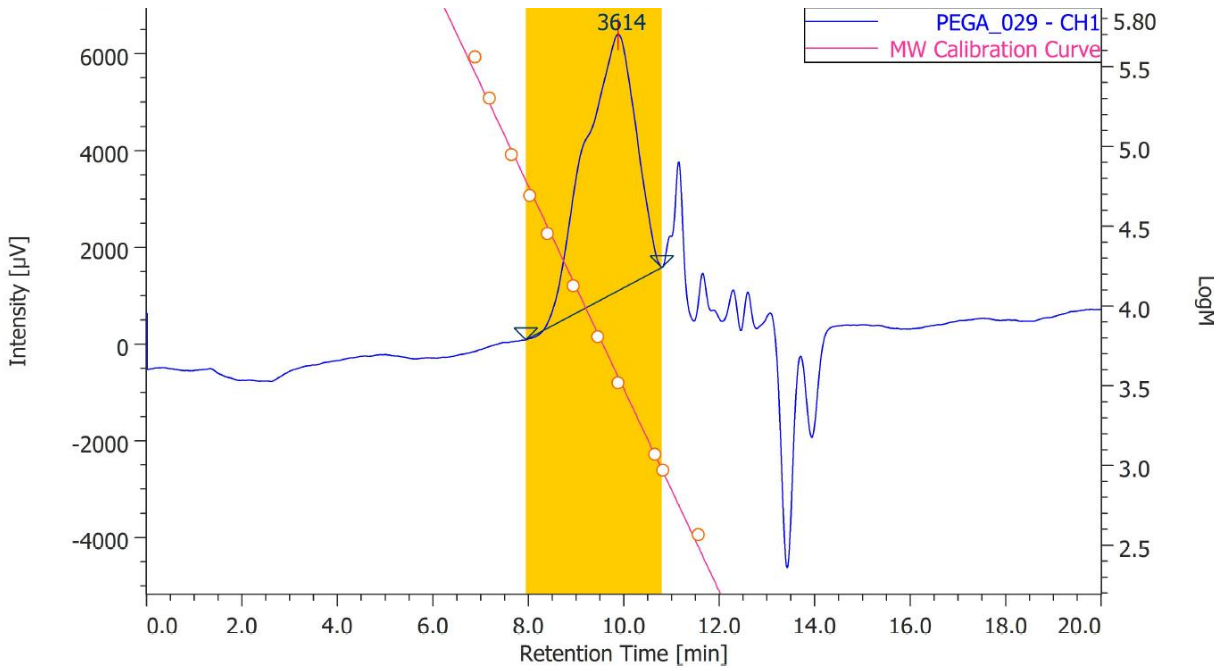

M_p (Da)	M_n (Da)	M_w (Da)	Đ
3614	3990	6446	1.62

FigureS 102. SEC trace of P(EGA-*alt*-SA) prepared as in entry 10, Table 1.

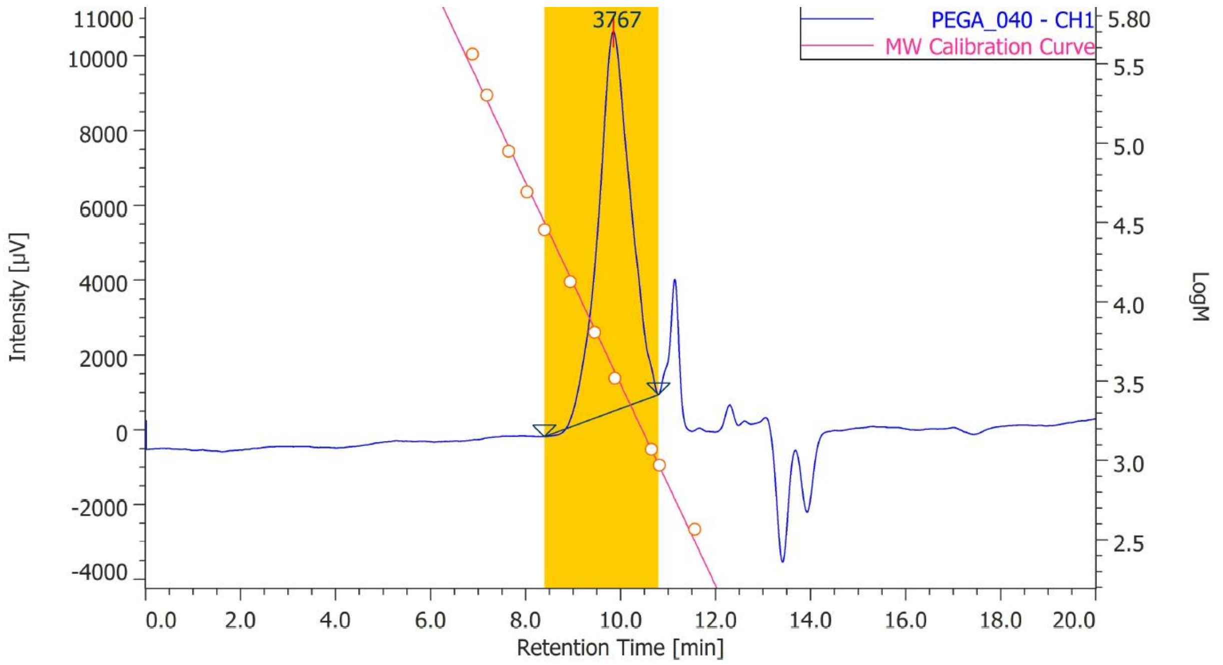

M_p (Da)	M_n (Da)	M_w (Da)	Đ
3767	3244	4106	1.27

FigureS 103. SEC trace of P(EGB-*alt*-SA) prepared as in entry 11, Table 1.

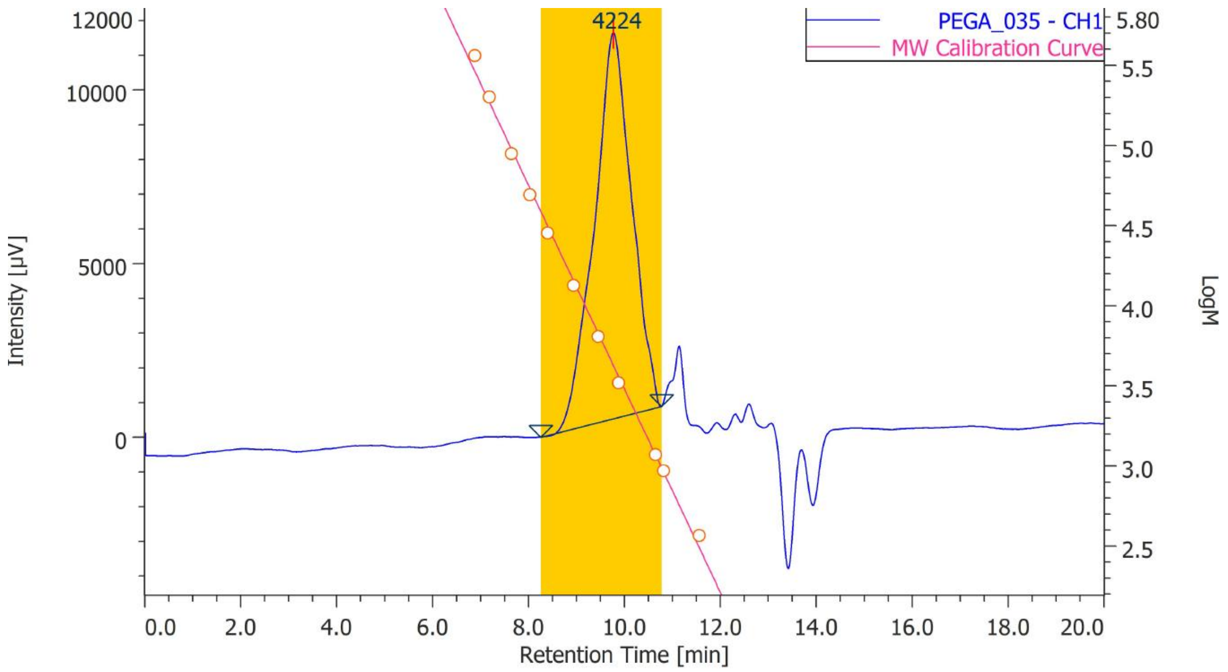

M_p (Da)	M_n (Da)	M_w (Da)	Đ
4224	3720	5051	1.36

FigureS 104. SEC trace of P(EGH-*alt*-SA) prepared as in entry 12, Table 1.

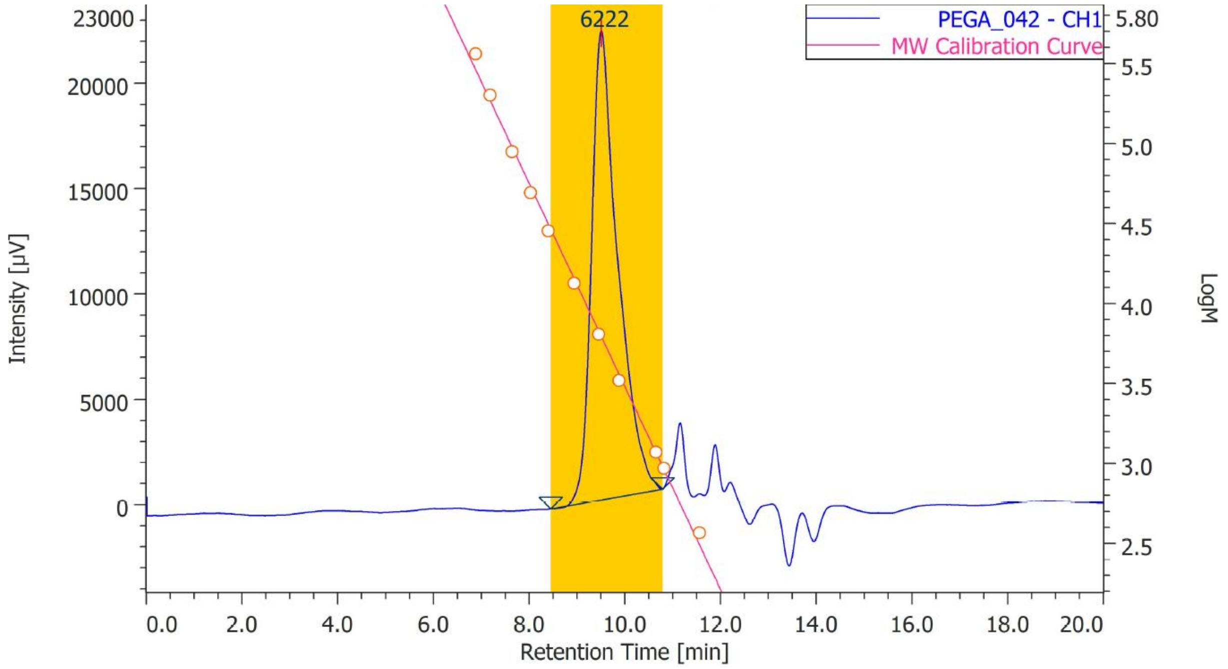

M_p (Da)	M_n (Da)	M_w (Da)	Đ
6222	4568	5525	1.21

FigureS 105. SEC trace of P(EGH-*alt*-PA) prepared as in entry 10, Table 1.

8. HR-MALDI FT-ICR MS analyses of polymers

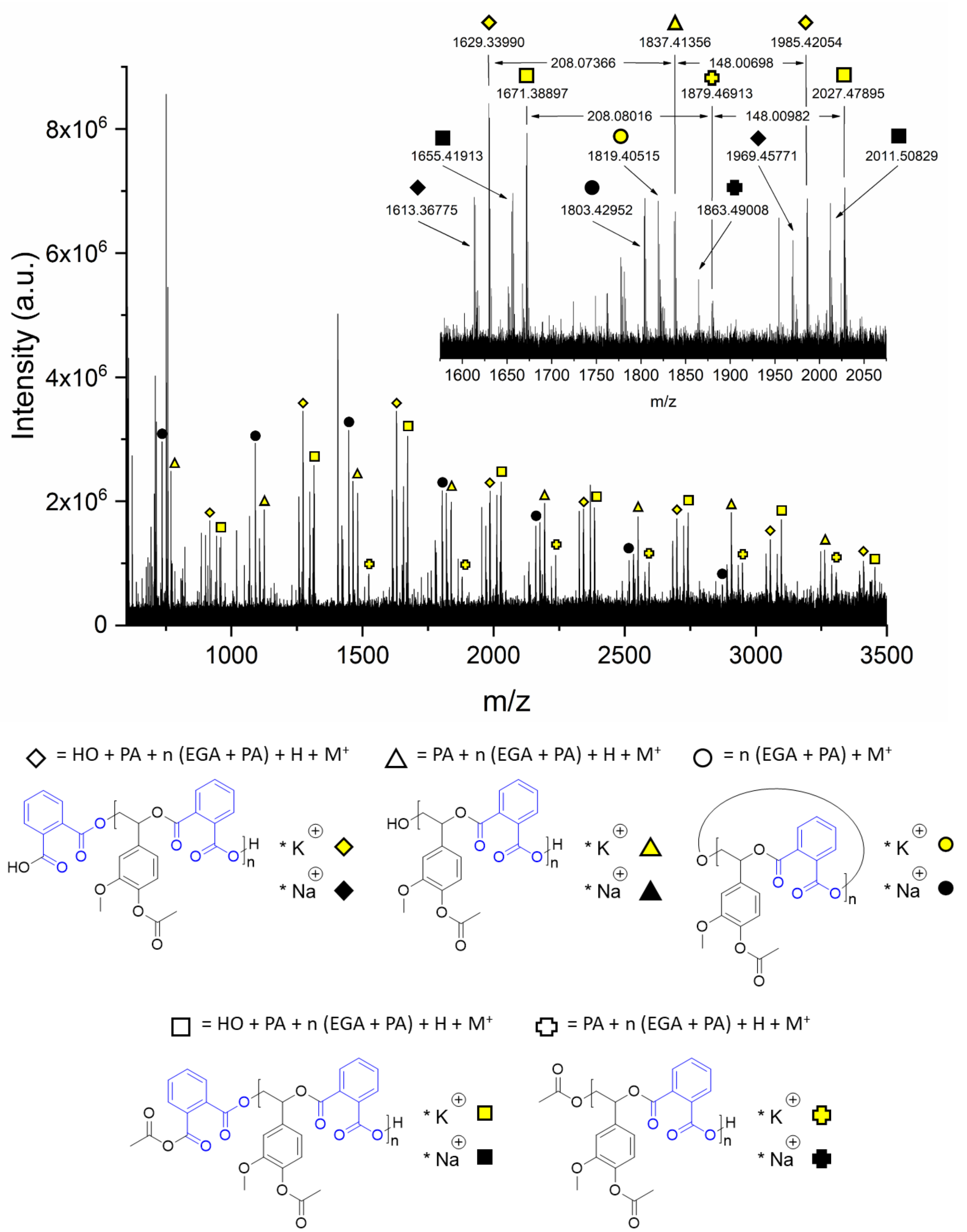

FigureS 106. High Resolution MALDI spectrum of P(EGA-*alt*-PA) as obtained in entry 1, Table S1. a) Region from 600 to 3500 m/z. b) Region from 1575 to 2075 m/z. Symbols ◇, △, ○, □, and ✠ indicate the species at the bottom. Sodium cations are labeled only in insert (b) for clarity.

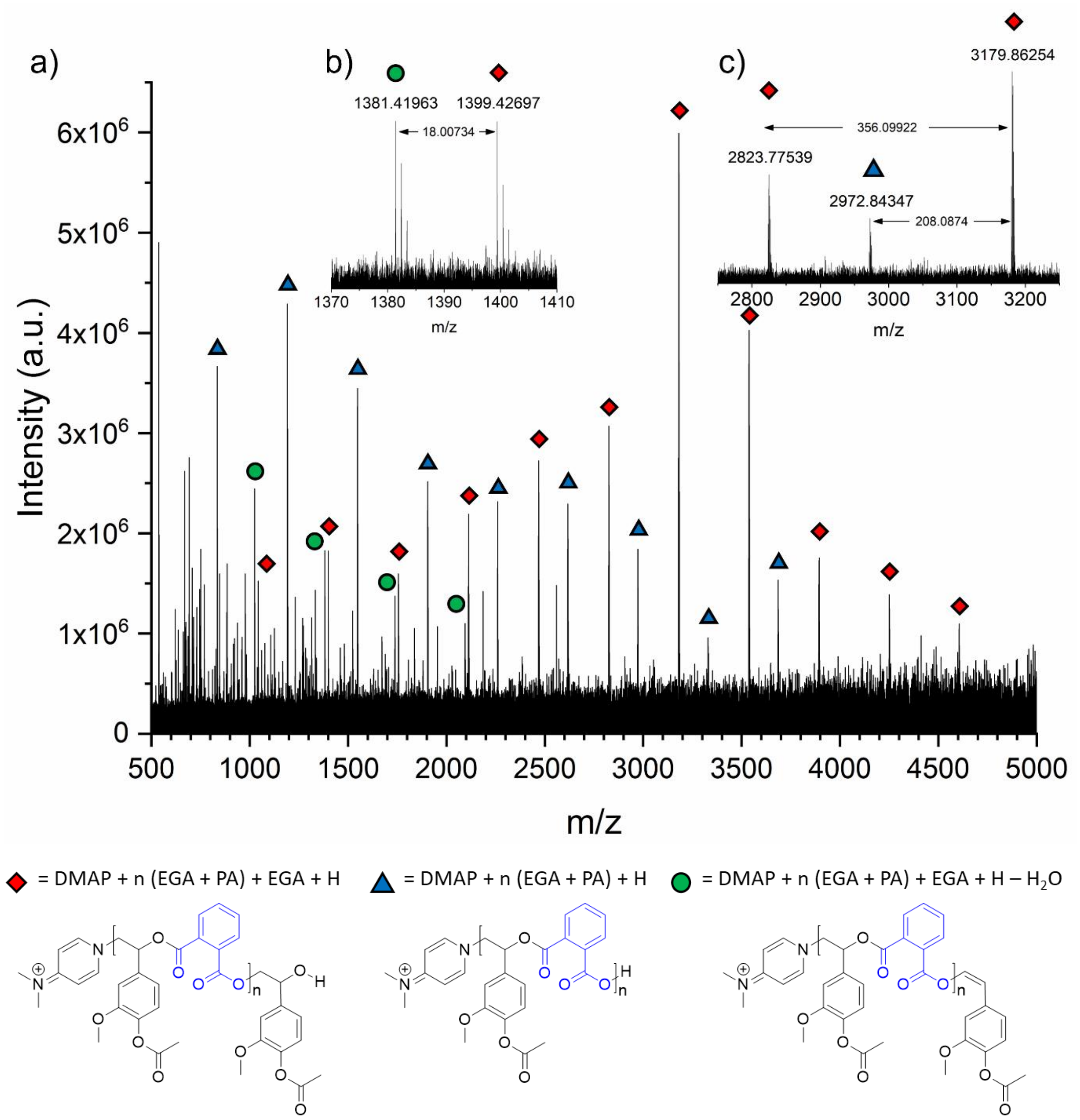

FigureS 107. High Resolution MALDI spectrum of P(EGA-*alt*-PA) as obtained in entry 2, Table S1. a) Region from 500 to 5000 m/z. b) Region from 1370 to 1410 m/z. c) Region from 2700 to 3300 m/z. Symbols ◇, △, and ○, indicate the species at the bottom.

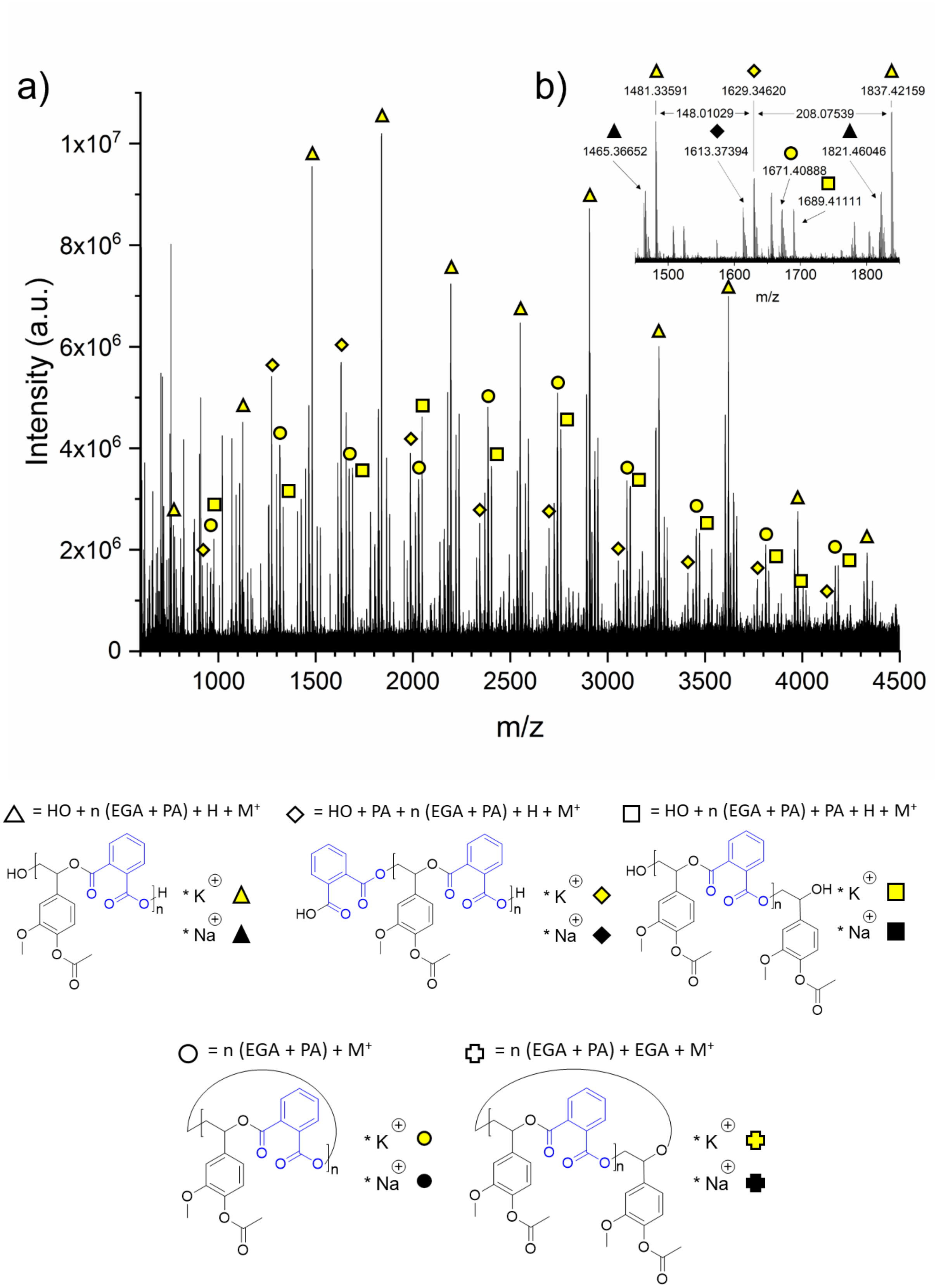

FigureS 108. High Resolution MALDI spectrum of P(EGA-*alt*-PA) as obtained in entry 3, Table S1. a) Region from 600 to 4500 m/z. b) Region from 1450 to 1850 m/z. Symbols ◇, △, □ and ○ indicate the species at the bottom. Sodium cations are labeled only in insert (b) for clarity.

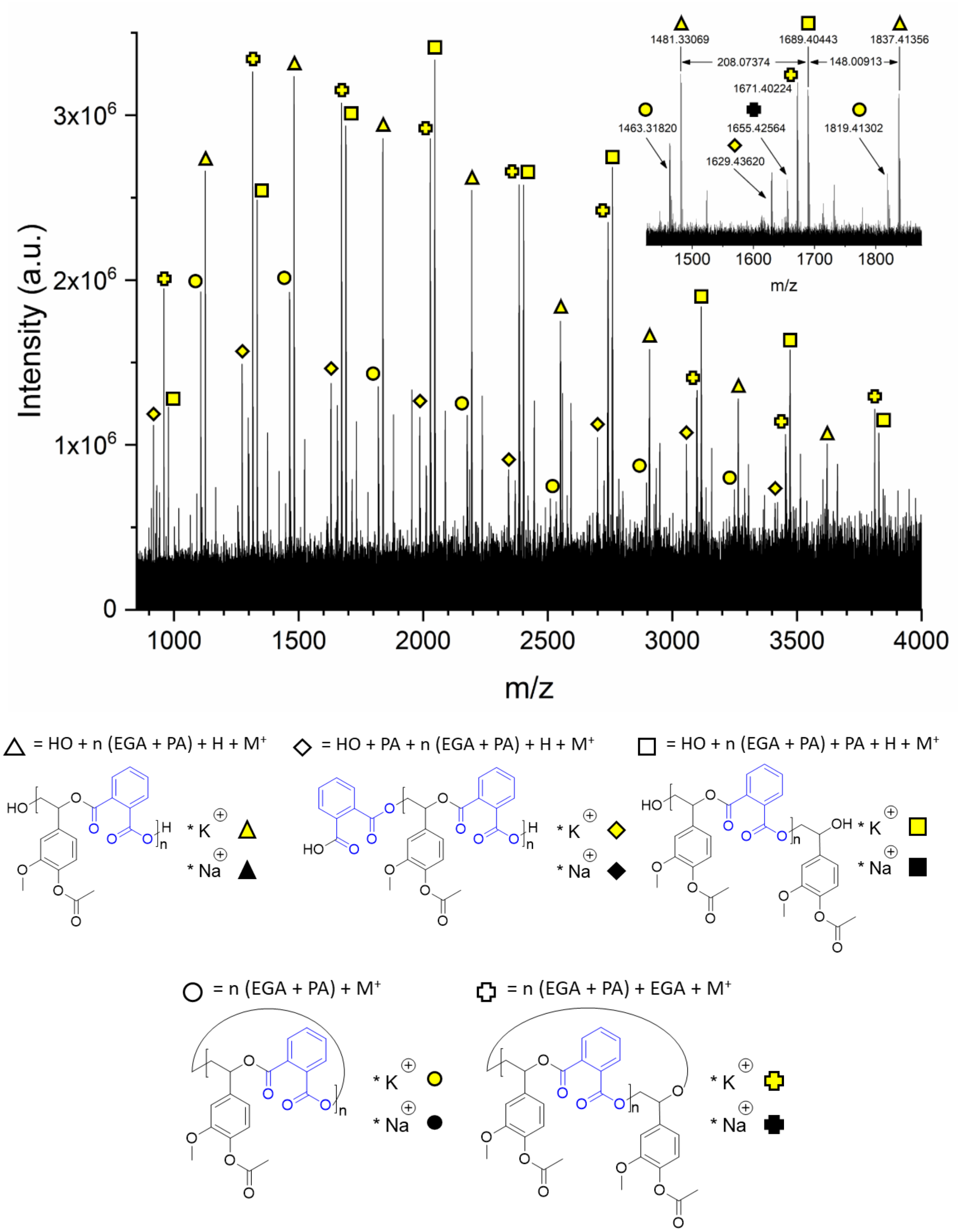

FigureS 109. High Resolution MALDI spectrum of P(EGA-*alt*-PA) as obtained in entry 4, Table S1. a) Region from 1425 to 1875 m/z. Symbols ◇, △, ○, □, and ✚ indicate the species at the bottom. Sodium cations are labeled only in insert (b) for clarity.

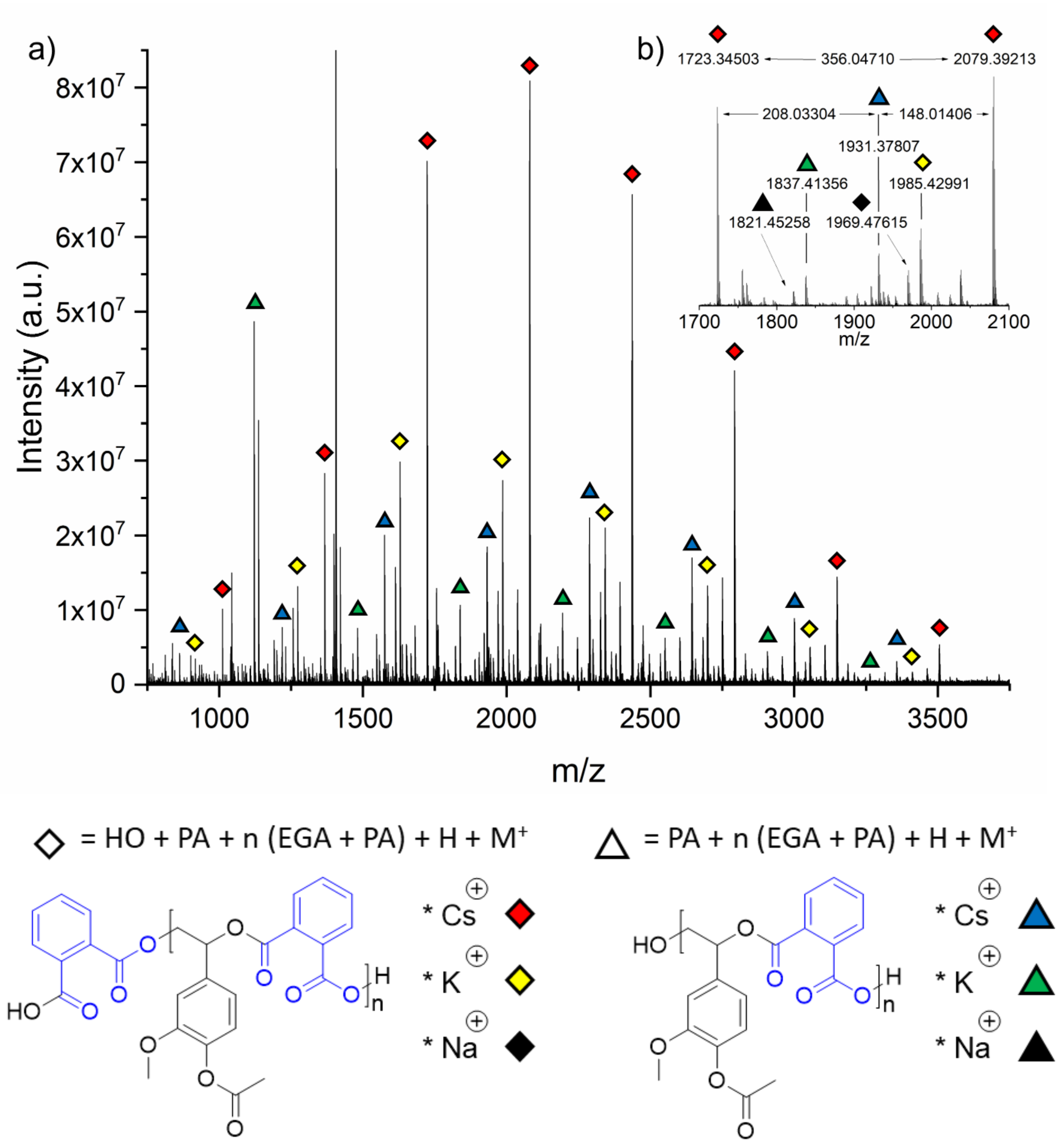

FigureS 110. High Resolution MALDI spectrum of P(EGA-*alt*-PA) as obtained in entry 9, Table S1. a) Region from 350 to 3750 m/z. b) Region from 1700 to 2100 m/z. Symbols ◇ and △ indicate the species at the bottom. Sodium cations are labeled only in insert (b) for clarity.

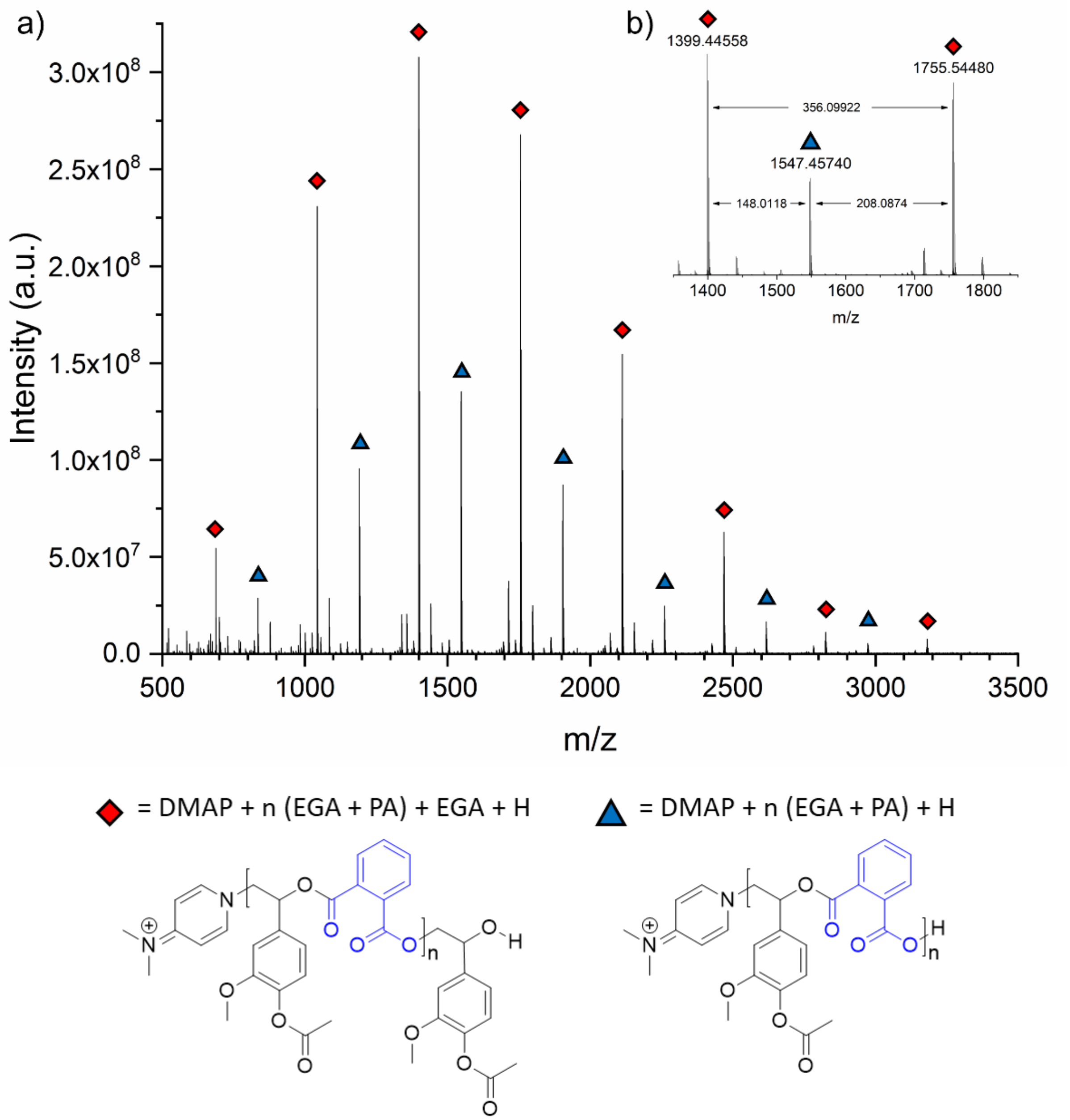

FigureS 111. High Resolution MALDI spectrum of P(EGA-*alt*-PA) as obtained in entry 10, Table S1. a) Region from 500 to 3500 m/z. b) Region from 1350 to 1850 m/z. Symbols ◇, and △ indicate the species at the bottom.

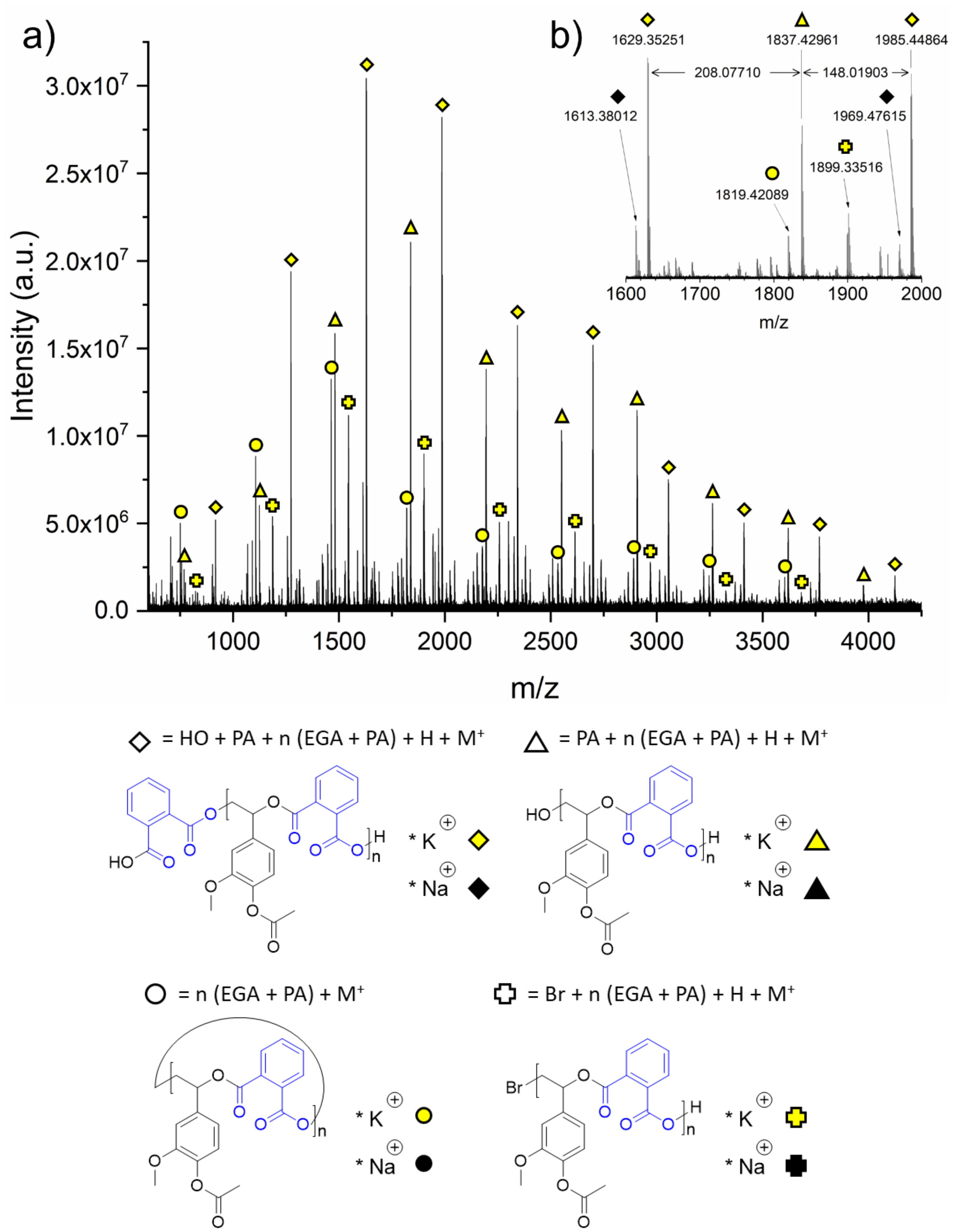

FigureS 112. High Resolution MALDI spectrum of P(EGA-*alt*-PA) as obtained in entry 11, Table S1. a) Region from 600 to 4250 m/z. b) region from 1600 to 2000 m/z. Symbols ◇, △, ○, and ✚ indicate the species at the bottom. Sodium cations are labeled only in insert (b) for clarity.

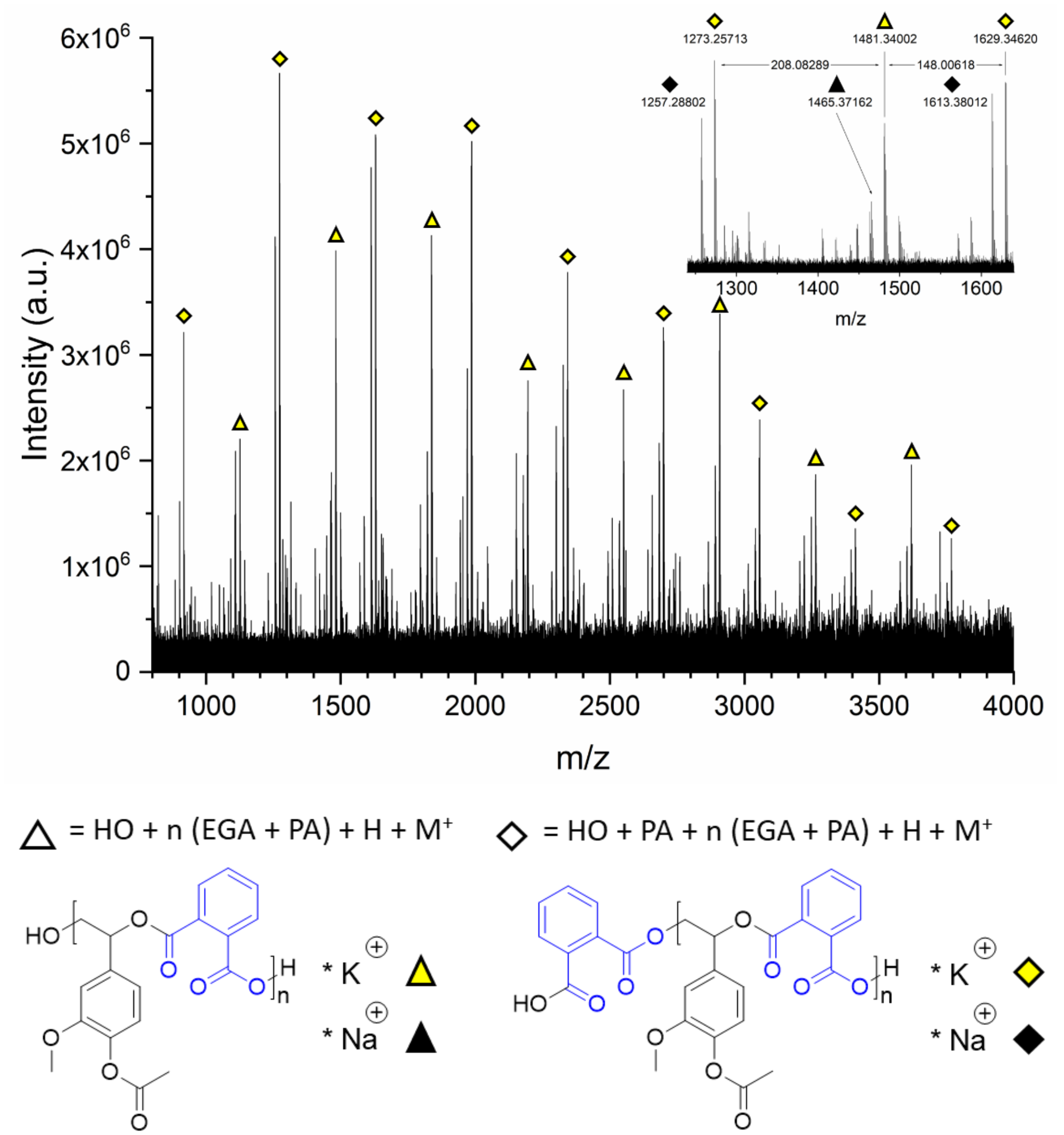

FigureS 113. High Resolution MALDI spectrum of P(EGA-*alt*-PA) as obtained in entry 12, Table S1. a) Region from 1240 to 1640 m/z. Symbols ◇, and △ indicate the species at the bottom. Sodium cations are labeled only in insert (b) for clarity.

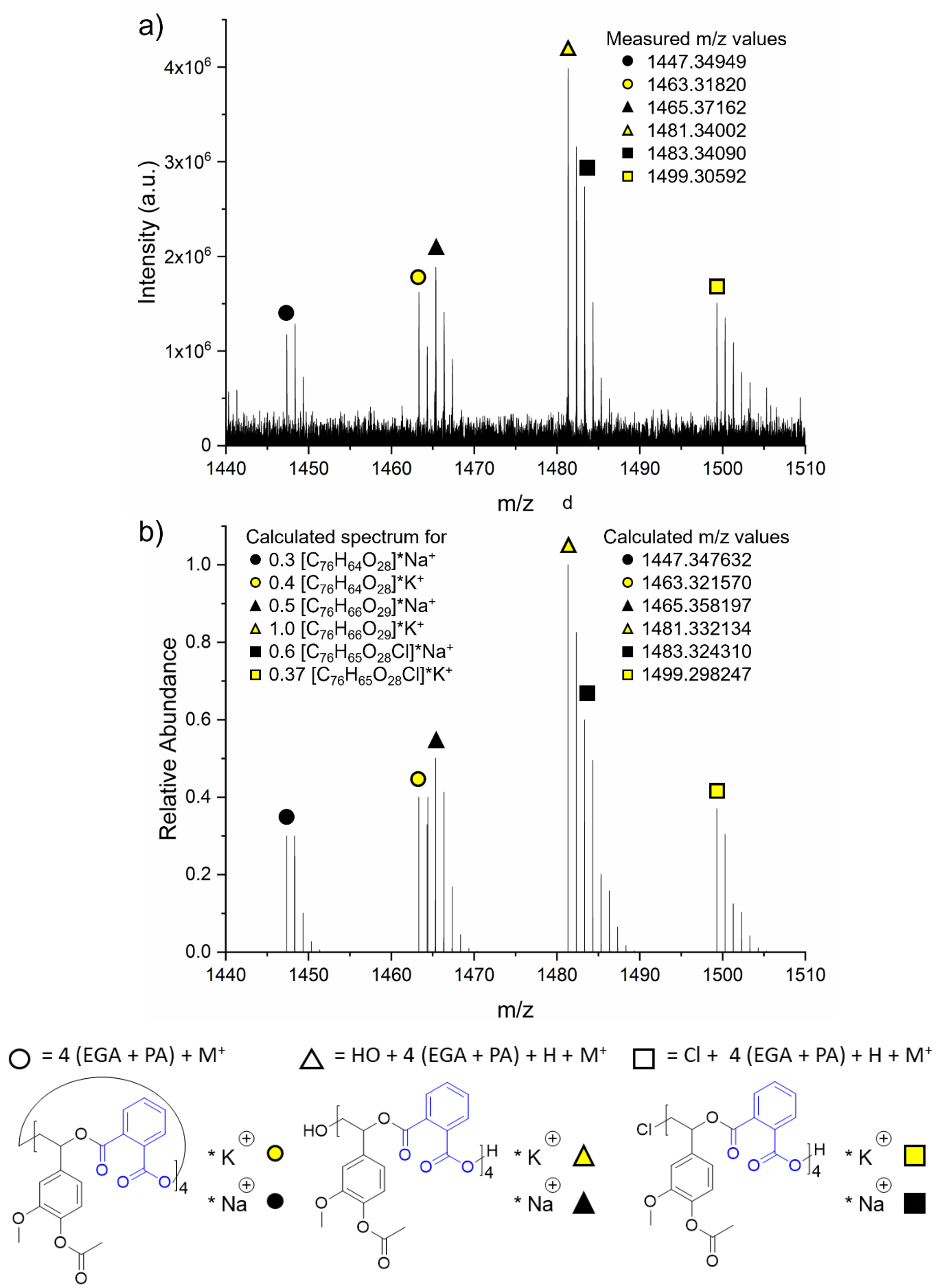

FigureS 114. Comparison between High Resolution MALDI spectrum of P(EGA-*alt*-PA) as obtained in entry 12, Table S1 in the region from 1440 to 1510 m/z (a), and calculated spectrum for the species at the bottom indicated with symbols ○, △, and □ (b). Measured and calculated isotopic peaks values are reported in the captions.

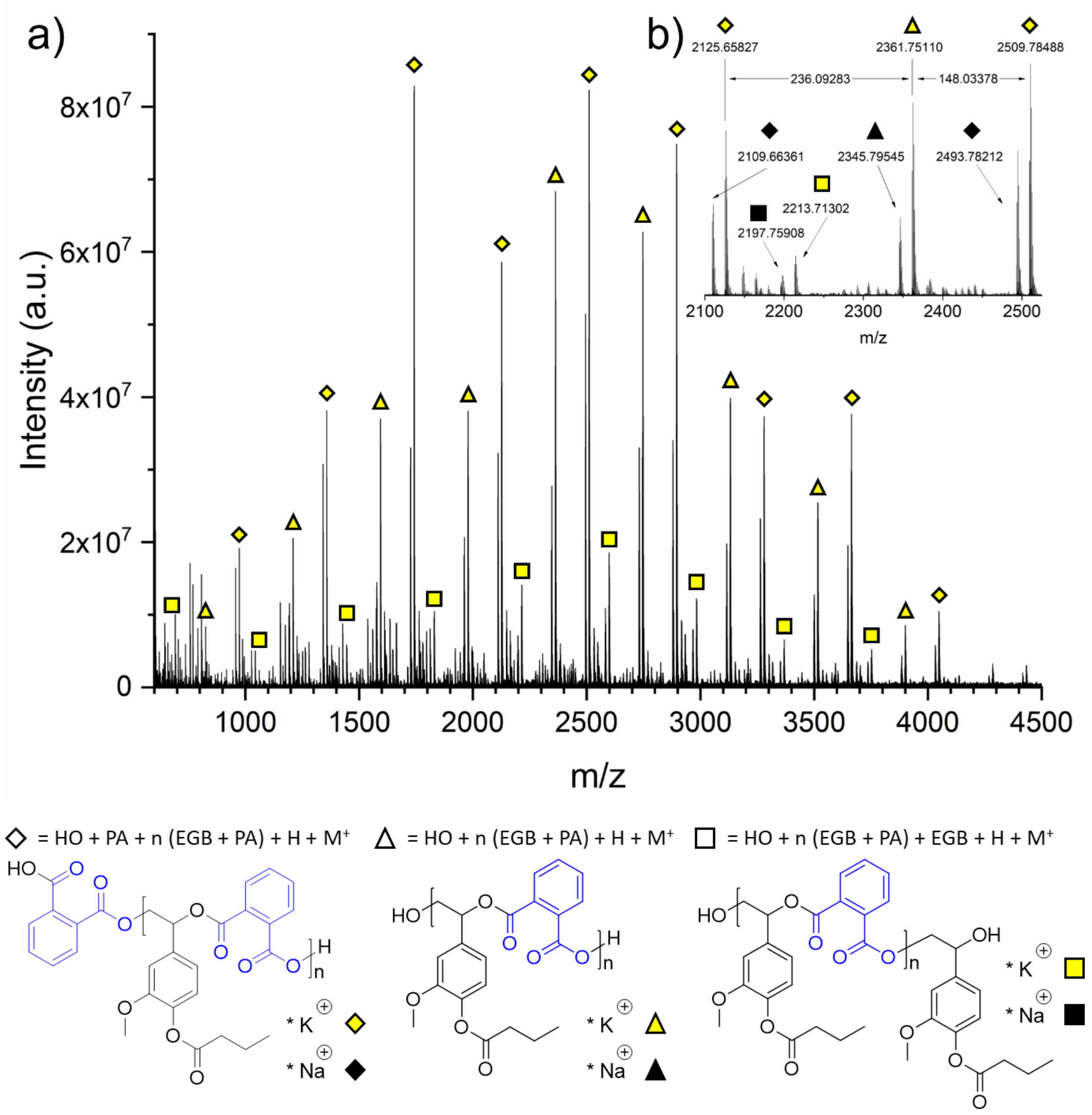

FigureS 115. High Resolution MALDI spectrum of P(EGB-*alt*-PA) as obtained in entry 2, Table 1. a) Region from 600 to 4500 m/z. b) Region from 2100 to 2550 m/z. Symbols ◇, △, and □ indicate the species at the bottom. Sodium cations are labeled only in insert (b) for clarity.

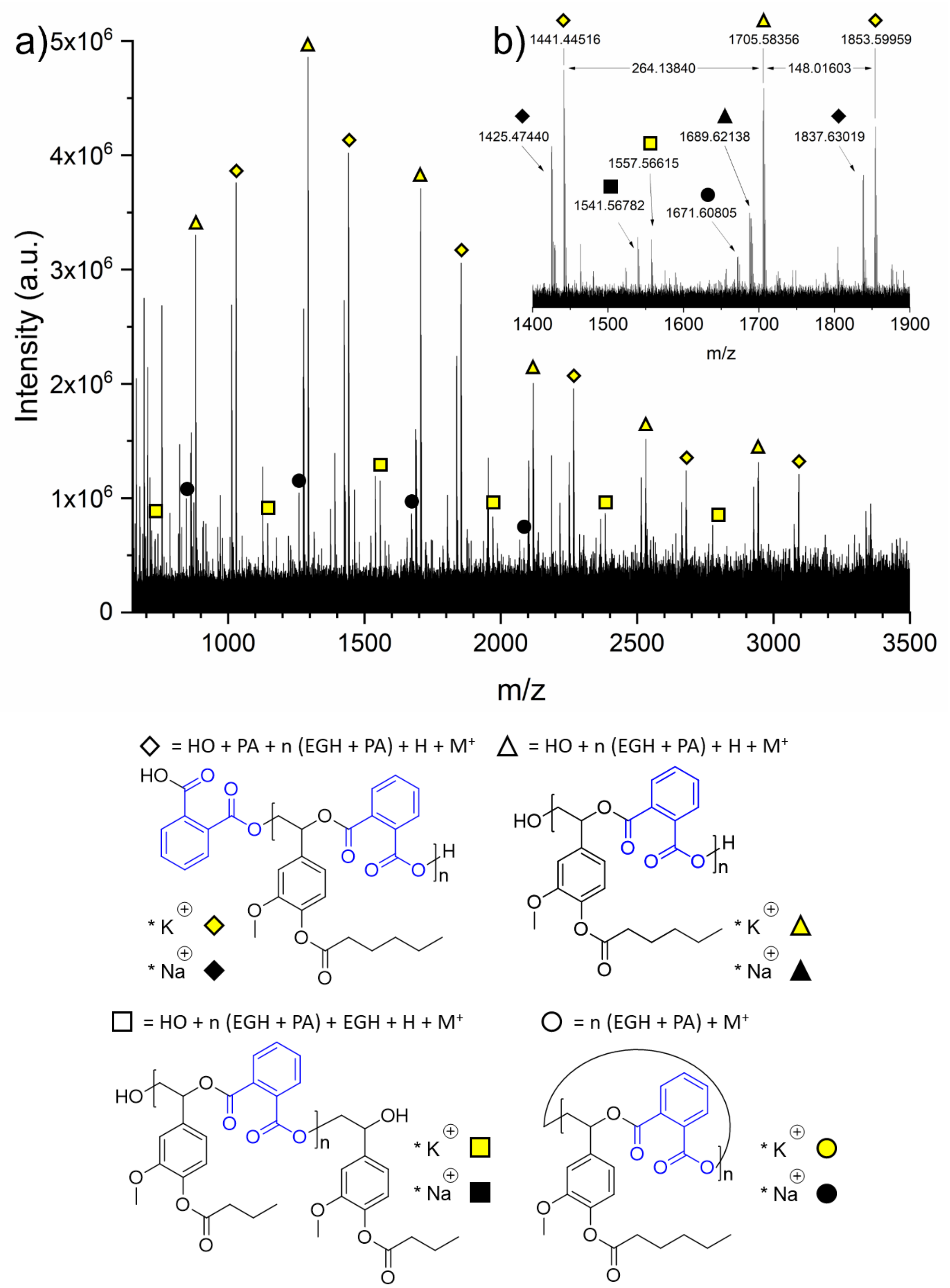

FigureS 116. High Resolution MALDI spectrum of P(EGH-*alt*-PA) as obtained in entry 3, Table 1. a) Region from 650 to 3500 m/z. b) Region from 1400 to 1900 m/z. Symbols ◇, △, □, and ○ indicate the species at the bottom.

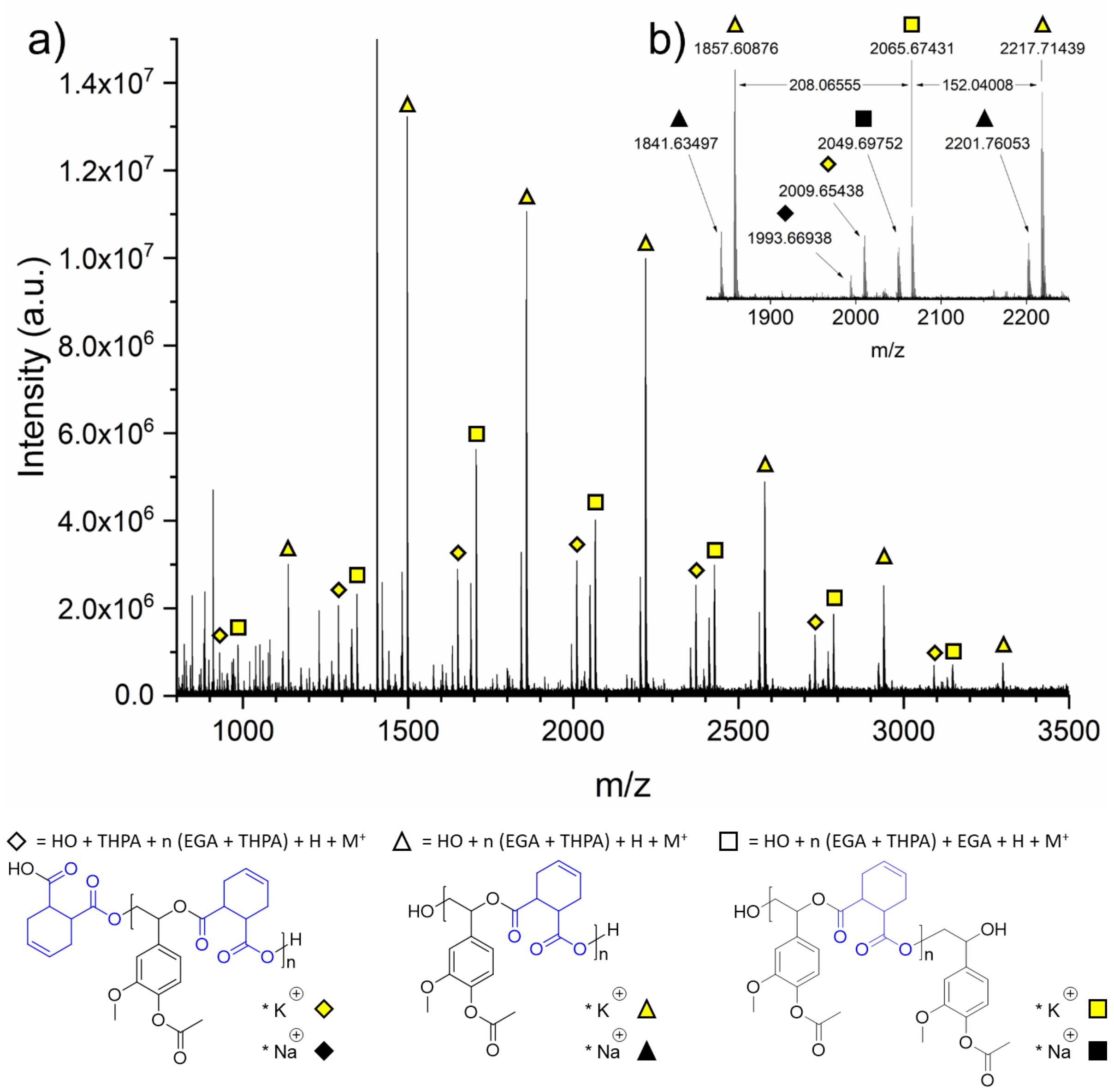

FigureS 117. High Resolution MALDI spectrum of P(EGA-*alt*-THPA) as obtained in entry 4, Table 1. a) Region from 800 to 3500 m/z. b) Region from 1825 to 2050 m/z. Symbols ◇, △, and □ indicate the species at the bottom. Sodium cations are labeled only in insert (b) for clarity.

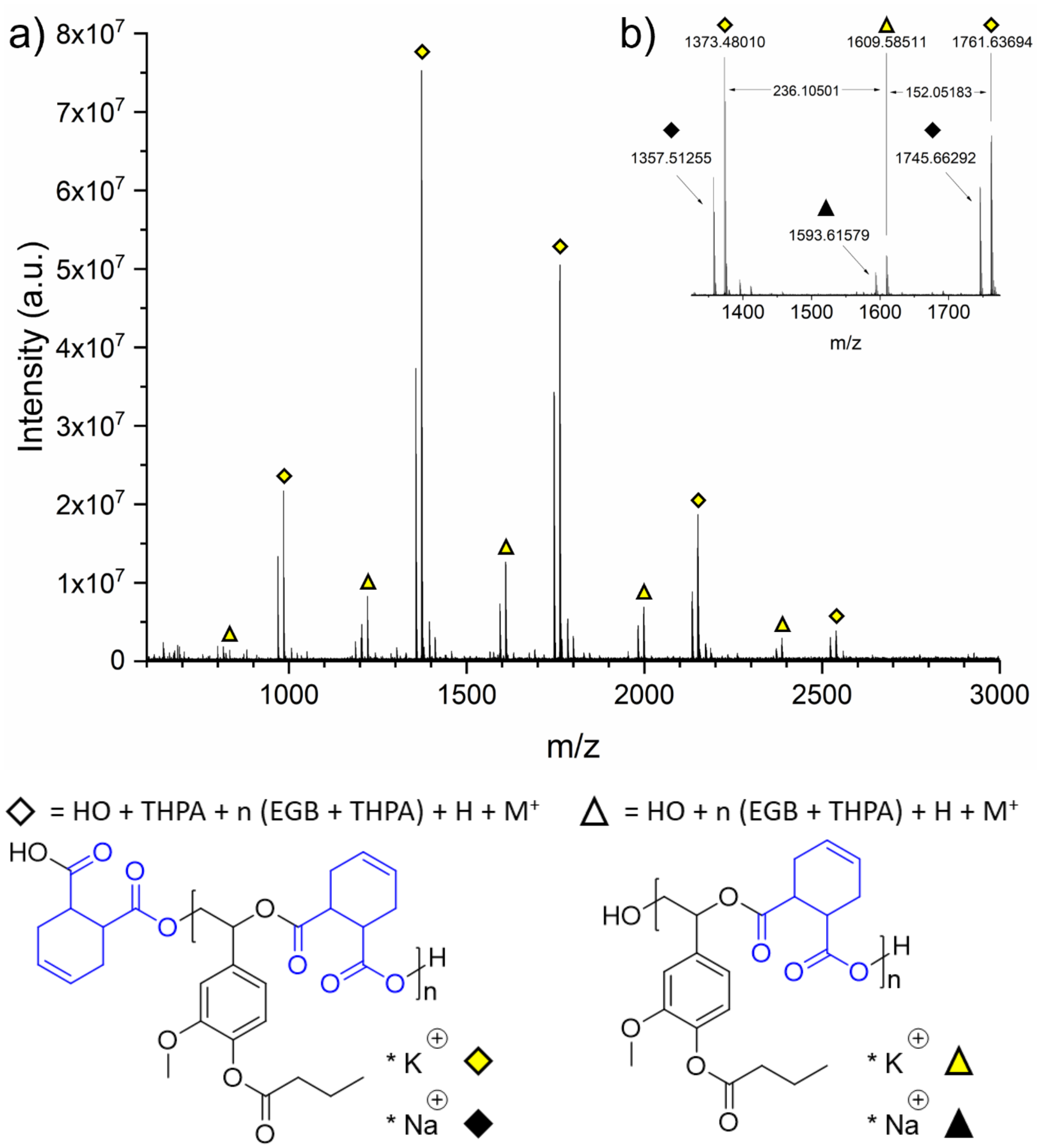

FigureS 118. High Resolution MALDI spectrum of P(EGB-*alt*-THPA) as obtained in entry 5, Table 1. a) Region from 600 to 3000 m/z. b) Region from 1325 to 1775 m/z. Symbols ◇, and △ indicate the species at the bottom. Sodium cations are labeled only in insert (b) for clarity.

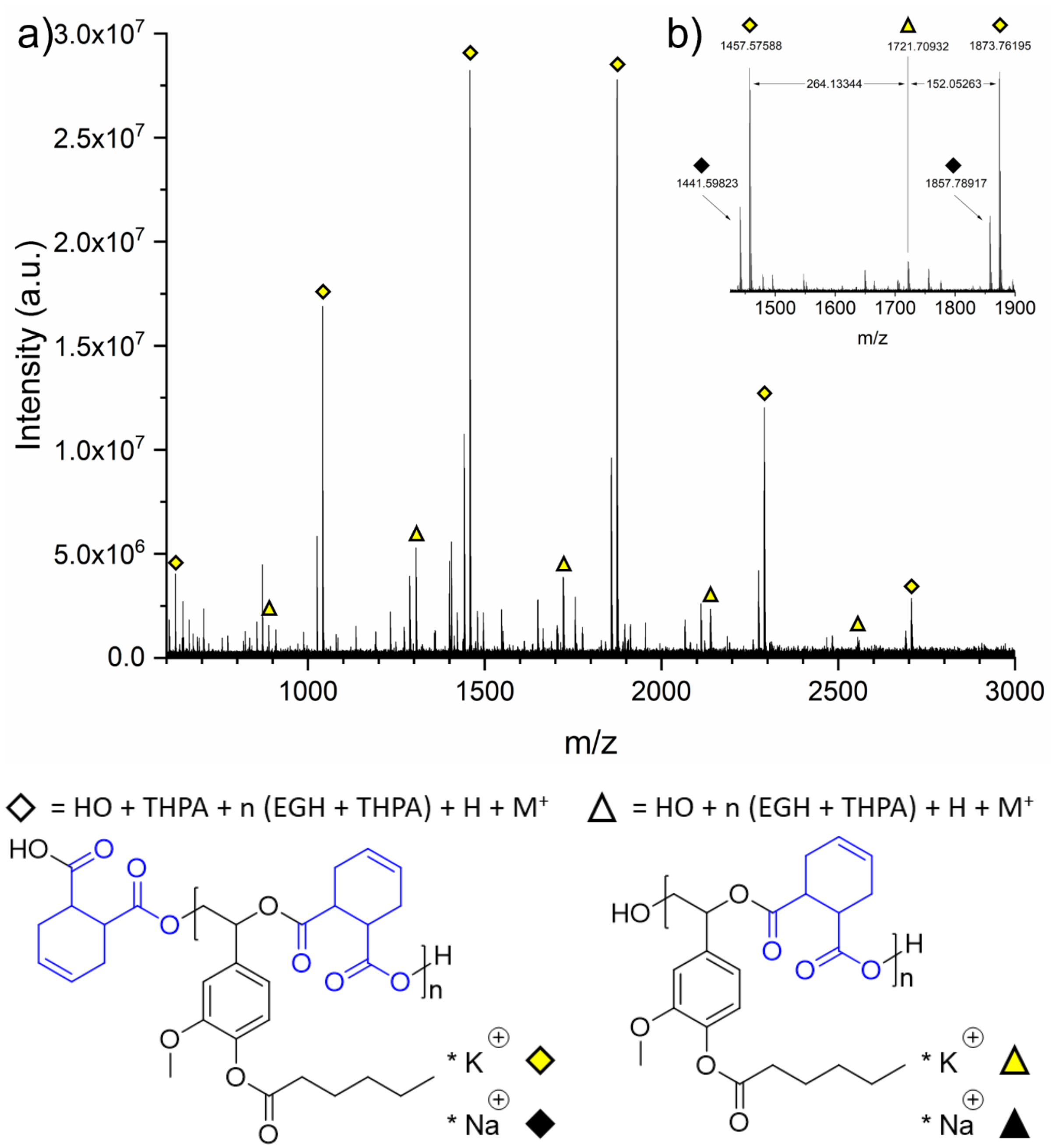

FigureS 119. High Resolution MALDI spectrum of P(EGH-*alt*-THPA) as obtained in entry 6, Table 1. a) Region from 600 to 3000 m/z. b) Region from 1425 to 1900 m/z. Symbols ◇, and △ indicate the species at the bottom. Sodium cations are labeled only in insert (b) for clarity.

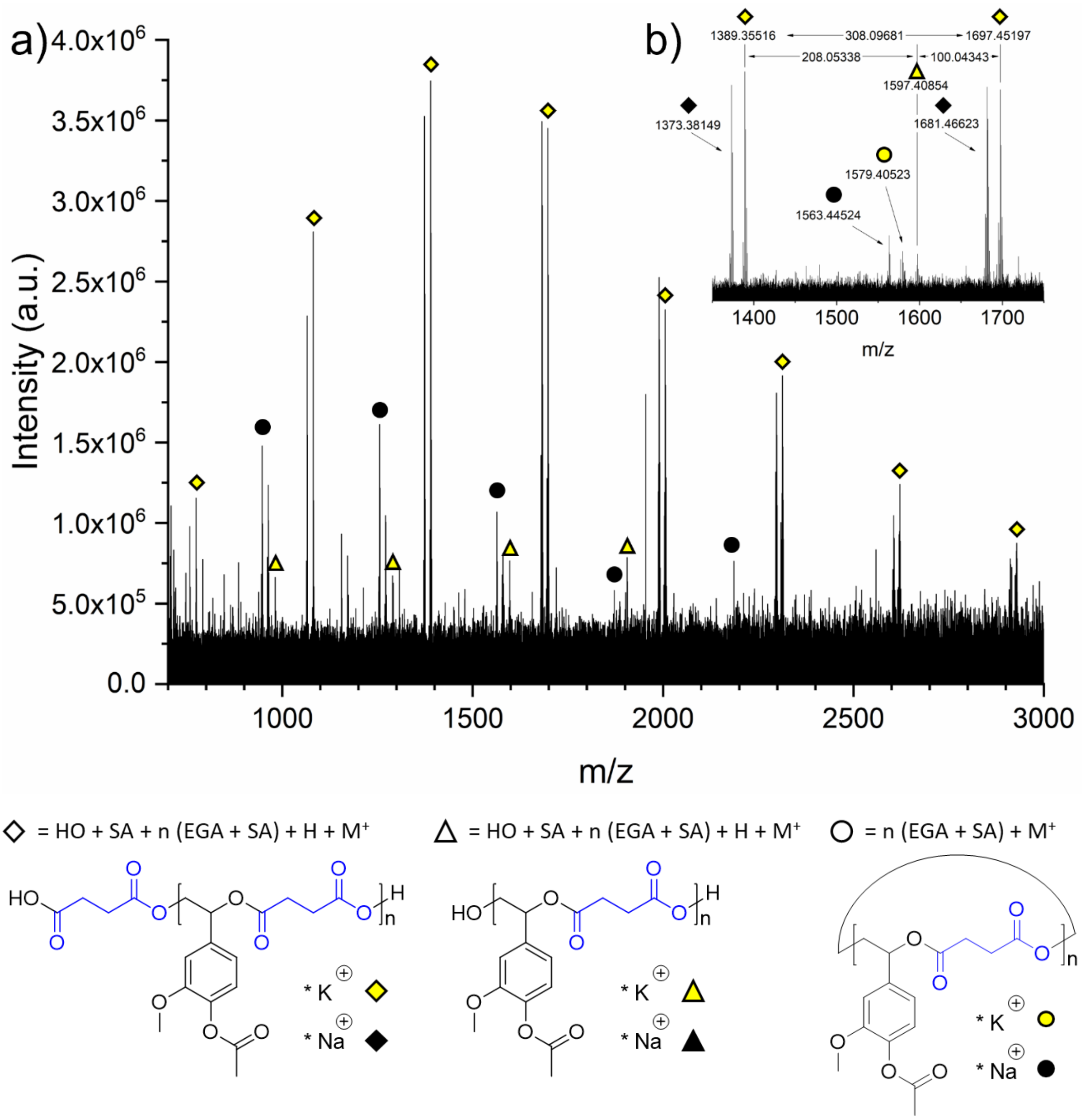

FigureS 120. High Resolution MALDI spectrum of P(EGA-*alt*-SA) as obtained in entry 10, Table 1. a) Region from 600 to 3000 m/z. b) Region from 1350 to 1750 m/z. Symbols ◇, △, and ○ indicate the species at the bottom.

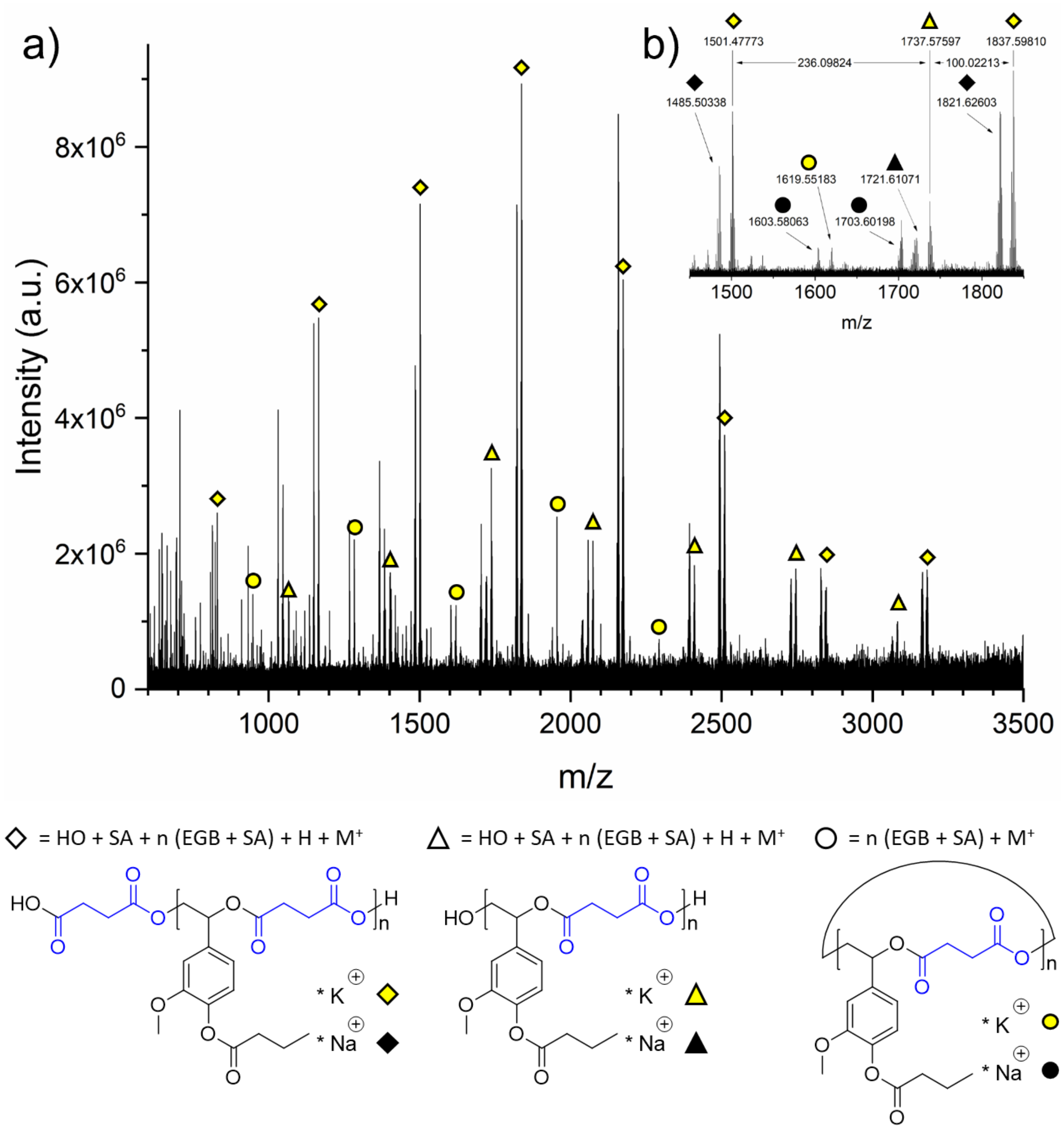

FigureS 121. High Resolution MALDI spectrum of P(EGB-*alt*-SA) as obtained in entry 11, Table 1. a) Region from 600 to 3500 m/z. b) Region from 1450 to 1850 m/z. Symbols ◇, △, and ○ indicate the species at the bottom. Sodium cations are labeled only in insert (b) for clarity.

9. DSC analyses of polymers

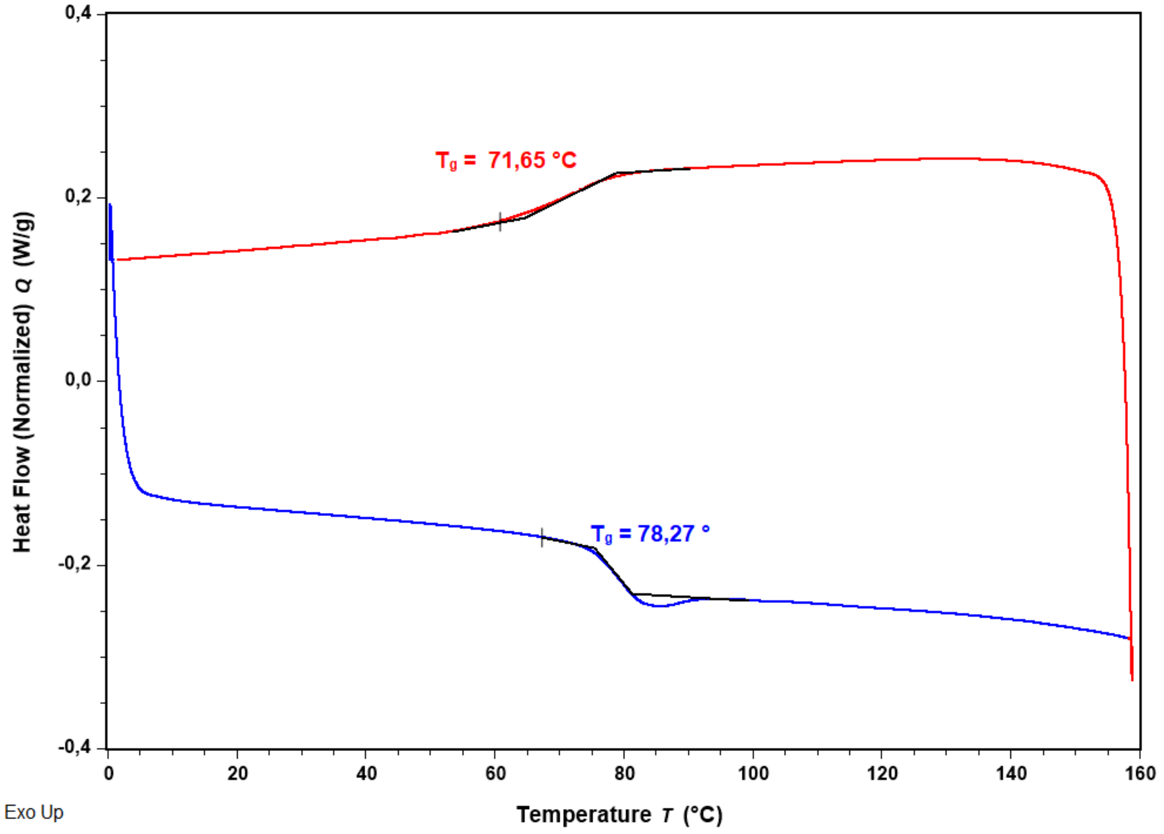

FigureS 122. DSC thermogram of P(EGA-*alt*-PA) prepared as in entry 23, Table S1.

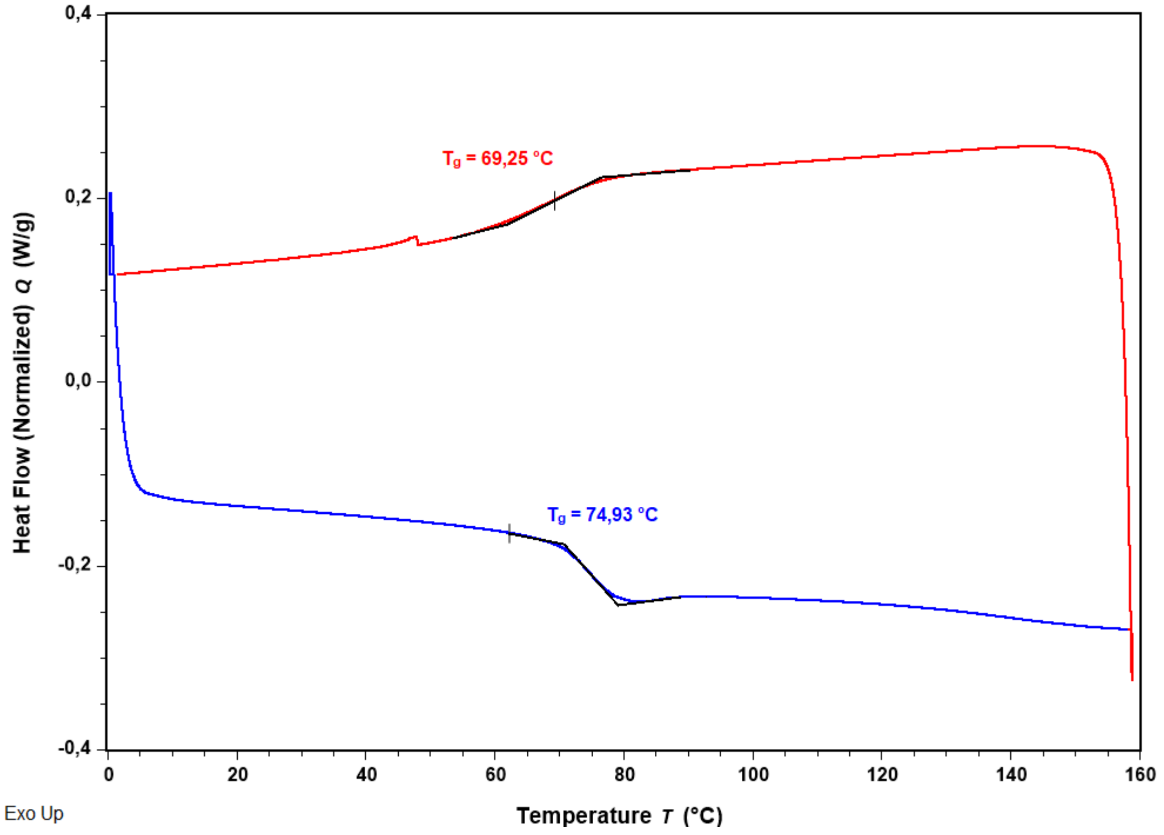

FigureS 123. DSC thermogram of P(EGA-*alt*-PA) prepared as in entry 1, Table 1.

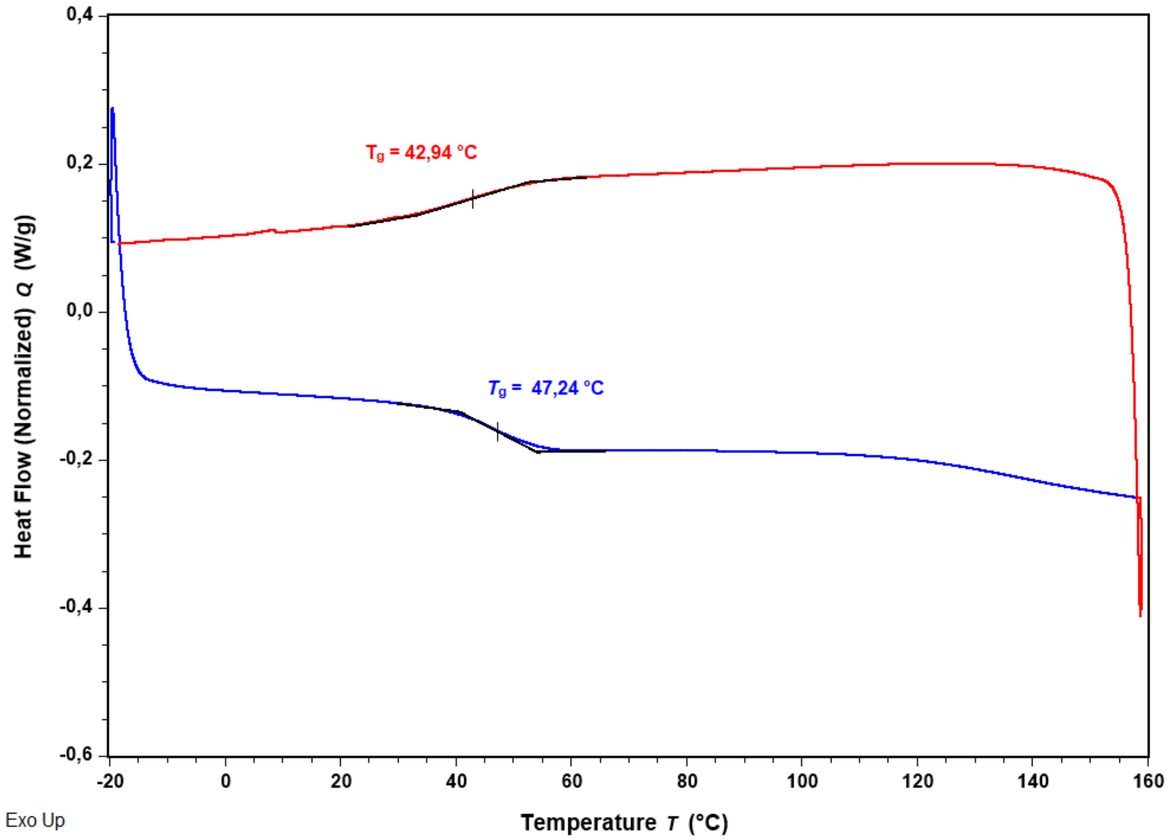

FigureS 124. DSC thermogram of P(EGB-*alt*-PA) prepared as in entry 2, Table 1.

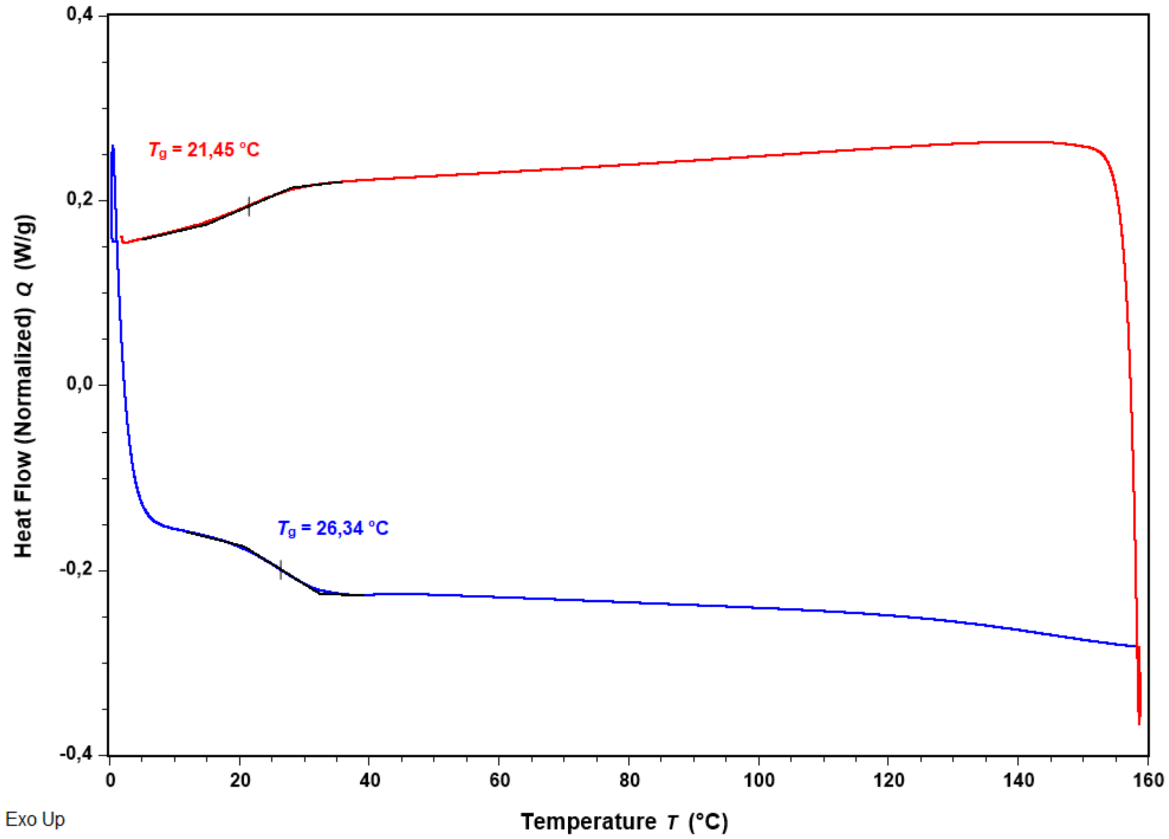

FigureS 125. DSC thermogram of P(EGH-*alt*-PA) prepared as in entry 3, Table 1.

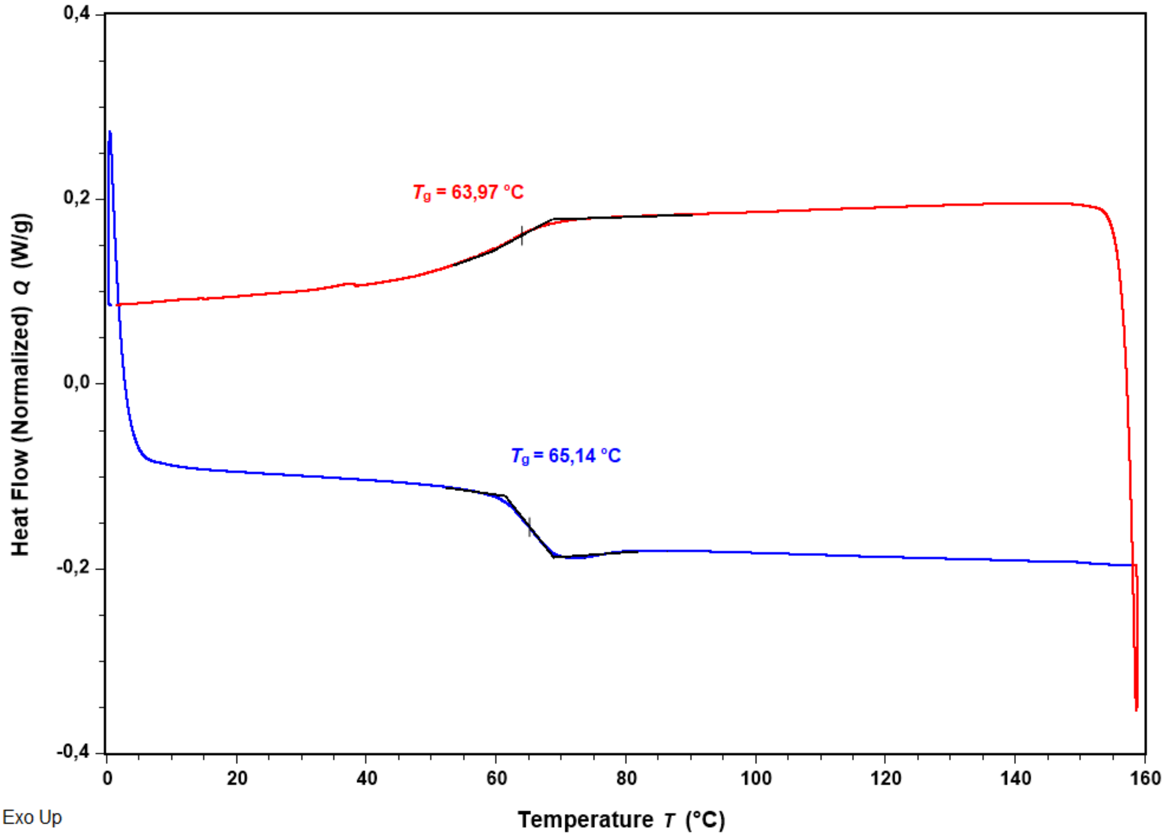

FigureS 126. DSC thermogram of P(EGA-*alt*-THPA) prepared as in entry 4, Table 1.

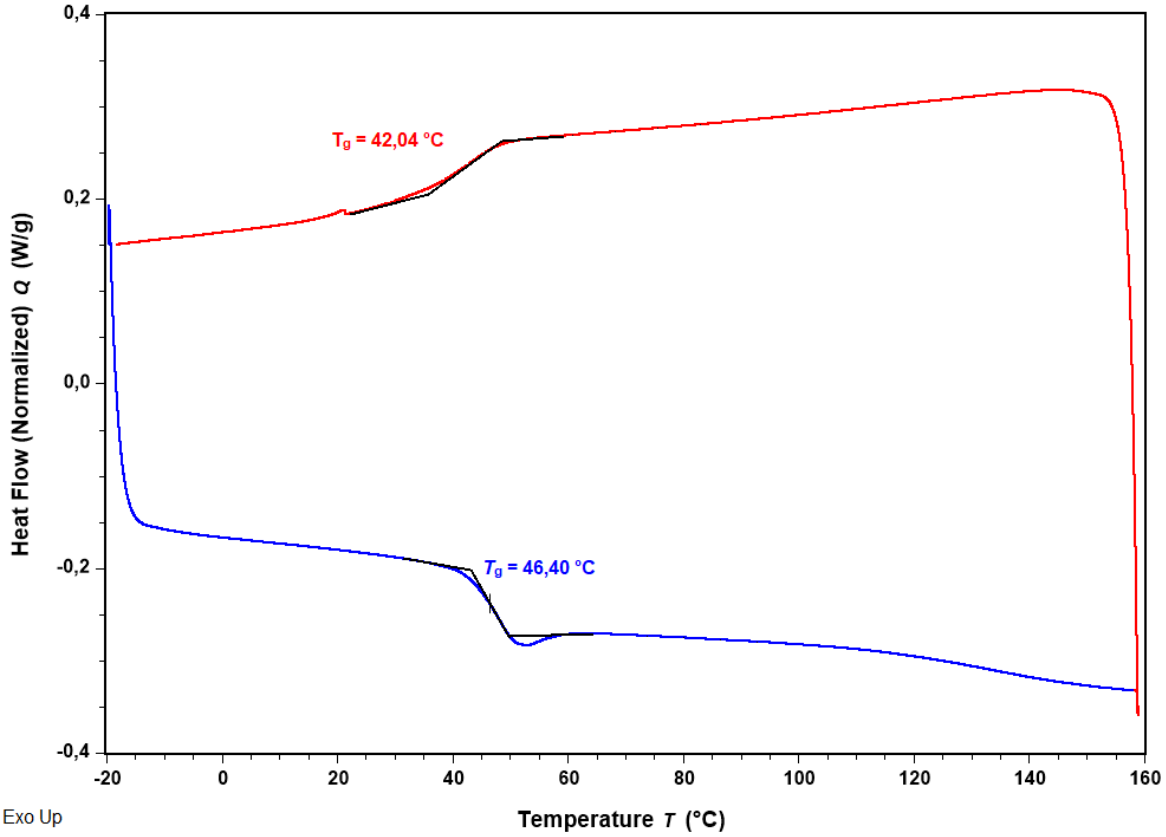

FigureS 127. DSC thermogram of P(EGB-*alt*-THPA) prepared as in entry 5, Table 1.

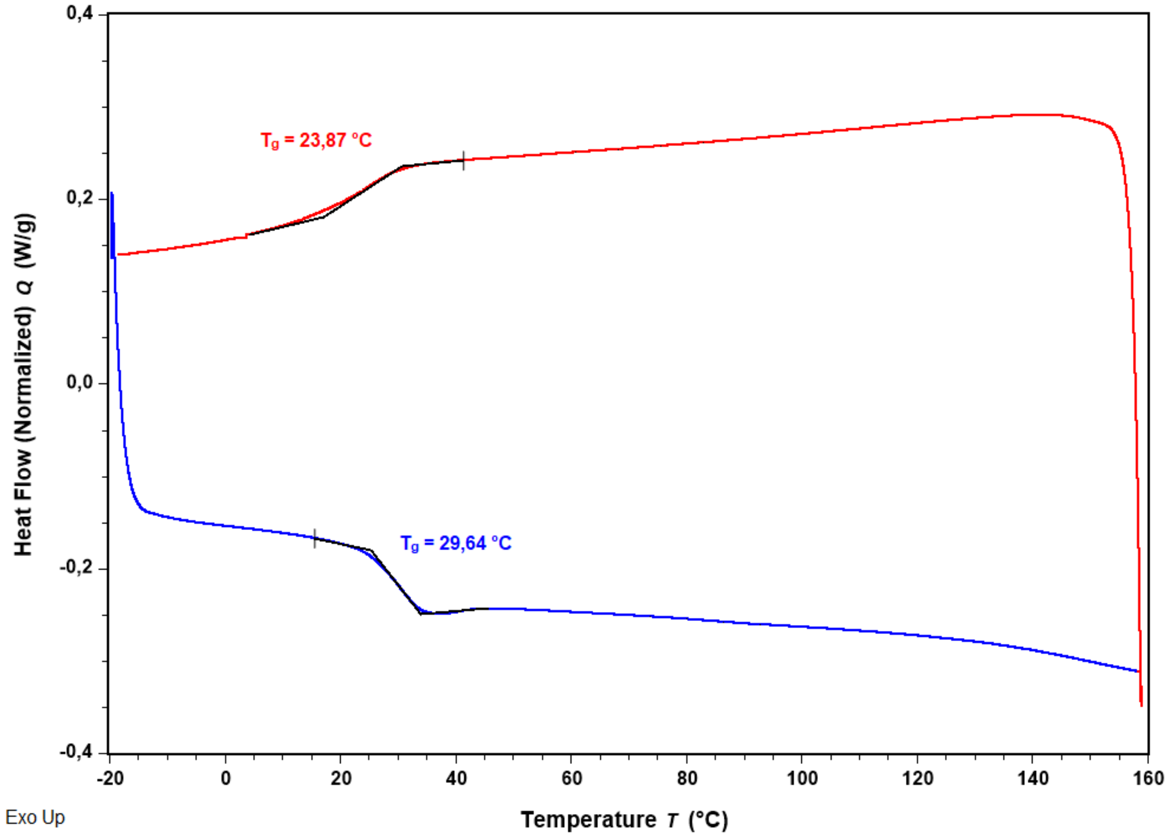

FigureS 128. DSC thermogram of P(EGH-*alt*-THPA) prepared as in entry 6, Table 1.

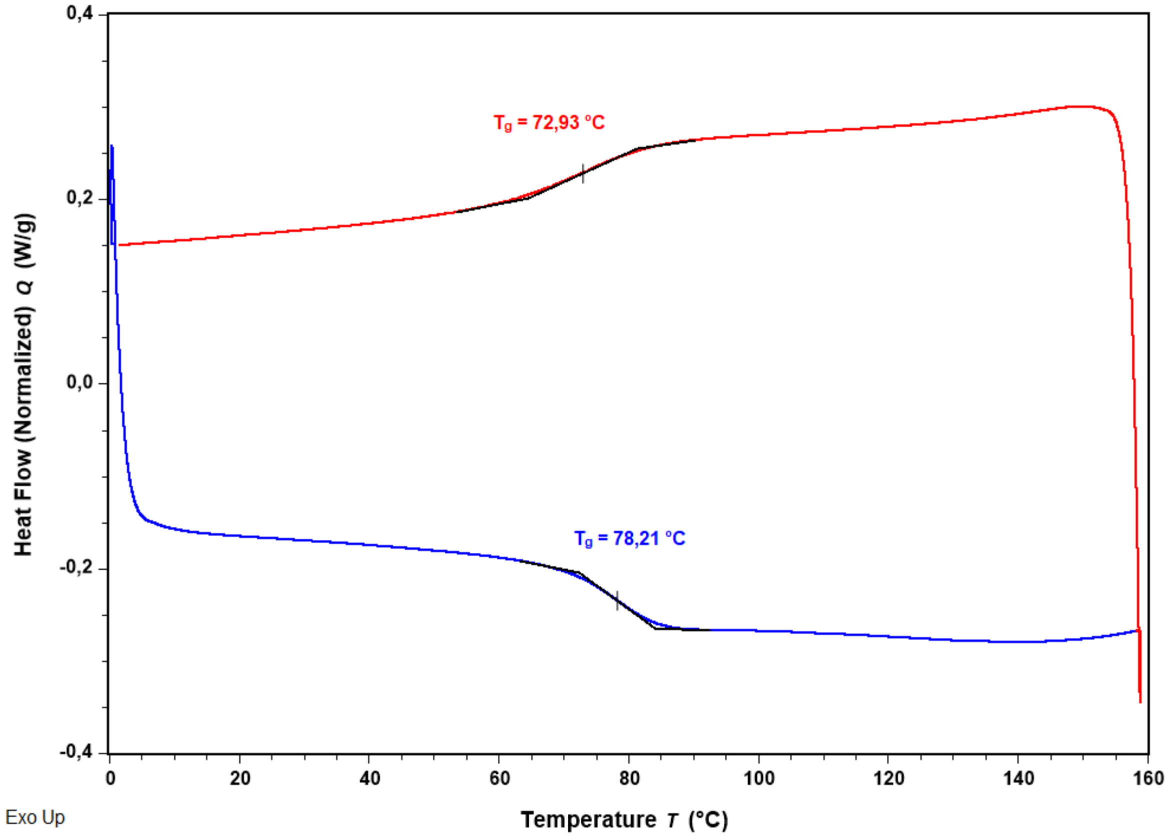

FigureS 129. DSC thermogram of P(EGA-*alt*-MA) prepared as in entry 7, Table Y.

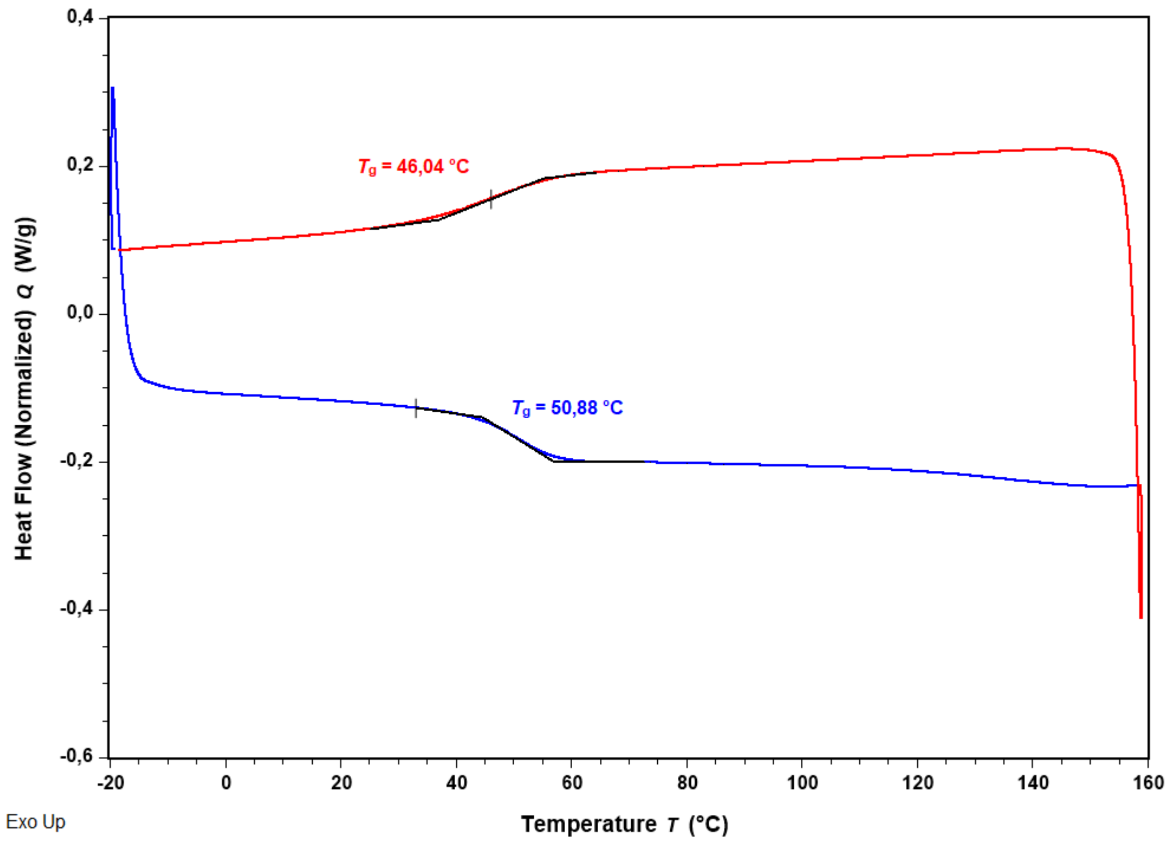

FigureS 130. DSC thermogram of P(EGB-*alt*-MA) prepared as in entry 8, Table 1.

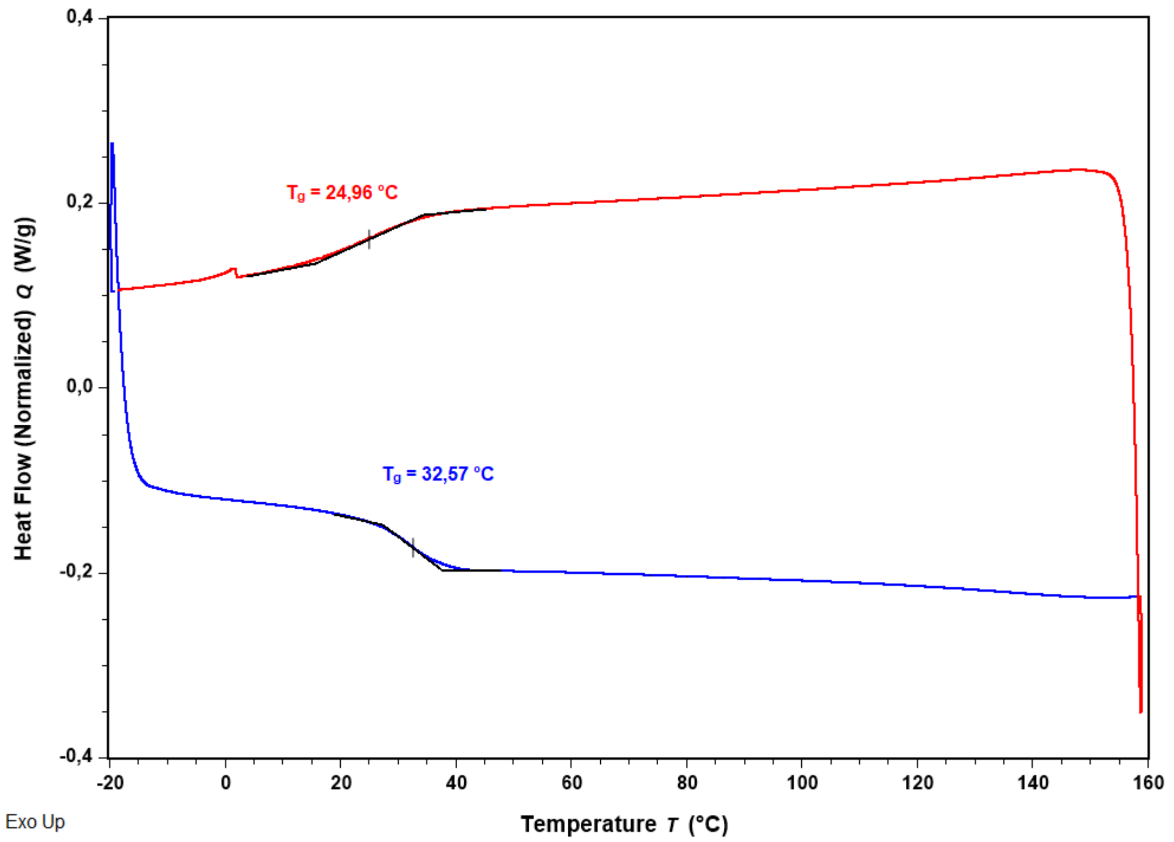

FigureS 131. DSC thermogram of P(EGH-*alt*-MA) prepared as in entry 9, Table 1.

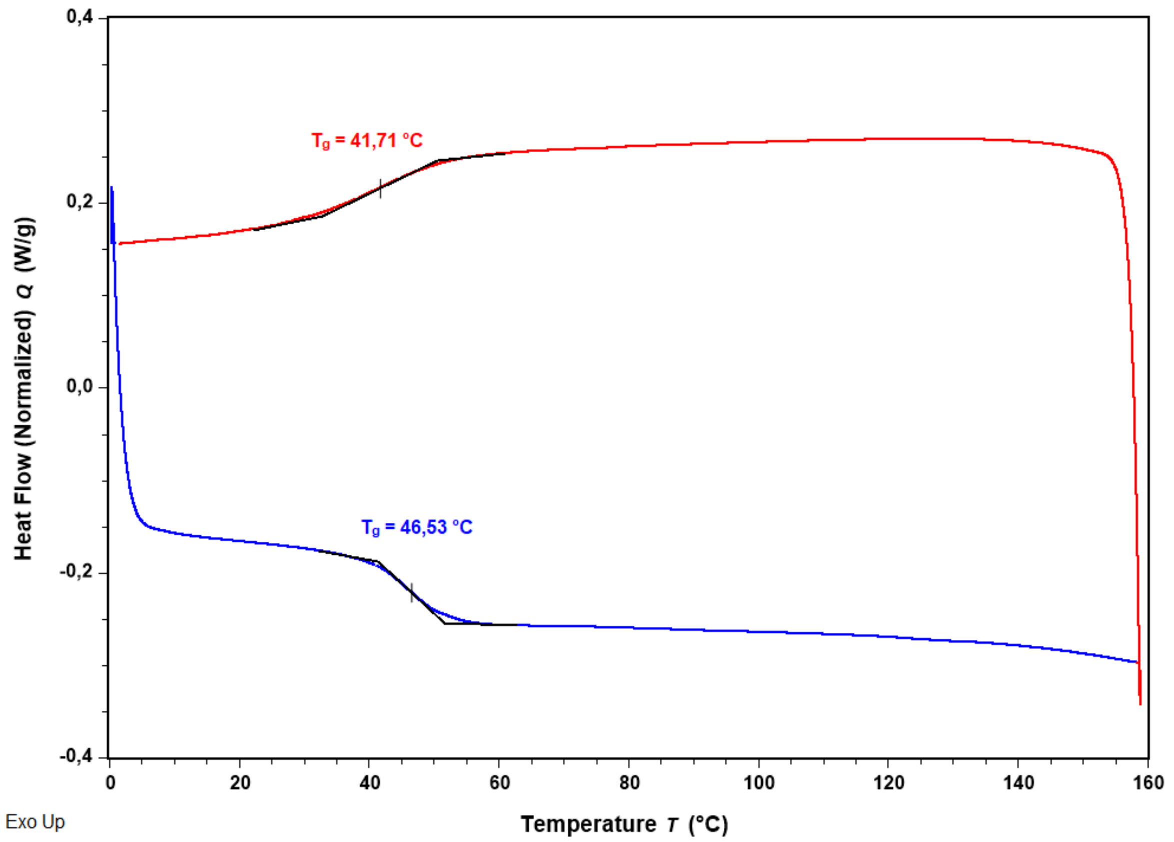

FigureS 132. DSC thermogram of P(EGA-*alt*-SA) prepared as in entry 10, Table 1.

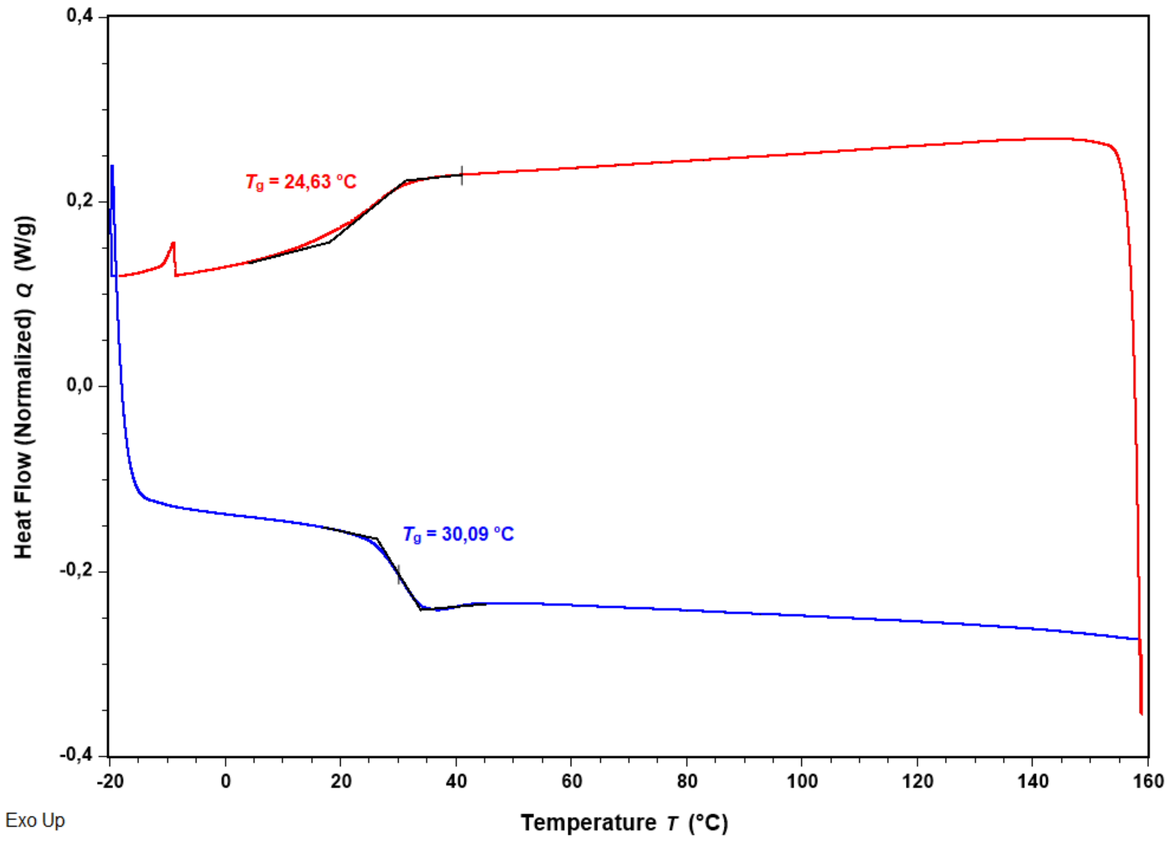

FigureS 133. DSC thermogram of P(EGB-*alt*-SA) prepared as in entry 11, Table 1.

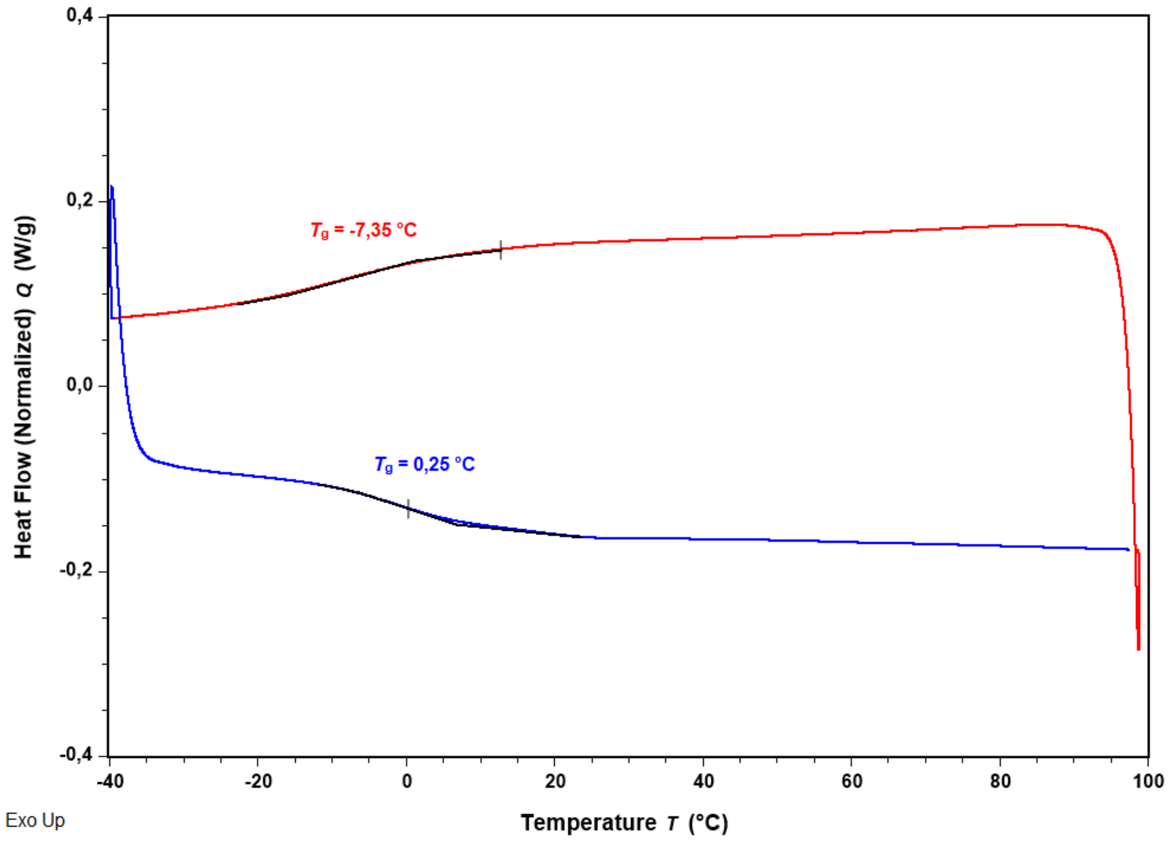

FigureS 134. DSC thermogram of P(EGH-*alt*-SA) prepared as in entry 12, Table 1.

References

[31] H. Takeshima, K. Satoh, M. Kamigaito, “Bio-Based Functional Styrene Monomers Derived from Naturally Occurring Ferulic Acid for Poly(vinylcatechol) and Poly(vinylguaiacol) via Controlled Radical Polymerization” *Macromolecules* **2017**, *50*, 4206–4216.

[38] E. Rigo, C. Totée, V. Ladmiral, S. Caillol, P. Lacroix-Desmazes, “4-Vinyl Guaiacol: A Key Intermediate for Biobased Polymers” *Molecules* **2024**, *29*, 2507.

[74] M. R. Monaco, D. Fazzi, N. Tsuji, M. Leutzsch, S. Liao, W. Thiel, B. List, "The Activation of Carboxylic Acids via Self-Assembly Asymmetric Organocatalysis: A Combined Experimental and Computational Investigation" *J. Am. Chem. Soc.* **2016**, *138*, 14740–14749.

[99] P. Petermeier, J. P. Bittner, T. Jonsson, P. Domínguez de María, E. Byström, S. Kara, "Integrated preservation of water activity as key to intensified chemoenzymatic synthesis of bio-based styrene derivatives" *Commun. Chem.* **2024**, *7*, 57.
